# Multidirectional characterization of cellular composition and spatial architecture in human multiple primary lung cancers

**DOI:** 10.1038/s41419-023-05992-w

**Published:** 2023-07-25

**Authors:** Yawei Wang, Di Chen, Yu Liu, Daiwang Shi, Chao Duan, Jinghan Li, Xiang Shi, Yong Zhang, Zhanwu Yu, Nan Sun, Wei Wang, Yegang Ma, Xiaohan Xu, Wuxiyar Otkur, Xiaolong Liu, Tian Xia, Huan Qi, Hai-long Piao, Hong-Xu Liu

**Affiliations:** 1 Department of Thoracic Surgery, Liaoning Cancer Hospital & Institute, Cancer Hospital of China Medical University, 110042 Shenyang, China; 2 CAS Key Laboratory of Separation Science for Analytical Chemistry, Dalian Institute of Chemical Physics, Chinese Academy of Sciences, 116023 Dalian, China; 3 Department of Thoracic Surgery, Affiliated Hospital of Qingdao University, 266000 Qingdao, China; 4 Department of Thoracic Surgery, Cancer Hospital of Dalian University of Technology, Liaoning Cancer Hospital & Institute, 110042 Shenyang, China; 5 Department of Pathology, Liaoning Cancer Hospital & Institute, 110042 Shenyang, China; 6 Department of Biochemistry & Molecular Biology, School of Life Sciences, China Medical University, 110122 Shenyang, China

**Keywords:** Cancer, Lung cancer

## Abstract

Multiple primary lung cancers (MPLCs) pose diagnostic and therapeutic challenges in clinic. Here, we orchestrated the cellular and spatial architecture of MPLCs by combining single-cell RNA-sequencing and spatial transcriptomics. Notably, we identified a previously undescribed sub-population of epithelial cells termed as *CLDN2*^+^ alveolar type II (AT2) which was specifically enriched in MPLCs. This subtype was observed to possess a relatively stationary state, play a critical role in cellular communication, aggregate spatially in tumor tissues, and dominate the malignant histopathological patterns. The CLDN2 protein expression can help distinguish MPLCs from intrapulmonary metastasis and solitary lung cancer. Moreover, a cell surface receptor−*TNFRSF18*/*GITR* was highly expressed in T cells of MPLCs, suggesting TNFRSF18 as one potential immunotherapeutic target in MPLCs. Meanwhile, high inter-lesion heterogeneity was observed in MPLCs. These findings will provide insights into diagnostic biomarkers and therapeutic targets and advance our understanding of the cellular and spatial architecture of MPLCs.

## Introduction

Lung cancer is one of the leading causes of morbidity and mortality due to cancer globally [1]. Smoking and atmospheric pollution are the major causes of lung cancer [2], and the presentation, symptoms, and pathology display extreme diversity [3]. Multiple primary lung cancers (MPLCs) [3] refer to several primary tumors growing synchronously in the lung. With the widespread of high-resolution computed tomography (HRCT), lung cancer screening has entered a new era in which MPLCs are being diagnosed more frequently. It has been reported that up to 15% of patients with lung cancer harbor a second primary lung lesion [4]. However, it is still difficult to distinguish MPLC and intrapulmonary metastasis (IPM) in the clinic, especially in cases of similar histologies.

Existing diagnostic criteria of lung cancer are mainly based on comprehensive histopathological assessment (CHA) [5] and next-generation sequencing (NGS) [6], which are insufficient to meet clinical requirements, especially in MPLCs. Therefore, significant efforts have been made worldwide to explore novel and accurate methods of identifying the complicated relationship between multiple separate lung tumor lesions.

Recently, single-cell RNA-sequencing (scRNA-seq) has been used to study the molecular and cellular atlas of lung cancer [7–9]. However, most of the studies were limited to single primary tumors and adjacent normal tissues, and lacked evaluation of spatially resolved molecular profiles and cellular landscape. In this study, to delineate the cellular composition and spatial architectural landscape of MPLCs, we performed high-resolution profiling of MPLC based on both scRNA-seq and spatial transcriptomics (ST). We characterized 133,923 cells from 19 tissue samples and integrated them with 37,616 spatial transcriptome spots from 12 tissue samples in four MPLC patients. We also investigated multiple independent scRNA-seq and bulk RNA-seq datasets to help contrast and validate key characteristics of MPLCs. In general, we described the cellular and spatial characteristics as well as the high inter-lesion heterogeneity of MPLCs. Interestingly, one previously unrecognized epithelial cell sub-population termed as *CLDN2*^+^ AT2 cells were found to be specific in MPLCs. Furthermore, cellular and molecular features of different histopathological pattern regions in invasive MPLCs-lung adenocarcinomas (LUAD) were also described. To the best of our knowledge, this is the first study to orchestrate the cellular and spatial architecture and identify potential biomarkers and therapeutic targets in MPLCs by combining scRNA-seq and ST.

## Results

### Single-cell transcriptomics-driven overview of MPLCs cell compositions

**Fig. 1:**
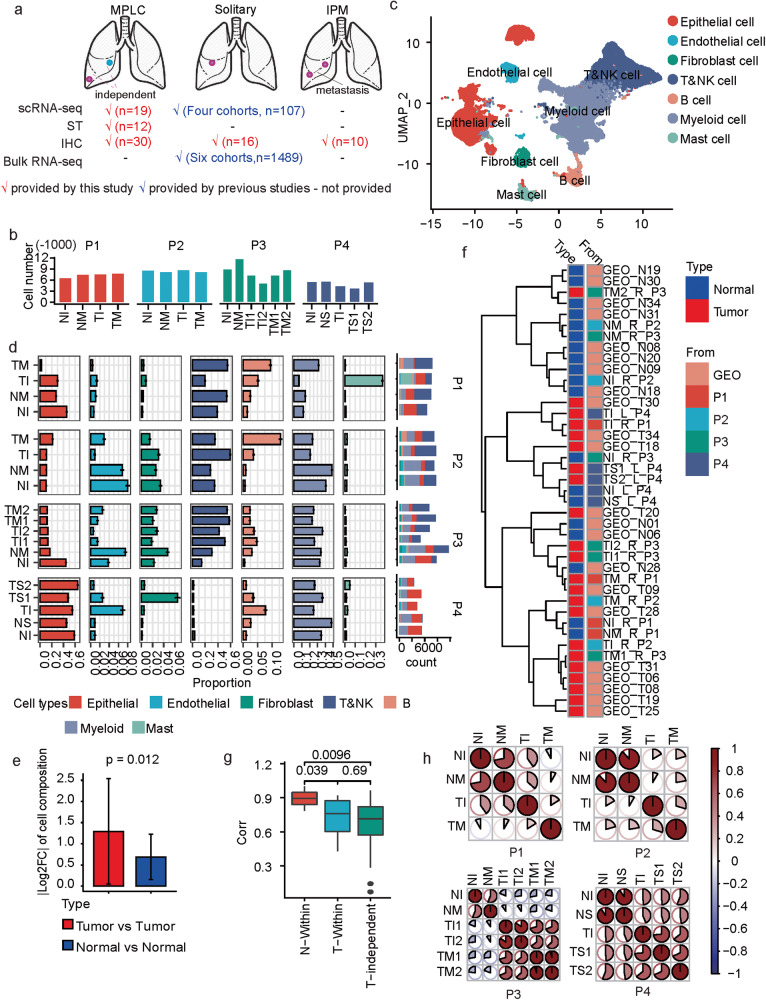
scRNA-seq based tumor heterogeneity overview of MPLCs. **a** Overview about the integrative data resources. **b** Number of cells in each sample after quality control. **c** Uniform Manifold Approximation and Projection (UMAP) of the 133923 cells based on scRNA-seq data. The cell types were annotated based on canonical cell type markers. **d** The proportions of different cell types within each measured sample. The *y*-axis represents the samples, and *x*-axis represents the percentage or total count. The proportions were estimated based on a bootstrap method, and represented in the form of mean ± s.d. The colors represent cell types. **e** The change of the cell type proportion between samples from different tumor lesions (red) or tumor-adjacent tissues (blue) within the same MPLCs patient. |·| means absolute value. Data represent mean ± s.d. *t* test, unpaired, one-sided. **f** Clustering both the samples collected by this study and the other LUAD tumor and adjacent normal tissue samples from an independent study (GEO dataset ID: GSE131907, *n* = 11 for both tumor and normal samples) based on the Spearman correlations between the cell type composition of two samples. See also Supplementary Fig. 2 for the clustering details. The sample names from GEO131907 begin with GEO, while the MPLC sample names begin with T or N. The letters L and R in the MPLC sample names represent left and right lung. **g** Boxplot of the correlations between different types of samples. *t* test, paired and one sided for comparing T-Within and N-Within, unpaired and two sided for the other comparisons. **h** The similarities between different samples within the same MPLCs patient. The similarity is based on the Spearman correlation coefficients between the average expressions of variable genes in two samples. MPLC multiple primary lung cancer, IPM intrapulmonary metastasis, ST spatial transcriptomics, IHC immunohistochemistry, TI tumor sample from the inferior lobe, TM tumor tissue from middle lobe, TS tumor tissue from superior lobe, NI normal tissue adjacent to TI, NM normal tissue adjacent to TM, NS normal tissue adjacent to TS, Log2FC log2-transformed fold change. N-within: correlations between normal samples within the same MPLCs patient. T-within: correlations between different tumor lesions within the same MPLCs patient. T-independent: correlations between different tumor tissues from different LUAD patients from GSE131907. corr: Spearman correlation coefficient between the cell type compositions of two samples.

To investigate cell heterogeneity and building blocks of MPLCs at single-cell levels, we integratively analyzed multiple data resources including scRNA-seq and ST data of MPLC samples, scRNA-seq and bulk RNA-seq datasets of independent solitary lung cancer cohorts, as well as the immunohistochemistry (IHC) data about MPLCs, solitary LUAD and IPM samples (Fig. 1a), and interesting cellular and molecular patterns of MPLCs were identified.

Firstly, we collected 19 tissue samples from four MPLCs patients (one squamous carcinoma and three adenocarcinomas), and each patient had two separate lung tumor lesions (Supplementary Fig. 1a, the clinicopathologic data are presented in Supplementary Table 1). For the first two patients (P1 and P2), we collected one tissue sample from each tumor lesion. For the third patient (P3), to understand intra-lesion heterogeneity, two repetitive tissue samples from each tumor lesion were collected. For the last patient (P4), two tissue samples from one of the larger tumor lesion and one tissue sample from the other small tumor lesion were collected. As comparisons, one normal tissue sample adjacent to each tumor lesion was collected. scRNA-seq was performed on all the collected 19 samples (Supplementary Fig. 1a). After standard data processing and quality control (Methods), we obtained transcriptomic profiles of 133,923 cells in total, and about 5000–9000 cells in each sample (Fig. 1b).

Clustering the scRNA-seq profiles helps identify seven major cell types including epithelial, endothelial, fibroblast, myeloid, mast, B and T&NK cells (Methods, Fig. 1c and supplementary Fig. 1b,c). These samples showed marked differences in terms of cell type compositions (Fig. 1d), and the differences between separate tumor samples from the same patient were significantly higher than that between the normal samples (Fig. 1e). Comparing tumor and normal tissues, B cells showed a significantly higher average proportion in the tumor tissues, while epithelial and endothelial cells were relatively enriched in normal tissues (Supplementary Fig. 1d, e). Samples from P4 were with few T&NK cells, perhaps because the two lesions were both adenocarcinoma in situ (AIS) with less immune infiltration.To elucidate whether multiple primary tumor lesions in the same patient shared more similarities than independent tumor tissues from different lung cancer patients or not, we integrated with the scRNA-seq data of solitary LUAD and normal lung tissues from another lung cancer cohort (GSE131907) [10]. The cells in GSE131907 were also annotated into the seven cell types. We clustered the 19 MPLCs samples and the 22 tumor and normal lung samples collected in GSE131907 based on the cell type composition similarities (Supplementary Fig. 2). We observed that the tissues from the same tumor lesion or two normal tissues of the same patient could be clustered together (e.g., TS1_L_P4 and TS2_L_P4, NI_L_P4 and NS_L_P4), but distinct tumor lesions of the same patient were apart (e.g., TI_R_P1 and TM_R_P1, Fig. 1f and supplementary Fig. 2). Meanwhile, the cell composition similarities between lesions within the same MPLCs patients (T-Within) were significantly lower than that between the matched normal samples (N-Within), but no significant difference was observed when compared to those between totally independent tumor tissues (T-independent) (Fig. 1g). Additionally, the tumor tissues from different lesions also showed less similarities than the normal tissues based on the gene expression profiles (Fig. 1h). In summary, MPLCs showed high inter-lesion heterogeneity in cellular compositions and gene expressions.

### Epithelial cell analysis identifies *CLDN2*^+^ AT2 as an MPLCs specific cell type

**Fig. 2:**
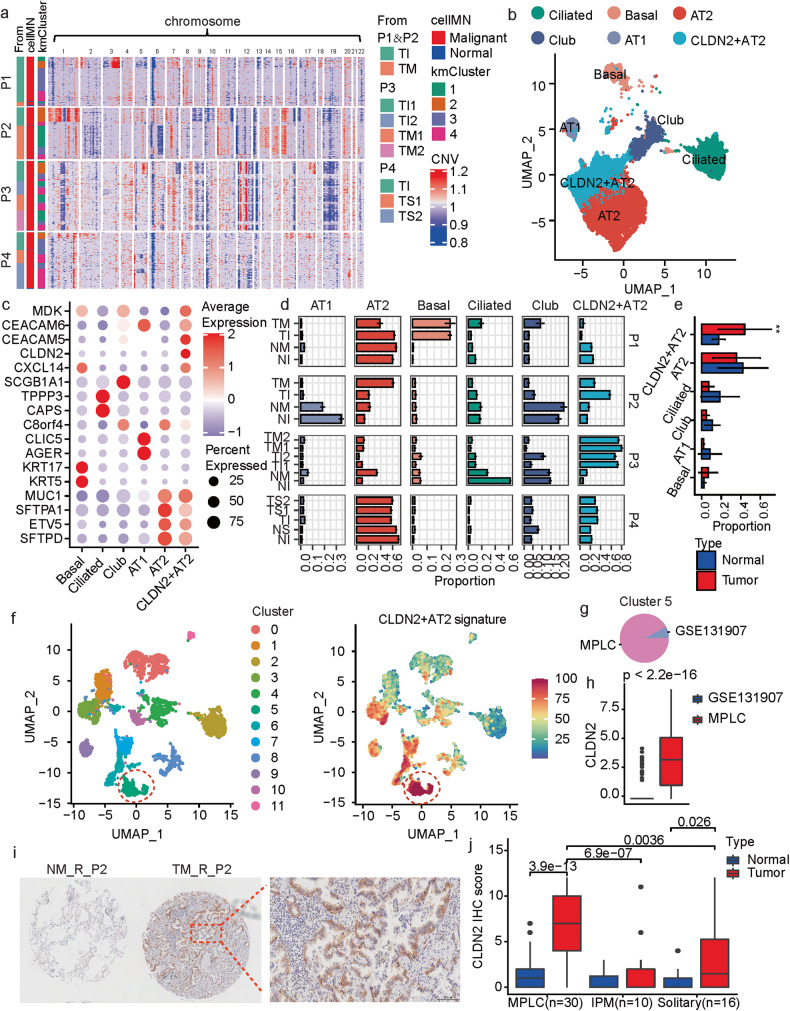
Subclassification and characterization of epithelial cells in MPLCs. **a** Heatmap of CNV profiles estimated from scRNA-seq of tumor lesions from each patient. The vertical axis is arranged by origin patients, samples, cell malignant or not, and on the CNV-based clustering results. The horizontal axis displays all chromosomes in numerical order. kmCluster: the k-means clustering results of epithelial cells based on the CNV profiles. cellMN: whether the cell is malignant or not. **b** UMAP plot colored by the sub-populations of epithelial cells. **c** Dot plot of the expression of marker genes for the epithelial cell sub-populations. **d** Bar plot of the bootstrap proportions of epithelial cell sub-populations within each measured sample. **e** The epithelial cell sub-population proportions in tumor lesions (red) or the adjacent normal tissues (blue) from the four MPLC patients. Data represent mean ± s.d. **P < 0.01, *t* test, unpaired. **f** UMAP view of the clustering results (left) and *CLDN2*^+^ AT2 signatures (right) of epithelial cells from both the MPLCs patients and the dataset GSE131907. Batch effects were removed by harmony. The *CLDN2*^+^ AT2 signature was calculated based on the cell-wise gene set variation analysis, and the top15 ranked (based on *P*-values) marker genes for the *CLDN2*^+^ AT2 subtype were utilized as the signature gene set. **g** Pie plot of the data source of cells in Cluster 5 in (**f**). **h** Box plot of the differential expressions of *CLDN2* between cells from the MPLC patients and GSE131907. Wilcox test, unpaired. **i** Immunohistochemistry (IHC) of CLDN2 expressions on the MPLCs samples. Samples NM_R_P2 and TM_R_P2 were taken as examples. Red border area was magnified and presented on the right. **j** Box plot of the differential CLDN2 IHC scores in MPLCs, IPM and solitary LUAD. Each IPM or MPLCs patient possessed two LUAD lesions at ipsilateral different lobes. Wilcox test, unpaired.

We first inferred chromosomal copy-number variations (CNVs) from the scRNA-seq data (with T&NK cells as reference) and identified the malignant and non-malignant epithelial cells (Methods). Most of the epithelial cells identified in the tumor tissues were malignant (Fig. 2a). The separate tumor tissues possessed different cellular CNV patterns. For instance, copy number gains in chromosome (Chr) 3 were only observed in the tumor tissues from the inferior lobe lesion (TI) but not the middle lobe lesion (TM) of P1. For each patient, there was always one CNV-based cluster specific to one tumor lesion (Fig. 2a, kmCluster 2 was specific for TI in P1, P2, and P4, as well as for TI1 in P3). In conclusion, different tumor lesions in MPLC patients may originate from different chromosomal variations.

Next, sub-populations of epithelial cells were identified (Fig. 2b). These sub-populations showed similar data qualities (Supplementary Fig. 3a). In addition to common epithelial cell sub-populations including alveolar types I (AT1) and II (AT2), club cells, basal cells and ciliated cells expressing canonical epithelial markers (Fig. 2c), we defined one previously unreported sub-population annotated as *CLDN2*^+^ AT2 cells that showed high levels of *CLDN2*, *CXCL14*, *CEACAM5*, *CEACAM6* and *MDK*, as well as AT2 cell markers (e.g., *MUC1* and *SFTPD*, Fig. 2b, c). The collected samples showed high heterogeneity in the epithelial cell sub-population compositions (Fig. 2d). AT2 and *CLDN2*^+^ AT2 types dominated among these sub-populations (Fig. 2d). The basal cell is a candidate cell-of-origin for human lung squamous carcinoma (LUSC) [11]. Notably, specifically in P1, an MPLC-LUSC patient, the tumor tissues had a much higher proportion of basal cells than the normal tissues.

Claudin-2 (Cldn-2) is a tight junction protein that mediates paracellular water or ion transport and has emerged to play a role in cancer [12–14]. The *CLDN2*^+^ AT2 cells showed significantly higher proportions in tumor tissues than normal tissues (Fig. 2e), especially in the three MPLC-LUAD patients (Fig. 2d). However, the different tumor lesions from the same patient did not show a significant alteration in the *CLDN2*^+^ AT2 cell proportions (Supplementary Fig. 3b).

To explore whether the specificity of *CLDN2*^+^ AT2 cell type, we did an integrative analysis of the epithelial cells from GSE131907 and the four MPLCs patients, re-classified them into multiple clusters and calculated a *CLDN2*^+^ AT2 signature based on the top-15 marker genes (Fig. 2f). Cluster5 showed the highest *CLDN2*^+^ AT2 signature scores, and it was mainly composed of the MPLCs epithelial cells (Fig. 2g). Meanwhile, in the Cluster5, the MPLCs epithelial cells also showed significantly higher expressions in *CLDN2* than GSE131907 (Fig. 2h). Additionally, we also checked the other four scRNA-seq datasets about lung cancer or lung cells, and only limited number of cells showed high *CLDN2* expressions (Supplementary Fig. 3c–f). These results indicate that *CLDN2*^+^ AT2 cells may be one MPLCs-specific cell type.

Furthermore, IHC was applied to verify the expression of CLDN2 in paraffin-embedded tissues of the four MPLCs patients. CLDN2 was over-expressed in the tumor tissues of P2 and P3, and specifically located at the cytomembrane of AT2 cells (Fig. 2i, Supplementary Fig. 4a and Data 1). The low expressions of CLDN2 in samples of P1 and P4 maybe due to their relatively low *CLDN2* + AT2 cell proportions as shown in Fig. 2d. Moreover, we tested and compared the CLDN2 protein expression in a larger sample set including 30 MPLCs, 10 IPM and 16 solitary LUAD patients. Results showed the CLDN2 IHC scores were significantly higher in the tumor tissues of MPLCs than in IPM and solitary LUADs. Meanwhile, the expressions of CLDN2 were remarkably higher in the tumor tissues than normal ones in MPLCs (Fig. 2j).

The 425 paneled NGS was conducted in all the 80 tumor lesions from the above 30 MPLCs and 10 IPM patients (Supplementary Table 2). The shared and unique tumor-specific gene mutations between separate tumor lesions within the same patient were analyzed. Results showed that the shared mutations in MPLCs patients were significantly lower than in IPM (Supplementary Fig. 4b). It has been recognized that the number of shared mutations is important evidence to distinguish MPLCs from IPM [6]. Interestingly, the CLDN2 IHC scores were significantly reduced in samples with larger shared mutations (Supplementary Fig. 4c). Taken together, the enrichment of *CLDN2*^+^ AT2 as well as the CLDN2 protein expression are potential evidence to distinguish MPLCs from IPM.

### The *CLDN2*^+^ AT2 cells possess a stationary state

**Fig. 3:**
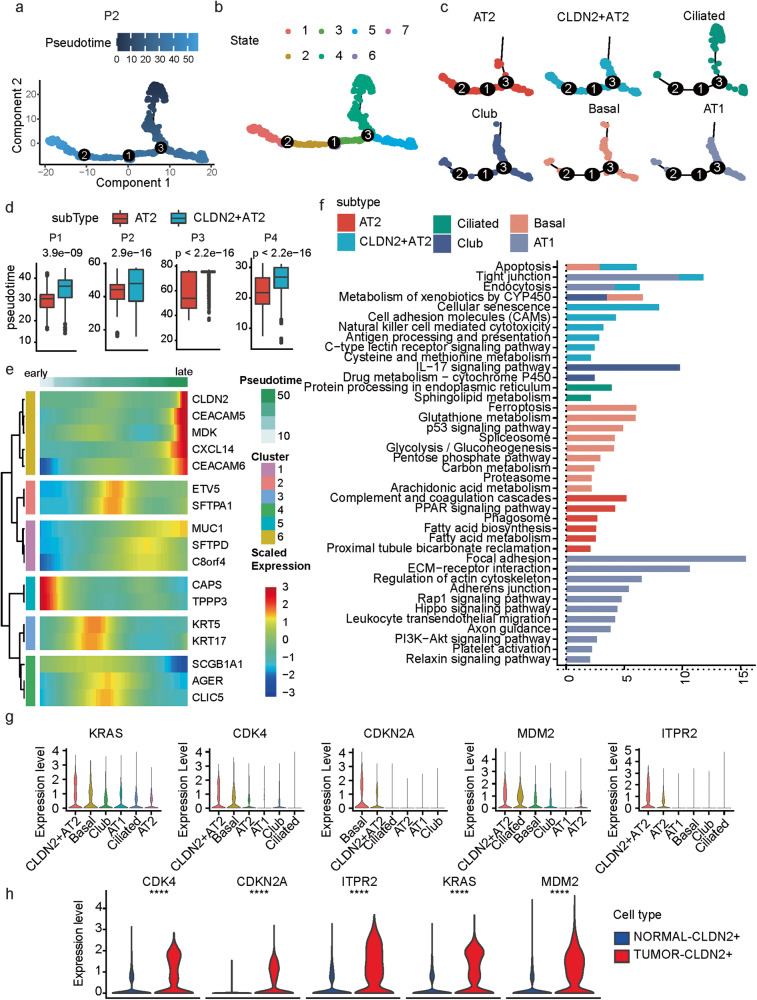
The *CLDN2*^+^ AT2 cells possess a stationary state. **a**, **b** Pseudotime trajectories of malignant epithelial cells in patient P2 are colored by the pseudotime (**a**) and states (**b**). **c** Pseudotime trajectories splitted by epithelial cell subtypes in patient P2. **d** Box plot of differences in pseudotimes between AT2 and *CLDN2*^+^ AT2 cells. Wilcox test, unpaired. **e** Heatmap of the pseudotime-dependent expression alterations of epithelial subtype marker genes. **f** Pathway enrichment results based on the marker genes of each epithelial subtype. Colors represent different subtypes. P: hypergeometric distribution. **g** Violin plot of the expressions of *CLDN2*-relevant cellular senescence marker genes in different epithelial cell subtypes. **h** Violin plot of the differential expression of *CLDN2*-relevant cellular senescence marker genes between *CLDN2*^+^ AT2 cells from the tumor and normal tissues. Wilcox test, unpaired. ****P < 0.0001.

To further characterize the epithelial subtypes, we performed pseudotime analysis for the epithelial cells. The cells were ordered along the predicted pseudotime trajectories, and different cell states were recognized accordingly (Fig. 3a, b, Supplementary Fig. 5a). In general, the AT2 and *CLDN2*^+^ AT2 cells were enriched by similar states which were different from the club, ciliated, basal and AT1 cells (e.g., for P1, AT2, and *CLDN2*^+^ AT2 cells were mainly from cell state 3 while the other subtypes were enriched by cell state 1 or 2; Fig. 3c, and Supplementary Fig. 5a). Comparing AT2 and *CLDN2*^+^ AT2 cells in the tumor tissues, we found that the *CLDN2*^+^ AT2 cells were with significant later pseudotimes than the AT2 cells (Fig. 3d). Meanwhile, the expressions of these subtype marker genes also changed with the pseudotimes where the expressions of the *CLDN2*^+^ AT2 marker genes including *CLDN2*, *MDK*, *CEACAM5*, *CEACAM6* and *CXCL14* increased significantly over the pseudotimes (Fig. 3e and Supplementary Fig. 5b).

Next, according to pathway enrichment analysis of the over-expressed subtype marker genes, we found different subtypes were mainly related to distinct pathways (Fig. 3f). Notably, the marker genes of *CLDN2*^+^ AT2 cells showed a specific enrichment in cellular senescence [15] (Fig. 3f), suggesting the *CLDN2*^+^ AT2 cells as one kind of senescent epithelial cells, just in agreement with the later pseudotimes of the *CLDN2*^+^ AT2 cells. The expressions of *CLDN2* were also significantly associated with five cellular senescence-associated genes including *ITPR2*, *MDM2*, *CDKN2A*, *KRAS* and *CDK4* (Pearson correlation coefficient > 0.4, Supplementary Fig. 5c). The expressions of *ITPR2*, *MDM2*, *KRAS* and *CDK4* were also highest in the *CLDN2*^+^ AT2 cells (Fig. 3g), and higher in the tumor-derived *CLDN2*^+^ AT2 cells than the normal ones (Fig. 3h). Meanwhile, the epithelial cells of solitary LUAD (based on GSE131907) showed significantly lower levels in the senescence signature than MPLCs (Supplementary Fig. 5d, e), suggesting the senescent character may be specific to the *CLDN2*^+^ AT2 cells of MPLCs. Cellular senescence refers to a state of irreversible cell cycle arrest [16], indicating the *CLDN2*^+^ AT2 cells were in a stationary state and cannot divide indefinitely and thus may prevent the metastasis of the cancer cells in MPLCs.

### The *CLDN2*^+^ AT2 cells play a critical role in cell–cell communications

**Fig. 4:**
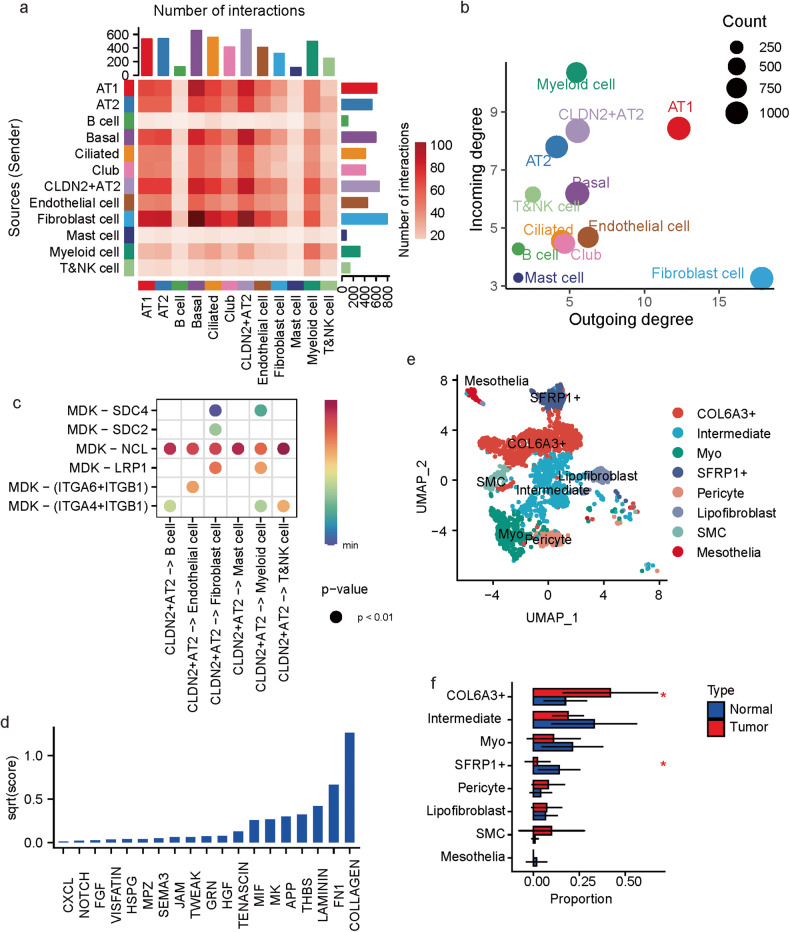
Cell–cell interactions in MPLCs. **a** Heatmap of the cell–cell interactions between different cell types. The row and column, respectively, stand for the source and target cell types of the interactions. The top and right colored bar plot respectively represent the sum of column and row of values displayed in the heatmap. **b** Scatter plot of the interaction strength of different cell types. **c** Bubble plot of the interactions originated from the *CLDN2*^+^ AT2 cells and mediated by MDK. **d** Bar plot of the summarized communication probabilities on signaling pathways based on the interactions from fibroblast cells to *CLDN2*^+^ AT2 cells. **e** UMAP plot of the subpopulations of fibroblast cells. Myo: myofibroblast, SMC: smooth muscle cells. **f** Bar plot of the average proportions of each fibroblast cell sub-population in tumor and tumor-adjacent tissues. Data represent mean ± s.d. *P < 0.05, *t* test, unpaired.

The intercellular communication was evaluated by CellChat [17] which modeled the cell–cell interactions based on both single-cell gene expressions and interactions between signaling ligands and receptors. Fibroblast cells were with the largest number of ligand–receptor interactions as signal senders (sender: ligand is over-expressed, receiver: receptor is over-expressed), while *CLDN2*^+^ AT2 cells possessed the largest number of interactions as the signal receivers (Fig. 4a). Considering the cell communication network topology, fibroblast and myeloid cells respectively had the largest outcoming and incoming degrees, and the *CLDN2*^+^ AT2 cells displayed the third highest incoming degree next to the myeloid and AT1 cells, and displayed higher incoming and outcoming degrees than the AT2 cells (Fig. 4b).

As the signal senders, the *CLDN2*^+^ AT2 cells mainly interacted with the other cell types via over-expressed ligands like MDK and APP (Fig. 4c and Supplementary Fig. 6a). As the signal receivers, the *CLDN2*^+^ AT2 cells mainly received the signals from fibroblast cells (Fig. 4a), of which the over-expressed ligands were mostly collagens (Fig. 4d and Supplementary Fig. 6b). Further clustering of the fibroblast cells based on the scRNA-seq profiles identify eight subtypes (Fig. 4e), and one subtype showed over expressions in multiple collagens such as COL6A3, COL6A2 and COL1A2 (Supplementary Fig. 6c). Since the interactions from fibroblast cells to *CLDN2*^+^ AT2 cells were mostly mediated by over-expressed collagens, the *COL6A3*^+^ fibroblast cells may be the main signal source of *CLDN2*^+^ AT2 cells. Besides, like the *CLDN2*^+^ AT2 cells, this *COL6A3*+ fibroblast subtype was also more significantly enriched by the tumor tissues than the normal ones (Fig. 4f).

### Elucidation of the spatial architecture of MPLCs by integration of ST with scRNA-seq

To further understand the spatial organization of different cell types and relevant heterogeneity features of MPLCs, ST was applied on 12 samples, including paired tumor and tumor-adjacent normal tissues from each of the two tumor lesions of the first three MPLCs patients (P1-P3, Fig. 1a). After standard data processing and quality control, each sample had about 2000–4000 spatial spots (Supplementary Fig. 7a, b). Then, the ST data were integrated with the scRNA-seq data using an anchoring-based integration method (Methods), which transferred the annotation information defined by the scRNA-seq data to the spatial spots. Every spot was assigned a dominant cell type based on the probabilities of being each cell type.

**Fig. 5:**
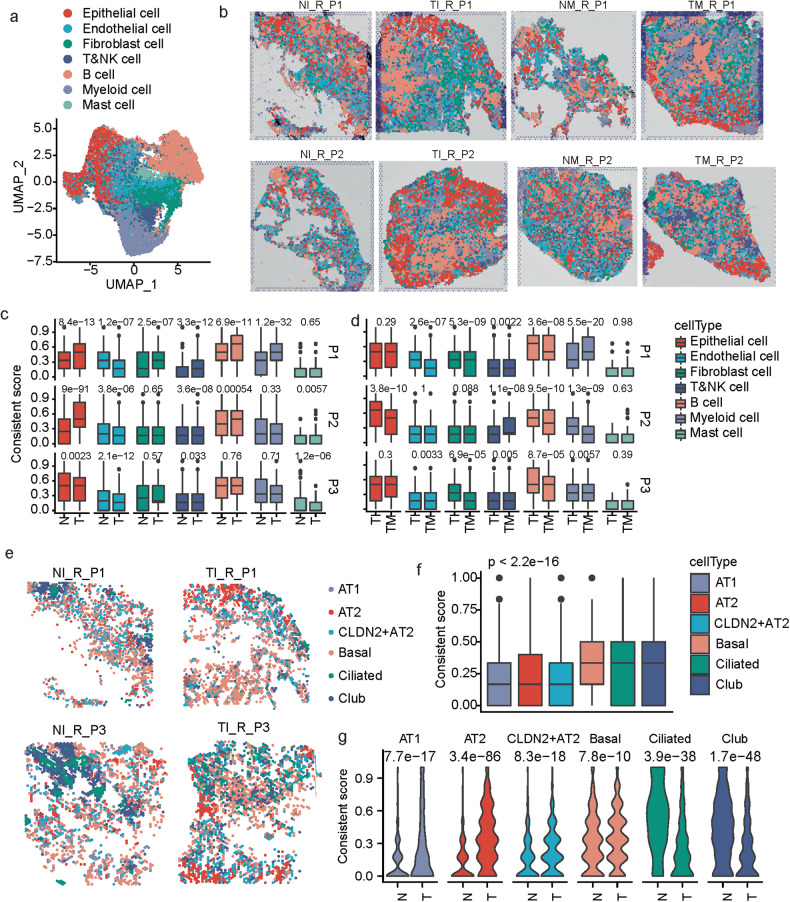
Spatial features of different cell types in MPLCs. **a** UMAP plot of 37616 spatial spots colored by cell types. The cell types of each spot were predicted by integrating the ST-seq data with the scRNA-seq data. **b** Spatial RNA-seq barcoded spots of the samples from patients P1 and P2, labeled by the predicted cell types. **c** Box plot of the difference of consistent scores of spots from tumor and normal tissues of the same patient in terms of different dominant cell types. **d** Box plot of the difference of consistent scores of spots from different tumor lesions of the same patient in terms of different dominant cell types. **e** Spatial spots colored by the predicted dominant epithelial cell subtypes. Four samples were displayed as examples. **f** Box plot of the difference of consistent scores of the spatial spots dominated by different epithelial cell subtypes. **g** Violin plot of the difference of consistent scores of spots from tumor and normal tissues in terms of different dominant epithelial cell subtypes. Kruskal test, unpaired.

Our results showed that the spatial spots were also annotated by seven major cell types (Fig. 5a, b and Supplementary Fig. 7c). That epithelial or B cells prefer to aggregate together spatially in the tumor tissues rather than the normal tissues (Fig. 5b). To analyze the aggregated trend of each cell type quantitatively, we designed one consistency score, which calculated the proportion of each spot’s direct neighbor spots that had the same cell type as the investigated one. The epithelial- and B cell-dominated spots had remarkably higher consistency scores in the tumor tissues than the normal tissues as expected, and the endothelial spots showed opposite changes (Fig. 5c), suggesting that these cell types spatially re-organized during benign-malignant transformation. When comparing two different lesions from the same patient, the consistency scores for B, fibroblast and T&NK cells were also significantly different in the three patients (P1–P3); the other cell types also showed patient-specific changes (Fig. 5d and supplementary Fig. 7d), indicating the inter-lesion spatial heterogeneity distribution of the cell types in MPLCs.

In addition, we also examined which two cell types were more likely to be near each other in spatial (Methods, supplementary Fig. 8a, b). For example, there was a higher proportion of T&NK–myeloid pairs in the TM_R_P1 sample than the TI_R_P1 sample. On clustering these samples according to the proportion profiles of all the pairwise cell types, we found that not only were the normal and tumor tissues completely separated, but also different lesion tissues from the same patients were not clustered nearby each other (supplementary Fig. 8c). These differences between tumor and normal tissues were especially significant in terms of the co-localization between endothelial and several other cell types, i.e., endothelial–mast, endothelial–endothelial, endothelial–epithelial, and endothelial–myeloid, as well as B-mast (supplementary Fig. 8d).

The dominant epithelial subtypes were also annotated (Fig. 5e). The spatial consistent scores of these sub-types were also computed. The alveolar cells including AT1, AT2 and *CLDN2*^+^ AT2 cells showed significantly lower consistent scores than the basal, ciliated and club cells (Fig. 5f), indicating that the basal ciliated and club cells were more spatially aggregated than the alveolar cells. Meanwhile, all these subtypes showed significant changes in the consistent scores between the tumor and normal tissues in the MPLC patients. The alveolar cells showed remarkable higher levels of spatial aggregation in the tumor tissues, while the other three sub-types showed reversed alterations (Fig. 5g), these differences suggest again the remarkable alterations of cellular spatial architecture during benign-malignant transformation. Meanwhile, separate lesions of the same MPLCs patient also showed significant differences in spatial aggregations (Supplementary Fig. 8e).

### Spatial and cellular characterization of LUAD histopathological patterns

**Fig. 6:**
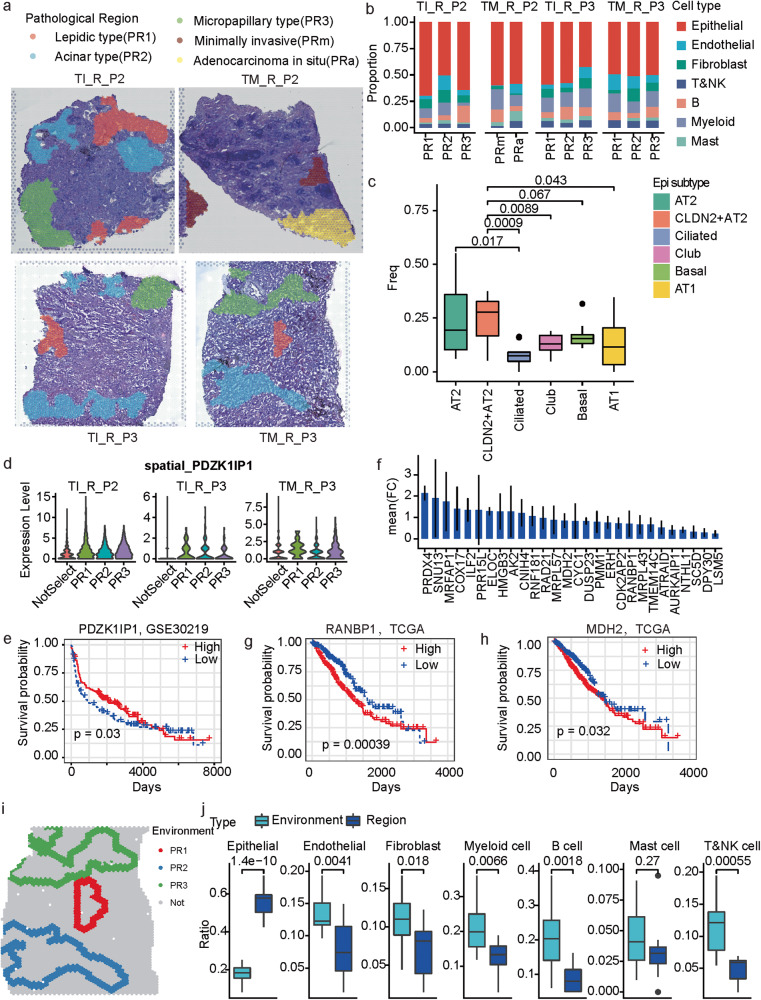
The cellular and molecular profiles of different histologic patterns in MPLC-LUAD. **a** Pathological classification of malignant regions into histologic patterns including lepidic type (PR1), acinar type (PR2), micropapillary type (PR3), minimally invasive (PRm) and adenocarcinoma in situ (PRa). **b** The main cell type compositions of the pathological regions across the four investigated samples. **c** Boxplot of the proportions of different epithelial cell sub-populations in the pathological regions across all the investigated samples. *t* test, unpaired. AT2 and *CLDN2*^+^ AT2 are, respectively, compared to the other sub-populations. **d** Violin plot of the spatially resolved high expression of *PDZK1IP1* in the region PR1. **e** KM-plot of the survival curves of patients in GSE30219. The patients were separated into two groups based on the expression of *PDZK1IP1*. **f** Bar plot of the mean fold change of PR3 marker genes shared by the three observed samples. **g**, **h** KM-plot of the survival curves of TCGA-LUAD patients. The patients were separated into two groups according to whether the expression of *RANBP1* (**g**) and *MDH2* (**h**) was higher than the median level. **i**. Spatial neighbors (the nearest three circles of spots) of the malignant regions. Colors represent different malignant regions. Sample TM_R_P3 is displayed as one example. **j** Box plot of the difference of cell compositions between the malignant regions and corresponding spatial neighbors. *t* test, unpaired.

In MPLC-LUAD, disease progression, and prognosis are associated with the appearance of morphologically diverse tumor regions [18, 19], termed histopathological patterns, and classified as lepidic, acinar, papillary, micropapillary, and solid [20]. The malignant regions in the MPLC-LUAD patients (P2 and P3) were further divided into lepidic (PR1), acinar (PR2), micropapillary (PR3), minimally invasive (MIA, PRm), and adenocarcinoma in situ (AIS, PRa) according to the latest WHO classification [21] (Fig. 6a). Mapping these regions onto the spatially resolved cell types revealed that although these regions were mainly composed of epithelial cells, as expected, there were also immune and mesenchymal cells, and the cell type proportions varied with both pathological sub-types and tumor tissues (Fig. 6b). Among the epithelial sub-populations, there was also marked heterogeneity in both histopathological pattern and in different samples (Supplementary Fig. 9a). Notably, the *CLDN2*^+^ AT2 cells showed higher proportions in the histopathological patterns than multiple sub-types (Fig. 6c), but AT2 cells simply showed significant higher proportions than the ciliated cells, which implies *CLDN2*^+^ AT2 cells represent a malignant epithelial sub-type.

For each of the individual histopathological region marked sample, we calculated the marker genes of each histopathological region based on ST data (Supplementary Fig. 9b and Supplementary Table 3). Comparing the marker genes of the same pathological region among different samples revealed that only one PR1 marker gene and 28 marker genes of PR3 were shared by the three samples (supplementary Fig. 9c). *PDZK1IP1* (also called *MAP17*) is a ROS-dependent oncogene [22], and its expression levels can predict the sensitivity of platinum-based therapy, EGFR inhibitors and proteasome inhibitors in LUAD patients [23]. Here, it was identified as one potential marker of lepidic pattern in MPLC-LUAD, as it showed higher expression in PR1 than PR2 and PR3 for all three samples (Fig. 6d). The high expression of PDZK1IP1 was associated with better prognosis in LUAD (Fig. 6e). The shared PR3 marker genes not only showed higher expression in the PR3 regions than the PR1 and PR2 regions (Fig. 6f), most of them also showed significantly higher expression in the tumor tissues than normal tissues based on the TCGA-LUAD bulk RNA-seq data (supplementary Fig. 9d). High expression of three of them, including *RANBP1*, *MDH2*, and *LSM5*, was associated with poor prognosis in LUAD (Fig. 6g, h and supplementary Fig. 9e). This is somewhat consistent with the fact that the LUAD micropapillary sub-type is associated with unfavorable prognosis clinically [24].

Additionally, the surrounding environment of the histopathologic patterns, i.e., the nearest three circles round the selected histopathological regions based on the spatial spots, were also examined (Fig. 6i). The surrounding environment was enriched by significantly different cell compositions from the histopathological regions (Fig. 6j), where less epithelial cells but more endothelial, fibroblast, myeloid, B and T&NK cells were observed. Furthermore, relatively more B, fibroblast, endothelial, and T&NK cells were observed in the other spatial regions (Supplementary Fig. 9f).

### Investigation of the molecular commonness and differences between different lesions in MPLCs patients

**Fig. 7:**
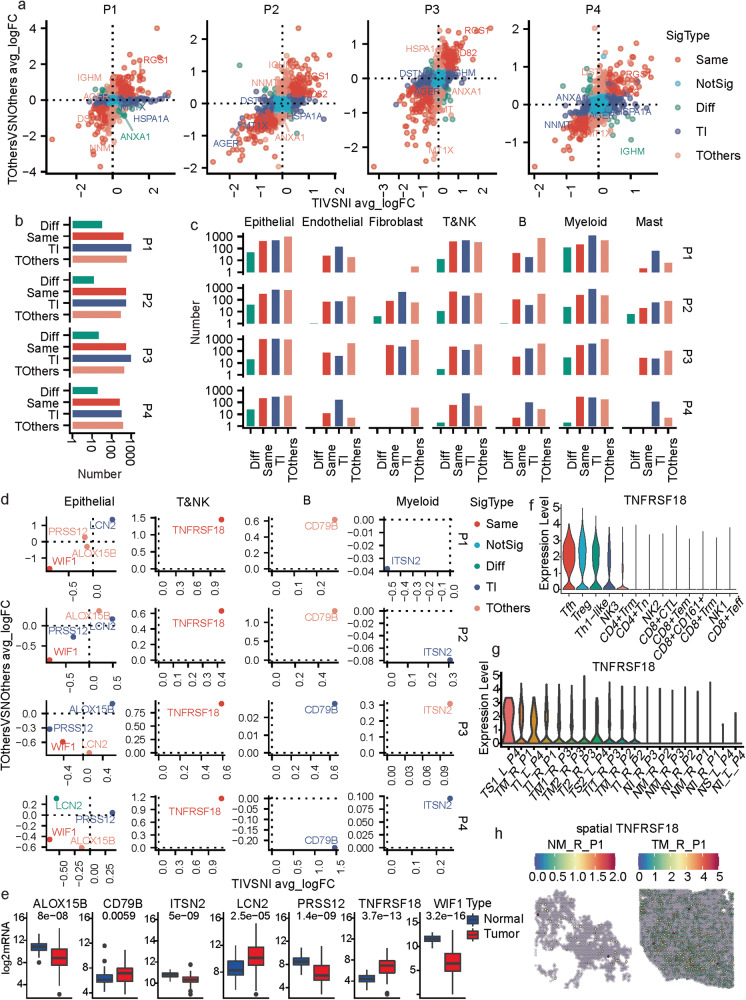
Molecular and cellular commonness and differences between lesions within the same MPLCs patient. **a** Scatter plot of the differential expressions of genes in the two different tumor lesions comparing to the corresponding adjacent normal tissues in each of the four MPLC patients. The *x*-axis represents the average log2FC (avg_logFC) computed by comparing the tumor and normal tissues in the inferior lobe (TIVSNI) of one patient. The *y*-axis represents the average log2FC (avg_logFC) computed by comparing the tumor and normal tissues in the other diseased lobe (TOthersVSNOthers) of one patient. The point colors represent the significance type. **b** Summary of the number of different significant types of genes identified based on each patient. **c** Summary of the number of different significant types of genes identified based on each cell type and each patient. **d** Seven Genes showed expressional change in all four MPLCs patients in certain cell types. **e** Box plot of the seven gene expressions in normal and tumor tissues based on the TCGA-LUAD dataset (*p*-value: kruskal test). **f** Violin plot of the expressions of *TNFRSF18* across T&NK cell subpopulations. **g** Violin plot of the expressions of *TNFRSF18* across all samples. **h** Spatially featured plot of the expressions of *TNFRSF18* in two samples of P1.

The gene expressional changes between cells from the tumor and tumor-adjacent normal tissues can help gain insights into the potential key molecular mechanisms underlying the development and progression of MPLCs. Each of the four patients had one tumor sample from the inferior lobe (TI); we first compared the gene expression profiles of cells from TI to its adjacent normal sample (TIVSNI), calculated the average log2FC (avg_logFC) and p-values, and performed this calculation for the other lesions (TOthersVSNOthers) from the same patient. Each gene was assigned two sets of differential expression analysis results, and the avg_logFCs calculated based on TIVSNI and TOthersVSNOthers were represented by the x and y axes, respectively (Fig. 7a). Scatters falling in the first and third quadrants mainly represented genes with similar expressional changes for the two lesions, while those in the other quadrants showed opposite changes. The genes were further classified into five types, including Same (different lesions show the same trends in the gene expressional changes), NotSig (not significant), OP (different lesions show opposite trends in gene expressional changes), TI (the gene expressional changes are specific to TI), and TOthers (the gene expressional changes are specific to TOthers). More genes were identified as OP, TI or TOthers types rather than the Same type (Fig. 7b). Notably, only two genes including regulator of G-protein signaling 1 (*RGS1*) and *CD82* were classified into the Same type for all four MPLCs patients with higher expression in tumor tissues than normal tissues (Fig. 7a). High expression of *RGS1* indicated favorable prognosis in LUAD (Supplementary Fig. 10a). *CD82* was reported to suppress lung cancer metastasis [25, 26] and its high expression was associated with poor LUAD prognosis. Therefore, the high co-expression of *RGS1* and *CD82* in MPLC lesions may serve as a diagnostic biomarker for MPLCs.

Cell-type-specific gene expressional alterations were also investigated (Supplementary Fig. 10b). Similarly, there were also more OP, TI, and TOthers types of changes than the Same type (Fig. 7c), indicating again the high molecular heterogeneity of different lesions of the same MPLCs patient. Various genes that were significantly changed, such as *CD79B*, *CLDN2*, *SPP1* and *IGHG3* (Fig. 7d and Supplementary Fig. 10b) were the marker genes of the identified cell types or sub-populations, probably owing to the high heterogeneity in cellular compositions across the tumor samples, as observed above. Regarding the expression of genes with concordant results across all four MPLCs patients (Fig. 7d), we noted that expression levels of *WIF1* and *TNFRSF18* were decreased or increased, in the epithelial and T&NK cells of both lesions, respectively. In addition, *ALOX15B*, *LCN2*, *PRSS12*, *CD79B* and *ITSN2* showed tumor lesion-specific changes for all four patients. The aforementioned seven genes also showed significant expressional changes between the tumor and normal tissues based on the TCGA-LUAD bulk RNA-seq data (Fig. 7e).

TNFRSF18, also known as glucocorticoid-induced TNF receptor (GITR), a cell surface receptor expressed by immune cells (mainly Tregs), acts as a key regulator in inflammatory and immune responses [27] and is an emerging molecular target in cancer immunotherapy [28, 29]. Notably, it showed significant higher expressions in the tumor tissues than the normal ones based on four independent LUAD cohorts (Supplementary Fig. 10c), and its high expression was associated with poor LUAD prognosis (Supplementary Fig. 10d). Among the T&NK cells of MPLCs samples, *TNFRSF18* showed high expressions in the Treg, Tfh, and Th1-like cells (Fig. 7f and Supplementary Fig. 11a–c). Meanwhile, it was constitutively expressed at higher levels in the T&NK cells of tumor tissues than normal tissues based on both the scRNA-seq data (Fig. 7g) and spatial transcriptomics (Fig. 7h). Therefore, TNFRSF18 can be a promising target in immunotherapy of unresectable MPLCs.

## Discussion

A diagnostic challenge in MPLCs is to distinguish MPLCs from intrapulmonary metastasis [3]. Meanwhile, the cellular and spatial characteristics of MPLCs are still unclear. Here, we constructed the cellular composition and spatial architecture of human MPLCs by integrating scRNA-seq with ST, and potential biomarkers were identified. Our study also investigated the molecular and cellular basis of MPLCs, and described the inter-lesion heterogeneity of MPLCs, thereby throwing lights on potential clinical diagnostic biomarkers and therapeutic targets of MPLC.

We showed that separate tumors from the same MPLCs patient were different from each other in cellular composition and gene expression profiles. Additionally, CNV analysis indicated that malignant epithelial cells within different tumor lesions were originated from different chromosomal variations, which provided a strong evidence of nonhomologous evolution as a determinant of MPLCs progression. By integrating with ST, we found that the spatial organization of multiple cell types were different between the separate tumor lesions of the same patient.

Defining the specific characteristics of MPLCs is also crucial for diagnosis and treatment in clinic. In this study, a major finding is that a newly identified *CLDN2*^+^ AT2 sub-type is specifically enriched in MPLCs. Besides the extra marker genes (*CLDN2*, *CXCL14*, *CEACAM5*, *CEACAM6,* and *MDK*), several remarkable features of *CLDN2*^+^ AT2 were identified. Firstly, *CLDN2*^+^ AT2 cells were more significantly enriched in the tumor tissues than the normal tissues, especially in three MPLC-LUAD patients. Secondly, the *CLDN2*^+^ AT2 cells possessed a stationary state as this type of cells showed significant later pseudotimes and high expressions of cellular senescence marker genes. This stationary state may prevent the metastasis of the cancer cells in MPLCs. Thirdly, the *CLDN2*^+^ AT2 cells play key roles in cellular communication, especially as signal receivers, where a COL6A3^+^ fibroblast cell type was its main signal source. Spatially, this *CLDN2*^+^ AT2 cell type was also significantly enriched in the malignant histopathological patterns of MPLCs. Moreover, this *CLDN2*^+^ AT2 cell type preferred to aggregate spatially in the tumor tissues. Intriguingly, none of the other five independent lung or lung cancer scRNA-seq datasets showed high *CLDN2*^+^ AT2 signatures. Meanwhile, the CLDN2 protein expression can help distinguish MPLCs from IPM and solitary LUAD. Taken together, the *CLDN2*^+^ AT2 cell may be an exclusive epithelial cell sub-type in MPLC patients. However, the latent mechanisms that driven the emergence of *CLDN2*^*+*^ AT2 cells and contribute to MPLCs need further experimental investigations.

In the last decade, high-throughput sequencing technologies have revolutionized lung cancer research by the whole genome genotyping [30], targeted NGS mutation detection [31], and high yield RNA sequencing [32]. NGS can help distinguish MPLCs from IPM based on whether the separate tumors harbored different driver mutations ^3^, however, direct diagnostic biomarkers were not determined. Integrating scRNA and ST, can not only help identify potential biomarkers or targets, but also contributes to a comprehensive cellular and spatial description of MPLCs at the single cell level. To our knowledge, this is the first study to investigate the specific cellular biomarkers in MPLCs by integrating scRNA-seq and ST.

The infiltrated immune cells are important constituents of the tumor microenvironment and adjuvant or neoadjuvant immunotherapy has greatly improved the prognosis of lung cancer [33, 34]. However, many patients still do not respond to anti-CTLA4 or anti-PD-1/PD-L1 blockade in clinical practice [35]. Here, we discovered a key regulator in inflammatory and immune response factor, *TNFRSF18* (*GITR*) [36–38], that was constitutively highly expressed in the T&NK cells of tumor tissues than normal tissues. TNFRSF18 was found highly expressed in Tregs of lung cancer tumor tissues which were non-responsive to anti-PD-1 therapy [39]. Recently, the first in-human phase-I trial of anti-TNFRSF18 (TRX518) was initiated in stage III or IV malignant melanoma (NCT01239134) [40]. Therefore, we would suggest that TNFRSF18 may be a potential target for immunotherapy in inoperable MPLC patients.

In addition, we also found that RGS1 and CD82 were co-over-expressed by all tumor lesions for all four MPLCs patients. RGS1 serves as a prognostic marker for poor outcomes in multiple types of cancers, including myeloma [41], gastric cancer [42], and B cell lymphoma [43]. However, in LUAD, we found that high expression of RGS1 was associated with a favorable prognosis. CD82, also known as KAI1, is an established metastasis suppressor in various malignancies, including lung cancer [44]. Therefore, the co-high-expression of RGS1 and CD82 implies less likelihood of the presence of IPM and may serve as a latent diagnostic biomarker for MPLCs.

Overall, our work reconciles molecular and phenotypic heterogeneity based on scRNA-seq and ST in MPLCs and demonstrates a new approach to identify the relationship between tumor lesions in multiple lung cancers. Our findings described here serve as a resource to better understand the development and progression of MPLCs, and the complex targeted therapeutic strategies. Moreover, the identified characteristics in molecular and cellular profiles are pertinent to the diagnosis of MPLCs.

## Materials and methods

### MPLCs Patients

Four patients with synchronous two lung lesions undergone R0 surgical resection in Liaoning Cancer Hospital & Institute between December 2020 and November 2021 were collected. None of them had received neoadjuvant radiotherapy, chemotherapy or immunotherapy before surgical resection. All patients were preoperatively diagnosed as MPLCs based on HRCT [45] by a multidisciplinary team (MTD) including a radiologist, thoracic surgeon, and oncologist. The multidimensional postoperative diagnostic criteria were based on CHA [5] and NGS [6]. The clinicopathologic characteristics and NGS data were respectively presented in Supplementary Tables 1 and 2. The project protocol was approved by the Institutional Ethics Committee of Liaoning Cancer Hospital & Institute before the initiation of the study. All patients provided informed consent for the use of the tumor tissues for research.

### Single-cell sample preparation

The resected tumor tissues or paracancer tissues were washed with phosphate-buffered saline (PBS) and completely immersed in MASC tissue storage solution (Miltenyi, Germany) at 4 °C. Single-cell suspensions were prepared according to 10x Genomics (US) Single Cell Protocols (CG000053, https://support.10xgenomics.com/).

### Single-cell RNA sequencing

The single-cell suspension was loaded into Chromium microfluidic chips with 3’ v2 chemistry and barcoded with a 10× Chromium Controller (10X Genomics, US). RNA from the barcoded cells was subsequently reverse-transcribed and sequencing libraries were constructed with reagents from a Chromium Single Cell 3’ v2 reagent kit (10X Genomics, US) according to the manufacturer’s instructions. Sequencing was performed with Illumina (US) according to the manufacturer’s instructions.

### Sample preparation for Visium spatial transcriptomics sequencing

Fresh tissues were concurrently frozen and embedded in optical cutting tissue (OCT) compound in solid carbon dioxide. The RNA quality of the OCT-embedded sample was assessed by Agilent 2100 (US). RNA integrity number (RIN) of samples greater than 7 were used for follow-up stRNA-seq. Cryosections (10 μm) were performed on a Leica CM3050 (Germany) and bright-field images were taken on a Leica Aperio Versa8 whole-slide scanner (Germany) at 20× resolution.

### Tissue optimization

The Visium Spatial Tissue Optimization Slide & Reagent kit (10X Genomics, US) was used to optimize permeabilization conditions for the tissues according to the Visium Spatial Tissue Optimization User Guide (CG000238, https://support.10xgenomics.com/). Briefly, the Visium Spatial Tissue Optimization workflow includes placing tissue sections on 7 Capture Areas on a Visium Tissue Optimization slide. The sections were fixed, stained, and then permeabilized at different times. mRNA released during permeabilization binds to oligonucleotides on the Capture Areas. Fluorescent cDNA was synthesized on the slide and imaged. The permeabilization time that results in maximum fluorescence signal with the lowest signal diffusion is optimal. If the signal was the same at two time points, the longer permeabilization time was considered optimal.

### Visium sequencing libraries preparation

The Visium Spatial Gene Expression Slide & Reagent kit (10X Genomics, US) was used to construct sequencing libraries according to the Visium Spatial Gene Expression User Guide (CG000239, https://support.10xgenomics.com/). A 10 μm frozen tissue section was placed on one of the Visium gene expression slide capture areas in a slide. After tissue Hematoxylin and Eosin (H&E) staining, bright-field images were acquired. Tissue permeabilization was performed for optimal minutes, as established in the Tissue Optimization procedure. Then reverse transcription experiment was conducted and sequencing libraries were prepared following the manufacturer’s protocol.

### Sequencing and raw data processing for ST

Sequencing was performed with a Novaseq PE150 platform according to the manufacturer’s instructions (Illumina, US) at an average depth of 300 million read-pairs per sample. For raw data processing, we used an in-house script to perform basic statistics, and evaluate the data quality and GC content along the sequencing cycles. Raw FASTQ files and histology images were processed by sample with the Space Ranger (version spaceranger-1.2.0, 10X Genomics, US). The filtered gene-spots matrix and the fiducial-aligned low-resolution image were used for down-stream analysis.

### DNA Extraction and Library Construction for Next Generation Sequencing

DNA was extracted by the QIAamp DNA FFPE Tissue KIT (QIAGEN, Germany) with modified protocols. The purified DNA is quantified by a Picogreen fluorescence assay using the provided lambda DNA standards (Invitrogen, USA). Then, library construction with the KAPA Hyper DNA Library Prep Kit (KAPA Biosystems, USA), containing mixes for end repair, dA addition and ligation, were performed in 96-well plates (Eppendorf, Germany). Dual-indexed sequencing libraries are PCR amplified for 4-7 cycles.

### Hybrid Selection and Ultra-deep Next Generation Sequencing of DNA

The PCR master mix is added to directly amplify (6-8 cycles) the captured library from the washed beads. After amplification, the samples are purified by AMPure XP beads, quantified by KAPA qPCR Library Quantification (KAPA Biosystems, USA) and sized on bioanalyzer 2100 (Agilent, USA). Libraries are normalized to 2.5 nM and pooled. Deep Sequencing is performed on Illumina HiSeq 4000 using PE75 V1 Kit. Cluster generation and sequencing is performed according to the manufacturer’s protocol.

### Immunohistochemical (IHC) analysis

CLDN2 protein expression was analyzed in tissue microarrays. Each MPLCs or IPM patient sampled 4 paraffin sections respectively from 2 tumor tissues and two corresponding adjacent tissues. Each solitary LUAD patient sampled 2 paraffin sections respectively from tumor tissues and adjacent normal tissues. The tissue microarrays were deparaffinized in xylene and dehydrated in gradient alcohol before antigen retrieval with an autoclave. Hydrogen peroxide (0.3%) was used to block endogenous peroxidase activity and nonspecific immunoglobulin-binding sites were blocked by incubation with normal goat serum for 30 min at 37 °C. The tissue microarrays were incubated with rabbit anti-CLDN2 (1:400, No.ab53032, Abcam) overnight at 4 °C. Then, the sections were incubated with biotinylated goat anti-rabbit IgG as a secondary antibody (Maixin Kit, China) for 1 hour at room temperature, followed by incubation with horseradish peroxidase-conjugated streptavidin-biotin (Maixin Kit, China) for 30 minutes at room temperature. The peroxidase reaction was developed with 3’-diaminobenzidine tetrahydrochloride (Maixin Kit, China).

### Semi-quantitative assessment and scoring

The CLDN2 IHC score = percent positivity × staining intensity. Cells were considered positive for CLDN2 if the cytomembrane was stained. The value of the percent positivity was defined as “0” if 0%, “1” if 1-10%, “2” if 11-50%, “3” if 51-80%, and “4” if >80%. The staining intensity was scored as “0” (no staining), “1” (weakly stained), “2” (moderately stained) and “3” (strongly stained). Both the percent positivity and the staining intensity were assessed by two doubly blinded investigators.

### Data processing, batch correction, cluster annotation, and data integration

The Seurat v.3.2.0 package was utilized for the scRNA-seq data analysis. Basically, cells with less than 250 unique genes or more than 25% mitochondrial transcripts or log10(nFeature_RNA)/log10(nCount_RNA) <= 0.75 and genes including MALAT1, mitochondrial genes, ribosomal genes and hemoglobin genes were excluded. Next, we utilized the NormalizeData and FindVariableFeatures functions with default parameters to do data normalization and choose the top-2000 variable genes. We used the ScaleData function to regress out the nFeature_RNA and percent_mito variable during scaling. Next, principal component analysis (PCA) was performed on the normalized and scaled gene expression matrix to do dimensionality reduction and the batch effects were removed by the harmony method based on the top 50 principal components from PCA (Seurat v3.2.0 and harmony v0.1.0 packages). UMAP was performed based on the harmony reduction space to do further dimension reduction. The cells were clustered on the harmony reduction space using the Lovain algorithm on k-nearest neighbors graph utilizing the FindNeighbors (reduction = ‘harmony’ and dims = 1:30) and FindClusters (resolution=0.5) functions. We used the FindAllMarkers function (logfc.threshold = 0.5, test.use = "wilcox", min.pct = 0.2, only.pos = TRUE, assay = "RNA") to get a list of differentially expressed genes for each cluster. Then, the clusters were annotated based on canonical cell type marker genes.

For the ST, spots with less than 250 unique genes or more than 20% mitochondrial transcripts or more than 1% hemoglobin transcripts and genes including MALAT1, mitochondrial genes, ribosomal genes, and hemoglobin genes were also excluded. Seurat package was also utilized for data normalization, dimensionality reduction, clustering and identification of cluster marker genes. Different from the scRNA-seq data, batch effects here were removed by the anchors-based integration method [46] of the Seurat package. We utilized the function FindIntegrationAnchors to identify anchors (pairs of cells from each dataset that are contained within each other’s neighborhoods) between all the collected samples, and then utilized the function IntegrateData to complete the dataset integration.

Integration of the scRNA-seq data and spatial transcriptomics was also performed by the anchors-based integration method ^48^. Firstly, we utilized the function FindIntegrationAnchors to identify anchors between the cells and spots from the scRNA-seq and ST. Then, the function TransferData was utilized to help transfer the cell type labels defined by the scRNA-seq data to the spots of the ST, and predicted probabilities of each cell type for the spots were saved as an assay and used for further analysis.

### CNV analysis

For each patient, the CNVs of cells from tumor tissue samples were estimated by the inferCNV [47] v.1.2.1 package where T&NK cells were used as the reference cells, and the epithelial cells of the tumor samples were used for the observations. To identify malignant and non-malignant epithelial cells, we clustered the reference and observation cells at the same time based on the inferred CNV profiles by the k-means method (k = 7), and epithelial cells grouped into the clusters dominated by the reference cells were defined as the non-malignant cells while the others were defined as the malignant ones. To compare the epithelial cell CNVs of the multiple lesions of the same patient, the CNV profiles of the epithelial cells were re-clustered by the k-means method (k = 4 here), and then lesion-specific clusters were identified by comparing the clusters enriched by each lesion.

### Pseudotime trajectory analysis

Firstly, we extracted the scRNA-seq data of the epithelial cells of the tumor samples. Next, we utilized the Monocle2 [48] package to determine the pseudotime trajectories of each patient separately, where the cell-gene expression matrix in the form of UMI counts was used as input of Monocle2. The estimateSizeFactors and estimateDispersions functions with default parameters were used to preprocess the UMI count matrix, and the detectGenes function with a parameter of min_expr = 0.1 was used to retain genes expressed in more than 10% cells. The DDRTree-based reduction was performed by the reduceDimension function of Monocle2 with the parameters of max_components = 2 and method = ‘DDRTree’. The cell ordering was performed by the orderCells function, and the state enriched by the basal cells was selected as the root state since basal cells are progenitors of the airway epithelium [49].

### Identification of the sub-populations of each major cell type

We identified the sub-populations of the epithelial and fibroblast cells. For each cell type, we extracted the scRNA-seq data of the corresponding cells, and re-analyzed these cells by performing data normalization, scaling, dimensionality reduction, batch effects correction, clustering and identification of cluster marker genes based on Seurat v.3.2.0 with the same parameters as described for analyzing the whole scRNA-seq dataset except that the resolutions for clustering epithelial and fibroblast cells were respectively 0.1 and 0.3. Then, the re-clustered groups were annotated based on the identified marker genes.

### ST-based consistency score and co-localization profile

For each spot, we obtained its direct neighbor spots based on the row and column coordinates of these spots arranged in a checkerboard pattern spatially, and calculated the proportion of spots with the same predicted cell type as the input one, and this was defined as the consistency score for the spot. Meanwhile, for all the spots of one sample, we identified all pairwise neighborhood spots and calculated the proportions of cell type pairs as the co-localization profile.

### Comparison of the gene expressional changes between the multiple lesions of the same patient

Each of the four patients had one tumor sample from the inferior lobe (TI), we first compared the gene expression profiles of cells from this tumor sample to its adjacent normal sample (TIVSNI), calculated the average log2FC (avg_logFC) and *p*-values using FindMarkers function of the Seurat package, and next did this calculation based on the other disease lesions (TOthersVSNOthers) from the same patient. Considering both avg_logFCs and *p*-values, the genes were further classified into five significant types including Same (p_TIVSNI < 0.01, p_TOthersVSNOthers <0.01, |TIVSNI avg_logFC| > 0.25, |TOthersVSNOthers avg_logFC| > 0.25, and in the first or third quadrants, i.e., with the same trends in the gene expressional changes for two lesions), NotSig (p_TIVSNI > 0.01 or p_TOthersVSNOthers > 0.01 or |TIVSNI avg_logFC| < 0.25 and |TOthersVSNOthers avg_logFC| < 0.25), OP (p_TIVSNI < 0.01 and p_TOthersVSNOthers <0.01, |TIVSNI avg_logFC| > 0.25, |TOthersVSNOthers avg_logFC| > 0.25, and in the second or fourth quadrants, i.e., with opposite trends in gene expressional changes for two lesions), TI (p_TIVSNI < 0.01, |TIVSNI avg_logFC| > 0.25, and not included in Same or OP, i.e., the gene expressional changes are specific to TI), and TOthers (p_TOthersVSNOthers <0.01, |TOthersVSNOthers avg_logFC| > 0.25, and not included in Same or OP, i.e., the gene expressional changes are specific to TOthers).

### Pathway enrichment analysis

Based on the marker genes of each subtype, we performed pathway enrichment by the enricher function of the clusterProfiler [50] v3.14.3 package, and pathway information was obtained from the KEGG database.

### Statistics

Detailed computational and statistical methods are reported in the Methods or figure legends. All statistical analyses were performed by R (v 3.6.3), and the *t* test in R was Welch *t* test by default which did not require the two samples have similar variances. The statistical tests were two-sided by default, and one-sided tests were specifically stated.

## Supplementary information

Supplementary materials

Table S1-MPLC

Table S2 NGS data

Table S3-selected Region markers.

Supplementary Data 1

check list

## Data Availability

The single-cell RNA sequencing and spatial transcriptomics data are available from GEO (accession codes GSE200972 and GSE200916).

## Code availability

The codes for this study are available from GitHub (https://github.com/diChen310/scRNA-ST_lung).
