# Supplementary material for: Multidirectional characterization of cellular composition and spatial architecture in human multiple primary lung cancers
Source: Cell Death Dis. 2023 Jul 25;14(7):462. doi: 10.1038/s41419-023-05992-w (PMC10366158; doi:10.1038/s41419-023-05992-w)
Supplement: Supplementary file 1 — Supplementary materials [file 41419_2023_5992_MOESM1_ESM.docx]

**Supporting Information**

**Supplementary figures**

**
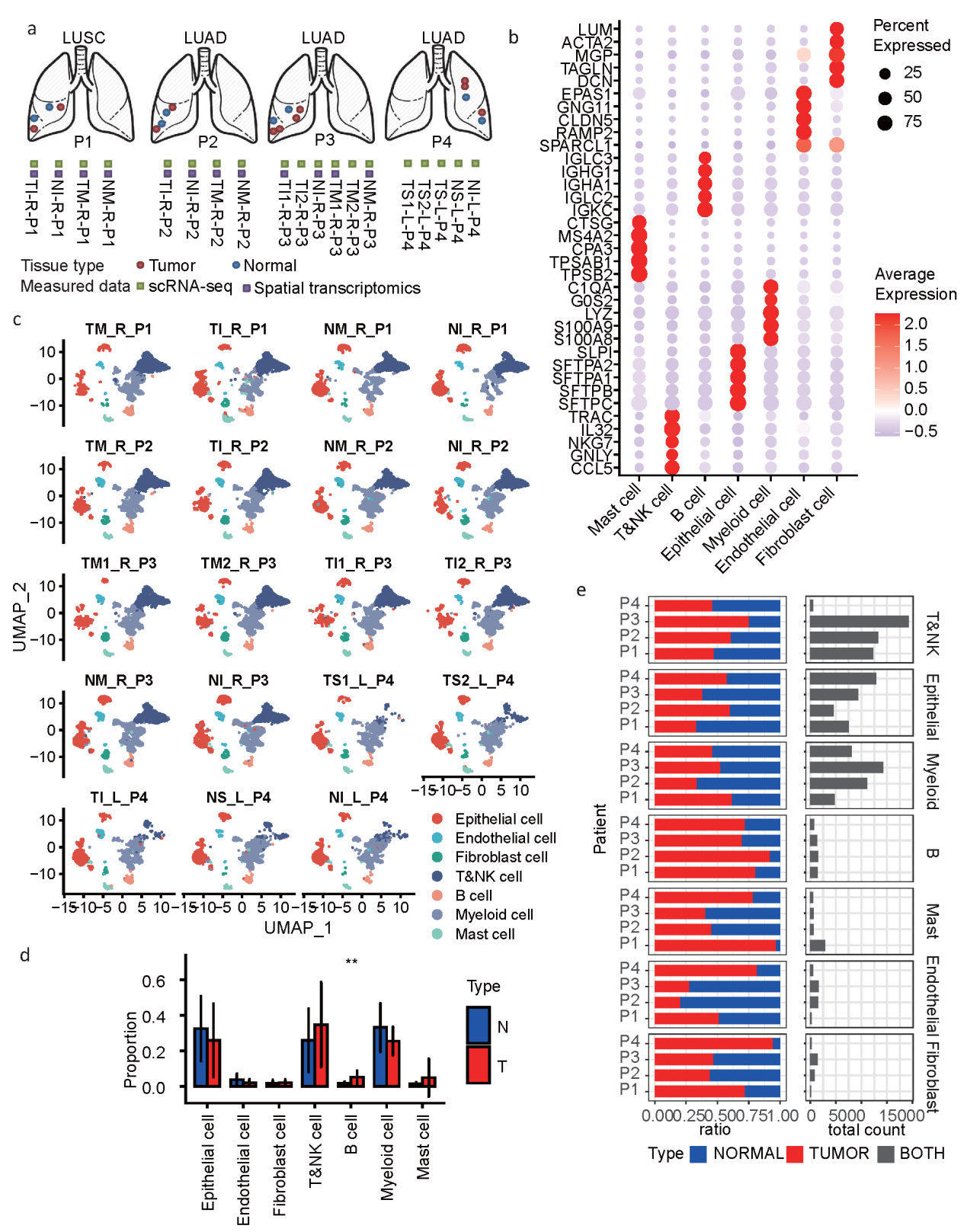
**

**Supplementary Fig 1. High resolution description of tumor heterogeneity for MPLC based on scRNA-seq.**
a. Overview about sample origin and data measurement. TI: tumor tissue from inferior lobe; TM: tumor tissue from middle lobe; TS: tumor tissue from superior lobe; NI: normal tissue adjacent to TI; NM: normal tissue adjacent to TM; NS: normal tissue adjacent to TS; R: right; L: left.

b. Dot plot of the marker genes for cell type annotation. Node size is proportional to the percent of cells expressed the gene. Node color represents the average expression of one gene.

c. Umap projection of single cells from each sample. Colors represent different cell types.

d. The cell type proportions in tumor lesions (red) or the adjacent normal tissues (blue) from the four MPLC patients. Data represent mean ± s.d. **: p < 0.01, Kruskal test.
e. The left part represents the proportion of cells from normal and tumor tissues. The right part represents the total count of cells in terms of each cell type for each patient.


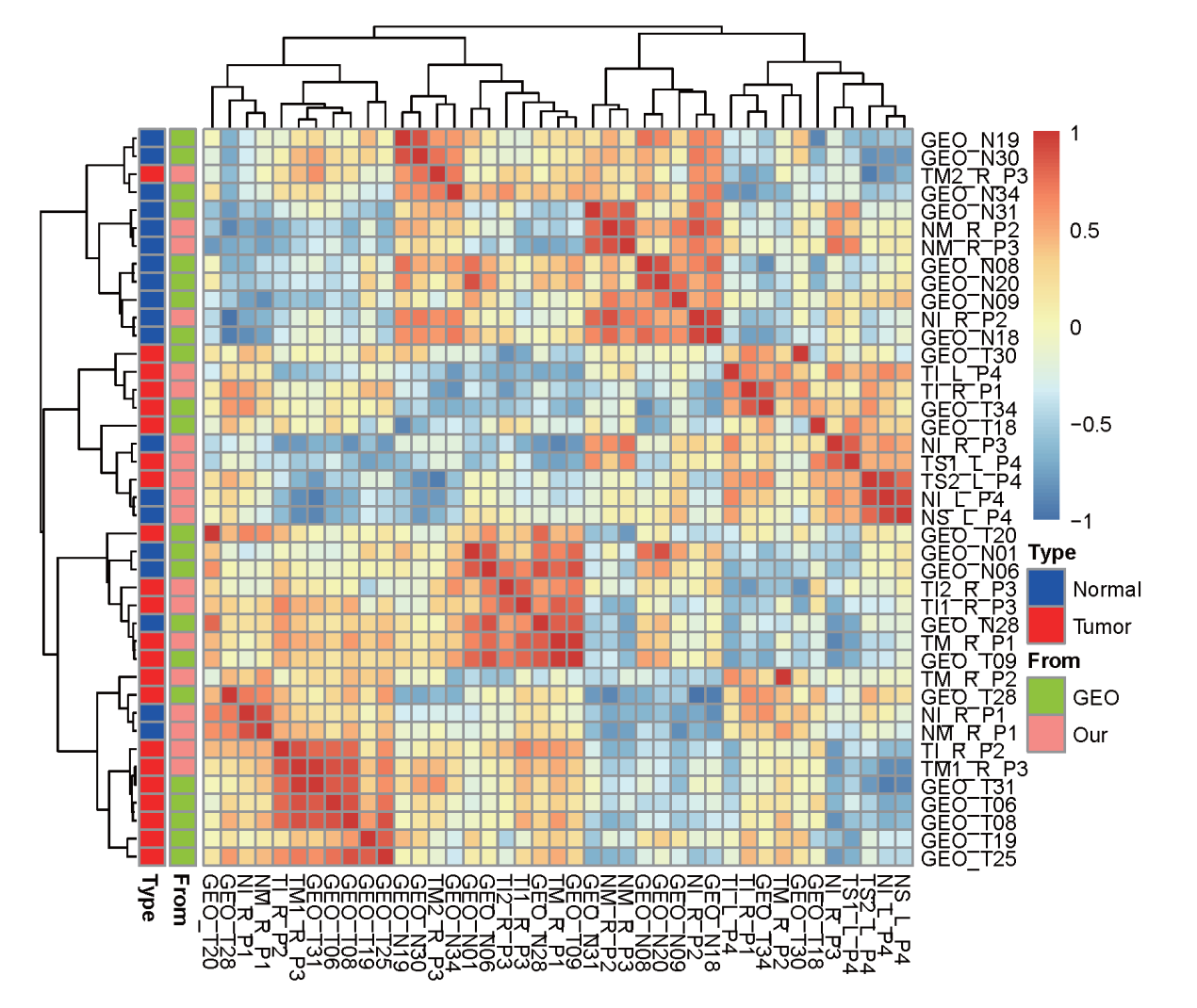
**Supplementary Fig. 2** Heatmap showing the clustering of both samples collected by this study and the other lung samples from an independent study (GEO dataset id:GSE131907). The colors in the heatmap represent the spearman correlation coefficient between the cell type compositions of two samples.


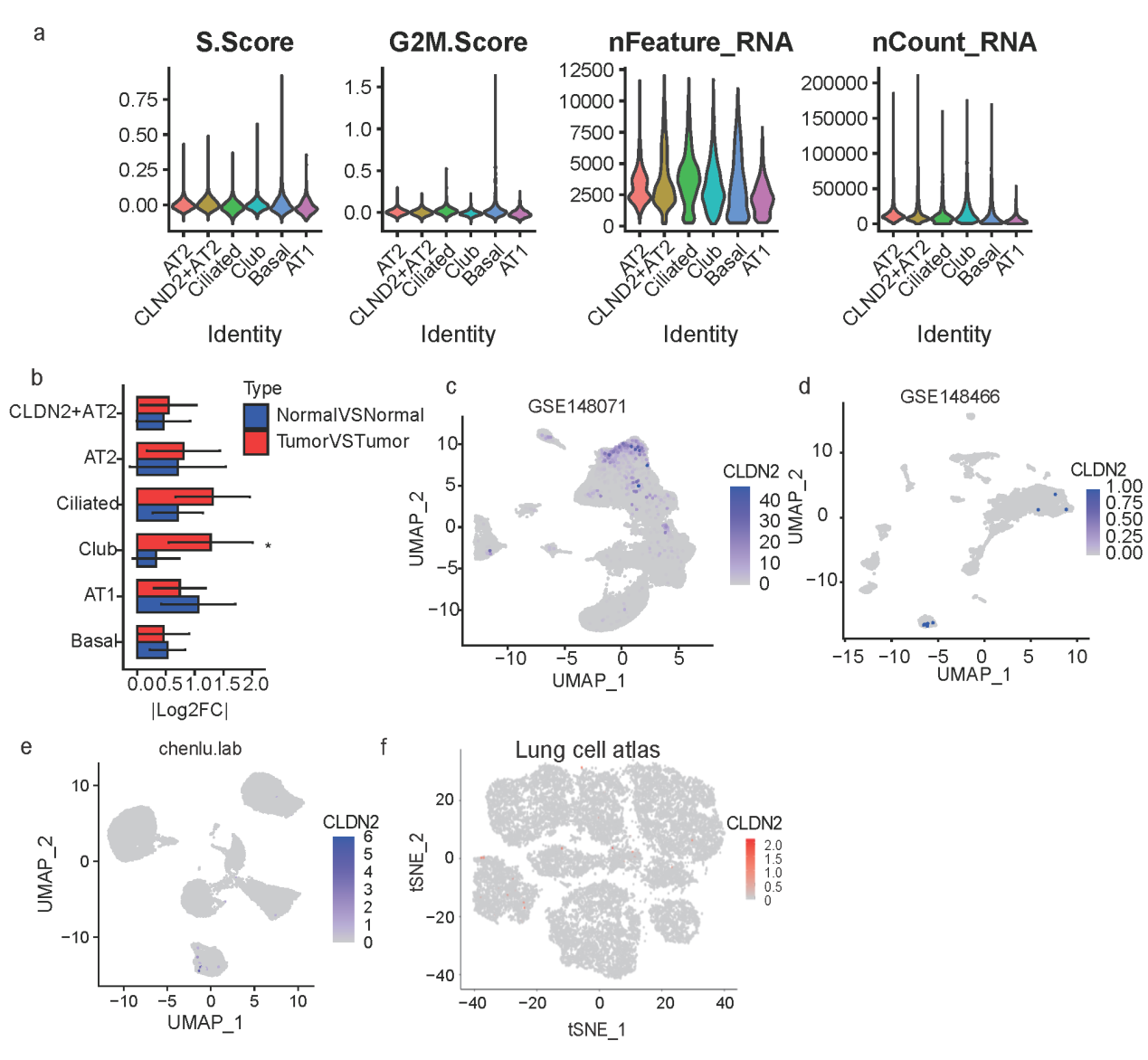


**Supplementary Fig. 3. CLDN2^+^ AT2 subtype is specific to MPLC**

a. Violin plot of the cell cycle scores (S.Score and G2M.Score), nFeature_RNA and nCount_RNA of different epithelial subtypes.

b. The log2-transformed fold change (Log2FC) of the epithelial cell sub-population proportions of samples from different tumor lesions (red) or tumor adjacent normal tissues (blue) in the MPLC patients. Data represents mean ± s.d.
c-f. Umap projection of the single cells in dataset GSE148071 (c), GSE148466 (d), chenlu.lab (e) and lung cell atlas (f). Point colors represent the expressions of CLDN2. GSE148071 and GSE148466 were from Gene Expression Omnibus. chenlu.lab data were from <http://lungcancer.chenlulab.com/#/cells> (selected data: AT2 cells in LUAD). Lung cell atlas was from <https://asthma.cellgeni.sanger.ac.uk/> (selected dataset:lung atlas epithelial).

**
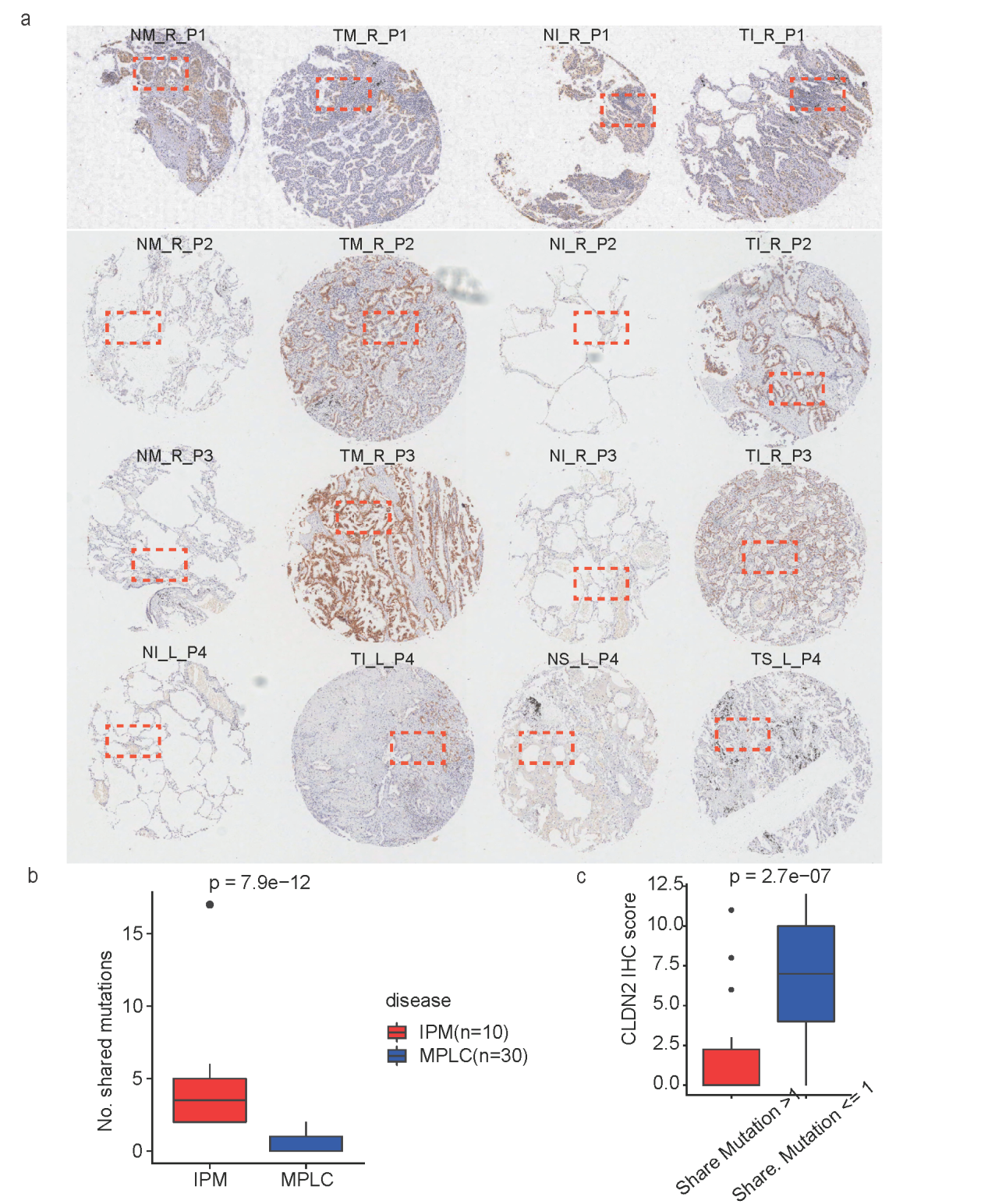
**

**Supplementary Fig. 4. CLDN2 protein expression can be a biomarker of MPLCs.**

a. Tissue microarray was conducted in samples from four MPLCs (P1-P4) to detect the expression and location of CLDN2 protein. Red border area was magnified 200× and presented in Supplementary Data 1.

b. Box plot of the number of shared mutations in IPM and MPLC patients. Each IPM or MPLC patient possessed two LUAD lesions at ipsilateral different lobes.
c. Box plot of the CLDN2 IHC scores. The colors represent whether the number of shared mutations of the two lesions in the patient (either MPLC or IPM) is larger than 1.


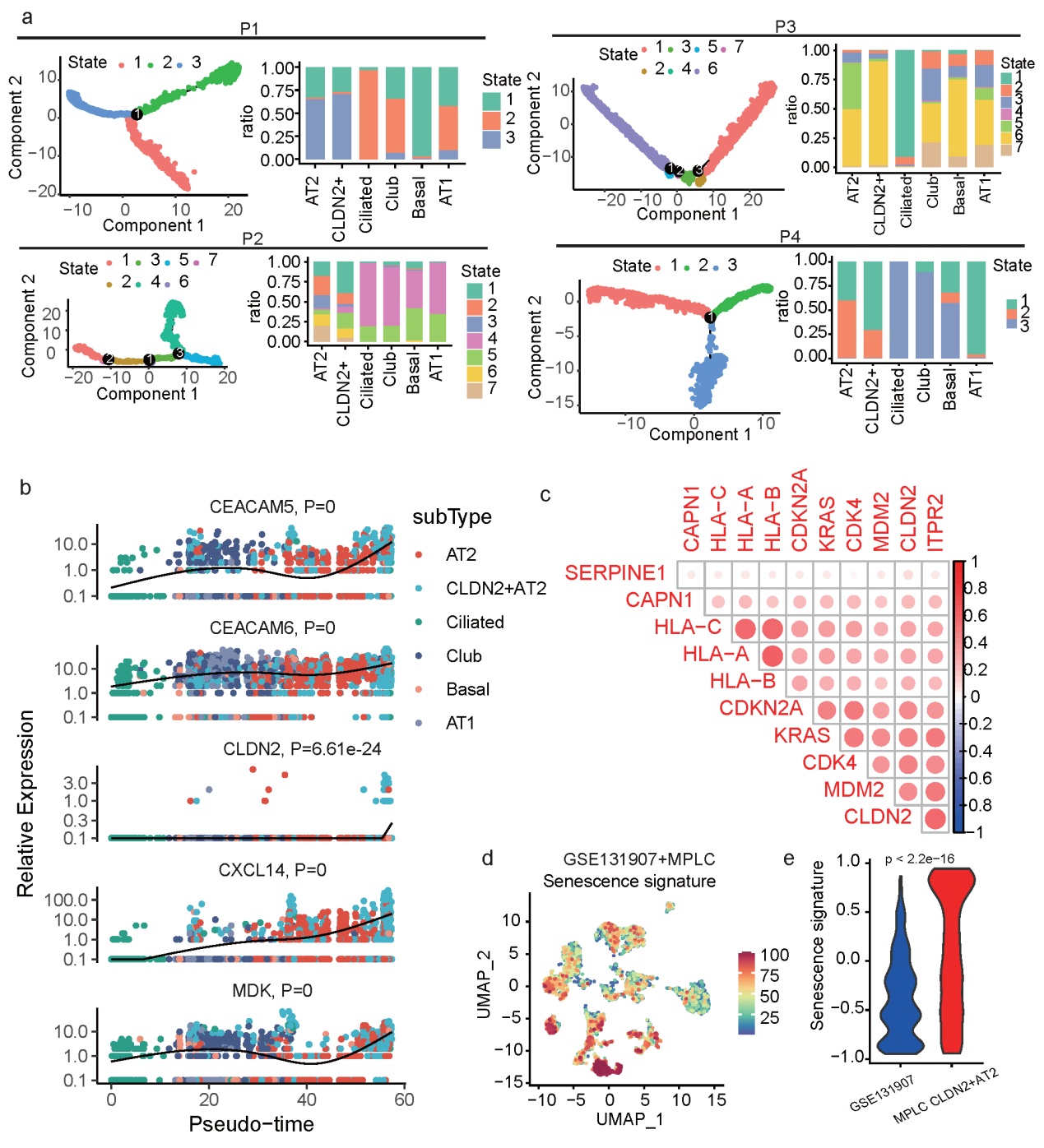
**Supplementary Fig. 5. Pseudotime analysis of the epithelial cells.**

a. Pseudotime trajectory of the epithelial cells in each patient. Colors represent the different transcriptional states identified based on the trajectories.

b. Plot expressions of the CLDN2^+^ AT2 marker genes as a function of pseudotime. P: R package monocle.

c. The Pearson correlation coefficients between CLDN2 and genes involved in cellular senescence. Only those with coefficient > 0.1 were displayed.

d. Umap projection of the integrated single cells in GSE131907 and the MPLC samples in this study. The colors represent the senescence signatures of the cells. The signature was computed based on the cell-wise gene set variation analysis, and the cellular senescence genes (*MDM2****,*** *CDK4*, *KRAS*, *CDKN2A*, *ITPR2*) were utilized as the signature geneset.

e. Violin plot of the senescence signature scores of cells in GSE131907 and the CLDN2^+^ AT2 cells in MPLC. P: Wilcox-test, un-paired.


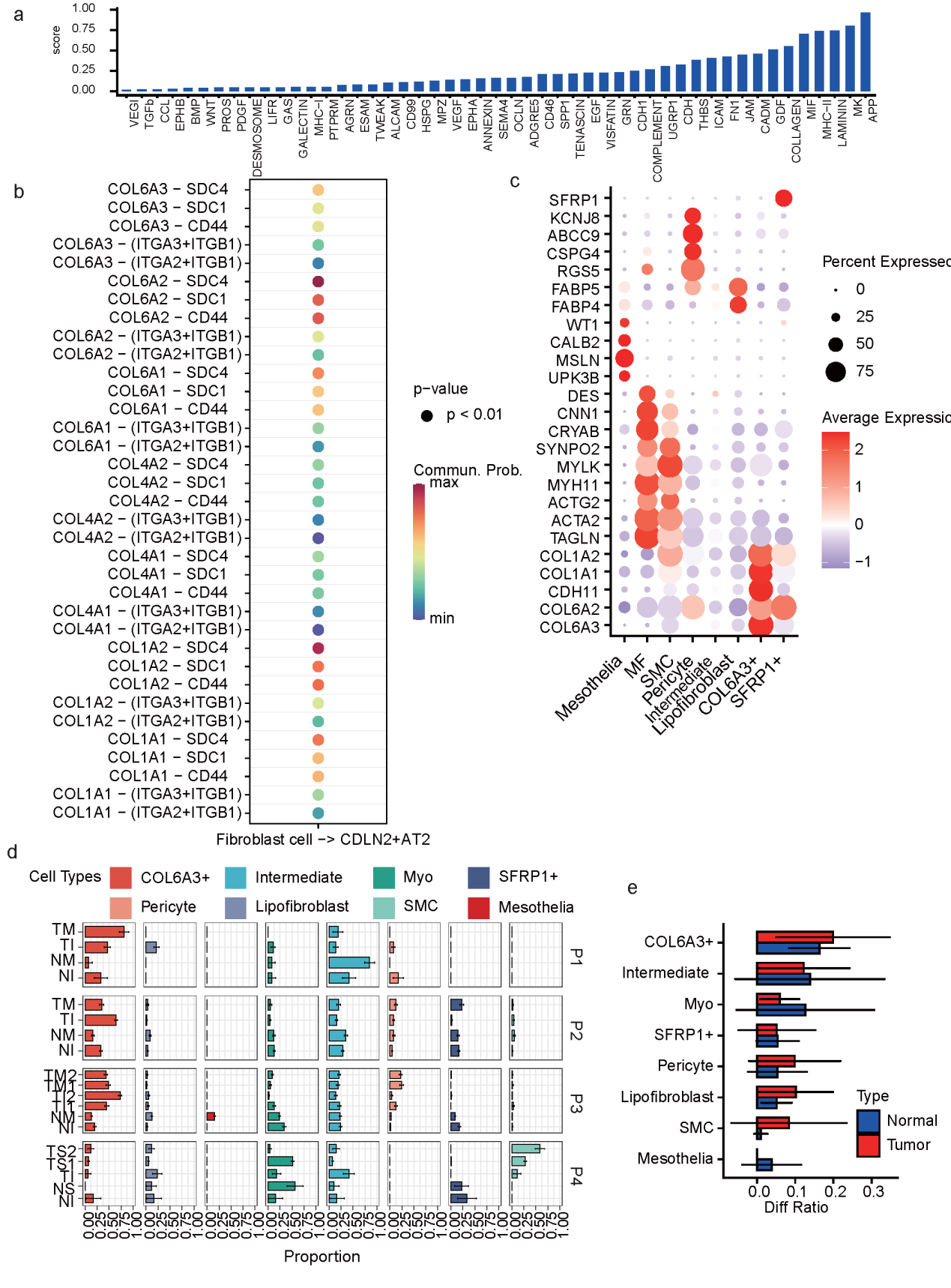


**Supplementary Fig. 6. Cell-cell interactions in MPLC.**

a. Pathway scores based on the interactions (ligand-receptor pairs) started from epithelial cells. Each bar refers to one pathway.

b. Bubble plot of the interactions originated from the fibroblast cells and mediated by collagens.

c. Dot plot of the expression of marker genes for the sub-populations of fibroblast cells.

**
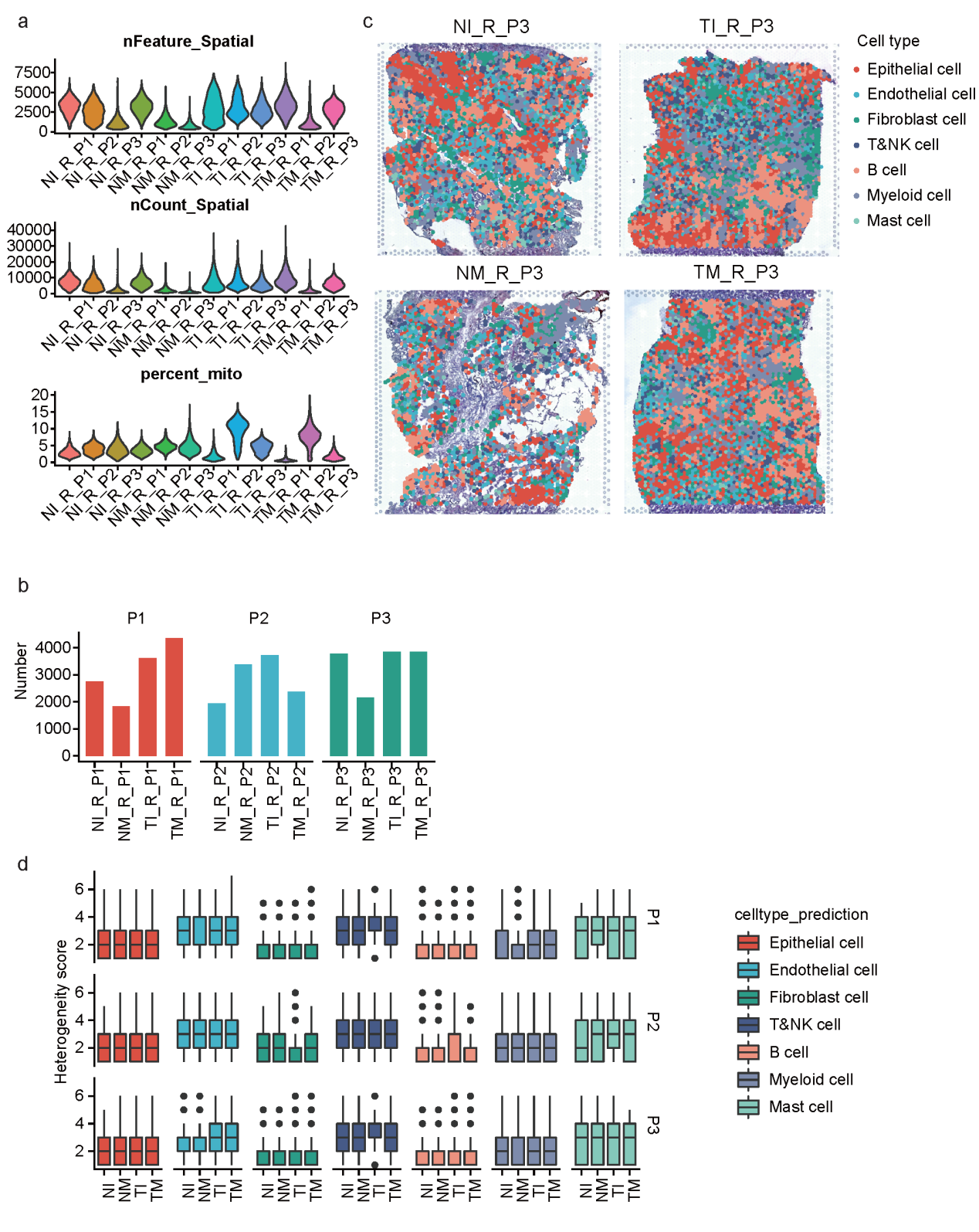
**

**Supplementary Fig. 7. Spatial features of different cell types in MPLC.**

a. Violin plot showing the spatially resolved number of genes (nFeature_Spatial), UMIs (nCount_Spatial) and the percentage of mitochondria (mito) in each measured sample.
b. The number of spatial spots after quality control.
c. Spatial RNA-seq barcoded spots of the samples from patients P3, labeled by the predicted cell types.
d. Box plot showing the distributions of heterogeneity scores of different cell types in each sample.

**
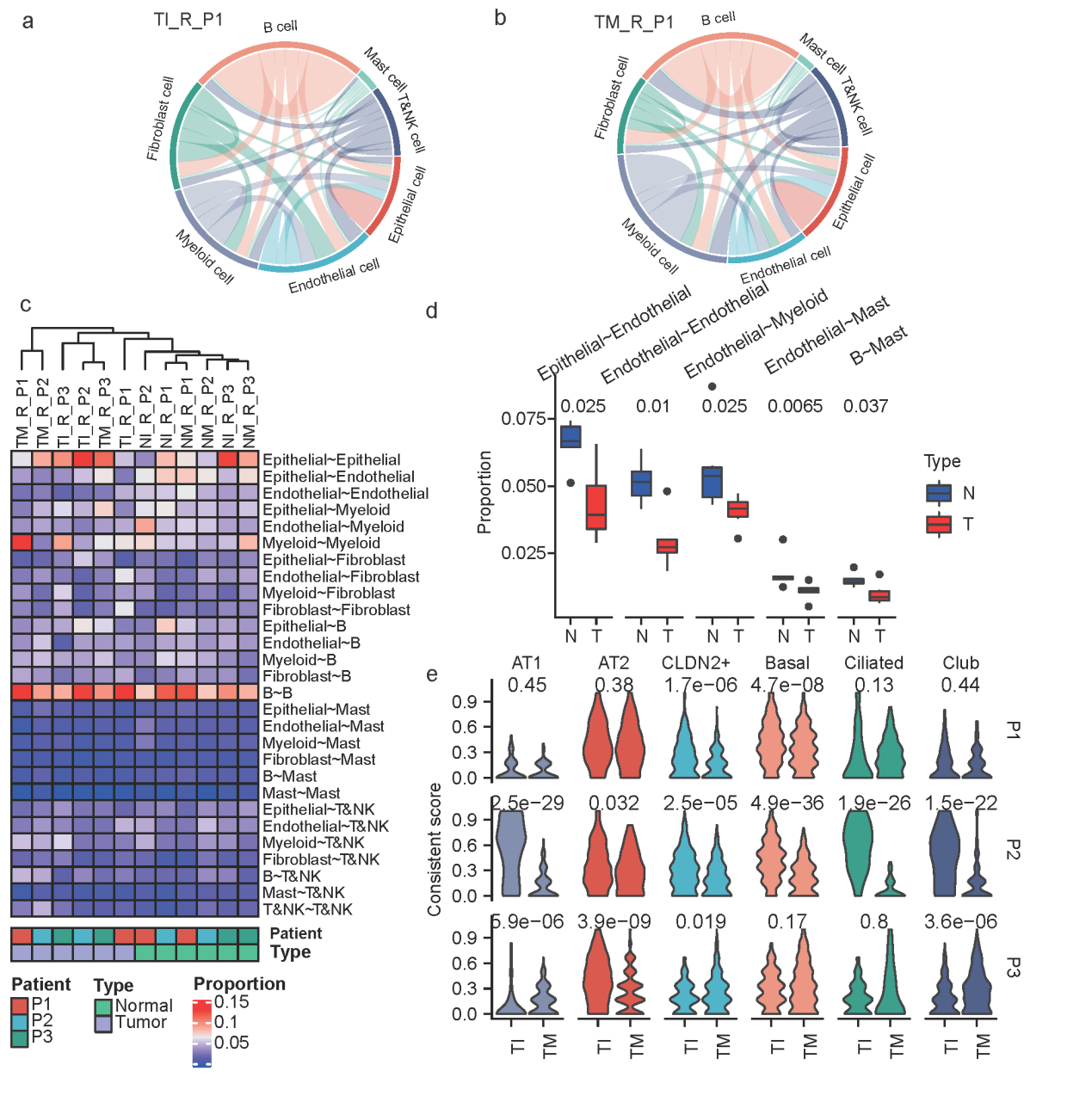
**

**Supplementary Fig. 8. Spatial features of different cell types in MPLC.**

a, b. Chord diagram showing the spatial neighborhood relations between different cell types in the sample TI_R_P1 (a) and TM_R_P1 (b).
c. Heatmap showing the clustering of samples based on the composition profiles of different types of spatial neighborhood relations.
d. Box plot showing the differences of tumor and normal tissues in terms of five types of spatial neighborhood relations.
e. Violin plot of the differences of consistent scores of spots from different tumor lesions of the same patient in terms of different dominant epithelial subtypes (p-value: Kruskal-test, unpaired)


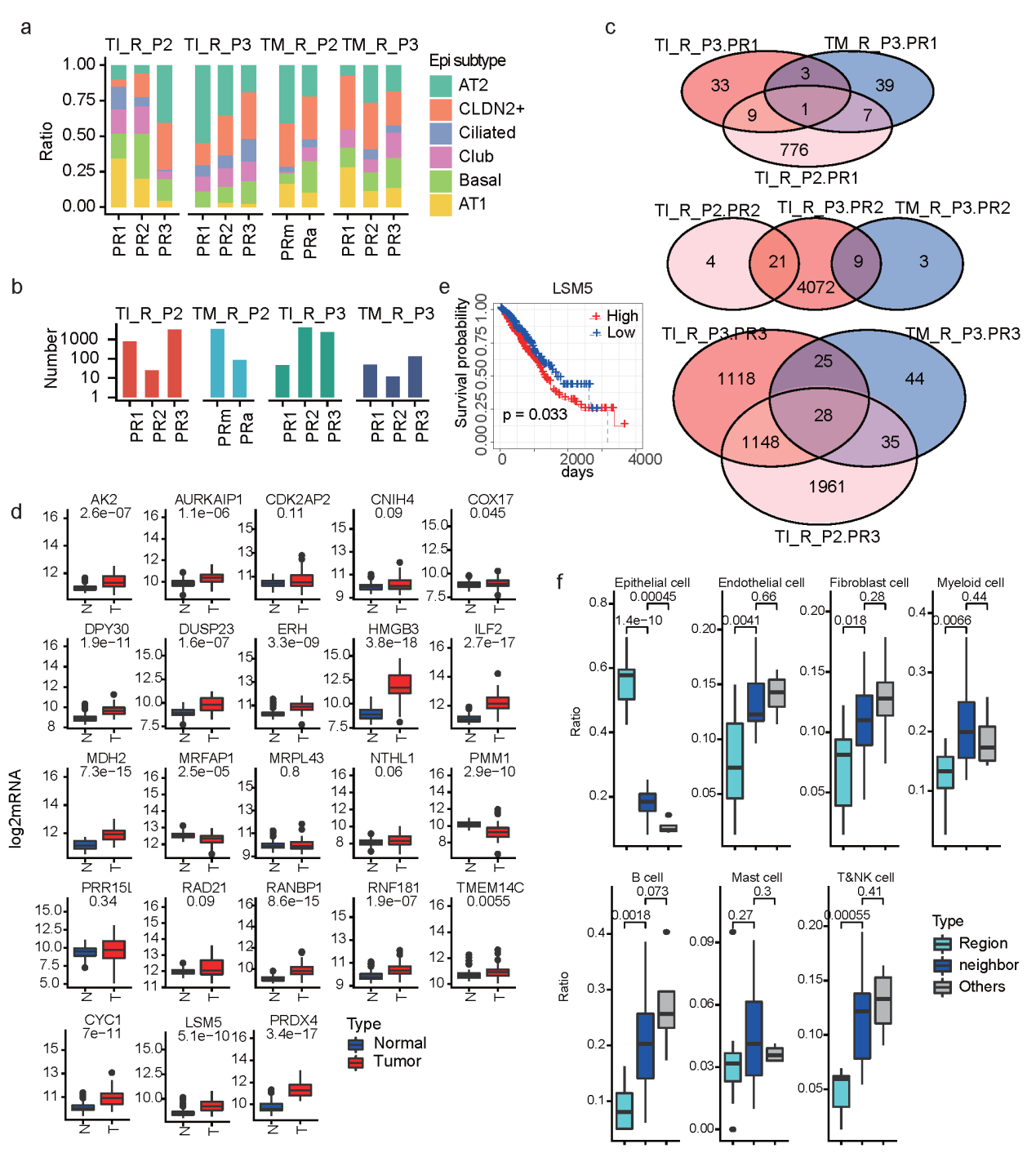


**Supplementary Fig. 9. The potential pathological marker genes for different histologic patterns in MPLC.**
a. The epithelial cell sub-population compositions of the pathological regions across the four investigated samples.

b. The number of differentially expressed genes of the pathological regions across the four investigated samples.

c. Venn plot of the overlap of the differentially expressed genes of histological regions in the three observed samples.
d. Boxplot of the shared PR3 gene expressions between tumor and normal tissues in the TCGA-LUAD dataset (p-value: Kruskal-test, unpaired).
e. KM-plot of the survival curves of TCGA-LUAD patients. The patients were separated into two groups according to whether LSM5 expression was higher than the median level.
f. Box plot of the difference of cell compositions between the malignant regions, corresponding spatial neighbors and the other remaining regions. T-test, unpaired.


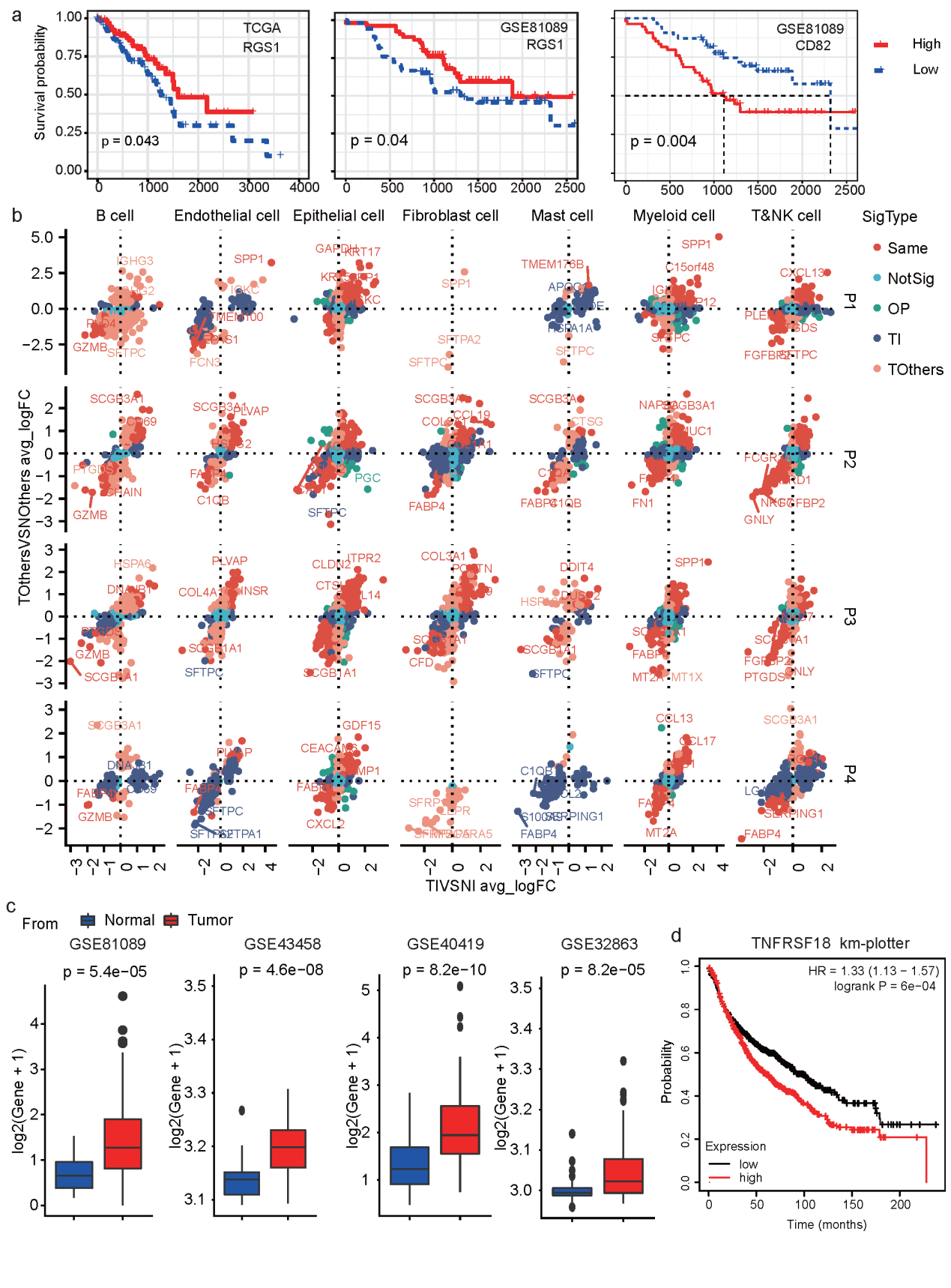


**Supplementary Fig. 10. Molecular and cellular commonness and differences between lesions within the same MPLC patient.**
a. KM-plot of the survival curves of RGS1 or CD82 in the TCGA-LUAD or GSE81089. The patients were separated into two groups according to whether gene expression was higher than the median level.

b. Scatter plot of the cell type-specific differential expressions of genes in the two different tumor lesions comparing to the corresponding adjacent normal tissues in each of the four MPLC patients. The x axis represents the average log2FC (avg_logFC) computed by comparing each type of cells in the tumor and normal tissues in the inferior lobe (TIVSNI) of one patient. The y axis represents the average log2FC (avg_logFC) computed by comparing each type of cells the tumor and normal tissues in the other diseased lobe (TOthersVSNOthers) of one patient. The point colors represent similar meanings as Figure 7a.
c. Box plot showing the differential expressions of TNFRSF18 between tumor and normal tissues in lung cancer based on four GEO datasets. Wilcox-test, un-paired.

d. KM-plot of the survival curve about TNFRSF18 in lung cancer based on km-plotter (<https://kmplot.com/analysis/index.php?p=service&cancer=lung>).


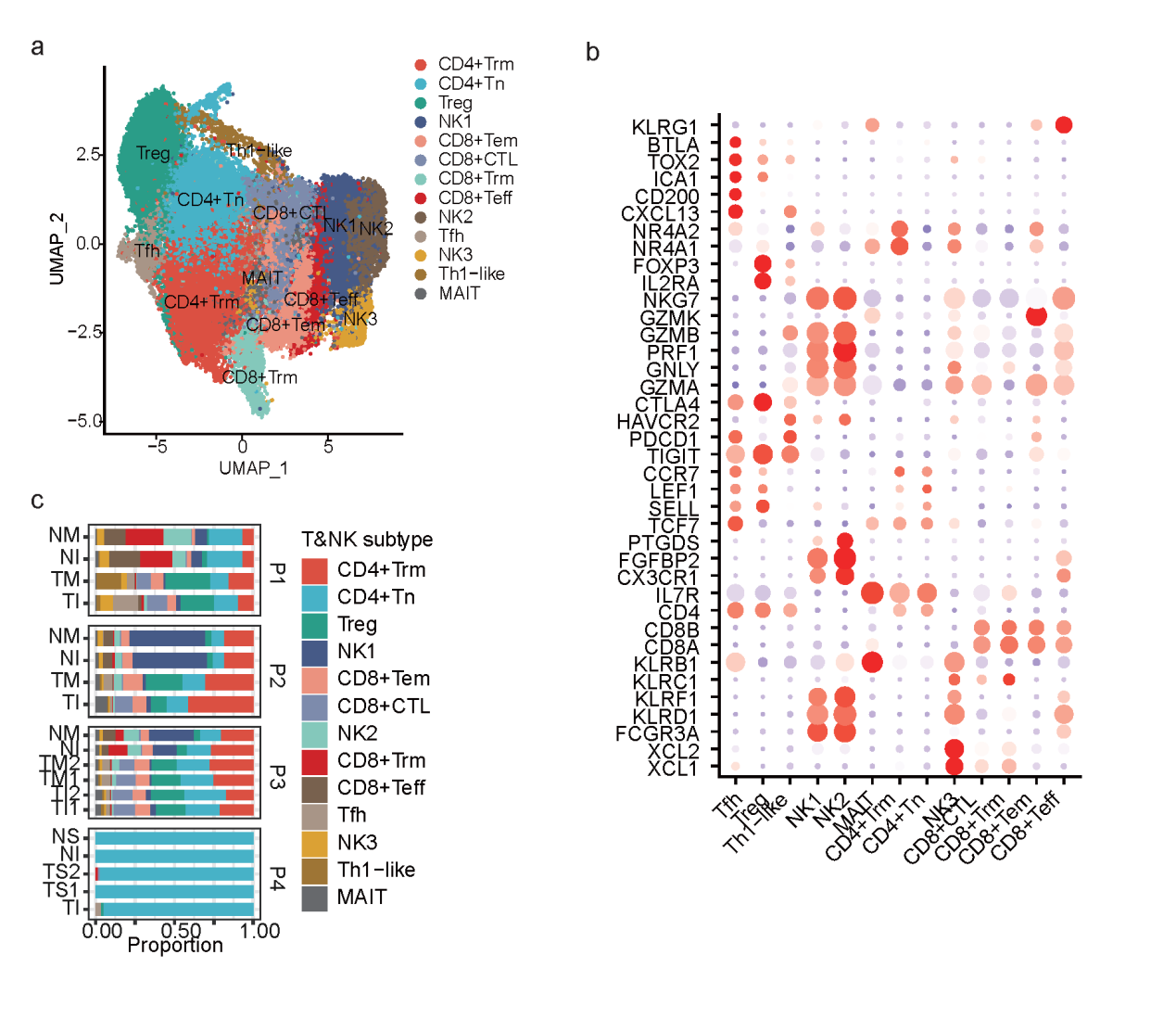


**Supplementary Fig. 11. Sub-category of the Organismal Systems**

a. UMAP plot of the subpopulations of T&NK. Trm: tissue-resident memory T; Tn: naïve T; Treg: regulatory T; Tem: effector memory T; CTL: cytotoxic T lymphocyte; Teff: effector T; MAIT: mucosal-associated invariant T; Tfh: T follicular helper; Th1: T helper type 1.

b. Dot plot of the expression of marker genes for the sub-populations of T&NK cells.

c. Bar plot of the proportions of sub-populations of T&NK (D), myeloid (E) and B (F) cells in each sample of the MPLC patients.

**Supplementary Tables**

**Supplementary Table 1. The clinicopathologic data of four MPLC patients.**

**Supplementary Table 2. The mutational files of the tumor lesions in 31 MPLC patients based on 425 paneled NGS.**

**Supplementary Table 3. Significant markers for different pathological regions from four spatially analyzed samples. The FindAllMarkers function in Seurat was utilized to get a list of differentially expressed genes for each pathological region. Only genes with |avg_logFC| > 1 and p_val_adj < 0.01 were listed.**

**Supplementary Data**

**Supplementary Data 1. The 200× magnified IHC images.**
