# Supplementary material for: Multidirectional characterization of cellular composition and spatial architecture in human multiple primary lung cancers
Source: Cell Death Dis. 2023 Jul 25;14(7):462. doi: 10.1038/s41419-023-05992-w (PMC10366158; doi:10.1038/s41419-023-05992-w)
Supplement: Supplementary file 2 — Table S1-MPLC [file 41419_2023_5992_MOESM2_ESM.doc]

**Table S1: The clinicopathologic datas of 4 MPLC patients.**

| **Characteristics** | **P1** | **P2** | **P3** | **P4** |
| --- | --- | --- | --- | --- |
| **SCC or ADC** | SCC | ADC | ADC | ADC |
| **Age** | 69 | 62 | 71 | 48 |
| **Gender** | Male | Male | Male | Female |
| **Smoking** | Yes | Yes | Yes | No |
| **Lesion number** | 2 | 2 | 2 | 2 |
| **Lesion location** | 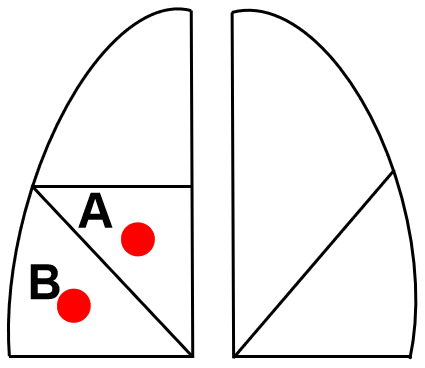 | 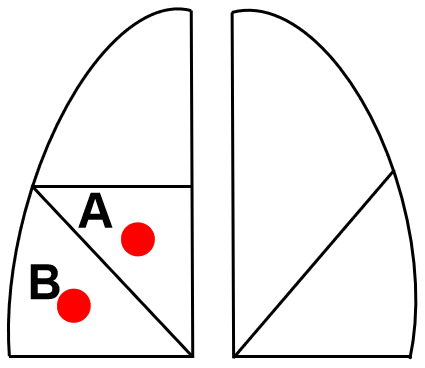 | 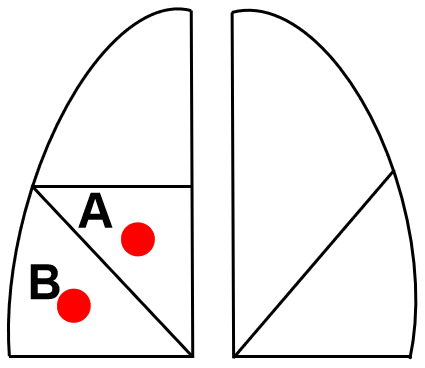 | 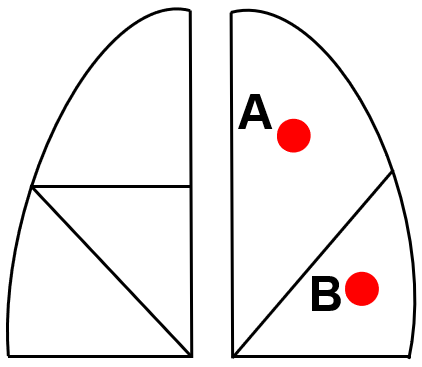 |
| **CT gross size (cm)** | A: 3.0*2.5*2.0  B: 1.8*1.5*1.0 | A: 1.1*0.8*0.6  B: 1.5*1.3*0.3 | A: 1.2*1.2*1.0  B: 3.0*2.5*1.8 | A: 1.2*1.0*0.8  B: 1.2*0.6*0.6 |
| **CT solid size (cm)** | A: 3.0*2.5*2.0  B: 1.5*1.3*0.8 | A: 0*0*0  B: 0.7*0.5*0.2 | A: 0.8*0.6*0.4  B: 1.8*1.8*1.6 | A: 0*0*0  A: 0*0*0 |
| **CT type** | A: pure solid  nodule  B: solid-predominant nodule | A: GGN  B: GGO-predominant part-solid nodule | A: solid-predominant  nodule  B: solid-predominant  nodule | A: GGN  B: GGN |
| **CT morphology**  **Spiculation** | A: yes  B: no | A: no  B: no | A: no  B: no | A: no  B: no |
| **CT morphology**  **Air-bronchogram** | A: no  B: yes | A: no  B: yes | A: no  B: no | A: no  B: no |
| **lymphatic metastasis** | No | No | No | No |
| **Pathological gross size (cm)** | A: 2.8*2.3*2.0  B: 1.6*1.3*0.9 | A: 0.7*0.5*0.3  B: 1.3*1.1*0.2 | A: 1.2*1.2*0.9  B: 2.8*2.5*1.8 | A: 1.1*1.0*0.9  B: 1.1*0.6*0.6 |
| **Pathological maximal invasive degree (cm)** | A: 2.8  B: 1.6 | A: 0.3  B: 0.6 | A: 0.9  B: 2.5 | A: 0  B: 0 |
| **Pathology (HE)** | A: SCC (moderate + poor), invading visceral pleura  B: SCC (moderate) | A: MIA  B: IA  (lepidic: 30%; papillary: 30%; micropapillary: 40%) | A: IA  (lepidic: 10%; papillary: 50%; micropapillary: 40%)  B:IA  (lepidic: 15%; papillary: 75%; micropapillary: 10%), invading visceral pleura | A: AIS  B:AIS |
| **Pathology (IHC)** | A: CK7（-），CK5/6（+），TTF1（-），P63（+），P40（+），Ki-67 70%(+)  B: CK7（-），CK5/6（+），TTF1（-），P63（+），P40（+/-），Ki-67 20%(+) | A: CK（+），CK7（+），TTF1（+），NapsinA（+），CK5/6（-），P63（+），P40（-），ALK(D5F3)（-），Ki-67 5%（+）  B: CK（+），CK7（+），TTF1（+），NapsinA（+），CK5/6（-），P63（-），P40（-），ALK(D5F3)（-），Ki-67 10%（+） | A: CK7（+），TTF1（+），NapsinA（+/-），CK5/6（-），P63（-），P40（-），ALK(D5F3)（-），Ki-67 10%（+）  B: CK7（+），TTF1（+），NapsinA（+），CK5/6（-），P63（-），P40（-），ALK(D5F3)（-），Ki-67 10%（+） | A: CK7（+），TTF1（+），NapsinA（+），CK5/6（-），P63（-），P40（-），Ki-67 5%（+）  B: CK7（+），TTF1（+），NapsinA（+），CK5/6（-），P63（+/-），P40（-），ALK(D5F3)（-），Ki-67 5%（+） |
| **Tumor mutation burden** | A: 7.4/MB  B: 12.7/MB | A: 6.1/MB  B: 1.1/MB | A: 5.1/MB  B: 3.1/MB | A: 2.1/MB  B: 0/MB |
