# Supplementary material for: Multidirectional characterization of cellular composition and spatial architecture in human multiple primary lung cancers
Source: Cell Death Dis. 2023 Jul 25;14(7):462. doi: 10.1038/s41419-023-05992-w (PMC10366158; doi:10.1038/s41419-023-05992-w)
Supplement: Supplementary file 4 — Table S3-selected Region markers. [file 41419_2023_5992_MOESM4_ESM.docx]

"p_val" "avg_logFC" "pct.1" "pct.2" "p_val_adj" "cluster" "gene" "From"

"1" 3.10657664203862e-136 2.70728529717152 0.996 0.879 1.03787619033868e-131 "PR2" "C16orf89" "TI_R_P3"

"2" 1.54757545517545e-119 18.5089922529259 0.979 0.816 5.17029483819566e-115 "PR2" "GDF15" "TI_R_P3"

"3" 2.70959533190046e-119 2.12404863720948 0.979 0.825 9.05248704434625e-115 "PR2" "EPCAM" "TI_R_P3"

"4" 4.66925088582579e-117 5.66603078434041 0.956 0.732 1.55995002844554e-112 "PR2" "MALL" "TI_R_P3"

"5" 3.54124470468622e-111 6.36382188123318 0.954 0.698 1.18309444338862e-106 "PR2" "ATP11A" "TI_R_P3"

"6" 5.99322148427192e-109 3.08536325478527 0.988 0.848 2.00227536568041e-104 "PR2" "CLPTM1L" "TI_R_P3"

"7" 4.61201823022349e-106 6.79900892177789 0.954 0.712 1.54082917053536e-101 "PR2" "TACSTD2" "TI_R_P3"

"8" 4.87779106983334e-105 1.88863761243393 0.939 0.758 1.62962121852062e-100 "PR2" "APOD" "TI_R_P3"

"9" 6.47200214389315e-103 10.0747892686179 0.907 0.604 2.16223119625326e-98 "PR2" "SFTPD" "TI_R_P3"

"10" 1.11598070074814e-99 9.18786607190088 0.928 0.711 3.72837992312946e-95 "PR2" "KRT15" "TI_R_P3"

"11" 1.05629359152864e-98 7.05691982106242 0.984 0.845 3.52897125993804e-94 "PR2" "TM7SF3" "TI_R_P3"

"12" 2.67000716693995e-97 1.98620147555953 0.947 0.744 8.92022694402969e-93 "PR2" "CLDN4" "TI_R_P3"

"13" 2.60536699846374e-95 4.80508539656865 0.898 0.639 8.70427060516753e-91 "PR2" "STEAP4" "TI_R_P3"

"14" 3.09548228185638e-95 22.751484732852 0.898 0.633 1.0341696755454e-90 "PR2" "SCGB3A1" "TI_R_P3"

"15" 7.68040777681017e-92 9.3753424894402 0.879 0.593 2.56594743415451e-87 "PR2" "TFF3" "TI_R_P3"

"16" 1.44659924320765e-91 5.0073127434215 0.914 0.631 4.83294341163245e-87 "PR2" "PLCXD1" "TI_R_P3"

"17" 7.40232896490399e-91 2.52787190493502 0.9 0.608 2.47304408388477e-86 "PR2" "SFTA3" "TI_R_P3"

"18" 2.33087219734616e-88 1.16061222772023 0.954 0.728 7.78721092411378e-84 "PR2" "SMIM22" "TI_R_P3"

"19" 3.44228932296257e-87 4.93752340916856 0.919 0.653 1.15003443990856e-82 "PR2" "CXCL17" "TI_R_P3"

"20" 9.39107300937042e-86 1.99185245854859 0.979 0.864 3.13746358170056e-81 "PR2" "TXNDC17" "TI_R_P3"

"21" 6.1118088361536e-85 4.58079689987545 0.912 0.673 2.04189421407056e-80 "PR2" "TRIM2" "TI_R_P3"

"22" 9.07343911749156e-85 7.21559312612878 0.902 0.651 3.03134527476275e-80 "PR2" "ELF3" "TI_R_P3"

"23" 3.91639192477178e-84 8.85922426562366 0.982 0.864 1.308427378147e-79 "PR2" "PRDX5" "TI_R_P3"

"24" 4.06006325462815e-84 15.7510059161713 0.888 0.632 1.35642653273872e-79 "PR2" "AZGP1" "TI_R_P3"

"25" 5.41036047835438e-84 4.32822108662688 0.686 0.334 1.80754733221342e-79 "PR2" "SNX25" "TI_R_P3"

"26" 1.26667118027232e-83 14.4490877134977 0.919 0.684 4.23182174617179e-79 "PR2" "CLDN2" "TI_R_P3"

"27" 4.92739787721845e-83 2.37354485704999 0.967 0.814 1.64619435679991e-78 "PR2" "CAPN2" "TI_R_P3"

"28" 1.67076362530311e-82 3.00881473071188 0.981 0.86 5.58185419577515e-78 "PR2" "VAMP8" "TI_R_P3"

"29" 6.761644997543e-82 1.28624750016577 0.907 0.65 2.25899797722914e-77 "PR2" "MLPH" "TI_R_P3"

"30" 5.93819847291559e-80 2.26421413339153 0.972 0.855 1.98389272781637e-75 "PR2" "COX7B" "TI_R_P3"

"31" 7.00958363551802e-80 5.27891850958019 0.933 0.71 2.34183179679021e-75 "PR2" "PON2" "TI_R_P3"

"32" 1.15135397120243e-79 1.64021974811243 0.965 0.826 3.84655848239019e-75 "PR2" "MAL2" "TI_R_P3"

"33" 1.13669101548914e-78 6.71561086925682 0.904 0.678 3.79757101364767e-74 "PR2" "FOLR1" "TI_R_P3"

"34" 3.39202686625373e-78 2.38630712712728 0.96 0.808 1.13324225574671e-73 "PR2" "GTF2I" "TI_R_P3"

"35" 6.39552889584057e-78 4.47594765885537 0.97 0.809 2.13668224881138e-73 "PR2" "CDK4" "TI_R_P3"

"36" 1.82963598655229e-77 12.4350105043863 0.768 0.487 6.11263086747255e-73 "PR2" "CEACAM5" "TI_R_P3"

"37" 2.2092561896608e-77 2.04792846819157 0.875 0.602 7.38090400403776e-73 "PR2" "LAPTM4B" "TI_R_P3"

"38" 5.61645022874357e-77 2.14408122432808 0.772 0.453 1.87639985692094e-72 "PR2" "IRX2" "TI_R_P3"

"39" 4.0897159169053e-76 3.67959980899802 0.937 0.75 1.36633319067889e-71 "PR2" "NDUFB9" "TI_R_P3"

"40" 6.55222012278637e-76 1.62199868343354 0.858 0.581 2.1890312208217e-71 "PR2" "ALCAM" "TI_R_P3"

"41" 7.13511925258192e-76 2.640517726261 0.868 0.596 2.38377199109509e-71 "PR2" "ACSL5" "TI_R_P3"

"42" 7.25148193489437e-76 4.60061483400673 0.926 0.734 2.42264759962886e-71 "PR2" "CAND1" "TI_R_P3"

"43" 1.66210426544446e-75 3.85078410338346 0.791 0.459 5.55292414042341e-71 "PR2" "ITPR3" "TI_R_P3"

"44" 8.85590365473492e-73 2.5283812803077 0.912 0.72 2.95866885201039e-68 "PR2" "ADI1" "TI_R_P3"

"45" 3.79891875757441e-72 2.37823662233525 0.947 0.769 1.26918076771803e-67 "PR2" "HINT1" "TI_R_P3"

"46" 1.32112921708686e-71 5.7426962247686 0.932 0.746 4.41376060136549e-67 "PR2" "PCSK1N" "TI_R_P3"

"47" 1.68719389889672e-71 1.58019326843975 0.907 0.685 5.63674609682405e-67 "PR2" "CADM1" "TI_R_P3"

"48" 2.1012935189704e-71 4.24147503312111 0.926 0.748 7.02021151752822e-67 "PR2" "OCIAD2" "TI_R_P3"

"49" 2.50372133814411e-71 5.7132436339844 0.658 0.334 8.36468261860567e-67 "PR2" "CLDN18" "TI_R_P3"

"50" 5.61993728098434e-71 5.91376228950688 0.816 0.534 1.87756484620406e-66 "PR2" "TMEM163" "TI_R_P3"

"51" 1.55666758716208e-70 1.31569097895957 0.805 0.507 5.20067074194978e-66 "PR2" "TSPAN13" "TI_R_P3"

"52" 1.57352487025473e-70 6.4935870936328 0.926 0.737 5.25698923903403e-66 "PR2" "WSB1" "TI_R_P3"

"53" 1.95625336573765e-69 1.95520670722521 0.933 0.737 6.53564686959292e-65 "PR2" "RAB21" "TI_R_P3"

"54" 2.77939220920422e-69 5.83501267662245 0.951 0.842 9.28567143173039e-65 "PR2" "ZFP36L1" "TI_R_P3"

"55" 1.9196796408221e-68 2.53588105979628 0.944 0.786 6.41345771202254e-64 "PR2" "COX7A2" "TI_R_P3"

"56" 3.66547772245634e-68 5.84382012754894 0.853 0.601 1.22459945229544e-63 "PR2" "DUSP6" "TI_R_P3"

"57" 7.3082458109437e-68 3.41866705443719 0.84 0.552 2.44161184297818e-63 "PR2" "ADGRF5" "TI_R_P3"

"58" 3.22385497998572e-67 3.61749704665961 0.97 0.81 1.07705771026343e-62 "PR2" "LGMN" "TI_R_P3"

"59" 3.32641511671938e-67 2.13406647534807 0.791 0.5 1.11132202634478e-62 "PR2" "ETS2" "TI_R_P3"

"60" 9.01501016444834e-67 2.10837889099195 0.867 0.589 3.01182474584055e-62 "PR2" "MRPL14" "TI_R_P3"

"61" 1.38744900635477e-66 1.82026406532288 0.923 0.767 4.63532838533064e-62 "PR2" "MAGED2" "TI_R_P3"

"62" 2.3445613934333e-66 3.69184736051023 0.923 0.741 7.83294515932132e-62 "PR2" "FAM107B" "TI_R_P3"

"63" 4.8990496149265e-66 3.22922725451771 0.93 0.767 1.63672348585079e-61 "PR2" "DPYSL2" "TI_R_P3"

"64" 6.91053965818049e-66 5.62228168569565 0.939 0.742 2.30874219440152e-61 "PR2" "SFTA1P" "TI_R_P3"

"65" 7.12130324718687e-66 5.477371593633 0.902 0.695 2.37915620185266e-61 "PR2" "NDUFC1" "TI_R_P3"

"66" 7.56466013812076e-66 2.93792851222384 0.684 0.367 2.52727730554477e-61 "PR2" "FGGY" "TI_R_P3"

"67" 8.71576950826027e-66 1.66880270297824 0.975 0.838 2.91185143501467e-61 "PR2" "COX5B" "TI_R_P3"

"68" 1.08480661851745e-65 2.17428076071692 0.882 0.68 3.62423043180493e-61 "PR2" "DHCR24" "TI_R_P3"

"69" 1.11252158979399e-65 1.54317035036331 0.889 0.68 3.71682337934275e-61 "PR2" "EPHX1" "TI_R_P3"

"70" 1.1340569153094e-65 2.54954652981602 0.896 0.668 3.78877074835716e-61 "PR2" "ST14" "TI_R_P3"

"71" 1.42893023443087e-65 7.28111106336296 0.928 0.743 4.77391302021011e-61 "PR2" "BEX3" "TI_R_P3"

"72" 2.0124605419276e-65 6.54448763095775 0.742 0.441 6.72342942452592e-61 "PR2" "BEX2" "TI_R_P3"

"73" 2.74510804926311e-65 2.27455770098368 0.851 0.577 9.17113148178313e-61 "PR2" "EFNA1" "TI_R_P3"

"74" 4.61786349150529e-65 1.74331417122792 0.826 0.537 1.542782013877e-60 "PR2" "ABCA3" "TI_R_P3"

"75" 4.71327699124593e-65 1.70237976801907 0.707 0.389 1.57465871000535e-60 "PR2" "TMEM243" "TI_R_P3"

"76" 5.45861420919948e-65 2.18503806440854 0.923 0.732 1.82366842115145e-60 "PR2" "ECM1" "TI_R_P3"

"77" 1.35742984473628e-64 7.45776847239133 0.963 0.842 4.53503736827943e-60 "PR2" "ROMO1" "TI_R_P3"

"78" 1.40440468056259e-64 5.92922924607275 0.977 0.832 4.69197559729156e-60 "PR2" "KRT7" "TI_R_P3"

"79" 4.23110084929033e-64 3.00331192785498 0.649 0.334 1.4135684827394e-59 "PR2" "SLC12A2" "TI_R_P3"

"80" 4.55442454231189e-64 3.22499159487729 0.809 0.552 1.52158769534098e-59 "PR2" "PDCD6" "TI_R_P3"

"81" 9.98395161604548e-64 2.40124372084898 0.921 0.78 3.33553839540463e-59 "PR2" "TRMT112" "TI_R_P3"

"82" 1.41481319138787e-63 1.19200463575685 0.916 0.721 4.72674939110774e-59 "PR2" "HSPE1" "TI_R_P3"

"83" 2.4713863595086e-63 2.14701592672921 0.965 0.86 8.25665468848229e-59 "PR2" "BCAP31" "TI_R_P3"

"84" 3.61585849824399e-63 5.37723767816061 0.925 0.758 1.20802216567834e-58 "PR2" "NDUFB11" "TI_R_P3"

"85" 4.86737249116506e-63 2.53140304243645 0.572 0.252 1.62614047557333e-58 "PR2" "AL365226.2" "TI_R_P3"

"86" 7.76458703691605e-63 4.05046816120959 0.914 0.708 2.59407088316328e-58 "PR2" "ATP5IF1" "TI_R_P3"

"87" 8.08564497004966e-63 1.68904762392427 0.914 0.728 2.70133312804389e-58 "PR2" "TRIM28" "TI_R_P3"

"88" 2.36045431430692e-62 2.63825210074077 0.818 0.536 7.88604181866799e-58 "PR2" "CLDN1" "TI_R_P3"

"89" 2.99744113336265e-62 5.31968780905499 0.972 0.871 1.00141510824513e-57 "PR2" "SRP14" "TI_R_P3"

"90" 4.18014370998282e-62 3.25446177177584 0.904 0.71 1.39654421206816e-57 "PR2" "SLC25A5" "TI_R_P3"

"91" 4.47349345703951e-62 4.23247488207659 0.909 0.74 1.49454942906233e-57 "PR2" "DBI" "TI_R_P3"

"92" 7.27433350378389e-62 2.58270448410699 0.802 0.54 2.43028208027916e-57 "PR2" "S100A14" "TI_R_P3"

"93" 4.7280123540226e-61 1.56083505204429 0.914 0.747 1.57958164735541e-56 "PR2" "GDE1" "TI_R_P3"

"94" 5.3354746058998e-61 1.79313126428399 0.744 0.442 1.78252871108507e-56 "PR2" "CRNDE" "TI_R_P3"

"95" 5.63435838837321e-61 3.87682270434915 0.874 0.674 1.88238279397161e-56 "PR2" "TKT" "TI_R_P3"

"96" 6.85763807361553e-61 2.62212569638938 0.714 0.398 2.29106830401421e-56 "PR2" "TMEM125" "TI_R_P3"

"97" 7.36156992972893e-61 1.57568149989172 0.763 0.485 2.45942689782314e-56 "PR2" "SDHA" "TI_R_P3"

"98" 1.72441166882189e-60 2.01276846030101 0.691 0.382 5.76108694436707e-56 "PR2" "MGST1" "TI_R_P3"

"99" 1.88745823368047e-60 4.54159507704148 0.84 0.651 6.30580921290307e-56 "PR2" "NDUFS6" "TI_R_P3"

"100" 2.49023585899184e-60 3.95614605190308 0.951 0.801 8.31962898130584e-56 "PR2" "ATP6V0E1" "TI_R_P3"

"101" 4.6881066266655e-60 1.89889875887999 0.926 0.748 1.56624954290268e-55 "PR2" "UBA1" "TI_R_P3"

"102" 6.57945247312351e-60 3.52441382336493 0.956 0.802 2.19812927674583e-55 "PR2" "ATP5MD" "TI_R_P3"

"103" 1.323623831739e-59 3.02717607732585 0.763 0.487 4.42209485945682e-55 "PR2" "TSTD1" "TI_R_P3"

"104" 1.36246915549444e-59 1.33109379503531 0.911 0.737 4.55187320159138e-55 "PR2" "CD9" "TI_R_P3"

"105" 1.46545427168712e-59 2.45325151879176 0.67 0.362 4.89593617627949e-55 "PR2" "LIPH" "TI_R_P3"

"106" 1.47838133995706e-59 3.38292867521726 0.939 0.789 4.93912421866255e-55 "PR2" "MDM2" "TI_R_P3"

"107" 2.64712063043781e-59 1.34848400667522 0.823 0.54 8.84376531422967e-55 "PR2" "MPZL2" "TI_R_P3"

"108" 3.29142735015022e-59 6.09839660150868 0.833 0.601 1.09963296341169e-54 "PR2" "BHLHE40" "TI_R_P3"

"109" 4.18049610800353e-59 4.04185432886456 0.898 0.685 1.3966619447229e-54 "PR2" "SNRPG" "TI_R_P3"

"110" 4.40933483998527e-59 6.78206653349146 0.849 0.625 1.47311467669068e-54 "PR2" "HMGN3" "TI_R_P3"

"111" 6.94679509314852e-59 2.7523357226751 0.851 0.619 2.32085477266999e-54 "PR2" "WFDC3" "TI_R_P3"

"112" 1.14889246618998e-58 1.37488646674032 0.514 0.216 3.83833484029409e-54 "PR2" "HPGD" "TI_R_P3"

"113" 1.19345173667603e-58 1.20972268099966 0.898 0.692 3.98720290706094e-54 "PR2" "NONO" "TI_R_P3"

"114" 2.24319249631812e-58 2.9958098005051 0.882 0.664 7.49428181094921e-54 "PR2" "CD46" "TI_R_P3"

"115" 2.6088538468423e-58 2.79026253568716 0.882 0.689 8.71591981691544e-54 "PR2" "TESC" "TI_R_P3"

"116" 2.87815606371327e-58 1.74020094113546 0.868 0.632 9.61563159325965e-54 "PR2" "CCT2" "TI_R_P3"

"117" 3.35942782102439e-58 1.43350732524779 0.746 0.451 1.12235124072604e-53 "PR2" "C1orf116" "TI_R_P3"

"118" 7.47749944978255e-58 2.26222914078796 0.895 0.719 2.49815779117785e-53 "PR2" "SCD" "TI_R_P3"

"119" 8.316733166815e-58 2.38736203886766 0.802 0.564 2.77853738370122e-53 "PR2" "CLDN7" "TI_R_P3"

"120" 1.05951912939199e-57 1.45465859269161 0.489 0.203 3.53974745938571e-53 "PR2" "AC104031.1" "TI_R_P3"

"121" 1.16246167416896e-57 6.96781396937554 0.925 0.762 3.88366820723107e-53 "PR2" "PLS3" "TI_R_P3"

"122" 1.26725420563635e-57 1.94218488136194 0.561 0.265 4.23376957561048e-53 "PR2" "EIF1AY" "TI_R_P3"

"123" 1.70864449047048e-57 19.5259494669541 0.961 0.844 5.70841037821284e-53 "PR2" "CHCHD2" "TI_R_P3"

"124" 3.05790098083584e-57 2.32563846787881 0.756 0.479 1.02161413868745e-52 "PR2" "SORL1" "TI_R_P3"

"125" 5.88071167402205e-57 9.34607105513289 0.933 0.797 1.96468696317403e-52 "PR2" "HSPA8" "TI_R_P3"

"126" 8.70515058075185e-57 1.48921048234719 0.563 0.265 2.90830375752339e-52 "PR2" "MSLN" "TI_R_P3"

"127" 9.20819912890342e-57 1.56320191540977 0.788 0.516 3.07636724697534e-52 "PR2" "DDR1" "TI_R_P3"

"128" 3.22254538948911e-56 1.16531498176711 0.712 0.434 1.07662018917442e-51 "PR2" "MACC1" "TI_R_P3"

"129" 4.1145282735747e-56 1.13450677661941 0.893 0.726 1.37462275091857e-51 "PR2" "NAA38" "TI_R_P3"

"130" 5.49469937947799e-56 1.0663134477358 0.821 0.542 1.8357241156898e-51 "PR2" "GPAA1" "TI_R_P3"

"131" 6.60179009307933e-56 2.96914298643327 0.818 0.552 2.20559205219687e-51 "PR2" "ANAPC16" "TI_R_P3"

"132" 1.02527472092184e-55 1.49804335437604 0.672 0.391 3.42534031512778e-51 "PR2" "FNIP2" "TI_R_P3"

"133" 2.05793746824357e-55 1.11128734141923 0.968 0.859 6.87536328765493e-51 "PR2" "ARF1" "TI_R_P3"

"134" 2.97512750675361e-55 1.20045888696055 0.87 0.624 9.93960348731314e-51 "PR2" "SOX4" "TI_R_P3"

"135" 3.40804789738937e-55 2.62612134183962 0.828 0.579 1.13859472203882e-50 "PR2" "TMEM98" "TI_R_P3"

"136" 3.49555347805588e-55 1.50013224615282 0.789 0.492 1.16782946148369e-50 "PR2" "ARSD" "TI_R_P3"

"137" 3.72456733563209e-55 5.75761703880152 0.835 0.627 1.24434070116132e-50 "PR2" "CYB5A" "TI_R_P3"

"138" 4.57149079243948e-55 3.14159816729874 0.875 0.661 1.5272893588461e-50 "PR2" "USP22" "TI_R_P3"

"139" 4.59732538664378e-55 2.04195878627011 0.653 0.353 1.53592043842382e-50 "PR2" "SOD3" "TI_R_P3"

"140" 2.69050173830559e-54 2.56461222125512 0.93 0.796 8.98869725750514e-50 "PR2" "ATP5MF" "TI_R_P3"

"141" 4.301698832169e-54 3.60471281513921 0.863 0.661 1.43715456283934e-49 "PR2" "ANAPC11" "TI_R_P3"

"142" 4.67778293280962e-54 1.99222094380879 0.949 0.79 1.56280050002237e-49 "PR2" "HSPA5" "TI_R_P3"

"143" 4.95542777966414e-54 5.00977136637018 0.942 0.837 1.65555886690799e-49 "PR2" "TOMM7" "TI_R_P3"

"144" 8.22427566019072e-54 5.78979120683443 0.912 0.712 2.74764825531312e-49 "PR2" "SEM1" "TI_R_P3"

"145" 1.1396761641099e-53 1.97463596783943 0.905 0.729 3.80754409667477e-49 "PR2" "NDUFB2" "TI_R_P3"

"146" 1.50889601879471e-53 3.87992836705117 0.975 0.854 5.04107070919126e-49 "PR2" "DDX5" "TI_R_P3"

"147" 1.5442134830563e-53 1.38150350186807 0.54 0.253 5.1590628255428e-49 "PR2" "GGTLC1" "TI_R_P3"

"148" 1.90206090865738e-53 1.05002177502363 0.967 0.843 6.35459528973346e-49 "PR2" "COX6A1" "TI_R_P3"

"149" 2.58916961912697e-53 1.37239798104541 0.663 0.366 8.65015678054128e-49 "PR2" "PTPRN2" "TI_R_P3"

"150" 3.59332012531906e-53 1.60334779320548 0.837 0.589 1.20049232066785e-48 "PR2" "FLNB" "TI_R_P3"

"151" 3.6804169800883e-53 1.15586545612445 0.43 0.161 1.2295905088777e-48 "PR2" "MACROD2" "TI_R_P3"

"152" 3.98304183764504e-53 1.0040590957024 0.63 0.333 1.33069444753883e-48 "PR2" "ETV4" "TI_R_P3"

"153" 4.45740559994167e-53 2.93223089376535 0.861 0.643 1.48917463688451e-48 "PR2" "CAPN1" "TI_R_P3"

"154" 1.36252872364073e-52 1.31100281928521 0.825 0.563 4.55207221281132e-48 "PR2" "ETNK1" "TI_R_P3"

"155" 1.37066574523988e-52 1.04421457491392 0.456 0.179 4.5792571882719e-48 "PR2" "TSPAN8" "TI_R_P3"

"156" 2.21304631795954e-52 3.50807198515962 0.647 0.345 7.39356644367102e-48 "PR2" "MSMO1" "TI_R_P3"

"157" 2.98357140565707e-52 1.38394719326672 0.851 0.639 9.96781370915969e-48 "PR2" "COX17" "TI_R_P3"

"158" 3.07056291874044e-52 5.8333613954355 0.93 0.769 1.02584436552199e-47 "PR2" "SUMO2" "TI_R_P3"

"159" 4.6007297380448e-52 1.48927462960975 0.625 0.33 1.53705779818339e-47 "PR2" "SUSD2" "TI_R_P3"

"160" 5.40820861803941e-52 1.82651243502226 0.763 0.511 1.80682841720079e-47 "PR2" "CDH1" "TI_R_P3"

"161" 6.58519314342692e-52 2.25944676762626 0.828 0.614 2.2000471772875e-47 "PR2" "SPCS1" "TI_R_P3"

"162" 8.48373859182547e-52 1.64556309888503 0.812 0.555 2.83433222614297e-47 "PR2" "PTPRF" "TI_R_P3"

"163" 1.84651526892634e-51 1.79124278252932 0.937 0.786 6.16902286195602e-47 "PR2" "SOD1" "TI_R_P3"

"164" 2.2972348276111e-51 2.00670008563436 0.821 0.636 7.67483183556591e-47 "PR2" "VAMP5" "TI_R_P3"

"165" 3.18638245931697e-51 1.23456682924772 0.663 0.373 1.06453851583321e-46 "PR2" "QPRT" "TI_R_P3"

"166" 3.59872152320751e-51 1.93611982840383 0.782 0.53 1.2022968736884e-46 "PR2" "GPC4" "TI_R_P3"

"167" 4.36983946656828e-51 2.75409206587913 0.881 0.67 1.4599196673858e-46 "PR2" "HSPD1" "TI_R_P3"

"168" 4.70693731965486e-51 1.66001601423664 0.891 0.693 1.57254068912349e-46 "PR2" "SERINC2" "TI_R_P3"

"169" 4.74837293773022e-51 2.35274587380526 0.763 0.536 1.58638391476629e-46 "PR2" "ERBB3" "TI_R_P3"

"170" 5.98355495991253e-51 1.88163316103057 0.804 0.554 1.99904587655718e-46 "PR2" "MARCKSL1" "TI_R_P3"

"171" 6.18942018787516e-51 1.14677535260966 0.895 0.704 2.06782339056721e-46 "PR2" "YWHAQ" "TI_R_P3"

"172" 6.65615666016795e-51 1.10686163049327 0.611 0.324 2.22375537859551e-46 "PR2" "TMEM41B" "TI_R_P3"

"173" 7.6819473935932e-51 3.34901464625497 0.872 0.662 2.56646180472555e-46 "PR2" "PRPF8" "TI_R_P3"

"174" 8.13236614440189e-51 4.51727869864468 0.781 0.524 2.71694220518323e-46 "PR2" "CEBPD" "TI_R_P3"

"175" 9.8908714122781e-51 1.89082000427956 0.758 0.495 3.30444123012799e-46 "PR2" "MYO1D" "TI_R_P3"

"176" 1.22543701638509e-50 1.92564335908566 0.882 0.667 4.09406252804094e-46 "PR2" "TNFRSF12A" "TI_R_P3"

"177" 1.54529913708142e-50 1.61893639993946 0.807 0.564 5.16268988707533e-46 "PR2" "NDUFA6" "TI_R_P3"

"178" 2.02816566842402e-50 5.70702737882425 0.968 0.865 6.77589868163781e-46 "PR2" "MYL12A" "TI_R_P3"

"179" 2.99241111543908e-50 2.73862421744104 0.733 0.455 9.99734629557042e-46 "PR2" "PACSIN2" "TI_R_P3"

"180" 3.15101316424001e-50 2.61110180200116 0.693 0.417 1.05272198804094e-45 "PR2" "C5orf38" "TI_R_P3"

"181" 6.39688847829242e-50 1.22840795644694 0.918 0.746 2.13713647171271e-45 "PR2" "SYNGR2" "TI_R_P3"

"182" 7.67054444861886e-50 2.63581889184928 0.832 0.612 2.56265219483907e-45 "PR2" "PPA1" "TI_R_P3"

"183" 1.10152547448677e-49 1.81429032064706 0.911 0.755 3.68008645771284e-45 "PR2" "PARK7" "TI_R_P3"

"184" 1.15750306734073e-49 4.43892808637238 0.889 0.691 3.86710199767865e-45 "PR2" "TALDO1" "TI_R_P3"

"185" 1.39211714868733e-49 2.79516817751276 0.928 0.812 4.65092418204949e-45 "PR2" "COX8A" "TI_R_P3"

"186" 2.06626555871473e-49 5.15143169159323 0.954 0.838 6.90318660511005e-45 "PR2" "DSTN" "TI_R_P3"

"187" 2.87307841001008e-49 1.25166778097299 0.814 0.57 9.59866766000267e-45 "PR2" "GANAB" "TI_R_P3"

"188" 2.9574788289598e-49 1.70495383147336 0.958 0.834 9.88064101967181e-45 "PR2" "UBL5" "TI_R_P3"

"189" 3.33574150455748e-49 1.70686190729128 0.823 0.585 1.11443787925761e-44 "PR2" "TGFBR2" "TI_R_P3"

"190" 4.08048140213592e-49 1.39263618421815 0.788 0.528 1.36324803163959e-44 "PR2" "SCOC" "TI_R_P3"

"191" 4.97541822647158e-49 1.34544617760039 0.547 0.267 1.66223747528189e-44 "PR2" "AKAP17A" "TI_R_P3"

"192" 6.91211744831343e-49 1.17883944235441 0.561 0.281 2.30926931830703e-44 "PR2" "EGFR" "TI_R_P3"

"193" 1.53193305530761e-48 1.40692411961721 0.57 0.29 5.11803514447718e-44 "PR2" "TMEM30B" "TI_R_P3"

"194" 1.5680592010232e-48 2.69535414511983 0.914 0.781 5.23872898469841e-44 "PR2" "ANXA11" "TI_R_P3"

"195" 1.83420704276678e-48 4.63570492085871 0.942 0.837 6.12790230917953e-44 "PR2" "EEF1B2" "TI_R_P3"

"196" 2.93097300512462e-48 2.15420898322886 0.86 0.672 9.79208771282084e-44 "PR2" "H2AFV" "TI_R_P3"

"197" 6.71278676137808e-48 1.80100301429944 0.9 0.703 2.2426749291088e-43 "PR2" "HNRNPA3" "TI_R_P3"

"198" 1.73626248691632e-47 2.97550326337101 0.898 0.721 5.80067934253873e-43 "PR2" "HNRNPU" "TI_R_P3"

"199" 1.78817668561721e-47 2.10134979654087 0.809 0.599 5.97411948897853e-43 "PR2" "RNF181" "TI_R_P3"

"200" 2.08469091413582e-47 1.48810415997406 0.521 0.249 6.96474387503637e-43 "PR2" "PPP2R3B" "TI_R_P3"

"201" 2.30861171101383e-47 4.43433932003338 0.918 0.775 7.71284086532609e-43 "PR2" "MZT2B" "TI_R_P3"

"202" 2.45365549969909e-47 1.23596342553294 0.853 0.633 8.19741765894468e-43 "PR2" "ZDHHC9" "TI_R_P3"

"203" 3.39479573637035e-47 2.95401608341547 0.709 0.439 1.13416730756397e-42 "PR2" "PIN4" "TI_R_P3"

"204" 3.7803408123474e-47 1.30833222347539 0.612 0.331 1.26297406199714e-42 "PR2" "DPP4" "TI_R_P3"

"205" 5.21342768785429e-47 1.81214021792939 0.661 0.395 1.74175405623524e-42 "PR2" "VKORC1L1" "TI_R_P3"

"206" 5.62314693854931e-47 1.09326779706866 0.877 0.658 1.87863716069994e-42 "PR2" "REEP5" "TI_R_P3"

"207" 6.32076871887702e-47 1.02584935577749 0.642 0.365 2.11170562128962e-42 "PR2" "WDR33" "TI_R_P3"

"208" 9.52176349517652e-47 1.23722614813276 0.63 0.352 3.18112596610352e-42 "PR2" "NEDD4L" "TI_R_P3"

"209" 9.91076780741127e-47 1.52636202787626 0.905 0.742 3.31108841677803e-42 "PR2" "ATP6AP1" "TI_R_P3"

"210" 1.49200920497862e-46 1.81349677550238 0.886 0.729 4.98465355291306e-42 "PR2" "XRCC6" "TI_R_P3"

"211" 1.79067176918393e-46 6.54366155889164 0.94 0.82 5.98245531366659e-42 "PR2" "HNRNPA2B1" "TI_R_P3"

"212" 1.94239582938889e-46 1.1645987694566 0.886 0.706 6.48935022640534e-42 "PR2" "CD164" "TI_R_P3"

"213" 1.9509942794678e-46 1.71437960470624 0.744 0.5 6.51807678827398e-42 "PR2" "NDUFAF8" "TI_R_P3"

"214" 1.97662208991839e-46 7.51380839423275 0.961 0.817 6.60369674020837e-42 "PR2" "HNRNPK" "TI_R_P3"

"215" 3.20318973980627e-46 4.87893963214456 0.861 0.661 1.07015366017188e-41 "PR2" "PPP1CA" "TI_R_P3"

"216" 3.21754936391493e-46 2.04996909726098 0.733 0.481 1.07495106699034e-41 "PR2" "HUWE1" "TI_R_P3"

"217" 3.26096619172211e-46 1.2112393211327 0.7 0.462 1.08945619499244e-41 "PR2" "FDFT1" "TI_R_P3"

"218" 3.83376067307276e-46 1.64958625735566 0.749 0.473 1.28082110326688e-41 "PR2" "AEBP2" "TI_R_P3"

"219" 3.91663532464871e-46 2.07904548468785 0.705 0.441 1.30850869561189e-41 "PR2" "MYO6" "TI_R_P3"

"220" 6.28954314218408e-46 3.1581592822623 0.804 0.572 2.10127346837228e-41 "PR2" "SIVA1" "TI_R_P3"

"221" 6.91888179825756e-46 1.25770239699761 0.888 0.721 2.31152921997987e-41 "PR2" "COMT" "TI_R_P3"

"222" 7.94753094924396e-46 1.92116627271063 0.8 0.576 2.65519061483292e-41 "PR2" "HLA-DMB" "TI_R_P3"

"223" 1.08609356498325e-45 1.96760845814789 0.593 0.319 3.62852999125255e-41 "PR2" "HABP2" "TI_R_P3"

"224" 1.54515098555968e-45 1.4881545250101 0.781 0.534 5.16219492765634e-41 "PR2" "CDK2AP2" "TI_R_P3"

"225" 1.85817122385241e-45 1.15609710856964 0.854 0.676 6.20796424176852e-41 "PR2" "SRRM2" "TI_R_P3"

"226" 2.06401325755244e-45 1.61388830315151 0.796 0.562 6.89566189215696e-41 "PR2" "ABHD2" "TI_R_P3"

"227" 2.21904641684164e-45 2.51092031134109 0.751 0.508 7.41361217402623e-41 "PR2" "C7orf50" "TI_R_P3"

"228" 2.77057912071948e-45 3.14666080456682 0.765 0.521 9.25622778441173e-41 "PR2" "TMEM256" "TI_R_P3"

"229" 2.88625160630139e-45 1.24928779024256 0.707 0.435 9.64267799149233e-41 "PR2" "RAB27A" "TI_R_P3"

"230" 3.11939935699108e-45 1.31431393655353 0.661 0.398 1.04216013117715e-40 "PR2" "RASSF7" "TI_R_P3"

"231" 3.15829806768872e-45 4.07456721311885 0.821 0.604 1.05515580143412e-40 "PR2" "FIS1" "TI_R_P3"

"232" 3.29866031551152e-45 2.90875141618489 0.884 0.736 1.10204942480924e-40 "PR2" "ATP6AP2" "TI_R_P3"

"233" 3.3714955382683e-45 1.80153352259865 0.73 0.474 1.12638294438006e-40 "PR2" "TSFM" "TI_R_P3"

"234" 4.19259507393649e-45 2.12439371535666 0.819 0.619 1.40070408825144e-40 "PR2" "IRF2BP2" "TI_R_P3"

"235" 5.91848836961151e-45 1.2393471515251 0.53 0.257 1.97730777940351e-40 "PR2" "SLC16A7" "TI_R_P3"

"236" 6.0083674752574e-45 1.61891909679031 0.775 0.514 2.00733548980874e-40 "PR2" "SPTBN1" "TI_R_P3"

"237" 6.01994339593786e-45 2.21423086332586 0.775 0.537 2.01120288914888e-40 "PR2" "MGAT4B" "TI_R_P3"

"238" 6.0429414979218e-45 1.25249106599108 0.756 0.505 2.01888632504069e-40 "PR2" "PCBD1" "TI_R_P3"

"239" 6.41286533537869e-45 2.95764919895964 0.737 0.479 2.14247417989667e-40 "PR2" "TSPAN6" "TI_R_P3"

"240" 7.53667765334488e-45 6.33770371378301 0.846 0.655 2.51792863720599e-40 "PR2" "SELENOH" "TI_R_P3"

"241" 9.45107943272723e-45 1.64676889209904 0.719 0.448 3.15751112767984e-40 "PR2" "SH3BGRL2" "TI_R_P3"

"242" 1.12488165216292e-44 2.02314643839692 0.674 0.42 3.75811711171109e-40 "PR2" "CDKN2A" "TI_R_P3"

"243" 1.19940368862908e-44 2.18695248974996 0.791 0.579 4.00708778334088e-40 "PR2" "PERP" "TI_R_P3"

"244" 1.49834596623271e-44 2.78041060040752 0.868 0.673 5.00582403858686e-40 "PR2" "HLA-F" "TI_R_P3"

"245" 1.70125843963478e-44 4.39455596414688 0.882 0.678 5.68373432097584e-40 "PR2" "ACADVL" "TI_R_P3"

"246" 2.24218368757179e-44 1.1896223687502 0.567 0.301 7.4909114818086e-40 "PR2" "STK39" "TI_R_P3"

"247" 2.64846779850618e-44 1.05848728311808 0.596 0.336 8.84826606802928e-40 "PR2" "SLC25A4" "TI_R_P3"

"248" 3.37688613827245e-44 3.73351162146593 0.96 0.837 1.12818388993544e-39 "PR2" "GLUL" "TI_R_P3"

"249" 3.60417094663527e-44 1.4031792415249 0.795 0.552 1.20411747156138e-39 "PR2" "CREG1" "TI_R_P3"

"250" 4.04100851848784e-44 1.5033250447938 0.602 0.333 1.3500605359416e-39 "PR2" "OCLN" "TI_R_P3"

"251" 4.94802996555584e-44 1.83502740585034 0.721 0.47 1.65308733119255e-39 "PR2" "MGAT5" "TI_R_P3"

"252" 5.30397295799165e-44 3.18201903854298 0.837 0.61 1.77200432553543e-39 "PR2" "KDELR1" "TI_R_P3"

"253" 6.24526882801943e-44 1.04767515679442 0.511 0.248 2.08648186275301e-39 "PR2" "TEPP" "TI_R_P3"

"254" 8.40434842765747e-44 1.65286811530782 0.567 0.301 2.80780876619608e-39 "PR2" "DDX3Y" "TI_R_P3"

"255" 9.18659772529898e-44 1.31548518783731 0.698 0.44 3.06915043404514e-39 "PR2" "EPDR1" "TI_R_P3"

"256" 9.33968466651687e-44 1.161200155624 0.814 0.581 3.12029525023662e-39 "PR2" "SNRNP200" "TI_R_P3"

"257" 1.16829538107883e-43 4.96357741577684 0.828 0.603 3.90315803864627e-39 "PR2" "PLXND1" "TI_R_P3"

"258" 1.23291246466019e-43 1.55660898854924 0.856 0.663 4.11903725318321e-39 "PR2" "MORF4L2" "TI_R_P3"

"259" 1.3003332472841e-43 1.2633094257461 0.498 0.242 4.34428334585146e-39 "PR2" "GTPBP6" "TI_R_P3"

"260" 1.38013239966802e-43 1.75713855438518 0.746 0.498 4.61088433405088e-39 "PR2" "MAPK13" "TI_R_P3"

"261" 1.46819504415796e-43 2.32130934128651 0.774 0.55 4.90509282302731e-39 "PR2" "NBDY" "TI_R_P3"

"262" 1.48074554384116e-43 3.26345386012402 0.905 0.743 4.94702278741892e-39 "PR2" "PDIA6" "TI_R_P3"

"263" 1.73510597480952e-43 3.37875774643302 0.77 0.56 5.79681555124113e-39 "PR2" "MRPL20" "TI_R_P3"

"264" 1.7689264368929e-43 1.78951566688971 0.795 0.57 5.90980633301548e-39 "PR2" "CERS2" "TI_R_P3"

"265" 2.27409894537583e-43 1.86898523943625 0.902 0.741 7.59753716660612e-39 "PR2" "NDUFA2" "TI_R_P3"

"266" 2.86212813109038e-43 1.2505533266173 0.684 0.416 9.56208387315984e-39 "PR2" "HLA-DOA" "TI_R_P3"

"267" 3.79639065897185e-43 1.77951887587985 0.849 0.611 1.2683361552559e-38 "PR2" "AZIN1" "TI_R_P3"

"268" 4.07358538085664e-43 3.6597359686243 0.837 0.65 1.36094413989039e-38 "PR2" "H2AFZ" "TI_R_P3"

"269" 4.2890932373153e-43 1.78208879718359 0.689 0.444 1.43294315965467e-38 "PR2" "HMGB3" "TI_R_P3"

"270" 4.89891151019854e-43 3.48445393057145 0.646 0.395 1.63667734644223e-38 "PR2" "OGT" "TI_R_P3"

"271" 5.17073988511974e-43 2.16429404429797 0.956 0.841 1.72749248821966e-38 "PR2" "CANX" "TI_R_P3"

"272" 5.44356308141542e-43 1.51546252207074 0.777 0.528 1.81863998987008e-38 "PR2" "HNRNPF" "TI_R_P3"

"273" 5.80404951954302e-43 3.92705398637172 0.751 0.513 1.93907490398413e-38 "PR2" "UXT" "TI_R_P3"

"274" 6.33384144804124e-43 3.39706752793826 0.793 0.58 2.1160730893761e-38 "PR2" "TBCA" "TI_R_P3"

"275" 8.04672840488622e-43 2.73868318681073 0.968 0.849 2.68833149278844e-38 "PR2" "CD151" "TI_R_P3"

"276" 8.73366710091833e-43 2.10948373722315 0.753 0.525 2.91783084174581e-38 "PR2" "JUN" "TI_R_P3"

"277" 8.90424096917341e-43 1.57590647683915 0.461 0.206 2.97481786539114e-38 "PR2" "VSIG2" "TI_R_P3"

"278" 9.19415249561925e-43 1.13310206241508 0.877 0.674 3.07167440726143e-38 "PR2" "RBX1" "TI_R_P3"

"279" 1.01227777992592e-42 1.3732732418692 0.654 0.391 3.38191883495452e-38 "PR2" "TST" "TI_R_P3"

"280" 1.50057752642508e-42 1.64405650278808 0.679 0.427 5.01327945803355e-38 "PR2" "FDPS" "TI_R_P3"

"281" 1.53395451040587e-42 2.57317854521468 0.612 0.367 5.12478862381495e-38 "PR2" "DHRSX" "TI_R_P3"

"282" 1.92137008602176e-42 1.69592988845296 0.846 0.653 6.41910532039009e-38 "PR2" "CIB1" "TI_R_P3"

"283" 2.57915269077748e-42 1.57528559957038 0.442 0.196 8.61669122461847e-38 "PR2" "GJB1" "TI_R_P3"

"284" 3.12401190481766e-42 2.84950437680707 0.923 0.806 1.04370113728053e-37 "PR2" "ATP5MC2" "TI_R_P3"

"285" 4.30045797967215e-42 3.41799506145157 0.889 0.72 1.43674000642867e-37 "PR2" "NDUFB4" "TI_R_P3"

"286" 4.75829401142836e-42 1.2787704585758 0.46 0.207 1.5896984462781e-37 "PR2" "PRR15L" "TI_R_P3"

"287" 6.65644649092645e-42 1.70327820441293 0.749 0.529 2.22385220815362e-37 "PR2" "BLVRA" "TI_R_P3"

"288" 7.21081643590347e-42 3.10450680216982 0.893 0.729 2.40906166307099e-37 "PR2" "TAF10" "TI_R_P3"

"289" 1.10517691485095e-41 1.16482529000391 0.911 0.749 3.69228555482554e-37 "PR2" "NDUFB1" "TI_R_P3"

"290" 1.35945610317216e-41 3.96536893902441 0.826 0.635 4.54180689508787e-37 "PR2" "HOPX" "TI_R_P3"

"291" 2.25415804682704e-41 3.13907890482232 0.916 0.774 7.53091661864445e-37 "PR2" "HNRNPC" "TI_R_P3"

"292" 2.48026972206273e-41 1.435811367626 0.804 0.597 8.28633311443936e-37 "PR2" "RALY" "TI_R_P3"

"293" 2.54152248569654e-41 1.29726230635405 0.791 0.558 8.49097247246357e-37 "PR2" "EIF1AX" "TI_R_P3"

"294" 3.24118353091178e-41 1.32430290064041 0.672 0.405 1.08284700584232e-36 "PR2" "EPS8L2" "TI_R_P3"

"295" 3.55878272661575e-41 2.37776426277512 0.84 0.652 1.18895372113505e-36 "PR2" "CUTA" "TI_R_P3"

"296" 3.81208349188767e-41 1.87360412953113 0.698 0.47 1.27357897380475e-36 "PR2" "SEC11C" "TI_R_P3"

"297" 5.97116686384883e-41 2.27496622036507 0.74 0.516 1.99490713754325e-36 "PR2" "NEU1" "TI_R_P3"

"298" 7.59747333520934e-41 1.58813027458873 0.726 0.492 2.53823986656009e-36 "PR2" "MID1IP1" "TI_R_P3"

"299" 7.62392209327994e-41 2.07351351958612 0.847 0.66 2.54707613214389e-36 "PR2" "EIF3E" "TI_R_P3"

"300" 1.78796852530365e-40 2.27980348720225 0.728 0.489 5.97342404618696e-36 "PR2" "COA3" "TI_R_P3"

"301" 2.15473710617886e-40 3.09553130713965 0.87 0.67 7.19876119803295e-36 "PR2" "CHMP2A" "TI_R_P3"

"302" 3.06044341330634e-40 1.87989839641298 0.828 0.611 1.02246353995151e-35 "PR2" "BANF1" "TI_R_P3"

"303" 3.57621915554324e-40 1.95050509165612 0.43 0.198 1.19477905767544e-35 "PR2" "SLPI" "TI_R_P3"

"304" 3.93300681094927e-40 2.87899167894469 0.863 0.682 1.31397824547004e-35 "PR2" "NCL" "TI_R_P3"

"305" 4.50759525529984e-40 1.64373046170096 0.693 0.452 1.50594249884312e-35 "PR2" "TMEM54" "TI_R_P3"

"306" 7.45446336644653e-40 1.16217687009645 0.477 0.235 2.49046166609612e-35 "PR2" "CAPN8" "TI_R_P3"

"307" 7.53958991522034e-40 2.40642476648394 0.911 0.741 2.51890159477596e-35 "PR2" "OS9" "TI_R_P3"

"308" 8.03435056368573e-40 1.8126629013182 0.881 0.694 2.68419617982177e-35 "PR2" "COPS9" "TI_R_P3"

"309" 8.51855380290616e-40 1.29433267036542 0.847 0.639 2.84596364001292e-35 "PR2" "ECHS1" "TI_R_P3"

"310" 8.54939299100207e-40 1.00596878994271 0.57 0.325 2.85626670436388e-35 "PR2" "HYI" "TI_R_P3"

"311" 8.55244643488187e-40 1.70429761377672 0.64 0.4 2.85728682942968e-35 "PR2" "ERG28" "TI_R_P3"

"312" 1.04201865609096e-39 1.18440157791112 0.949 0.847 3.4812801281343e-35 "PR2" "TXNIP" "TI_R_P3"

"313" 1.33723814150235e-39 1.11188134371057 0.575 0.307 4.46757890694521e-35 "PR2" "SLC6A20" "TI_R_P3"

"314" 1.54654894708325e-39 1.60937098991247 0.804 0.618 5.16686537731043e-35 "PR2" "NDUFC2" "TI_R_P3"

"315" 1.70913017597451e-39 2.09076477217309 0.691 0.44 5.71003300491325e-35 "PR2" "SLC44A4" "TI_R_P3"

"316" 1.7733512306147e-39 4.49105427193772 0.872 0.69 5.92458912636065e-35 "PR2" "LAMTOR4" "TI_R_P3"

"317" 1.96237778083677e-39 1.22004160750572 0.914 0.737 6.55610792799757e-35 "PR2" "RRBP1" "TI_R_P3"

"318" 2.03218371895872e-39 1.26080986516308 0.723 0.454 6.78932258666917e-35 "PR2" "COX20" "TI_R_P3"

"319" 2.46679179943685e-39 2.28198979519435 0.847 0.668 8.24130472273856e-35 "PR2" "ATP5PF" "TI_R_P3"

"320" 2.48530114627937e-39 1.60818353244519 0.593 0.342 8.30314259960474e-35 "PR2" "HACD3" "TI_R_P3"

"321" 2.78369390425623e-39 1.37898679305383 0.458 0.217 9.30004296472964e-35 "PR2" "HSD17B6" "TI_R_P3"

"322" 3.20052452925789e-39 1.58859111466177 0.418 0.184 1.06926323997977e-34 "PR2" "CYP4B1" "TI_R_P3"

"323" 3.91454910222722e-39 1.12453243216839 0.495 0.241 1.30781170956309e-34 "PR2" "SCIN" "TI_R_P3"

"324" 4.50105239964506e-39 1.57783179698483 0.586 0.337 1.50375659619742e-34 "PR2" "MRPL36" "TI_R_P3"

"325" 5.74681193572173e-39 3.1747778544844 0.798 0.611 1.91995239960527e-34 "PR2" "SRI" "TI_R_P3"

"326" 6.35903047127154e-39 1.4442559992364 0.87 0.691 2.12448849014711e-34 "PR2" "LRPAP1" "TI_R_P3"

"327" 7.84908029287462e-39 1.82337124742865 0.621 0.368 2.62229923504648e-34 "PR2" "EBP" "TI_R_P3"

"328" 8.56769690247135e-39 1.53016816584726 0.807 0.6 2.86238185814665e-34 "PR2" "PHC2" "TI_R_P3"

"329" 9.0267890337769e-39 1.05379503142363 0.804 0.581 3.01575994829453e-34 "PR2" "ATP6V1E1" "TI_R_P3"

"330" 9.10205176663849e-39 1.43290802970089 0.935 0.78 3.04090447471625e-34 "PR2" "PLXNB2" "TI_R_P3"

"331" 9.64679491793365e-39 1.25393919940604 0.718 0.481 3.22289771413245e-34 "PR2" "RARRES3" "TI_R_P3"

"332" 1.45515864845181e-38 1.27634739988732 0.712 0.486 4.86153952861265e-34 "PR2" "UQCC2" "TI_R_P3"

"333" 1.9036461407154e-38 1.61475189854835 0.602 0.361 6.35989139151608e-34 "PR2" "ENDOD1" "TI_R_P3"

"334" 2.03521212523883e-38 1.93338218050709 0.496 0.257 6.79944018921042e-34 "PR2" "NT5E" "TI_R_P3"

"335" 2.18161258529508e-38 1.58869651872037 0.681 0.446 7.28854948621234e-34 "PR2" "F11R" "TI_R_P3"

"336" 2.30546196277381e-38 2.24741474267139 0.867 0.672 7.70231787143101e-34 "PR2" "S100A13" "TI_R_P3"

"337" 2.33675784099152e-38 1.72230860712458 0.835 0.632 7.80687427096858e-34 "PR2" "VAPA" "TI_R_P3"

"338" 2.80255650659886e-38 1.01108370500267 0.544 0.296 9.36306103289613e-34 "PR2" "ST6GALNAC1" "TI_R_P3"

"339" 3.43471839164598e-38 1.77809488159875 0.798 0.601 1.14750506746501e-33 "PR2" "TUFM" "TI_R_P3"

"340" 3.8847505543212e-38 1.03003624608013 0.711 0.493 1.29785631269317e-33 "PR2" "TCEAL8" "TI_R_P3"

"341" 4.54447837637578e-38 1.80486996754724 0.712 0.487 1.51826478076339e-33 "PR2" "LMO7" "TI_R_P3"

"342" 4.79750380295327e-38 1.15273010144732 0.649 0.391 1.60279804552866e-33 "PR2" "SMARCA5" "TI_R_P3"

"343" 4.86925367011592e-38 3.67659760829457 0.933 0.822 1.62676895864903e-33 "PR2" "UQCR10" "TI_R_P3"

"344" 5.71664829833633e-38 1.22719209724611 0.546 0.301 1.90987502999119e-33 "PR2" "FAM3B" "TI_R_P3"

"345" 7.5648803675837e-38 3.50510951751083 0.812 0.629 2.52735088200604e-33 "PR2" "SRP9" "TI_R_P3"

"346" 7.72275461828594e-38 1.05098939699823 0.593 0.342 2.58009509042315e-33 "PR2" "SC5D" "TI_R_P3"

"347" 8.22338287117563e-38 1.26355242370989 0.628 0.377 2.74734998343107e-33 "PR2" "MPST" "TI_R_P3"

"348" 8.99465020242967e-38 2.52140920264008 0.847 0.658 3.00502268612973e-33 "PR2" "ST13" "TI_R_P3"

"349" 1.24147395053957e-37 1.2064958385976 0.67 0.441 4.14764032135766e-33 "PR2" "SYPL1" "TI_R_P3"

"350" 1.26378864402206e-37 1.53484176908146 0.649 0.419 4.22219148081331e-33 "PR2" "ETFB" "TI_R_P3"

"351" 1.56056448489192e-37 1.67236912036677 0.746 0.507 5.21368988757542e-33 "PR2" "SLC12A7" "TI_R_P3"

"352" 1.7697026924819e-37 1.26086369802583 0.74 0.522 5.91239972531277e-33 "PR2" "CCPG1" "TI_R_P3"

"353" 2.01247590079498e-37 1.05452308601642 0.511 0.269 6.72348073696596e-33 "PR2" "TMEM191B" "TI_R_P3"

"354" 2.33094817328354e-37 1.6165668038582 0.595 0.356 7.78746475212297e-33 "PR2" "ZNF117" "TI_R_P3"

"355" 2.6753689336213e-37 2.2248049796936 0.879 0.708 8.93814007033539e-33 "PR2" "NOP10" "TI_R_P3"

"356" 3.19919439551001e-37 2.30720528247448 0.809 0.62 1.06881885559594e-32 "PR2" "ENSA" "TI_R_P3"

"357" 3.78085552422795e-37 1.34030432191418 0.679 0.436 1.26314602208932e-32 "PR2" "RNF145" "TI_R_P3"

"358" 4.50859251686302e-37 1.82510347157684 0.805 0.591 1.50627567395877e-32 "PR2" "SSU72" "TI_R_P3"

"359" 4.53107686364488e-37 1.62573105273096 0.775 0.553 1.51378746937512e-32 "PR2" "PRDX3" "TI_R_P3"

"360" 5.17790748733493e-37 1.14743423561645 0.653 0.423 1.72988711244373e-32 "PR2" "TMEM106C" "TI_R_P3"

"361" 5.31474449606475e-37 2.9236021666601 0.595 0.335 1.77560298869027e-32 "PR2" "TMPRSS4" "TI_R_P3"

"362" 5.89671057959029e-37 1.49366562998302 0.863 0.679 1.97003203753532e-32 "PR2" "PPP1CB" "TI_R_P3"

"363" 7.46757008354891e-37 1.06390873340708 0.621 0.373 2.49484048921285e-32 "PR2" "MTUS1" "TI_R_P3"

"364" 1.06292642241314e-36 1.07368448881286 0.702 0.484 3.55113088464007e-32 "PR2" "ACAA1" "TI_R_P3"

"365" 1.40322413416301e-36 2.20448693647781 0.725 0.482 4.6880315098252e-32 "PR2" "SNRPE" "TI_R_P3"

"366" 1.43108665628831e-36 2.17213672515171 0.858 0.699 4.7811174099936e-32 "PR2" "PTGES3" "TI_R_P3"

"367" 1.56632705637414e-36 2.44239786804606 0.725 0.482 5.23294206264037e-32 "PR2" "GLB1" "TI_R_P3"

"368" 1.57240103359294e-36 1.00553903565151 0.607 0.356 5.25323461313066e-32 "PR2" "THOC2" "TI_R_P3"

"369" 1.7641777966768e-36 2.52358966913956 0.64 0.397 5.89394160091753e-32 "PR2" "CMAS" "TI_R_P3"

"370" 2.02798348518445e-36 2.41555117928795 0.728 0.511 6.77529002565274e-32 "PR2" "MRPL33" "TI_R_P3"

"371" 2.25791242826519e-36 2.10829072694995 0.611 0.379 7.54345963159119e-32 "PR2" "CTNNBIP1" "TI_R_P3"

"372" 2.74117214129198e-36 1.07983635502256 0.605 0.354 9.15798200684236e-32 "PR2" "CLIC6" "TI_R_P3"

"373" 3.00008043517919e-36 1.03680852211438 0.754 0.521 1.00229687258902e-31 "PR2" "TSPAN4" "TI_R_P3"

"374" 3.08413893309181e-36 1.24151626874347 0.647 0.416 1.03037997615664e-31 "PR2" "MRPS26" "TI_R_P3"

"375" 3.40781657723075e-36 3.2041695216575 0.881 0.738 1.13851744028702e-31 "PR2" "SEC61G" "TI_R_P3"

"376" 4.46708728138407e-36 1.4399153354871 0.761 0.542 1.4924091898376e-31 "PR2" "EMP2" "TI_R_P3"

"377" 4.92738245623121e-36 1.49819253502287 0.64 0.394 1.64618920480229e-31 "PR2" "SVIP" "TI_R_P3"

"378" 5.77801950372952e-36 1.18949602608121 0.795 0.603 1.930378536001e-31 "PR2" "HSPA9" "TI_R_P3"

"379" 6.04435851470382e-36 1.46982697488044 0.839 0.652 2.0193597361774e-31 "PR2" "EIF3I" "TI_R_P3"

"380" 6.15117489191361e-36 1.12183916747165 0.881 0.725 2.05504601963942e-31 "PR2" "DYNLRB1" "TI_R_P3"

"381" 6.15182762194204e-36 1.32406041124896 0.504 0.269 2.05526409021461e-31 "PR2" "INSIG1" "TI_R_P3"

"382" 8.71885392259034e-36 2.04817364004052 0.595 0.363 2.91288190699821e-31 "PR2" "CLK1" "TI_R_P3"

"383" 8.88431424107495e-36 3.1744931449975 0.925 0.776 2.96816054480073e-31 "PR2" "KDELR2" "TI_R_P3"

"384" 9.03666982027107e-36 2.02423847592401 0.616 0.367 3.01906102025436e-31 "PR2" "SLC50A1" "TI_R_P3"

"385" 9.7432332687931e-36 1.85652248717034 0.698 0.475 3.25511680277109e-31 "PR2" "CAT" "TI_R_P3"

"386" 1.32415776804538e-35 1.31049416420921 0.489 0.246 4.42387868726282e-31 "PR2" "PODXL2" "TI_R_P3"

"387" 1.421052378492e-35 1.00074240971512 0.828 0.633 4.74759389130392e-31 "PR2" "LAMTOR1" "TI_R_P3"

"388" 1.96267876730713e-35 1.40175406536264 0.956 0.852 6.55711349369638e-31 "PR2" "CDC42" "TI_R_P3"

"389" 1.96269508474826e-35 2.23774888162859 0.849 0.659 6.55716800863546e-31 "PR2" "TMEM219" "TI_R_P3"

"390" 2.08702364420846e-35 2.0283920126881 0.716 0.496 6.97253729293603e-31 "PR2" "COX14" "TI_R_P3"

"391" 2.41330729972939e-35 1.93546770968901 0.746 0.549 8.06261835766593e-31 "PR2" "DRAM1" "TI_R_P3"

"392" 2.50679986934837e-35 1.02616568926092 0.833 0.638 8.37496768350598e-31 "PR2" "MINOS1" "TI_R_P3"

"393" 2.60759642381583e-35 1.51527481989188 0.651 0.431 8.7117188923263e-31 "PR2" "MGLL" "TI_R_P3"

"394" 2.92808364718054e-35 1.2473107221362 0.702 0.462 9.78243465686545e-31 "PR2" "ALOX15B" "TI_R_P3"

"395" 3.55838339591564e-35 1.16169606429489 0.554 0.315 1.18882030874146e-30 "PR2" "CX3CL1" "TI_R_P3"

"396" 3.76955151288993e-35 1.12369655446894 0.646 0.392 1.2593694649414e-30 "PR2" "SPAG9" "TI_R_P3"

"397" 4.33934619795474e-35 1.51496659858746 0.732 0.512 1.4497321712747e-30 "PR2" "C1orf43" "TI_R_P3"

"398" 4.78006889418337e-35 1.26960912357461 0.612 0.37 1.59697321685772e-30 "PR2" "TIMM17B" "TI_R_P3"

"399" 6.24720161469647e-35 1.45683499070343 0.635 0.397 2.08712758745394e-30 "PR2" "IVD" "TI_R_P3"

"400" 7.68804675654052e-35 2.89519211031057 0.902 0.746 2.56849954089262e-30 "PR2" "TXN" "TI_R_P3"

"401" 8.63105051716084e-35 1.48001250326029 0.702 0.469 2.88354766727826e-30 "PR2" "GGCX" "TI_R_P3"

"402" 1.00958707628739e-34 1.60661649608316 0.682 0.458 3.37292946316855e-30 "PR2" "ERBB2" "TI_R_P3"

"403" 1.21661721426844e-34 3.36385817557229 0.833 0.652 4.06459645114943e-30 "PR2" "ZFAS1" "TI_R_P3"

"404" 1.38818338632177e-34 2.38957303770203 0.667 0.45 4.6377818753624e-30 "PR2" "NDUFB3" "TI_R_P3"

"405" 1.43935836871063e-34 3.36593553495235 0.826 0.651 4.80875237402533e-30 "PR2" "NEDD8" "TI_R_P3"

"406" 1.43939103094997e-34 4.04010381979738 0.705 0.488 4.80886149530076e-30 "PR2" "SMDT1" "TI_R_P3"

"407" 1.62917256545785e-34 1.83241711836816 0.749 0.534 5.44290262393812e-30 "PR2" "CYSTM1" "TI_R_P3"

"408" 1.64792495110956e-34 1.25800136670709 0.607 0.363 5.50555246916193e-30 "PR2" "PPP2R5A" "TI_R_P3"

"409" 2.28648036453331e-34 1.97146952819256 0.775 0.553 7.63890224986933e-30 "PR2" "TM4SF1" "TI_R_P3"

"410" 2.72221343751442e-34 1.45668452364141 0.691 0.451 9.09464287339191e-30 "PR2" "CNPY2" "TI_R_P3"

"411" 3.0268724808007e-34 1.96137909040759 0.739 0.525 1.01124782711071e-29 "PR2" "SNRPN" "TI_R_P3"

"412" 3.3426573775389e-34 1.59516472158687 0.537 0.304 1.11674840326197e-29 "PR2" "CNOT8" "TI_R_P3"

"413" 3.56419994802243e-34 1.97723059869131 0.633 0.406 1.19076356063481e-29 "PR2" "NADK" "TI_R_P3"

"414" 4.43479888512185e-34 1.63499196567059 0.811 0.577 1.48162195953036e-29 "PR2" "SEPT9" "TI_R_P3"

"415" 5.46946361237575e-34 1.13690990388592 0.418 0.2 1.82729309825861e-29 "PR2" "TACC2" "TI_R_P3"

"416" 5.6925812532644e-34 1.09196337567337 0.711 0.486 1.9018344709031e-29 "PR2" "RBMX" "TI_R_P3"

"417" 6.17544788735032e-34 1.37333457598884 0.779 0.577 2.06315538468487e-29 "PR2" "CD47" "TI_R_P3"

"418" 6.20403527800179e-34 1.19589760903765 0.756 0.525 2.07270614602762e-29 "PR2" "TCEAL9" "TI_R_P3"

"419" 7.81443073673961e-34 1.51573687166517 0.679 0.442 2.61072316483734e-29 "PR2" "POLR2A" "TI_R_P3"

"420" 7.9499243697382e-34 1.75729379440035 0.489 0.259 2.65599023268584e-29 "PR2" "MAST4" "TI_R_P3"

"421" 8.808619189918e-34 1.07558045365741 0.589 0.35 2.9428715851597e-29 "PR2" "RAB25" "TI_R_P3"

"422" 9.326758412647e-34 1.38947001540762 0.782 0.593 3.11597671808124e-29 "PR2" "MRPS21" "TI_R_P3"

"423" 1.02839026123238e-33 1.40346128034723 0.579 0.342 3.43574902375125e-29 "PR2" "MGST2" "TI_R_P3"

"424" 1.18466467456334e-33 1.30217214865815 0.621 0.392 3.95784621124866e-29 "PR2" "SLC39A4" "TI_R_P3"

"425" 1.2508770130705e-33 1.15645612473911 0.588 0.349 4.17905501296724e-29 "PR2" "UGCG" "TI_R_P3"

"426" 1.26588998974536e-33 1.59728507930702 0.626 0.395 4.22921186674026e-29 "PR2" "GPRC5A" "TI_R_P3"

"427" 1.67891058730321e-33 2.03239238899242 0.825 0.617 5.60907238112129e-29 "PR2" "MTCH1" "TI_R_P3"

"428" 1.8418023430488e-33 2.51334177418748 0.639 0.406 6.15327744789173e-29 "PR2" "CARD16" "TI_R_P3"

"429" 2.09365604165146e-33 1.5917971915481 0.642 0.407 6.99469546955338e-29 "PR2" "MIEN1" "TI_R_P3"

"430" 2.38456675841214e-33 1.12681482978012 0.639 0.41 7.96659908317913e-29 "PR2" "HMGB2" "TI_R_P3"

"431" 2.41817224520045e-33 1.08402168807015 0.511 0.283 8.07887165399019e-29 "PR2" "ANXA3" "TI_R_P3"

"432" 2.67618137139719e-33 1.47983261738981 0.832 0.65 8.94085434370086e-29 "PR2" "EIF4G1" "TI_R_P3"

"433" 2.8959542830504e-33 1.53587544064928 0.735 0.513 9.67509366424307e-29 "PR2" "MANF" "TI_R_P3"

"434" 2.95832461451568e-33 2.95827356080548 0.737 0.521 9.88346670463544e-29 "PR2" "TIMM8B" "TI_R_P3"

"435" 3.02245230750567e-33 1.03747059366162 0.574 0.346 1.00977109141457e-28 "PR2" "KLHDC2" "TI_R_P3"

"436" 3.50789811684148e-33 1.09796391080568 0.788 0.592 1.17195368185557e-28 "PR2" "RHEB" "TI_R_P3"

"437" 3.70293453712063e-33 1.77522815505887 0.863 0.715 1.23711339950663e-28 "PR2" "EIF4A1" "TI_R_P3"

"438" 4.06000106244222e-33 1.18225144189884 0.695 0.472 1.35640575495132e-28 "PR2" "DHRS7" "TI_R_P3"

"439" 4.17345629968792e-33 5.50527825704585 0.861 0.671 1.39431001516274e-28 "PR2" "C4orf3" "TI_R_P3"

"440" 4.47595266453246e-33 1.89787331417308 0.707 0.475 1.49537102569365e-28 "PR2" "SMS" "TI_R_P3"

"441" 4.89890617809855e-33 2.79311461154565 0.793 0.601 1.63667556504095e-28 "PR2" "SLIRP" "TI_R_P3"

"442" 6.92955760063376e-33 1.70935774060301 0.675 0.445 2.31509589879573e-28 "PR2" "METRN" "TI_R_P3"

"443" 7.10756396413172e-33 1.21806296774296 0.828 0.653 2.37456604477677e-28 "PR2" "VPS28" "TI_R_P3"

"444" 8.18130470326438e-33 1.12099004085252 0.554 0.313 2.7332920883136e-28 "PR2" "AK1" "TI_R_P3"

"445" 8.52525716147192e-33 1.3304940773681 0.689 0.458 2.84820316507615e-28 "PR2" "PABPC4" "TI_R_P3"

"446" 8.86985883875707e-33 1.34794342035016 0.619 0.406 2.96333113944035e-28 "PR2" "ARRDC3" "TI_R_P3"

"447" 9.22660322929584e-33 1.51976699262861 0.851 0.682 3.08251587287545e-28 "PR2" "NENF" "TI_R_P3"

"448" 9.23571282944824e-33 2.19094206644923 0.726 0.518 3.08555929919036e-28 "PR2" "ENY2" "TI_R_P3"

"449" 1.0892289858107e-32 2.09054555458008 0.93 0.815 3.63900511869497e-28 "PR2" "PLTP" "TI_R_P3"

"450" 1.11905690697771e-32 1.51533478552967 0.702 0.491 3.73865722052183e-28 "PR2" "MDH2" "TI_R_P3"

"451" 1.22283146470144e-32 2.10559928328742 0.575 0.348 4.08535764042104e-28 "PR2" "BEX4" "TI_R_P3"

"452" 1.47956518021779e-32 1.0252437294643 0.577 0.338 4.94307931058961e-28 "PR2" "YIPF6" "TI_R_P3"

"453" 1.9637758609036e-32 1.37250029237571 0.854 0.669 6.56077877369285e-28 "PR2" "SCARB2" "TI_R_P3"

"454" 2.19298201445277e-32 2.05245911833769 0.802 0.623 7.32653361208526e-28 "PR2" "LMAN2" "TI_R_P3"

"455" 2.25443970928737e-32 1.3154023733692 0.751 0.523 7.53185762475819e-28 "PR2" "SELENOK" "TI_R_P3"

"456" 2.48434026204282e-32 1.13161027419912 0.696 0.471 8.29993238145885e-28 "PR2" "NCKAP1" "TI_R_P3"

"457" 2.59878045808705e-32 1.91276883400529 0.612 0.38 8.68226563242302e-28 "PR2" "CCNG2" "TI_R_P3"

"458" 2.69509888610448e-32 1.53708536135202 0.6 0.367 9.00405586858647e-28 "PR2" "DLG3" "TI_R_P3"

"459" 2.86997324663583e-32 1.89561402217075 0.6 0.378 9.58829361968565e-28 "PR2" "GALNT10" "TI_R_P3"

"460" 2.94762226656094e-32 1.1597440533158 0.775 0.6 9.84771123035346e-28 "PR2" "EIF3H" "TI_R_P3"

"461" 3.01800576969892e-32 1.27843740038038 0.649 0.435 1.00828554759871e-27 "PR2" "RETREG2" "TI_R_P3"

"462" 3.07930986225818e-32 1.75229813567167 0.782 0.584 1.02876663188184e-27 "PR2" "CNDP2" "TI_R_P3"

"463" 3.25254587812243e-32 1.8736237925142 0.746 0.544 1.08664305242192e-27 "PR2" "IDH2" "TI_R_P3"

"464" 3.52661167866189e-32 1.18120851533953 0.728 0.519 1.17820569572415e-27 "PR2" "KRT10" "TI_R_P3"

"465" 3.92816296782598e-32 1.54617519988482 0.551 0.326 1.31235996592098e-27 "PR2" "ARMCX6" "TI_R_P3"

"466" 4.0759311717365e-32 1.25496182701431 0.495 0.27 1.36172784516545e-27 "PR2" "LRBA" "TI_R_P3"

"467" 4.31188944026668e-32 1.29550669621541 0.882 0.692 1.44055914309869e-27 "PR2" "MRFAP1" "TI_R_P3"

"468" 4.54335265527004e-32 1.06627516260633 0.581 0.357 1.51788868859917e-27 "PR2" "FDX1" "TI_R_P3"

"469" 4.58670394001332e-32 1.27192977700293 0.591 0.355 1.53237191931905e-27 "PR2" "TMC5" "TI_R_P3"

"470" 5.46252849701465e-32 1.70111250468101 0.725 0.505 1.82497614556762e-27 "PR2" "PSMB2" "TI_R_P3"

"471" 6.47015271094411e-32 1.0464099832974 0.412 0.198 2.16161331919932e-27 "PR2" "FAXDC2" "TI_R_P3"

"472" 8.67638292544574e-32 1.37585858327559 0.619 0.395 2.89869277156217e-27 "PR2" "MEAF6" "TI_R_P3"

"473" 9.16486415605295e-32 1.06602789744498 0.502 0.277 3.06188946589573e-27 "PR2" "RAB17" "TI_R_P3"

"474" 1.03133836295906e-31 1.31009972173504 0.633 0.401 3.44559833680992e-27 "PR2" "ZBED1" "TI_R_P3"

"475" 1.1356430183868e-31 2.24344440502181 0.7 0.48 3.79406976012846e-27 "PR2" "EFCAB14" "TI_R_P3"

"476" 1.32684000138874e-31 1.03943903407507 0.805 0.607 4.43283976063966e-27 "PR2" "NDUFV2" "TI_R_P3"

"477" 1.47561924982391e-31 1.00350739105866 0.681 0.44 4.9298963517367e-27 "PR2" "EIF2S3" "TI_R_P3"

"478" 1.61651059592029e-31 1.59696957388208 0.649 0.426 5.40060024991009e-27 "PR2" "DUT" "TI_R_P3"

"479" 1.82989283491018e-31 2.15273682658067 0.807 0.644 6.11348897215142e-27 "PR2" "PSMA7" "TI_R_P3"

"480" 2.24290561923845e-31 1.16350292555694 0.572 0.347 7.49332338331373e-27 "PR2" "FAM3A" "TI_R_P3"

"481" 2.33392764269155e-31 1.72784191930939 0.832 0.649 7.79741886146821e-27 "PR2" "PRKAR1A" "TI_R_P3"

"482" 2.52990523697353e-31 1.54138299620564 0.518 0.294 8.45216040620488e-27 "PR2" "FXYD3" "TI_R_P3"

"483" 2.88216468342642e-31 1.14815461786839 0.798 0.622 9.62902399085934e-27 "PR2" "SERP1" "TI_R_P3"

"484" 3.13008636415225e-31 1.83402730679961 0.704 0.494 1.04573055339962e-26 "PR2" "UBE2M" "TI_R_P3"

"485" 3.56602228172858e-31 2.82466314389748 0.777 0.597 1.1913723841027e-26 "PR2" "ERP29" "TI_R_P3"

"486" 3.66258079476136e-31 2.29405745987302 0.751 0.552 1.22363161772182e-26 "PR2" "SEC11A" "TI_R_P3"

"487" 4.03589870247612e-31 1.65120005118484 0.647 0.436 1.34835339751025e-26 "PR2" "FUNDC2" "TI_R_P3"

"488" 4.44041744774636e-31 2.02159987204989 0.609 0.395 1.48349906511758e-26 "PR2" "DCTD" "TI_R_P3"

"489" 4.47980976082775e-31 1.93811012187168 0.642 0.409 1.49665964299494e-26 "PR2" "RBM47" "TI_R_P3"

"490" 4.5930557527692e-31 1.48046570461619 0.679 0.449 1.53449399644266e-26 "PR2" "PUM2" "TI_R_P3"

"491" 4.62525982485645e-31 1.16685448477733 0.947 0.825 1.54525305488629e-26 "PR2" "ARPC1B" "TI_R_P3"

"492" 4.98946528629931e-31 1.30722207845831 0.802 0.61 1.66693045749974e-26 "PR2" "ATP5PD" "TI_R_P3"

"493" 5.01905197745127e-31 1.11789013388757 0.489 0.266 1.6768150751467e-26 "PR2" "LBR" "TI_R_P3"

"494" 5.74129687503596e-31 1.37654705855905 0.665 0.449 1.91810987298076e-26 "PR2" "FURIN" "TI_R_P3"

"495" 6.01348717108225e-31 1.59478333823844 0.811 0.642 2.00904592898687e-26 "PR2" "IQGAP1" "TI_R_P3"

"496" 8.35789094985576e-31 1.73317974266422 0.649 0.418 2.79228778743731e-26 "PR2" "IMPDH2" "TI_R_P3"

"497" 8.76830779858282e-31 1.45021451534625 0.74 0.564 2.92940395242854e-26 "PR2" "PPCS" "TI_R_P3"

"498" 1.02237870593069e-30 1.46194410820289 0.63 0.4 3.41566501864384e-26 "PR2" "CENPX" "TI_R_P3"

"499" 1.39705529862761e-30 1.0442100048037 0.872 0.741 4.66742204718498e-26 "PR2" "ARF4" "TI_R_P3"

"500" 1.79516609716187e-30 1.19756986073949 0.509 0.289 5.9974704140081e-26 "PR2" "ZDHHC16" "TI_R_P3"

"501" 2.35497705706556e-30 1.08110431191159 0.761 0.565 7.86774284995033e-26 "PR2" "HNRNPD" "TI_R_P3"

"502" 2.536594787237e-30 1.19256145538133 0.511 0.295 8.47450952468008e-26 "PR2" "IDI1" "TI_R_P3"

"503" 2.84003497182234e-30 1.65779073733537 0.688 0.491 9.48827283736125e-26 "PR2" "EI24" "TI_R_P3"

"504" 3.72126883994484e-30 2.79786814998646 0.702 0.47 1.24323870673717e-25 "PR2" "FAM3C" "TI_R_P3"

"505" 4.93916252504003e-30 2.22424991037101 0.725 0.51 1.65012480799062e-25 "PR2" "CHCHD1" "TI_R_P3"

"506" 5.0600554708031e-30 1.08608295774707 0.519 0.293 1.69051393224061e-25 "PR2" "FAM136A" "TI_R_P3"

"507" 5.29162018009043e-30 1.51430669107393 0.511 0.291 1.76787738596641e-25 "PR2" "TLR2" "TI_R_P3"

"508" 6.31590637268658e-30 1.08565940458669 0.586 0.37 2.11008116005086e-25 "PR2" "TBC1D15" "TI_R_P3"

"509" 6.34605095606991e-30 1.14481795306189 0.611 0.386 2.1201521639134e-25 "PR2" "RRM2B" "TI_R_P3"

"510" 1.13477783670264e-29 1.13347645848526 0.533 0.307 3.79117927463986e-25 "PR2" "SREBF2" "TI_R_P3"

"511" 1.17851327330984e-29 1.29452400963627 0.658 0.453 3.93729499480085e-25 "PR2" "SNRPC" "TI_R_P3"

"512" 1.19008154604127e-29 1.30825001642751 0.586 0.366 3.97594343716928e-25 "PR2" "ADK" "TI_R_P3"

"513" 1.32343074616922e-29 1.17434219107963 0.625 0.409 4.42144977987676e-25 "PR2" "POLR2G" "TI_R_P3"

"514" 1.3922982992759e-29 1.50747176464198 0.598 0.363 4.65152938805085e-25 "PR2" "ZMIZ2" "TI_R_P3"

"515" 1.51632135918089e-29 1.01415279321038 0.714 0.508 5.06587802888744e-25 "PR2" "ADH5" "TI_R_P3"

"516" 1.67514154660714e-29 1.58194422958895 0.647 0.43 5.59648039305979e-25 "PR2" "AAMP" "TI_R_P3"

"517" 1.71895598974543e-29 1.0273323526824 0.612 0.403 5.74286006614049e-25 "PR2" "RBBP4" "TI_R_P3"

"518" 2.04401520223043e-29 2.30426324623994 0.842 0.706 6.82885038913164e-25 "PR2" "C2" "TI_R_P3"

"519" 2.09010054899626e-29 1.3097144824751 0.782 0.591 6.9828169241416e-25 "PR2" "PSMB8" "TI_R_P3"

"520" 2.96609780006723e-29 1.59632196003411 0.726 0.527 9.90943614024461e-25 "PR2" "SF1" "TI_R_P3"

"521" 3.120795363128e-29 1.32355198778476 0.688 0.465 1.04262652286743e-24 "PR2" "SPCS2" "TI_R_P3"

"522" 3.30179809574713e-29 1.05108838267274 0.581 0.361 1.10309772580816e-24 "PR2" "DNPH1" "TI_R_P3"

"523" 3.88627246788846e-29 2.02258315237556 0.686 0.485 1.29836476879686e-24 "PR2" "NDUFAB1" "TI_R_P3"

"524" 4.61836895809242e-29 1.35830907537285 0.642 0.447 1.5429508852091e-24 "PR2" "LAMB3" "TI_R_P3"

"525" 5.07509389762718e-29 1.02410188560971 0.46 0.249 1.69553812025827e-24 "PR2" "DHCR7" "TI_R_P3"

"526" 8.38098475075202e-29 3.19099591235522 0.691 0.474 2.80000319537874e-24 "PR2" "COMMD4" "TI_R_P3"

"527" 1.09277653036221e-28 4.62450468847789 0.782 0.58 3.65085711028711e-24 "PR2" "DDIT4" "TI_R_P3"

"528" 1.12569246198894e-28 4.12480676498031 0.791 0.626 3.76082594625886e-24 "PR2" "PRR13" "TI_R_P3"

"529" 1.24134931926587e-28 1.37252296513159 0.496 0.284 4.14722394073533e-24 "PR2" "DLGAP1-AS1" "TI_R_P3"

"530" 1.24796902173864e-28 2.94102864206351 0.674 0.478 4.16933970472661e-24 "PR2" "TCEAL4" "TI_R_P3"

"531" 2.75931441566434e-28 1.26803274271963 0.753 0.562 9.21859353129299e-24 "PR2" "PSMA1" "TI_R_P3"

"532" 2.91235774018836e-28 1.26982799741066 0.791 0.619 9.72989597419529e-24 "PR2" "ARL6IP1" "TI_R_P3"

"533" 3.39316357604809e-28 1.27697848253089 0.784 0.614 1.13362201912191e-23 "PR2" "H2AFY" "TI_R_P3"

"534" 3.67407630838459e-28 1.35052072755323 0.689 0.511 1.22747215386821e-23 "PR2" "SEZ6L2" "TI_R_P3"

"535" 3.9163990037783e-28 1.25315649789628 0.705 0.507 1.30842974317229e-23 "PR2" "NME3" "TI_R_P3"

"536" 4.11781435795051e-28 6.41035359606331 0.902 0.761 1.37572059884769e-23 "PR2" "MT1E" "TI_R_P3"

"537" 4.15824180171191e-28 1.0232168699269 0.688 0.479 1.38922700353393e-23 "PR2" "ELOVL1" "TI_R_P3"

"538" 5.63741891887936e-28 1.3776931165603 0.616 0.399 1.8834052866084e-23 "PR2" "STAG2" "TI_R_P3"

"539" 6.21745670542556e-28 1.0927596929397 0.558 0.341 2.07719011071563e-23 "PR2" "ACTR1B" "TI_R_P3"

"540" 7.21178179545809e-28 1.99995400402088 0.625 0.413 2.40938418004459e-23 "PR2" "EIF2AK1" "TI_R_P3"

"541" 7.34633044112503e-28 1.11553060997522 0.784 0.618 2.45433553707546e-23 "PR2" "ATRAID" "TI_R_P3"

"542" 8.13952627521021e-28 1.32626972577614 0.567 0.353 2.71933433328498e-23 "PR2" "QPCT" "TI_R_P3"

"543" 8.50679141862367e-28 1.36168422549121 0.649 0.434 2.84203394504798e-23 "PR2" "G3BP2" "TI_R_P3"

"544" 8.89190299517879e-28 1.05291386720146 0.496 0.286 2.97069587165928e-23 "PR2" "C19orf33" "TI_R_P3"

"545" 9.45160709504754e-28 1.31757019590763 0.586 0.373 3.15768741438443e-23 "PR2" "GPCPD1" "TI_R_P3"

"546" 9.6960944333448e-28 1.01556090108586 0.444 0.241 3.23936818923617e-23 "PR2" "TMEM164" "TI_R_P3"

"547" 1.00444106382606e-27 1.49169500542163 0.563 0.343 3.35573715013647e-23 "PR2" "HES1" "TI_R_P3"

"548" 1.14171876846799e-27 1.67516945538707 0.582 0.377 3.81436823357471e-23 "PR2" "MCTS1" "TI_R_P3"

"549" 1.41003527554603e-27 1.39860556635357 0.507 0.296 4.71078685207174e-23 "PR2" "ARHGEF2" "TI_R_P3"

"550" 1.41182208352329e-27 1.17310460340422 0.751 0.576 4.71675639884296e-23 "PR2" "PSMA4" "TI_R_P3"

"551" 1.51757009396109e-27 3.15507748761445 0.663 0.457 5.07004992691462e-23 "PR2" "TSC22D3" "TI_R_P3"

"552" 1.56342239821394e-27 4.44730121066391 0.714 0.532 5.22323789019296e-23 "PR2" "MACF1" "TI_R_P3"

"553" 1.57295491080824e-27 1.00470827024149 0.637 0.435 5.25508506151924e-23 "PR2" "RNPEPL1" "TI_R_P3"

"554" 1.80598296636892e-27 1.11936825583752 0.705 0.521 6.03360849234191e-23 "PR2" "ITGA3" "TI_R_P3"

"555" 2.41945516173078e-27 2.24131982152552 0.772 0.581 8.08315774982638e-23 "PR2" "SRSF3" "TI_R_P3"

"556" 2.66216325470672e-27 3.78903287200062 0.867 0.741 8.89402121764969e-23 "PR2" "ATP6V0B" "TI_R_P3"

"557" 3.71728101742455e-27 3.03635076726488 0.816 0.632 1.24190641511137e-22 "PR2" "ATP5F1A" "TI_R_P3"

"558" 4.52194683521211e-27 3.42841930884011 0.619 0.412 1.51073721817602e-22 "PR2" "TMEM134" "TI_R_P3"

"559" 4.65421805634039e-27 1.17722107147715 0.377 0.191 1.55492771044276e-22 "PR2" "ST3GAL5" "TI_R_P3"

"560" 6.28744691578349e-27 1.11465536490779 0.591 0.381 2.10057314009411e-22 "PR2" "SELENOS" "TI_R_P3"

"561" 6.50478062619166e-27 1.14743041861132 0.646 0.438 2.17318215940437e-22 "PR2" "GGA2" "TI_R_P3"

"562" 7.43298019462545e-27 1.16642039109188 0.411 0.216 2.48328435322242e-22 "PR2" "HLF" "TI_R_P3"

"563" 7.5365660336114e-27 1.21084365184322 0.605 0.395 2.51789134616923e-22 "PR2" "WWC3" "TI_R_P3"

"564" 7.87574176189319e-27 1.79411446570961 0.749 0.552 2.6312065652309e-22 "PR2" "SFPQ" "TI_R_P3"

"565" 7.96105720427643e-27 2.01786091144615 0.774 0.591 2.65970960137671e-22 "PR2" "SLC38A10" "TI_R_P3"

"566" 8.31340338487392e-27 1.40606737755122 0.604 0.381 2.77742493685253e-22 "PR2" "PTPMT1" "TI_R_P3"

"567" 8.36115917685116e-27 1.06749187070476 0.761 0.575 2.7933796693942e-22 "PR2" "FUS" "TI_R_P3"

"568" 8.52572990452926e-27 1.2402269443422 0.714 0.524 2.84836110380418e-22 "PR2" "CYCS" "TI_R_P3"

"569" 8.83227907407614e-27 1.01714725535988 0.489 0.28 2.9507761158581e-22 "PR2" "WWC1" "TI_R_P3"

"570" 9.98060278433292e-27 3.47815093220324 0.598 0.402 3.33441958421778e-22 "PR2" "TFRC" "TI_R_P3"

"571" 9.99651862665577e-27 1.26216247452237 0.891 0.753 3.33973690797943e-22 "PR2" "ARPC2" "TI_R_P3"

"572" 1.100429082684e-26 1.12408493808022 0.539 0.331 3.67642352233897e-22 "PR2" "ALDOC" "TI_R_P3"

"573" 1.15986320624724e-26 1.09451529207866 0.696 0.486 3.8749869857514e-22 "PR2" "PHB" "TI_R_P3"

"574" 1.1610773214848e-26 1.15962923197961 0.754 0.565 3.87904322334858e-22 "PR2" "SLC25A39" "TI_R_P3"

"575" 1.2857966712785e-26 2.35043332691723 0.768 0.592 4.29571809907433e-22 "PR2" "FLOT1" "TI_R_P3"

"576" 1.39329073193647e-26 1.33411853556589 0.684 0.467 4.65484500632656e-22 "PR2" "SMIM37" "TI_R_P3"

"577" 1.3947743257681e-26 1.84594333489059 0.739 0.564 4.65980154495866e-22 "PR2" "SRSF2" "TI_R_P3"

"578" 1.79283037267783e-26 1.04028087869892 0.572 0.369 5.98966699207935e-22 "PR2" "ACSL1" "TI_R_P3"

"579" 1.84095568372038e-26 1.58114964278801 0.444 0.245 6.15044884374142e-22 "PR2" "SHISA2" "TI_R_P3"

"580" 2.20054783681349e-26 3.90827991033141 0.772 0.6 7.3518102680102e-22 "PR2" "EIF3F" "TI_R_P3"

"581" 2.27162756395945e-26 1.40315117243102 0.732 0.562 7.58928052843213e-22 "PR2" "TMEM14C" "TI_R_P3"

"582" 3.01509502880253e-26 1.06987426383552 0.561 0.356 1.00731309817264e-21 "PR2" "NDNF" "TI_R_P3"

"583" 3.28399658710463e-26 1.58394136959223 0.474 0.272 1.09715041978579e-21 "PR2" "NQO1" "TI_R_P3"

"584" 3.57992100225531e-26 1.38491362788847 0.54 0.334 1.19601580764348e-21 "PR2" "IMP3" "TI_R_P3"

"585" 3.70164502046941e-26 1.00223342134658 0.675 0.458 1.23668258488862e-21 "PR2" "STARD7" "TI_R_P3"

"586" 3.80078798528958e-26 3.30337707747267 0.889 0.74 1.26980525800539e-21 "PR2" "CD44" "TI_R_P3"

"587" 3.95170283463164e-26 1.23759205580254 0.661 0.472 1.32022440002208e-21 "PR2" "PTP4A1" "TI_R_P3"

"588" 4.19337537571573e-26 1.76738077880596 0.739 0.56 1.40096477927287e-21 "PR2" "RPN1" "TI_R_P3"

"589" 4.7745683076272e-26 1.06316546808084 0.53 0.318 1.59513552589517e-21 "PR2" "FBXW4" "TI_R_P3"

"590" 5.5404493138096e-26 1.49424353706157 0.604 0.398 1.85100871125065e-21 "PR2" "WDR6" "TI_R_P3"

"591" 5.5562061136937e-26 1.67700357913148 0.577 0.37 1.85627290052393e-21 "PR2" "UQCRC2" "TI_R_P3"

"592" 5.5749162906498e-26 1.73397848067685 0.795 0.632 1.86252378354319e-21 "PR2" "HSBP1" "TI_R_P3"

"593" 9.09696579151534e-26 1.97579702340031 0.807 0.65 3.03920530128736e-21 "PR2" "GPX3" "TI_R_P3"

"594" 1.09418623624464e-25 1.22100743831988 0.675 0.459 3.6555667966697e-21 "PR2" "PCNP" "TI_R_P3"

"595" 1.11658240283066e-25 1.51783722094976 0.854 0.73 3.73039014961696e-21 "PR2" "SSR2" "TI_R_P3"

"596" 1.29322822412382e-25 1.27735382040459 0.707 0.509 4.32054617397527e-21 "PR2" "RAB5B" "TI_R_P3"

"597" 1.32177358624589e-25 1.65551692922748 0.856 0.707 4.41591337428888e-21 "PR2" "UBE2D3" "TI_R_P3"

"598" 1.48896149142627e-25 1.47834202972161 0.632 0.438 4.97447144670603e-21 "PR2" "AK2" "TI_R_P3"

"599" 1.56657572070791e-25 1.1417770770919 0.754 0.567 5.23377282531306e-21 "PR2" "BAG6" "TI_R_P3"

"600" 1.59828345077859e-25 1.2003206760772 0.809 0.638 5.33970518070619e-21 "PR2" "CRTAP" "TI_R_P3"

"601" 2.10624034999619e-25 1.1519117279595 0.382 0.197 7.03673838530227e-21 "PR2" "SCEL" "TI_R_P3"

"602" 2.22142518032269e-25 1.76078936828941 0.742 0.551 7.42155938494007e-21 "PR2" "XRCC5" "TI_R_P3"

"603" 2.70166184064562e-25 1.23961187261604 0.684 0.493 9.02598204341295e-21 "PR2" "SPG21" "TI_R_P3"

"604" 2.8852175379543e-25 1.07678716189974 0.381 0.191 9.63922327255151e-21 "PR2" "YEATS4" "TI_R_P3"

"605" 3.06765343616418e-25 1.23961341894499 0.639 0.445 1.02487233648809e-20 "PR2" "TADA3" "TI_R_P3"

"606" 3.21720731621352e-25 1.07526012947935 0.712 0.514 1.07483679227377e-20 "PR2" "DDX3X" "TI_R_P3"

"607" 3.8658314548817e-25 1.42557767712311 0.523 0.32 1.29153563076143e-20 "PR2" "IRX3" "TI_R_P3"

"608" 4.01040926873181e-25 1.07890238423537 0.747 0.557 1.33983763259061e-20 "PR2" "SON" "TI_R_P3"

"609" 4.2413007406331e-25 1.03825118828313 0.57 0.353 1.41697616443811e-20 "PR2" "DHX30" "TI_R_P3"

"610" 4.72254967349241e-25 1.13978605342979 0.768 0.602 1.57775662041708e-20 "PR2" "ARF3" "TI_R_P3"

"611" 4.91611218164613e-25 1.1546443009193 0.651 0.449 1.64242391876615e-20 "PR2" "DCXR" "TI_R_P3"

"612" 1.05012362086308e-24 1.16296830743675 0.635 0.446 3.50835800494147e-20 "PR2" "MAGT1" "TI_R_P3"

"613" 1.16802636651878e-24 1.6427233101237 0.791 0.626 3.90225928790258e-20 "PR2" "RNH1" "TI_R_P3"

"614" 1.18484448820658e-24 1.51542635263955 0.544 0.338 3.95844695064937e-20 "PR2" "SULT1A1" "TI_R_P3"

"615" 1.26338277658797e-24 1.68907303508492 0.647 0.455 4.22083551830276e-20 "PR2" "STT3B" "TI_R_P3"

"616" 1.30046429373038e-24 1.20985390636314 0.733 0.544 4.34472115892383e-20 "PR2" "NUMA1" "TI_R_P3"

"617" 1.64621366219153e-24 1.22240233279548 0.704 0.504 5.4998352240157e-20 "PR2" "UBE2I" "TI_R_P3"

"618" 1.66770142397865e-24 1.08911856056398 0.567 0.383 5.57162368737028e-20 "PR2" "ARGLU1" "TI_R_P3"

"619" 2.00750701926833e-24 1.6162229499488 0.667 0.493 6.70688020067356e-20 "PR2" "ISCU" "TI_R_P3"

"620" 2.29032121045043e-24 1.61375849251941 0.679 0.48 7.65173413199385e-20 "PR2" "PDAP1" "TI_R_P3"

"621" 2.51386963949385e-24 1.82239935845829 0.486 0.291 8.39858707858499e-20 "PR2" "CP" "TI_R_P3"

"622" 2.59521628360935e-24 1.09721545777993 0.756 0.563 8.67035808191047e-20 "PR2" "RAB1B" "TI_R_P3"

"623" 2.68846376999265e-24 1.20053114051392 0.718 0.516 8.98188860916846e-20 "PR2" "RNPS1" "TI_R_P3"

"624" 3.11130368952515e-24 1.45930868982591 0.742 0.551 1.03945544963346e-19 "PR2" "RAB34" "TI_R_P3"

"625" 3.62862478501649e-24 1.28924548797689 0.705 0.525 1.21228725442616e-19 "PR2" "HNRNPAB" "TI_R_P3"

"626" 3.87324708696285e-24 1.37174702552496 0.712 0.524 1.29401311928342e-19 "PR2" "ATOX1" "TI_R_P3"

"627" 3.98195082145348e-24 1.39192909883465 0.537 0.336 1.33032994993939e-19 "PR2" "CASK" "TI_R_P3"

"628" 4.2492335617957e-24 1.45547619322064 0.651 0.468 1.41962644066033e-19 "PR2" "SF3B2" "TI_R_P3"

"629" 4.43346168291244e-24 1.46384475217729 0.574 0.381 1.48117521364422e-19 "PR2" "DDAH1" "TI_R_P3"

"630" 4.91895650556597e-24 1.33808130212134 0.744 0.553 1.64337417894453e-19 "PR2" "PSMD2" "TI_R_P3"

"631" 6.75128664299171e-24 1.8013916771663 0.626 0.423 2.2555373545571e-19 "PR2" "PCNA" "TI_R_P3"

"632" 7.38736627462827e-24 2.27403068565444 0.818 0.663 2.46804519869056e-19 "PR2" "SNRPD2" "TI_R_P3"

"633" 7.56409683768532e-24 1.53462093248266 0.684 0.483 2.52708911250229e-19 "PR2" "FAM234A" "TI_R_P3"

"634" 8.26604777090287e-24 1.42100848866497 0.526 0.315 2.76160389978094e-19 "PR2" "SRA1" "TI_R_P3"

"635" 1.00249172941867e-23 1.36788246560438 0.568 0.368 3.34922461881484e-19 "PR2" "MED21" "TI_R_P3"

"636" 1.04546160774986e-23 1.25630164715622 0.695 0.5 3.4927826853315e-19 "PR2" "MZT2A" "TI_R_P3"

"637" 1.41448508052158e-23 1.35813877044971 0.577 0.37 4.72565320551456e-19 "PR2" "LSM5" "TI_R_P3"

"638" 1.41496480663867e-23 2.40969775417003 0.607 0.415 4.72725592249915e-19 "PR2" "MEA1" "TI_R_P3"

"639" 1.61982127499645e-23 1.97751152519564 0.788 0.613 5.41166089763564e-19 "PR2" "IFNGR2" "TI_R_P3"

"640" 1.99902846207236e-23 1.4851033560855 0.411 0.219 6.67855418893753e-19 "PR2" "MPP7" "TI_R_P3"

"641" 2.18368477595683e-23 1.00901881126505 0.544 0.351 7.29547246799416e-19 "PR2" "RNF167" "TI_R_P3"

"642" 2.44990858825154e-23 1.18754635558862 0.507 0.315 8.18489960248956e-19 "PR2" "NASP" "TI_R_P3"

"643" 2.76847139518871e-23 1.31093173823343 0.582 0.386 9.24918608418595e-19 "PR2" "ALDH3A2" "TI_R_P3"

"644" 2.97908089746837e-23 1.34594258215343 0.695 0.515 9.95281137035209e-19 "PR2" "TM9SF2" "TI_R_P3"

"645" 2.98748835201245e-23 1.03688716426757 0.584 0.381 9.9808998352384e-19 "PR2" "SUCLG2" "TI_R_P3"

"646" 3.27350368050268e-23 1.03943160762641 0.726 0.587 1.09364484461914e-18 "PR2" "EIF4B" "TI_R_P3"

"647" 4.300349099372e-23 1.01432533434718 0.625 0.432 1.43670363060919e-18 "PR2" "ARPC1A" "TI_R_P3"

"648" 4.74640028499867e-23 1.27510096944456 0.64 0.452 1.58572487121521e-18 "PR2" "PNKD" "TI_R_P3"

"649" 5.20809618045445e-23 1.68332172695009 0.767 0.598 1.73997285292803e-18 "PR2" "PPP4C" "TI_R_P3"

"650" 5.5468586382231e-23 1.09142833150178 0.544 0.36 1.85315000244396e-18 "PR2" "PDXDC1" "TI_R_P3"

"651" 6.67164850073775e-23 1.71514368685595 0.789 0.668 2.22893104761147e-18 "PR2" "H1FX" "TI_R_P3"

"652" 6.92773408140673e-23 1.69932612381355 0.646 0.456 2.31448667925717e-18 "PR2" "CDK16" "TI_R_P3"

"653" 8.40007500338135e-23 1.11014938185541 0.519 0.328 2.80638105787967e-18 "PR2" "NT5C2" "TI_R_P3"

"654" 8.44146921433797e-23 1.01419345654558 0.586 0.396 2.82021044981817e-18 "PR2" "NARF" "TI_R_P3"

"655" 8.57538409063304e-23 1.12442165589187 0.525 0.336 2.86495007083959e-18 "PR2" "NT5C" "TI_R_P3"

"656" 1.03104062798681e-22 1.09397974551549 0.33 0.16 3.44460363404114e-18 "PR2" "P3H2" "TI_R_P3"

"657" 1.05279879630721e-22 2.08728381208417 0.474 0.281 3.51729549858276e-18 "PR2" "PHYH" "TI_R_P3"

"658" 1.11554797301188e-22 1.87297473542924 0.804 0.661 3.72693422303538e-18 "PR2" "PPT1" "TI_R_P3"

"659" 1.47284302701258e-22 1.7124259870825 0.83 0.665 4.92062126894633e-18 "PR2" "GSTO1" "TI_R_P3"

"660" 1.52742087693154e-22 1.23653711821018 0.681 0.503 5.10296040774057e-18 "PR2" "EIF6" "TI_R_P3"

"661" 1.60891027513827e-22 1.29860972495018 0.651 0.475 5.37520833820945e-18 "PR2" "CASC4" "TI_R_P3"

"662" 1.66855317540836e-22 1.27154026551491 0.572 0.386 5.57446930372179e-18 "PR2" "PAIP2" "TI_R_P3"

"663" 1.69093084418189e-22 1.87852846863983 0.651 0.465 5.64923085732727e-18 "PR2" "NHP2" "TI_R_P3"

"664" 2.12036568525504e-22 3.45077502266233 0.789 0.639 7.08392971786858e-18 "PR2" "ATP6V1F" "TI_R_P3"

"665" 3.11573521527087e-22 1.13819955121788 0.537 0.344 1.04093597806985e-17 "PR2" "MRPL40" "TI_R_P3"

"666" 3.32614208312725e-22 1.43122544124761 0.456 0.263 1.11123080855198e-17 "PR2" "HDAC6" "TI_R_P3"

"667" 3.41229706984565e-22 1.06193088078092 0.374 0.198 1.14001432806473e-17 "PR2" "SLC6A14" "TI_R_P3"

"668" 5.00638334452503e-22 1.20296047418206 0.605 0.41 1.67258261157237e-17 "PR2" "CREB3L2" "TI_R_P3"

"669" 5.0082715674399e-22 1.07187406438679 0.77 0.608 1.67321344796599e-17 "PR2" "APP" "TI_R_P3"

"670" 5.61783373206567e-22 1.546604332954 0.872 0.739 1.87686207154582e-17 "PR2" "COX6B1" "TI_R_P3"

"671" 5.70356449683794e-22 1.33904966477999 0.54 0.343 1.90550386274859e-17 "PR2" "RBM5" "TI_R_P3"

"672" 7.97144082456321e-22 3.15942754109926 0.732 0.579 2.66317866507832e-17 "PR2" "AKR1A1" "TI_R_P3"

"673" 9.47496606614268e-22 1.24998918505099 0.605 0.406 3.16549141303761e-17 "PR2" "H1F0" "TI_R_P3"

"674" 9.52508085090364e-22 1.93248845568254 0.84 0.686 3.1822342614784e-17 "PR2" "TUBA1C" "TI_R_P3"

"675" 1.20456540024703e-21 1.18437638409519 0.686 0.489 4.02433254568529e-17 "PR2" "DDB1" "TI_R_P3"

"676" 1.30226411337864e-21 1.09660954257452 0.551 0.355 4.3507341763867e-17 "PR2" "ERBIN" "TI_R_P3"

"677" 1.39667831564232e-21 1.44209451969454 0.821 0.664 4.66616258472944e-17 "PR2" "NDUFA13" "TI_R_P3"

"678" 1.62354764307836e-21 1.04242295217853 0.542 0.348 5.4241103207605e-17 "PR2" "SDC3" "TI_R_P3"

"679" 1.73478891197291e-21 1.09909582353506 0.565 0.385 5.7957562760103e-17 "PR2" "NAXE" "TI_R_P3"

"680" 2.00962454420617e-21 1.09108480712004 0.44 0.261 6.71395463973841e-17 "PR2" "FAM213A" "TI_R_P3"

"681" 2.37802839595096e-21 1.6962607627389 0.66 0.482 7.94475506803256e-17 "PR2" "SMIM14" "TI_R_P3"

"682" 2.37837979156078e-21 1.31001057482631 0.83 0.667 7.94592904562542e-17 "PR2" "SEPT2" "TI_R_P3"

"683" 2.52937239816077e-21 1.04917790026848 0.593 0.414 8.4503802450153e-17 "PR2" "UFC1" "TI_R_P3"

"684" 2.64990492046819e-21 1.26174461003725 0.675 0.496 8.85306734879218e-17 "PR2" "RTRAF" "TI_R_P3"

"685" 2.7218749998688e-21 1.19071554861872 0.647 0.453 9.09351218706169e-17 "PR2" "CASP4" "TI_R_P3"

"686" 3.02971768690655e-21 1.15808471614841 0.598 0.423 1.01219838201861e-16 "PR2" "SRSF7" "TI_R_P3"

"687" 3.20076702631167e-21 1.02543339376296 0.733 0.584 1.06934425582047e-16 "PR2" "BRK1" "TI_R_P3"

"688" 4.14689331375838e-21 1.16797615111631 0.705 0.555 1.38543558719354e-16 "PR2" "RER1" "TI_R_P3"

"689" 6.58081582905652e-21 1.06203245743506 0.602 0.42 2.19858476032949e-16 "PR2" "IRAK1" "TI_R_P3"

"690" 7.97740025932293e-21 1.36961547012051 0.605 0.417 2.6651696526372e-16 "PR2" "ABHD12" "TI_R_P3"

"691" 8.39945863550679e-21 1.64314475883948 0.647 0.461 2.80617513553646e-16 "PR2" "EPN1" "TI_R_P3"

"692" 8.76423220252716e-21 1.30410085352115 0.64 0.47 2.9280423365423e-16 "PR2" "ALKBH5" "TI_R_P3"

"693" 1.53676336817781e-20 1.15881824681964 0.567 0.382 5.13417273674523e-16 "PR2" "NT5DC2" "TI_R_P3"

"694" 1.67644404796411e-20 1.3803248184316 0.623 0.436 5.6008319198433e-16 "PR2" "CHI3L2" "TI_R_P3"

"695" 1.91399151300296e-20 1.40200270940361 0.6 0.41 6.39445424579159e-16 "PR2" "BUD31" "TI_R_P3"

"696" 2.12123513208057e-20 1.5506666689187 0.475 0.288 7.08683445276799e-16 "PR2" "ABCC3" "TI_R_P3"

"697" 2.91760318646909e-20 1.44282701672241 0.718 0.559 9.74742048567458e-16 "PR2" "NDUFB8" "TI_R_P3"

"698" 3.68063802512363e-20 1.04870142109886 0.549 0.367 1.22966435781355e-15 "PR2" "TEX264" "TI_R_P3"

"699" 3.99391468647295e-20 1.35800445207256 0.589 0.402 1.33432695760375e-15 "PR2" "METTL9" "TI_R_P3"

"700" 4.23700278234883e-20 1.0280446624362 0.525 0.347 1.41554025955492e-15 "PR2" "THOC7" "TI_R_P3"

"701" 4.74307290354988e-20 1.79858889660505 0.575 0.39 1.58461322634698e-15 "PR2" "BTG1" "TI_R_P3"

"702" 5.97375646586076e-20 1.0166347556727 0.614 0.443 1.99577229767942e-15 "PR2" "LSM3" "TI_R_P3"

"703" 6.42635256107409e-20 2.02894949455932 0.686 0.526 2.14698012712924e-15 "PR2" "LAMTOR2" "TI_R_P3"

"704" 7.61411868881553e-20 1.2129843301285 0.558 0.373 2.54380091274638e-15 "PR2" "LUC7L3" "TI_R_P3"

"705" 8.26665606808643e-20 1.28147836292716 0.549 0.377 2.76180712578699e-15 "PR2" "SPTSSA" "TI_R_P3"

"706" 8.29516743933471e-20 1.32289236492571 0.567 0.387 2.77133248980733e-15 "PR2" "DPY30" "TI_R_P3"

"707" 8.39620933682282e-20 1.11500102411481 0.484 0.315 2.80508957733914e-15 "PR2" "DLC1" "TI_R_P3"

"708" 8.49958173494403e-20 1.68745224343022 0.777 0.645 2.83962526182745e-15 "PR2" "CAST" "TI_R_P3"

"709" 9.47144663101735e-20 1.43259671412718 0.614 0.423 3.16431560495659e-15 "PR2" "TNFSF10" "TI_R_P3"

"710" 9.71499845987399e-20 1.14767716900703 0.711 0.531 3.2456838354593e-15 "PR2" "PTBP1" "TI_R_P3"

"711" 1.18379390667198e-19 2.08909399104892 0.433 0.255 3.95493706280042e-15 "PR2" "MKNK2" "TI_R_P3"

"712" 1.35729141000767e-19 1.01842426876143 0.407 0.237 4.53457487169464e-15 "PR2" "LSM6" "TI_R_P3"

"713" 3.4315599210585e-19 1.030644715358 0.712 0.539 1.14644985402643e-14 "PR2" "CSNK2B" "TI_R_P3"

"714" 3.59215158961663e-19 1.03511781084091 0.625 0.461 1.20010192457502e-14 "PR2" "SF3B5" "TI_R_P3"

"715" 4.52116828439998e-19 1.85639050850199 0.577 0.413 1.51047711213519e-14 "PR2" "GORASP2" "TI_R_P3"

"716" 7.88400954432801e-19 3.25275273736018 0.572 0.39 2.63396874866454e-14 "PR2" "EWSR1" "TI_R_P3"

"717" 9.29702381802417e-19 1.08251227126881 0.575 0.4 3.1060426873637e-14 "PR2" "IFNAR1" "TI_R_P3"

"718" 1.04434768831745e-18 2.07422226326333 0.795 0.647 3.48906119189978e-14 "PR2" "SH3BGRL" "TI_R_P3"

"719" 1.23309944096729e-18 1.09912802879507 0.558 0.383 4.11966192232762e-14 "PR2" "CPD" "TI_R_P3"

"720" 1.2803069706415e-18 2.10442126684841 0.795 0.654 4.27737755821618e-14 "PR2" "LITAF" "TI_R_P3"

"721" 1.28062608661834e-18 1.25706620805761 0.851 0.737 4.27844369278321e-14 "PR2" "YWHAB" "TI_R_P3"

"722" 1.56348213587232e-18 1.30202233333531 0.64 0.468 5.22343746773584e-14 "PR2" "TAX1BP1" "TI_R_P3"

"723" 1.60191266602466e-18 1.0429184901568 0.558 0.386 5.35183002592179e-14 "PR2" "HPS1" "TI_R_P3"

"724" 1.60904239412102e-18 1.08850537410635 0.686 0.533 5.37564973451891e-14 "PR2" "CMPK1" "TI_R_P3"

"725" 1.86189181648483e-18 1.77631499171316 0.411 0.245 6.22039436969417e-14 "PR2" "HSPB11" "TI_R_P3"

"726" 1.90683380155339e-18 2.31738416111342 0.272 0.131 6.37054104760973e-14 "PR2" "SCGB3A2" "TI_R_P3"

"727" 3.53821711175901e-18 4.03746918818567 0.718 0.555 1.18208295486757e-13 "PR2" "ERO1A" "TI_R_P3"

"728" 4.8961026380786e-18 1.24308875904275 0.449 0.286 1.63573893035568e-13 "PR2" "EMC6" "TI_R_P3"

"729" 5.07408549236201e-18 1.19688832279238 0.539 0.36 1.69520122214322e-13 "PR2" "CCDC47" "TI_R_P3"

"730" 5.09781833574963e-18 2.23838866559243 0.442 0.275 1.7031301277906e-13 "PR2" "CCNC" "TI_R_P3"

"731" 7.05840944265354e-18 1.72699296782891 0.53 0.366 2.35814401069612e-13 "PR2" "SEC24C" "TI_R_P3"

"732" 9.50140885187762e-18 4.51728944970219 0.861 0.752 3.1743256833238e-13 "PR2" "ANXA1" "TI_R_P3"

"733" 1.06807338228785e-17 1.72370828314769 0.823 0.702 3.56832636288549e-13 "PR2" "GRINA" "TI_R_P3"

"734" 1.14650790859734e-17 1.20673540182117 0.663 0.498 3.83036827183286e-13 "PR2" "EIF5" "TI_R_P3"

"735" 3.43109685434569e-17 1.0029959586709 0.382 0.22 1.14629514806835e-12 "PR2" "RAI1" "TI_R_P3"

"736" 3.67254581233391e-17 1.2576536857974 0.511 0.338 1.22696083044264e-12 "PR2" "CYP27A1" "TI_R_P3"

"737" 4.3696987771212e-17 1.70817839578714 0.647 0.491 1.45987266444842e-12 "PR2" "PSMC1" "TI_R_P3"

"738" 6.26260560070771e-17 3.44644261096239 0.674 0.521 2.09227390514044e-12 "PR2" "GABARAP" "TI_R_P3"

"739" 1.10769045470279e-16 1.04252768508562 0.402 0.246 3.70068304011656e-12 "PR2" "PEX19" "TI_R_P3"

"740" 1.11997240304452e-16 1.26781875448938 0.679 0.531 3.74171580133144e-12 "PR2" "CTDSP1" "TI_R_P3"

"741" 1.26123495041935e-16 1.0953323677196 0.661 0.512 4.21365984585599e-12 "PR2" "EML4" "TI_R_P3"

"742" 1.87472159864319e-16 1.03663058356401 0.6 0.447 6.26325738890703e-12 "PR2" "NAA10" "TI_R_P3"

"743" 3.13976639720528e-16 2.89657066602233 0.661 0.5 1.04896455564231e-11 "PR2" "VEGFB" "TI_R_P3"

"744" 3.2790435713972e-16 1.19354354873724 0.561 0.393 1.09549566676809e-11 "PR2" "ACTR1A" "TI_R_P3"

"745" 4.05960967116123e-16 1.0593954491921 0.588 0.424 1.35627499503826e-11 "PR2" "CAPRIN1" "TI_R_P3"

"746" 4.78192465518797e-16 1.24703031566198 0.681 0.52 1.59759320805175e-11 "PR2" "TGOLN2" "TI_R_P3"

"747" 4.91264362625764e-16 1.03429432723065 0.446 0.289 1.64126510909642e-11 "PR2" "NIPA2" "TI_R_P3"

"748" 6.56930567364151e-16 1.35378884151104 0.491 0.323 2.19473933250689e-11 "PR2" "OTUD5" "TI_R_P3"

"749" 6.62906182636508e-16 1.18382235778469 0.523 0.365 2.21470326557031e-11 "PR2" "COX7A2L" "TI_R_P3"

"750" 7.49706686762357e-16 1.80434384256687 0.633 0.497 2.50469506980436e-11 "PR2" "STMN1" "TI_R_P3"

"751" 8.89305558972836e-16 1.2896961200887 0.404 0.251 2.97108094197235e-11 "PR2" "ROGDI" "TI_R_P3"

"752" 1.16015005755799e-15 1.56871830142979 0.623 0.465 3.8759453272955e-11 "PR2" "SPTAN1" "TI_R_P3"

"753" 1.83475327315732e-15 1.00808509584985 0.423 0.273 6.12972721029128e-11 "PR2" "MAOA" "TI_R_P3"

"754" 2.23573179014827e-15 1.00463020732269 0.711 0.56 7.46935633770636e-11 "PR2" "G6PD" "TI_R_P3"

"755" 4.51485950251133e-15 1.96532735740437 0.498 0.329 1.50836941119401e-10 "PR2" "EIF4E2" "TI_R_P3"

"756" 4.57539995915815e-15 1.80576002315953 0.567 0.416 1.52859537235515e-10 "PR2" "NFE2L2" "TI_R_P3"

"757" 5.10416125175434e-15 1.19693582482119 0.611 0.467 1.70524923259861e-10 "PR2" "PNRC1" "TI_R_P3"

"758" 5.65139858513003e-15 1.45452409438997 0.556 0.394 1.88807575330609e-10 "PR2" "CCNL2" "TI_R_P3"

"759" 6.46982063986618e-15 1.46999497944464 0.614 0.474 2.16150237757289e-10 "PR2" "BNIP3" "TI_R_P3"

"760" 9.97063254468547e-15 1.44154963047018 0.611 0.472 3.33108862685397e-10 "PR2" "EIF2S2" "TI_R_P3"

"761" 1.22636483886422e-14 1.51509899663148 0.365 0.22 4.09716229016148e-10 "PR2" "CD83" "TI_R_P3"

"762" 1.33103634774225e-14 1.08087228806107 0.586 0.435 4.44685933417209e-10 "PR2" "MAPK1" "TI_R_P3"

"763" 1.61840117163613e-14 1.02140734347692 0.346 0.21 5.40691647431916e-10 "PR2" "SEC23IP" "TI_R_P3"

"764" 1.70740132387811e-14 2.19768077937937 0.44 0.292 5.70425708294439e-10 "PR2" "TFDP1" "TI_R_P3"

"765" 2.41149089851289e-14 1.0272916820536 0.542 0.406 8.05654994284173e-10 "PR2" "CHMP3" "TI_R_P3"

"766" 3.21228799002851e-14 17.7514847537777 0.728 0.616 1.07319329458862e-09 "PR2" "MGP" "TI_R_P3"

"767" 3.3122575585887e-14 1.02260535192513 0.653 0.501 1.1065921277489e-09 "PR2" "NCOA4" "TI_R_P3"

"768" 4.81312891875741e-14 1.14870598093347 0.526 0.373 1.60801824046766e-09 "PR2" "CLTB" "TI_R_P3"

"769" 5.22929553408151e-14 1.29546078016489 0.488 0.335 1.74705534498129e-09 "PR2" "NAA50" "TI_R_P3"

"770" 5.26317181113269e-14 1.02969780468479 0.709 0.569 1.75837307038132e-09 "PR2" "PET100" "TI_R_P3"

"771" 7.88089310372994e-14 1.4155577642269 0.418 0.272 2.63292757702513e-09 "PR2" "TSC2" "TI_R_P3"

"772" 9.30076639681632e-14 3.18831426603474 0.761 0.649 3.10729304551236e-09 "PR2" "PSMB1" "TI_R_P3"

"773" 1.74755276835345e-13 1.57499091533213 0.335 0.202 5.83839904379205e-09 "PR2" "NUDCD2" "TI_R_P3"

"774" 1.88050745735685e-13 2.03618512821899 0.672 0.526 6.28258736428349e-09 "PR2" "ATP6V0D1" "TI_R_P3"

"775" 3.9188929728272e-13 1.16510274238548 0.588 0.426 1.30926295329184e-08 "PR2" "PDGFC" "TI_R_P3"

"776" 1.24027308774268e-12 1.03853616840752 0.482 0.344 4.14362835883952e-08 "PR2" "TMEM159" "TI_R_P3"

"777" 1.40295705843451e-12 1.33144313636467 0.446 0.304 4.68713923652385e-08 "PR2" "DIAPH1" "TI_R_P3"

"778" 1.7888618911955e-12 1.01891354521623 0.698 0.561 5.97640869229505e-08 "PR2" "NPTN" "TI_R_P3"

"779" 2.01215182441976e-12 1.95344885416364 0.489 0.353 6.72239803020397e-08 "PR2" "CKLF" "TI_R_P3"

"780" 3.57420311359244e-12 1.22693435715068 0.635 0.49 1.1941055182201e-07 "PR2" "GLG1" "TI_R_P3"

"781" 6.14036531087839e-12 1.06153334901916 0.398 0.262 2.05143464671136e-07 "PR2" "SPG7" "TI_R_P3"

"782" 7.24262879408336e-12 1.26893767114712 0.463 0.324 2.41968985381531e-07 "PR2" "LMBRD1" "TI_R_P3"

"783" 7.34262744017955e-12 1.4079853950173 0.375 0.251 2.45309840148959e-07 "PR2" "TOP2A" "TI_R_P3"

"784" 1.02230321912853e-11 1.64264722377797 0.723 0.608 3.41541282478651e-07 "PR2" "S100A9" "TI_R_P3"

"785" 1.05483426864881e-11 1.30704746340488 0.628 0.491 3.5240958081288e-07 "PR2" "SERINC1" "TI_R_P3"

"786" 1.76409568104739e-11 1.13314201985724 0.374 0.245 5.89366726081124e-07 "PR2" "PIM3" "TI_R_P3"

"787" 3.97525159606952e-11 3.41828050706952 0.732 0.611 1.32809180573087e-06 "PR2" "TGM2" "TI_R_P3"

"788" 6.02310500999172e-11 4.23128936521301 0.307 0.187 2.01225915278813e-06 "PR2" "AAAS" "TI_R_P3"

"789" 8.42283801063919e-11 1.11374145719624 0.633 0.509 2.81398595097445e-06 "PR2" "NECTIN2" "TI_R_P3"

"790" 3.23429991574403e-10 4.891276801729 0.384 0.269 1.08054725885092e-05 "PR2" "ADIRF" "TI_R_P3"

"791" 6.08961028127714e-10 3.88267901361704 0.556 0.425 2.03447789887188e-05 "PR2" "STOM" "TI_R_P3"

"792" 1.28627487432064e-09 2.50361567506377 0.423 0.3 4.29731572761784e-05 "PR2" "TSC22D1" "TI_R_P3"

"793" 1.97874648347697e-09 1.27437956413189 0.432 0.304 6.6107941266482e-05 "PR2" "NUB1" "TI_R_P3"

"794" 2.49240894236598e-09 2.62989032036881 0.454 0.336 8.32688903555051e-05 "PR2" "FABP3" "TI_R_P3"

"795" 3.86452573879893e-09 1.10768305318696 0.516 0.405 0.000129109940407534 "PR2" "ANP32B" "TI_R_P3"

"796" 9.34892853833637e-09 1.9563010917177 0.581 0.467 0.00031233835353728 "PR2" "GM2A" "TI_R_P3"

"797" 1.5253760906042e-08 1.23668299156673 0.584 0.477 0.000509612898109957 "PR2" "DAP" "TI_R_P3"

"798" 3.92846676635639e-08 1.71129112696765 0.579 0.473 0.00131246146197201 "PR2" "DAD1" "TI_R_P3"

"799" 2.60332869556416e-07 1.55718969208534 0.525 0.407 0.00869746083901031 "PR2" "MYOF" "TI_R_P3"

"800" 2.00932113632478e-144 47.8439765223365 0.363 0.019 6.71294098434747e-140 "PR3" "IGLC7" "TI_R_P3"

"801" 1.07400344653003e-65 2.90859718798883 0.745 0.273 3.58813811451216e-61 "PR3" "MIA" "TI_R_P3"

"802" 1.99132193668496e-59 4.84700886655591 0.991 0.868 6.65280745827079e-55 "PR3" "PGC" "TI_R_P3"

"803" 1.56602423751831e-43 2.66590398013049 0.958 0.748 5.23193037512491e-39 "PR3" "EZR" "TI_R_P3"

"804" 1.87228385466173e-36 1.45456283970794 0.92 0.6 6.25511313003936e-32 "PR3" "EFNA1" "TI_R_P3"

"805" 2.04810006169561e-36 3.07109709589848 0.887 0.662 6.84249749611888e-32 "PR3" "SERPINB1" "TI_R_P3"

"806" 2.74840217953394e-36 5.99317193998957 0.929 0.672 9.18213684160494e-32 "PR3" "POR" "TI_R_P3"

"807" 2.87993380377465e-35 4.67489371333486 0.972 0.819 9.62157084503074e-31 "PR3" "IGFBP2" "TI_R_P3"

"808" 3.81589138050806e-34 1.6307021154108 0.335 0.084 1.27485115131394e-29 "PR3" "DMBT1" "TI_R_P3"

"809" 3.90261241640378e-34 2.03042319599335 0.958 0.752 1.30382378219634e-29 "PR3" "RRBP1" "TI_R_P3"

"810" 4.66675290798963e-34 2.59311138214502 0.943 0.764 1.55911547903025e-29 "PR3" "CLDN4" "TI_R_P3"

"811" 1.06557406337001e-32 3.9649238927512 0.778 0.436 3.55997638831288e-28 "PR3" "SLC11A2" "TI_R_P3"

"812" 1.9303886884094e-31 1.57369410132411 0.844 0.572 6.44923556910698e-27 "PR3" "DYNLT1" "TI_R_P3"

"813" 1.48783686530052e-30 1.97970899567664 0.962 0.825 4.97071418328252e-26 "PR3" "PLTP" "TI_R_P3"

"814" 3.80014405690191e-30 2.80624304832383 0.792 0.484 1.26959012797036e-25 "PR3" "CLDN3" "TI_R_P3"

"815" 1.44849219198188e-29 2.58670070400165 0.825 0.507 4.83926756419226e-25 "PR3" "ITPR2" "TI_R_P3"

"816" 1.82674969235233e-29 2.66033743428998 0.991 0.839 6.10298804717991e-25 "PR3" "MAL2" "TI_R_P3"

"817" 2.32412872833102e-29 1.47884106806552 0.821 0.512 7.7646816684811e-25 "PR3" "LCN2" "TI_R_P3"

"818" 3.97078066591795e-29 2.3480748405752 0.901 0.654 1.32659811267653e-24 "PR3" "CCT2" "TI_R_P3"

"819" 4.8756191432121e-28 3.61438934308097 0.967 0.836 1.62889559955573e-23 "PR3" "CTDSP2" "TI_R_P3"

"820" 1.27341693798996e-27 2.58499419386845 0.981 0.875 4.25435864813067e-23 "PR3" "TXNDC17" "TI_R_P3"

"821" 1.34693367517357e-27 2.42180527976562 0.698 0.362 4.49997071538737e-23 "PR3" "LAMP3" "TI_R_P3"

"822" 3.99630396899725e-27 2.44251246107363 0.986 0.854 1.33512519300229e-22 "PR3" "COX6A1" "TI_R_P3"

"823" 7.43546979268664e-27 3.13946028260989 0.92 0.629 2.48411610303868e-22 "PR3" "MARCH9" "TI_R_P3"

"824" 2.2784776739009e-26 2.35243376475323 0.925 0.726 7.61216606073552e-22 "PR3" "EIF4A1" "TI_R_P3"

"825" 3.52086049791717e-26 1.34347153605613 0.906 0.651 1.17628428374915e-21 "PR3" "ZDHHC9" "TI_R_P3"

"826" 4.87109260593965e-26 1.62024810722723 0.976 0.8 1.62738332871838e-21 "PR3" "COX7A2" "TI_R_P3"

"827" 6.23643063007679e-26 1.16881520590781 0.443 0.166 2.08352910920235e-21 "PR3" "CTSE" "TI_R_P3"

"828" 7.09413419269111e-25 1.3697885053392 0.991 0.866 2.37007929243617e-20 "PR3" "COX7B" "TI_R_P3"

"829" 8.70826347513999e-25 2.48224547598616 0.816 0.533 2.90934374440952e-20 "PR3" "XPOT" "TI_R_P3"

"830" 1.10712804346392e-24 1.76901273650301 0.929 0.739 3.69880408040862e-20 "PR3" "HSPE1" "TI_R_P3"

"831" 3.13464696904388e-24 1.07921723122913 0.783 0.496 1.04725420588787e-19 "PR3" "TSFM" "TI_R_P3"

"832" 3.41028106555974e-24 1.05529464819798 0.991 0.888 1.13934080119285e-19 "PR3" "PDIA3" "TI_R_P3"

"833" 3.81381586265027e-24 4.39426585391723 0.967 0.855 1.27415774155283e-19 "PR3" "RAP1B" "TI_R_P3"

"834" 5.06070865118893e-24 1.30042849637756 0.844 0.57 1.69073215327571e-19 "PR3" "FKBP2" "TI_R_P3"

"835" 7.78145404679829e-24 1.01192890857762 0.755 0.444 2.59970598249484e-19 "PR3" "TNRC18" "TI_R_P3"

"836" 1.22589063881013e-23 2.9581533718801 0.84 0.604 4.09557803520076e-19 "PR3" "ACSS2" "TI_R_P3"

"837" 1.67590953439802e-23 2.78603930891392 0.953 0.769 5.59904616347034e-19 "PR3" "PTTG1IP" "TI_R_P3"

"838" 3.06022442765853e-23 2.58351533491009 0.939 0.745 1.02239037903644e-18 "PR3" "TRIM28" "TI_R_P3"

"839" 3.49908071211133e-23 2.89827844451824 0.807 0.53 1.16900787510927e-18 "PR3" "PLEKHA5" "TI_R_P3"

"840" 3.85558194118864e-23 1.49872127072448 0.868 0.592 1.28811137073171e-18 "PR3" "CD47" "TI_R_P3"

"841" 4.0813285679451e-23 1.35006964219078 0.66 0.371 1.36353106126478e-18 "PR3" "NDNF" "TI_R_P3"

"842" 5.03913084555503e-23 2.17961128521051 0.882 0.681 1.68352322419148e-18 "PR3" "PPP1R14B" "TI_R_P3"

"843" 5.24746672926423e-23 2.14115737602139 0.868 0.617 1.75312615957989e-18 "PR3" "HSPA9" "TI_R_P3"

"844" 1.24302066271805e-22 2.06528436930548 0.929 0.742 4.15280773207472e-18 "PR3" "XRCC6" "TI_R_P3"

"845" 2.44771387847281e-22 3.77271136401225 0.623 0.369 8.17756729658982e-18 "PR3" "TNNC2" "TI_R_P3"

"846" 2.46904111675762e-22 1.67431354214237 0.896 0.654 8.24881946697553e-18 "PR3" "AGRN" "TI_R_P3"

"847" 2.66073312621682e-22 1.19839033170583 0.92 0.757 8.88924330137777e-18 "PR3" "RAB21" "TI_R_P3"

"848" 2.92907328586478e-22 2.96445379423525 0.882 0.691 9.78574094074564e-18 "PR3" "HNRNPDL" "TI_R_P3"

"849" 5.15575280382479e-22 4.43740369623344 0.948 0.787 1.72248545422982e-17 "PR3" "YWHAE" "TI_R_P3"

"850" 5.89623307305157e-22 2.70863830570753 0.934 0.772 1.9698725073758e-17 "PR3" "GNB1" "TI_R_P3"

"851" 7.05485247740677e-22 1.72175843420498 0.953 0.768 2.35695566417683e-17 "PR3" "AQP3" "TI_R_P3"

"852" 1.10345252098475e-21 2.61657220877455 0.807 0.536 3.68652452735796e-17 "PR3" "TSPAN13" "TI_R_P3"

"853" 1.67518686010195e-21 1.79067901025877 0.925 0.766 5.59663178091461e-17 "PR3" "UBA1" "TI_R_P3"

"854" 1.82878869908357e-21 1.00641007008022 0.811 0.553 6.10980016476831e-17 "PR3" "GPC4" "TI_R_P3"

"855" 2.4270867717368e-21 1.16328993919967 0.675 0.376 8.10865419569546e-17 "PR3" "TCP1" "TI_R_P3"

"856" 3.22558901713557e-21 1.58596221015471 0.877 0.661 1.07763703473482e-16 "PR3" "GPX3" "TI_R_P3"

"857" 3.47240119430947e-21 2.76185389076849 0.943 0.797 1.16009451500685e-16 "PR3" "TMEM59" "TI_R_P3"

"858" 4.70278585563447e-21 1.97637437106128 0.915 0.712 1.57115372650892e-16 "PR3" "SERINC2" "TI_R_P3"

"859" 7.17562952429404e-21 2.71084532064696 0.825 0.555 2.39730606777139e-16 "PR3" "COL18A1" "TI_R_P3"

"860" 7.71881032650482e-21 1.73129677977822 0.623 0.357 2.578777341982e-16 "PR3" "SLC22A31" "TI_R_P3"

"861" 7.73490291982452e-21 1.19665722652785 0.66 0.374 2.58415371648417e-16 "PR3" "TMC5" "TI_R_P3"

"862" 1.01590442025809e-20 2.84690441753785 0.986 0.845 3.39403507764024e-16 "PR3" "UQCRB" "TI_R_P3"

"863" 1.15467744153951e-20 1.52362131021398 0.925 0.734 3.85766186443936e-16 "PR3" "SCD" "TI_R_P3"

"864" 1.63353309226852e-20 1.17165726639093 0.778 0.571 5.45747070795991e-16 "PR3" "G6PD" "TI_R_P3"

"865" 2.32296763573495e-20 2.86660916004413 0.703 0.416 7.76080257422689e-16 "PR3" "TMEM63B" "TI_R_P3"

"866" 2.43495980523457e-20 1.64679765100202 0.962 0.829 8.13495721330817e-16 "PR3" "CAPN2" "TI_R_P3"

"867" 3.34137123550173e-20 6.13307207888271 0.901 0.75 1.11631871606877e-15 "PR3" "PSMB4" "TI_R_P3"

"868" 3.98022788062465e-20 2.18001607300476 0.825 0.596 1.32975433263789e-15 "PR3" "TUBB4B" "TI_R_P3"

"869" 4.19765734015094e-20 1.51138910486202 0.679 0.397 1.40239534077103e-15 "PR3" "PHLDA2" "TI_R_P3"

"870" 5.09692524909801e-20 1.37348344607885 0.807 0.558 1.70283175647115e-15 "PR3" "VDAC1" "TI_R_P3"

"871" 5.77771356230251e-20 1.76409308098475 0.906 0.726 1.93027632402965e-15 "PR3" "S100A16" "TI_R_P3"

"872" 5.90050841740518e-20 2.89735547809423 0.816 0.568 1.9713008571709e-15 "PR3" "MPZL2" "TI_R_P3"

"873" 6.06893967842226e-20 1.28255258967145 0.783 0.543 2.02757205716409e-15 "PR3" "ELOC" "TI_R_P3"

"874" 9.71724692790419e-20 1.34489645827907 0.792 0.543 3.24643502614351e-15 "PR3" "DDR1" "TI_R_P3"

"875" 1.1256243732048e-19 2.21903471171952 0.816 0.576 3.76059846843992e-15 "PR3" "NKX2-1" "TI_R_P3"

"876" 1.95822096476934e-19 1.43475062803171 0.566 0.29 6.54222042119789e-15 "PR3" "FABP6" "TI_R_P3"

"877" 1.99902069408994e-19 1.23328897557518 0.797 0.59 6.67852823688507e-15 "PR3" "ETNK1" "TI_R_P3"

"878" 2.1675143203918e-19 1.91988400086098 0.943 0.737 7.24144859299698e-15 "PR3" "DYNLRB1" "TI_R_P3"

"879" 3.77881258420281e-19 1.94462589374513 0.774 0.485 1.26246349625632e-14 "PR3" "CYC1" "TI_R_P3"

"880" 4.42556590748133e-19 1.91732957782165 0.637 0.351 1.47853731403044e-14 "PR3" "GGCT" "TI_R_P3"

"881" 5.29168584171368e-19 2.52342327279703 0.665 0.376 1.76789932285812e-14 "PR3" "KIAA0319L" "TI_R_P3"

"882" 1.39792151112556e-18 4.20616734902865 0.887 0.696 4.67031597651938e-14 "PR3" "UQCRH" "TI_R_P3"

"883" 1.39857666029495e-18 1.37779808900284 0.651 0.383 4.67250476437939e-14 "PR3" "AGPAT3" "TI_R_P3"

"884" 2.01173166819865e-18 1.99179596726449 0.877 0.694 6.72099433028485e-14 "PR3" "RBX1" "TI_R_P3"

"885" 2.07982228374098e-18 4.50844981319695 0.91 0.78 6.94847826775022e-14 "PR3" "TMED9" "TI_R_P3"

"886" 2.086264658011e-18 2.53288741719303 0.75 0.517 6.97000159594893e-14 "PR3" "RASSF3" "TI_R_P3"

"887" 2.20007711475755e-18 1.07211002354374 0.717 0.441 7.35023763269349e-14 "PR3" "SLC35B2" "TI_R_P3"

"888" 3.12926439758372e-18 1.02537930833303 0.783 0.569 1.04545594258874e-13 "PR3" "NPTN" "TI_R_P3"

"889" 3.26343658792987e-18 3.92148580401248 0.92 0.785 1.09028152966149e-13 "PR3" "CAPZB" "TI_R_P3"

"890" 4.29902805224774e-18 1.10433977956773 0.783 0.552 1.43626228197545e-13 "PR3" "RNF213" "TI_R_P3"

"891" 4.9006769396883e-18 2.11072687236882 0.745 0.503 1.63726715878046e-13 "PR3" "DNTTIP1" "TI_R_P3"

"892" 4.98291501800533e-18 1.20396978354166 0.887 0.698 1.6647420783654e-13 "PR3" "RNASET2" "TI_R_P3"

"893" 6.12715322908351e-18 2.28316434560471 0.797 0.604 2.04702062230451e-13 "PR3" "CTTN" "TI_R_P3"

"894" 6.73273715328548e-18 1.88288505614967 0.745 0.511 2.24934015554115e-13 "PR3" "SF3B1" "TI_R_P3"

"895" 7.38099239207207e-18 1.24835280185821 0.679 0.418 2.46591574826736e-13 "PR3" "WASHC1" "TI_R_P3"

"896" 7.57141385787591e-18 1.99460012529253 0.75 0.537 2.52953365577776e-13 "PR3" "ITGA3" "TI_R_P3"

"897" 8.20807126139915e-18 1.53332129725103 0.533 0.278 2.74223452772084e-13 "PR3" "CTNNBL1" "TI_R_P3"

"898" 8.3977634768993e-18 1.54429674934385 0.849 0.624 2.80560879999729e-13 "PR3" "NDUFV2" "TI_R_P3"

"899" 1.05656507414786e-17 1.16727484137506 0.491 0.243 3.5298782562206e-13 "PR3" "LAMA5" "TI_R_P3"

"900" 1.32609849441076e-17 3.10941259560262 0.816 0.627 4.43036245997691e-13 "PR3" "EIF4H" "TI_R_P3"

"901" 1.58899999940605e-17 2.29166194264183 0.858 0.661 5.30869009801567e-13 "PR3" "ATF4" "TI_R_P3"

"902" 1.68237434718908e-17 1.14789836066041 0.84 0.586 5.620644456524e-13 "PR3" "NDUFA6" "TI_R_P3"

"903" 1.96062679922576e-17 1.17343682481992 0.821 0.615 6.55025807353335e-13 "PR3" "ARF3" "TI_R_P3"

"904" 2.31131187011036e-17 1.83695467561692 0.675 0.414 7.72186182685169e-13 "PR3" "GPRC5A" "TI_R_P3"

"905" 2.58378685643723e-17 1.20362725660187 0.594 0.341 8.63217350867116e-13 "PR3" "GALNT2" "TI_R_P3"

"906" 2.73060255353863e-17 1.49054790209138 0.929 0.777 9.1226700711172e-13 "PR3" "PCBP1" "TI_R_P3"

"907" 3.40404261228994e-17 6.28060204633219 0.948 0.847 1.13725659633995e-12 "PR3" "EEF2" "TI_R_P3"

"908" 3.59651067956064e-17 1.88643192876807 0.774 0.522 1.20155825293441e-12 "PR3" "MRPS35" "TI_R_P3"

"909" 4.18281613769807e-17 1.15064723037421 0.755 0.514 1.39743704344355e-12 "PR3" "NECTIN2" "TI_R_P3"

"910" 4.80565581717953e-17 1.40167323956783 0.83 0.619 1.60552155196151e-12 "PR3" "TUFM" "TI_R_P3"

"911" 4.83564317581675e-17 2.9117511157166 0.816 0.61 1.61554002860862e-12 "PR3" "TMED3" "TI_R_P3"

"912" 5.31513495725999e-17 1.44865275003638 0.67 0.399 1.77573343787099e-12 "PR3" "PI4KA" "TI_R_P3"

"913" 7.31378327067154e-17 1.19051919568225 0.67 0.429 2.44346185289865e-12 "PR3" "LLPH" "TI_R_P3"

"914" 7.39962113492107e-17 1.01380691568719 0.722 0.511 2.47213942496578e-12 "PR3" "MDH2" "TI_R_P3"

"915" 8.68302237511924e-17 1.7012352916465 0.811 0.587 2.90091094530359e-12 "PR3" "CLDN7" "TI_R_P3"

"916" 8.73091868208945e-17 1.34671730292407 0.778 0.534 2.91691262249927e-12 "PR3" "CDH1" "TI_R_P3"

"917" 9.78880639798544e-17 1.33253327119865 0.901 0.769 3.27034232950296e-12 "PR3" "EIF5A" "TI_R_P3"

"918" 1.35674472846204e-16 1.15094998774376 0.58 0.349 4.53274846331884e-12 "PR3" "MROH6" "TI_R_P3"

"919" 1.57210252909304e-16 1.23524200392673 0.736 0.514 5.25223733944695e-12 "PR3" "MID1IP1" "TI_R_P3"

"920" 1.57916771275337e-16 2.12212039175226 0.736 0.478 5.27584141153774e-12 "PR3" "OCIAD1" "TI_R_P3"

"921" 1.74212930108346e-16 2.29302882699816 0.755 0.513 5.82027978198974e-12 "PR3" "NFKBIA" "TI_R_P3"

"922" 1.82446775518737e-16 4.43309951822892 0.835 0.658 6.09536432330548e-12 "PR3" "SNU13" "TI_R_P3"

"923" 3.20527582391601e-16 4.72534506184829 0.925 0.791 1.0708506000121e-11 "PR3" "IDS" "TI_R_P3"

"924" 5.3032417090846e-16 3.62665537176856 0.656 0.425 1.77176002258807e-11 "PR3" "DPY19L1" "TI_R_P3"

"925" 5.33730495849241e-16 1.30128870453606 0.811 0.584 1.78314021358273e-11 "PR3" "ANXA6" "TI_R_P3"

"926" 7.10528063589711e-16 1.68091518226725 0.858 0.659 2.37380320764687e-11 "PR3" "COX17" "TI_R_P3"

"927" 7.73604156396109e-16 1.13789933575343 0.915 0.76 2.58453412610376e-11 "PR3" "CAPNS1" "TI_R_P3"

"928" 8.03757541201265e-16 1.43625186068755 0.915 0.751 2.68527356939931e-11 "PR3" "ECM1" "TI_R_P3"

"929" 9.08648603155364e-16 8.99994459793525 0.915 0.735 3.03570411828175e-11 "PR3" "TGFBI" "TI_R_P3"

"930" 1.26140358024023e-15 1.47681553110551 0.698 0.485 4.21422322122458e-11 "PR3" "RAD21" "TI_R_P3"

"931" 1.27132947057892e-15 1.36899094007432 0.491 0.262 4.24738462825712e-11 "PR3" "GOT1" "TI_R_P3"

"932" 1.44865166533081e-15 2.51307144112 0.873 0.703 4.8398003487037e-11 "PR3" "PRDX4" "TI_R_P3"

"933" 1.45035094104961e-15 1.83824857899983 0.623 0.383 4.84547745895264e-11 "PR3" "TSKU" "TI_R_P3"

"934" 1.65473112394281e-15 1.45670330673482 0.764 0.552 5.52829121198053e-11 "PR3" "VGLL4" "TI_R_P3"

"935" 1.68341314605301e-15 1.16437286150981 0.764 0.546 5.62411497964849e-11 "PR3" "PTBP1" "TI_R_P3"

"936" 1.6958201699915e-15 1.58510864675593 0.759 0.551 5.66556560592462e-11 "PR3" "LASP1" "TI_R_P3"

"937" 1.80166750886507e-15 1.30032227888805 0.632 0.383 6.01919098036732e-11 "PR3" "GALNT6" "TI_R_P3"

"938" 2.6680140867238e-15 1.6506897163749 0.67 0.442 8.91356826233554e-11 "PR3" "PARP14" "TI_R_P3"

"939" 3.42418364090907e-15 1.14029150836738 0.774 0.572 1.14398551259131e-10 "PR3" "GPAA1" "TI_R_P3"

"940" 3.54043302758792e-15 1.11519273252994 0.774 0.567 1.18282327018685e-10 "PR3" "TMEM30A" "TI_R_P3"

"941" 3.63459046547506e-15 1.25556323503463 0.807 0.578 1.21428032861056e-10 "PR3" "SRSF2" "TI_R_P3"

"942" 3.96250309209315e-15 1.17748297808732 0.561 0.338 1.3238326580374e-10 "PR3" "MROH1" "TI_R_P3"

"943" 4.24073304592965e-15 1.32210357383027 0.712 0.459 1.41678650331464e-10 "PR3" "RCC2" "TI_R_P3"

"944" 4.4479283853577e-15 1.10009132794292 0.467 0.25 1.48600839426415e-10 "PR3" "PLPP2" "TI_R_P3"

"945" 4.52261514986694e-15 1.25896888944359 0.835 0.722 1.51096049541905e-10 "PR3" "UBE2D3" "TI_R_P3"

"946" 4.65672376231033e-15 1.95928839234348 0.868 0.701 1.55576484175026e-10 "PR3" "SDF4" "TI_R_P3"

"947" 4.90798054969437e-15 1.2214809887272 0.792 0.587 1.63970722184739e-10 "PR3" "NME4" "TI_R_P3"

"948" 5.75489628470354e-15 2.2368544596955 0.651 0.41 1.92265329975661e-10 "PR3" "PDHA1" "TI_R_P3"

"949" 5.81425252339641e-15 1.35724918932992 0.646 0.405 1.94248362554151e-10 "PR3" "RASGRF1" "TI_R_P3"

"950" 5.93544667279985e-15 1.68421442645602 0.844 0.649 1.9829733789157e-10 "PR3" "ACTN1" "TI_R_P3"

"951" 5.98530356920764e-15 1.0760272901633 0.708 0.508 1.99963006943658e-10 "PR3" "ASPH" "TI_R_P3"

"952" 6.00767044052843e-15 2.82137871247954 0.764 0.535 2.00710261747614e-10 "PR3" "ZNF706" "TI_R_P3"

"953" 6.20049508465524e-15 1.4713293867538 0.731 0.515 2.07152340283247e-10 "PR3" "NUDC" "TI_R_P3"

"954" 7.27470141122888e-15 1.0038222536532 0.783 0.578 2.43040499447746e-10 "PR3" "RAN" "TI_R_P3"

"955" 1.00270295263014e-14 2.47501145328601 0.807 0.594 3.34993029444202e-10 "PR3" "TM9SF3" "TI_R_P3"

"956" 1.13016262988834e-14 1.84145834252513 0.538 0.301 3.77576033019395e-10 "PR3" "OLFML3" "TI_R_P3"

"957" 1.27943688785931e-14 7.56495832123136 0.844 0.649 4.27447069864917e-10 "PR3" "CIRBP" "TI_R_P3"

"958" 1.44839066982836e-14 1.38124639978747 0.797 0.568 4.83892838882957e-10 "PR3" "PSMD2" "TI_R_P3"

"959" 1.65623772417749e-14 1.48317343227333 0.745 0.54 5.53332461270458e-10 "PR3" "EMC10" "TI_R_P3"

"960" 1.65997844466178e-14 2.17601785573965 0.642 0.402 5.54582198577053e-10 "PR3" "TMPRSS2" "TI_R_P3"

"961" 1.66880640455311e-14 1.17987935178448 0.726 0.51 5.57531531697149e-10 "PR3" "PSMD13" "TI_R_P3"

"962" 1.67711298962724e-14 1.0413096062146 0.613 0.37 5.60306678704565e-10 "PR3" "LLGL2" "TI_R_P3"

"963" 1.77008350610285e-14 2.87312987517778 0.896 0.701 5.91367198553901e-10 "PR3" "PDIA4" "TI_R_P3"

"964" 2.24072318214845e-14 1.12253345419122 0.665 0.439 7.48603207923977e-10 "PR3" "RAB5IF" "TI_R_P3"

"965" 2.39735522389171e-14 1.51436558723333 0.783 0.576 8.0093240674998e-10 "PR3" "AKT1" "TI_R_P3"

"966" 2.57384975329577e-14 2.69081008075421 0.684 0.447 8.59897464078582e-10 "PR3" "PTK2" "TI_R_P3"

"967" 2.75292281517306e-14 2.11676843642483 0.745 0.526 9.19723983321168e-10 "PR3" "YWHAG" "TI_R_P3"

"968" 2.75868214518364e-14 3.69076367180026 0.863 0.721 9.21648117884401e-10 "PR3" "MAGED1" "TI_R_P3"

"969" 2.79917915874515e-14 1.6962139491923 0.594 0.357 9.35177765145166e-10 "PR3" "PTTG1" "TI_R_P3"

"970" 3.27403829904969e-14 1.50371717858803 0.533 0.303 1.09382345532951e-09 "PR3" "GGA1" "TI_R_P3"

"971" 3.38514648276116e-14 4.12246556141491 0.972 0.856 1.13094358842568e-09 "PR3" "CTSA" "TI_R_P3"

"972" 3.88711090498207e-14 2.43108484655323 0.759 0.539 1.29864488224546e-09 "PR3" "PSMB6" "TI_R_P3"

"973" 3.97663354309241e-14 2.19572634920039 0.792 0.589 1.32855350041174e-09 "PR3" "EIF4A2" "TI_R_P3"

"974" 4.14177990641103e-14 7.64986504003529 0.858 0.677 1.38372724893286e-09 "PR3" "GPI" "TI_R_P3"

"975" 4.14983463223655e-14 2.24162902947949 0.764 0.563 1.38641825228391e-09 "PR3" "CBX3" "TI_R_P3"

"976" 4.69859061740965e-14 1.58188449761458 0.684 0.45 1.56975213937039e-09 "PR3" "GDI1" "TI_R_P3"

"977" 4.72772534716939e-14 1.20891010659585 0.651 0.408 1.57948576123582e-09 "PR3" "VPS25" "TI_R_P3"

"978" 4.74668976472147e-14 1.00922993605068 0.741 0.539 1.5858215834958e-09 "PR3" "KRT10" "TI_R_P3"

"979" 4.90421763061549e-14 3.3830171065984 0.792 0.568 1.63845006821233e-09 "PR3" "APRT" "TI_R_P3"

"980" 5.44162758656737e-14 1.69808233267995 0.67 0.443 1.81799336039629e-09 "PR3" "SRSF6" "TI_R_P3"

"981" 5.46518586124288e-14 3.01820528828913 0.764 0.585 1.82586394438263e-09 "PR3" "JUP" "TI_R_P3"

"982" 5.76104621531633e-14 1.21925456844737 0.642 0.43 1.92470793007503e-09 "PR3" "ADAM15" "TI_R_P3"

"983" 6.24754837623932e-14 1.21033306850139 0.703 0.48 2.08724343701779e-09 "PR3" "CNIH4" "TI_R_P3"

"984" 7.51832872396805e-14 1.10815193564105 0.651 0.422 2.51179844339049e-09 "PR3" "OGDH" "TI_R_P3"

"985" 8.69188316453608e-14 1.87355233570389 0.42 0.21 2.90387124643986e-09 "PR3" "TPD52" "TI_R_P3"

"986" 9.34020247080172e-14 1.73883287575104 0.693 0.476 3.12046824347015e-09 "PR3" "SYNCRIP" "TI_R_P3"

"987" 9.41889320385807e-14 1.06195527944522 0.542 0.326 3.14675803047694e-09 "PR3" "FAM83H" "TI_R_P3"

"988" 1.11345240666848e-13 1.08515138178604 0.637 0.421 3.71993314543871e-09 "PR3" "SNTB1" "TI_R_P3"

"989" 1.2301320610981e-13 1.45356396261693 0.726 0.51 4.10974820292264e-09 "PR3" "TRIOBP" "TI_R_P3"

"990" 1.2890760077853e-13 1.00468631523963 0.547 0.314 4.30667403440992e-09 "PR3" "SMC1A" "TI_R_P3"

"991" 1.45791060908157e-13 1.29652483868835 0.816 0.658 4.8707335538806e-09 "PR3" "MINOS1" "TI_R_P3"

"992" 1.67053995901666e-13 1.07541966199722 0.75 0.585 5.58110694907877e-09 "PR3" "BAG6" "TI_R_P3"

"993" 1.80592419643566e-13 1.18929432713477 0.656 0.443 6.03341214787191e-09 "PR3" "PSMD3" "TI_R_P3"

"994" 1.83984132548472e-13 1.76357678859568 0.703 0.464 6.14672588431191e-09 "PR3" "WARS" "TI_R_P3"

"995" 2.02814268797176e-13 1.32450880155438 0.797 0.613 6.77582190624486e-09 "PR3" "PPP4C" "TI_R_P3"

"996" 2.23596998965142e-13 1.29650457406586 0.637 0.409 7.47015213842643e-09 "PR3" "MLEC" "TI_R_P3"

"997" 2.76612860946151e-13 1.21920279582308 0.759 0.506 9.24135907134996e-09 "PR3" "RBMX" "TI_R_P3"

"998" 2.97454268837391e-13 1.31825088747728 0.708 0.482 9.9376496675884e-09 "PR3" "EIF3B" "TI_R_P3"

"999" 3.5594866910736e-13 1.01346227633045 0.632 0.421 1.18918890862078e-08 "PR3" "KIF22" "TI_R_P3"

"1000" 3.97308740612557e-13 1.18584129793104 0.807 0.59 1.32736877151249e-08 "PR3" "FUS" "TI_R_P3"

"1001" 4.21864765781273e-13 1.04766801286897 0.5 0.279 1.40940799599866e-08 "PR3" "MICALL2" "TI_R_P3"

"1002" 4.58470347733526e-13 1.11165524234163 0.731 0.532 1.53170358474294e-08 "PR3" "CMTM6" "TI_R_P3"

"1003" 4.81405227509325e-13 3.8728060163617 0.778 0.555 1.6083267245859e-08 "PR3" "RNPEP" "TI_R_P3"

"1004" 4.85046083265088e-13 1.60242879268137 0.792 0.621 1.62049045958033e-08 "PR3" "RNF181" "TI_R_P3"

"1005" 4.86669989101757e-13 2.55367450909038 0.651 0.405 1.62591576659006e-08 "PR3" "SNRPD3" "TI_R_P3"

"1006" 5.29236707622302e-13 1.72455071922675 0.689 0.482 1.76812691649535e-08 "PR3" "SLC38A2" "TI_R_P3"

"1007" 5.47785322671786e-13 1.23626761782299 0.741 0.525 1.83009598451417e-08 "PR3" "LAMTOR5" "TI_R_P3"

"1008" 5.81424951098272e-13 4.06327334053865 0.632 0.441 1.94248261912422e-08 "PR3" "SLC2A1" "TI_R_P3"

"1009" 7.72103294158622e-13 2.80931334385053 0.618 0.387 2.57951989545454e-08 "PR3" "FAM173A" "TI_R_P3"

"1010" 8.4958694559417e-13 1.02308477947458 0.627 0.446 2.83838502653556e-08 "PR3" "YARS" "TI_R_P3"

"1011" 8.56990140887295e-13 1.09219971619346 0.618 0.407 2.86311836169036e-08 "PR3" "BRD2" "TI_R_P3"

"1012" 1.02436954523255e-12 1.10708777554238 0.689 0.505 3.42231621366744e-08 "PR3" "CCT8" "TI_R_P3"

"1013" 1.68082260073636e-12 1.34387347348181 0.571 0.346 5.61546022680012e-08 "PR3" "SREBF1" "TI_R_P3"

"1014" 1.76207410713798e-12 3.11806024960106 0.915 0.814 5.88691338453729e-08 "PR3" "LAPTM4A" "TI_R_P3"

"1015" 2.04839465660621e-12 1.15540113116386 0.646 0.443 6.84348170825568e-08 "PR3" "DYNC1H1" "TI_R_P3"

"1016" 2.20804467112646e-12 1.20016743225189 0.703 0.492 7.37685644176637e-08 "PR3" "SEC11C" "TI_R_P3"

"1017" 2.8150655427161e-12 2.27114324772997 0.741 0.547 9.4048524716602e-08 "PR3" "MAPKAPK2" "TI_R_P3"

"1018" 3.09982406383161e-12 1.00382104445736 0.528 0.322 1.0356202214855e-07 "PR3" "SLC35E3" "TI_R_P3"

"1019" 3.26061996891925e-12 2.45641634177998 0.642 0.407 1.08934052541623e-07 "PR3" "CLCN7" "TI_R_P3"

"1020" 3.32857932726198e-12 1.03021992279037 0.745 0.528 1.11204506744496e-07 "PR3" "YIPF3" "TI_R_P3"

"1021" 3.74209504360001e-12 1.14639780092481 0.717 0.526 1.25019653311633e-07 "PR3" "SUMF2" "TI_R_P3"

"1022" 3.84817669163981e-12 1.00958438889351 0.75 0.551 1.28563735090995e-07 "PR3" "FAM50A" "TI_R_P3"

"1023" 4.48563814736743e-12 1.0091339980282 0.703 0.522 1.49860684865398e-07 "PR3" "RBM39" "TI_R_P3"

"1024" 5.29603323799418e-12 1.23531617759338 0.797 0.63 1.76935174448148e-07 "PR3" "H2AFY" "TI_R_P3"

"1025" 6.51670927348485e-12 1.46913019889742 0.358 0.177 2.17716740117855e-07 "PR3" "CRTAC1" "TI_R_P3"

"1026" 7.80427662300944e-12 1.76314512467552 0.623 0.422 2.60733077698122e-07 "PR3" "FBXW5" "TI_R_P3"

"1027" 9.99618914787734e-12 1.44017521904277 0.637 0.427 3.33962683241434e-07 "PR3" "ADRM1" "TI_R_P3"

"1028" 1.03356014651576e-11 1.21101690042228 0.67 0.488 3.45302109349452e-07 "PR3" "TMEM173" "TI_R_P3"

"1029" 1.09140795231519e-11 2.64105573003526 0.868 0.681 3.64628482788983e-07 "PR3" "CNN2" "TI_R_P3"

"1030" 1.14766243950278e-11 1.07765344346727 0.557 0.349 3.83422544413484e-07 "PR3" "PSMB5" "TI_R_P3"

"1031" 1.27085833151158e-11 2.19143775629678 0.863 0.681 4.24581059974704e-07 "PR3" "SEPT2" "TI_R_P3"

"1032" 1.37356322708372e-11 1.38805207390846 0.712 0.49 4.58893738536399e-07 "PR3" "ADD1" "TI_R_P3"

"1033" 1.59669026536566e-11 2.52008109004922 0.59 0.404 5.33438250756013e-07 "PR3" "ERGIC2" "TI_R_P3"

"1034" 1.74730828330162e-11 1.02603647440949 0.604 0.407 5.83758224368238e-07 "PR3" "CYBC1" "TI_R_P3"

"1035" 1.80409791022273e-11 1.11019595931562 0.75 0.54 6.02731070826313e-07 "PR3" "CSNK1D" "TI_R_P3"

"1036" 1.81848604314261e-11 1.09294361568097 0.844 0.698 6.07538002153516e-07 "PR3" "PPP1CB" "TI_R_P3"

"1037" 1.97586801174808e-11 1.36523244083977 0.858 0.715 6.60117744044915e-07 "PR3" "ACTN4" "TI_R_P3"

"1038" 2.07881655335166e-11 1.1552884491383 0.689 0.5 6.94511822309256e-07 "PR3" "HIF1A" "TI_R_P3"

"1039" 2.26050747875416e-11 1.01738917856212 0.5 0.304 7.55212943576978e-07 "PR3" "BCAM" "TI_R_P3"

"1040" 2.27472788021687e-11 1.33124479193057 0.632 0.443 7.59963837501654e-07 "PR3" "GSDMD" "TI_R_P3"

"1041" 2.78321854846275e-11 1.58690050168136 0.764 0.581 9.29845484855922e-07 "PR3" "PPP2R1A" "TI_R_P3"

"1042" 3.18467791354319e-11 1.29450021620686 0.75 0.574 1.06396904413564e-06 "PR3" "RHBDD2" "TI_R_P3"

"1043" 3.28060672729036e-11 1.18482650019084 0.693 0.514 1.09601790152044e-06 "PR3" "NCOA4" "TI_R_P3"

"1044" 3.43810906143125e-11 1.77584290583807 0.547 0.348 1.14863785633357e-06 "PR3" "AP1B1" "TI_R_P3"

"1045" 3.45109023048977e-11 1.60113621812129 0.797 0.645 1.15297473510433e-06 "PR3" "ATP5PO" "TI_R_P3"

"1046" 4.30116707055324e-11 1.28270628842341 0.788 0.611 1.43697690660113e-06 "PR3" "MRPS21" "TI_R_P3"

"1047" 4.59079441037621e-11 1.14461462627198 0.646 0.481 1.53373850456259e-06 "PR3" "EFR3A" "TI_R_P3"

"1048" 5.36547690697947e-11 1.69469858799786 0.599 0.39 1.79255217985277e-06 "PR3" "DYRK2" "TI_R_P3"

"1049" 6.33074810068205e-11 1.05610588859026 0.571 0.372 2.11503963295687e-06 "PR3" "EDEM1" "TI_R_P3"

"1050" 6.87331692232969e-11 1.31255936557857 0.642 0.458 2.29630645058112e-06 "PR3" "NRBP1" "TI_R_P3"

"1051" 7.0752845380333e-11 1.17885024065122 0.726 0.571 2.36378181131155e-06 "PR3" "RAB34" "TI_R_P3"

"1052" 7.47242001706985e-11 2.34131094526062 0.755 0.575 2.49646080350287e-06 "PR3" "SON" "TI_R_P3"

"1053" 8.22296499547287e-11 1.21041043169233 0.656 0.452 2.74721037533753e-06 "PR3" "PSMA3" "TI_R_P3"

"1054" 9.12145131395524e-11 1.12614347803478 0.66 0.447 3.04738566947931e-06 "PR3" "IPO7" "TI_R_P3"

"1055" 9.44001196212063e-11 1.02585951634305 0.557 0.346 3.15381359642488e-06 "PR3" "PTPA" "TI_R_P3"

"1056" 9.81820721510775e-11 1.28292336352048 0.703 0.484 3.28016484849535e-06 "PR3" "ECH1" "TI_R_P3"

"1057" 1.01502576087565e-10 1.11910119556862 0.561 0.357 3.39109956450945e-06 "PR3" "ALDOA" "TI_R_P3"

"1058" 1.0261159050472e-10 4.67444936355972 0.443 0.259 3.42815062717218e-06 "PR3" "SLCO2A1" "TI_R_P3"

"1059" 1.05268513947148e-10 1.70623298545185 0.401 0.223 3.51691578246025e-06 "PR3" "RHPN1" "TI_R_P3"

"1060" 1.16118694901594e-10 1.54933078770992 0.741 0.569 3.87940947796736e-06 "PR3" "CTBP1" "TI_R_P3"

"1061" 1.20712193144407e-10 1.21605564986461 0.717 0.508 4.03287366076149e-06 "PR3" "SZRD1" "TI_R_P3"

"1062" 1.31514763787017e-10 1.39785819791034 0.698 0.51 4.39377674336044e-06 "PR3" "LMO7" "TI_R_P3"

"1063" 1.33911903236715e-10 1.1098422443636 0.453 0.266 4.47386277523543e-06 "PR3" "PYROXD1" "TI_R_P3"

"1064" 1.46798281850685e-10 1.05380559932801 0.557 0.357 4.90438379834954e-06 "PR3" "TNS3" "TI_R_P3"

"1065" 1.87228991678946e-10 1.73869829074295 0.722 0.542 6.25513338300191e-06 "PR3" "ATOX1" "TI_R_P3"

"1066" 1.8854899859116e-10 2.88866468556925 0.684 0.474 6.29923349393207e-06 "PR3" "HEBP2" "TI_R_P3"

"1067" 1.97906974166439e-10 1.00980396845808 0.613 0.411 6.61187409992656e-06 "PR3" "PSMA2" "TI_R_P3"

"1068" 2.09778519109941e-10 1.05078992760242 0.627 0.42 7.00849054494401e-06 "PR3" "ATP5MC1" "TI_R_P3"

"1069" 2.11074069722044e-10 1.03767506935198 0.689 0.519 7.05177359534377e-06 "PR3" "MZT2A" "TI_R_P3"

"1070" 2.17536609921081e-10 1.54568477035903 0.566 0.367 7.2676806008534e-06 "PR3" "DDIT3" "TI_R_P3"

"1071" 2.17680919270192e-10 1.60578369130906 0.896 0.77 7.27250183189786e-06 "PR3" "POSTN" "TI_R_P3"

"1072" 2.18614371905746e-10 1.11796378759821 0.66 0.488 7.30368755099906e-06 "PR3" "PSMC3" "TI_R_P3"

"1073" 2.37307109038666e-10 3.70044450687575 0.656 0.505 7.92819320587278e-06 "PR3" "PUF60" "TI_R_P3"

"1074" 2.39877784590918e-10 2.1963305795064 0.651 0.44 8.01407690539797e-06 "PR3" "AHR" "TI_R_P3"

"1075" 2.6861806867854e-10 1.11810651182052 0.825 0.675 8.97426105648136e-06 "PR3" "ARPC4" "TI_R_P3"

"1076" 2.70051059443279e-10 1.00119414011517 0.774 0.622 9.02213584494051e-06 "PR3" "PHC2" "TI_R_P3"

"1077" 2.74311575805857e-10 1.68867219851238 0.877 0.724 9.16447543609788e-06 "PR3" "CD164" "TI_R_P3"

"1078" 3.27530650098497e-10 1.02683515685733 0.453 0.262 1.09424714891407e-05 "PR3" "RFX5" "TI_R_P3"

"1079" 4.41006943490111e-10 1.36822456769585 0.858 0.704 1.47336009750611e-05 "PR3" "TUBA1A" "TI_R_P3"

"1080" 5.2896518891834e-10 1.46402936130276 0.528 0.333 1.76721979965728e-05 "PR3" "DPM2" "TI_R_P3"

"1081" 5.41158094848455e-10 1.91308461610433 0.514 0.328 1.8079550790792e-05 "PR3" "AC005332.7" "TI_R_P3"

"1082" 6.57482664024535e-10 1.20022999403028 0.467 0.281 2.19658383223957e-05 "PR3" "CEMIP2" "TI_R_P3"

"1083" 8.18465666048755e-10 1.41030376687209 0.656 0.478 2.73441194370229e-05 "PR3" "GUSB" "TI_R_P3"

"1084" 9.80819228358787e-10 1.20783392533314 0.547 0.359 3.27681896002387e-05 "PR3" "DHX9" "TI_R_P3"

"1085" 1.04455389812564e-09 1.89759012834643 0.769 0.613 3.48975011824796e-05 "PR3" "RUNX1" "TI_R_P3"

"1086" 1.06707751121315e-09 1.48732875510487 0.646 0.453 3.56499925721202e-05 "PR3" "PPP2CA" "TI_R_P3"

"1087" 1.10007876024953e-09 1.90833295196807 0.618 0.439 3.67525313011764e-05 "PR3" "OAZ2" "TI_R_P3"

"1088" 1.27033964717944e-09 2.1543468110626 0.821 0.651 4.24407772726179e-05 "PR3" "SOX4" "TI_R_P3"

"1089" 1.34711124954139e-09 1.35678698759417 0.759 0.601 4.50056397359283e-05 "PR3" "PERP" "TI_R_P3"

"1090" 1.3712968684499e-09 1.50004633004429 0.59 0.423 4.58136570780429e-05 "PR3" "CPNE1" "TI_R_P3"

"1091" 1.41992019883563e-09 1.00821515508853 0.684 0.482 4.74381139228995e-05 "PR3" "NHP2" "TI_R_P3"

"1092" 1.49668552204195e-09 1.25406783739525 0.689 0.5 5.00027666058994e-05 "PR3" "HADHA" "TI_R_P3"

"1093" 1.7194154684624e-09 15.8250460928455 0.953 0.85 5.74439513858604e-05 "PR3" "BGN" "TI_R_P3"

"1094" 1.79173841554862e-09 1.03224556387653 0.675 0.49 5.98601887250638e-05 "PR3" "EMC4" "TI_R_P3"

"1095" 2.32659090918246e-09 1.09564180584217 0.594 0.395 7.77290756848768e-05 "PR3" "ECI1" "TI_R_P3"

"1096" 2.5833109833739e-09 1.41067280635604 0.566 0.394 8.63058366435387e-05 "PR3" "YTHDF2" "TI_R_P3"

"1097" 2.7025197573883e-09 1.03807770640997 0.547 0.354 9.02884825745858e-05 "PR3" "FAH" "TI_R_P3"

"1098" 2.96820130207753e-09 1.32192020290668 0.665 0.505 9.91646373011082e-05 "PR3" "AEBP2" "TI_R_P3"

"1099" 3.18959578305642e-09 1.31778213150741 0.788 0.614 0.000106561205516132 "PR3" "WDR83OS" "TI_R_P3"

"1100" 3.24972070735995e-09 5.58442975724461 0.792 0.656 0.000108569919112189 "PR3" "COL4A2" "TI_R_P3"

"1101" 3.2602029968351e-09 1.76905721199948 0.59 0.395 0.000108920121921264 "PR3" "GRSF1" "TI_R_P3"

"1102" 3.31556964984993e-09 1.34990037313586 0.448 0.268 0.000110769866431836 "PR3" "FAR1" "TI_R_P3"

"1103" 3.58869126800366e-09 1.17512983051206 0.575 0.377 0.000119894586572734 "PR3" "CANT1" "TI_R_P3"

"1104" 4.16319768096012e-09 1.25700273822118 0.509 0.333 0.000139088271323197 "PR3" "MAN1B1" "TI_R_P3"

"1105" 4.31003355444372e-09 1.04484417013382 0.67 0.494 0.00014399391102041 "PR3" "C8orf59" "TI_R_P3"

"1106" 4.47551382088448e-09 4.29896094080469 0.939 0.809 0.00014952244124193 "PR3" "ARPC5" "TI_R_P3"

"1107" 4.67877159618469e-09 1.04573626490109 0.764 0.589 0.000156313080256934 "PR3" "PRKCSH" "TI_R_P3"

"1108" 4.9041459351433e-09 1.32804311280451 0.792 0.668 0.000163842611547202 "PR3" "SNX3" "TI_R_P3"

"1109" 5.49652287549057e-09 3.23633552405037 0.274 0.133 0.000183633332747265 "PR3" "ATF3" "TI_R_P3"

"1110" 6.31399491240575e-09 2.32092599984307 0.755 0.59 0.000210944256028564 "PR3" "SCAMP2" "TI_R_P3"

"1111" 6.48533025731837e-09 1.01186454974748 0.712 0.54 0.00021666839856675 "PR3" "SUB1" "TI_R_P3"

"1112" 9.1473572121627e-09 1.05314027928091 0.599 0.43 0.000305604057101144 "PR3" "GLS" "TI_R_P3"

"1113" 9.87402582416183e-09 1.35527905606919 0.684 0.509 0.000329881328759423 "PR3" "HNRNPA0" "TI_R_P3"

"1114" 9.91357667708739e-09 1.51906324354489 0.59 0.419 0.000331202683204813 "PR3" "RANBP1" "TI_R_P3"

"1115" 1.00175650452206e-08 1.16470392026291 0.632 0.463 0.000334676830595777 "PR3" "SNX17" "TI_R_P3"

"1116" 1.12768273054002e-08 1.42204088981412 0.472 0.298 0.000376747523446115 "PR3" "SLC35A2" "TI_R_P3"

"1117" 1.15073049985558e-08 1.2096953537072 0.472 0.295 0.00038444755269675 "PR3" "TMEM191B" "TI_R_P3"

"1118" 1.31352644585141e-08 1.62468584840051 0.613 0.454 0.000438836050294498 "PR3" "TOR1AIP2" "TI_R_P3"

"1119" 1.52967085054113e-08 3.83408702965847 0.854 0.715 0.000511047734457287 "PR3" "NNMT" "TI_R_P3"

"1120" 1.6027820924843e-08 1.06423761356212 0.613 0.442 0.00053547346927808 "PR3" "GPS1" "TI_R_P3"

"1121" 1.87667734854514e-08 1.35195395250981 0.675 0.503 0.000626979135375447 "PR3" "SNRPB" "TI_R_P3"

"1122" 1.90914699196215e-08 1.26361596339 0.59 0.419 0.000637826918544636 "PR3" "MAPRE1" "TI_R_P3"

"1123" 1.95672102791099e-08 1.00321338054676 0.778 0.623 0.000653720928214782 "PR3" "APP" "TI_R_P3"

"1124" 2.00208893700055e-08 1.05254679645851 0.552 0.388 0.000668877892962514 "PR3" "CBX5" "TI_R_P3"

"1125" 2.40245839594517e-08 1.73992001694287 0.778 0.665 0.000802637325501322 "PR3" "SELENOM" "TI_R_P3"

"1126" 2.47979217281204e-08 1.13419260540461 0.632 0.489 0.000828473767014776 "PR3" "ALOX15B" "TI_R_P3"

"1127" 2.58412194062493e-08 1.40922746463746 0.844 0.727 0.000863329299143384 "PR3" "NOP10" "TI_R_P3"

"1128" 2.63316425191175e-08 1.86268861500187 0.505 0.319 0.000879713844921198 "PR3" "NDN" "TI_R_P3"

"1129" 2.69188266681235e-08 1.20065939349963 0.604 0.444 0.000899331080155338 "PR3" "OBSL1" "TI_R_P3"

"1130" 2.87096301668996e-08 1.00134526312123 0.358 0.21 0.00095916003424595 "PR3" "SNHG12" "TI_R_P3"

"1131" 2.93476806412807e-08 1.39316392517154 0.505 0.332 0.000980476662544548 "PR3" "JPT2" "TI_R_P3"

"1132" 3.17553295527057e-08 1.13558181132581 0.429 0.265 0.00106091380502635 "PR3" "ROGDI" "TI_R_P3"

"1133" 3.45262987850311e-08 1.40109175896732 0.84 0.704 0.0011534891161091 "PR3" "CCND1" "TI_R_P3"

"1134" 3.92779897053035e-08 1.13262278056459 0.708 0.544 0.00131223835806448 "PR3" "PEA15" "TI_R_P3"

"1135" 4.80159277228143e-08 1.17260934849181 0.66 0.472 0.0016041641292915 "PR3" "LRP10" "TI_R_P3"

"1136" 5.34454102715878e-08 1.94765711391629 0.67 0.505 0.00178555771176348 "PR3" "LSM7" "TI_R_P3"

"1137" 5.63047510573026e-08 1.25647876825244 0.58 0.416 0.00188108542807342 "PR3" "ITGA5" "TI_R_P3"

"1138" 5.77838509155163e-08 1.03131897875833 0.552 0.374 0.00193050067523648 "PR3" "HADH" "TI_R_P3"

"1139" 6.05100776712942e-08 1.09523222315483 0.486 0.323 0.00202158118492027 "PR3" "RAB3IP" "TI_R_P3"

"1140" 6.14407451013902e-08 1.04028826032178 0.429 0.275 0.00205267385309234 "PR3" "IGFBP6" "TI_R_P3"

"1141" 7.2703048475654e-08 1.59333334459483 0.84 0.724 0.00242893614652312 "PR3" "SULF1" "TI_R_P3"

"1142" 7.55249104967712e-08 1.64102647148739 0.58 0.406 0.00252321173478663 "PR3" "FAT1" "TI_R_P3"

"1143" 8.32845030311484e-08 1.02616874639236 0.335 0.191 0.00278245196176764 "PR3" "CXorf38" "TI_R_P3"

"1144" 8.47244034213593e-08 2.60432550514956 0.509 0.344 0.00283055759390419 "PR3" "TP53I11" "TI_R_P3"

"1145" 9.06095169910101e-08 1.44364168842749 0.358 0.216 0.00302717335315266 "PR3" "DSP" "TI_R_P3"

"1146" 9.11088022596225e-08 1.20661240395743 0.627 0.444 0.00304385397469173 "PR3" "PSMD8" "TI_R_P3"

"1147" 9.56541918621649e-08 1.41957214187358 0.642 0.493 0.00319571089592307 "PR3" "FAM129B" "TI_R_P3"

"1148" 1.2278273965785e-07 1.02610052710217 0.505 0.335 0.00410204854922911 "PR3" "ZFAND3" "TI_R_P3"

"1149" 1.26700048741524e-07 1.58985808301559 0.642 0.468 0.00423292192840557 "PR3" "B4GALT1" "TI_R_P3"

"1150" 1.43028832345272e-07 1.00947610765426 0.679 0.506 0.0047784502598232 "PR3" "DBNL" "TI_R_P3"

"1151" 1.45570010398557e-07 2.41069557409826 0.571 0.409 0.00486334847740541 "PR3" "HNRNPH3" "TI_R_P3"

"1152" 1.48566363019763e-07 1.51197155308856 0.613 0.481 0.00496345362212725 "PR3" "CDV3" "TI_R_P3"

"1153" 1.71638940150081e-07 1.06654793367195 0.623 0.455 0.00573428535147407 "PR3" "TMEM167A" "TI_R_P3"

"1154" 1.78335665836608e-07 3.47041982758753 0.462 0.296 0.00595801625993525 "PR3" "FSCN1" "TI_R_P3"

"1155" 1.82646590045221e-07 1.64781934332984 0.533 0.376 0.00610203992682079 "PR3" "DEGS1" "TI_R_P3"

"1156" 2.07449902987528e-07 2.40301009448486 0.325 0.184 0.00693069380891032 "PR3" "TTC7A" "TI_R_P3"

"1157" 2.10988175057388e-07 2.88792506310886 0.392 0.232 0.00704890394049228 "PR3" "MEGF6" "TI_R_P3"

"1158" 2.35469539794335e-07 1.3842310499795 0.448 0.295 0.00786680185498894 "PR3" "TMEM222" "TI_R_P3"

"1159" 2.43870579094198e-07 1.99576887422424 0.538 0.389 0.00814747217695805 "PR3" "PA2G4" "TI_R_P3"

"1160" 2.60375093409071e-07 2.63521183626734 0.637 0.466 0.00869887149570365 "PR3" "MACC1" "TI_R_P3"

"1161" 2.74927104966974e-07 1.52335854241317 0.693 0.54 0.00918503964984164 "PR3" "CLIC4" "TI_R_P3"

"1162" 5.76943307774372e-15 1.46314024109286 0.644 0.349 1.9275098969434e-10 "PR1" "HABP2" "TI_R_P3"

"1163" 3.70225503203472e-11 1.35288033185805 0.785 0.533 1.23688638365248e-06 "PR1" "PCBD1" "TI_R_P3"

"1164" 9.76523676725349e-11 1.1073517323797 0.837 0.627 3.26246795157172e-06 "PR1" "SCP2" "TI_R_P3"

"1165" 1.08347629484146e-10 2.25680978803911 0.756 0.477 3.61978595343584e-06 "PR1" "CRNDE" "TI_R_P3"

"1166" 1.71059091909729e-10 2.05489428751087 0.889 0.712 5.71491320161215e-06 "PR1" "TESC" "TI_R_P3"

"1167" 1.03524880629788e-09 5.03768510643315 0.978 0.85 3.45866273696057e-05 "PR1" "ATP1B1" "TI_R_P3"

"1168" 1.40072944522326e-08 1.29791816329657 0.733 0.563 0.000467969700354639 "PR1" "ERBB3" "TI_R_P3"

"1169" 1.88488947841761e-08 2.72593502169983 0.896 0.76 0.00062972272584454 "PR1" "NDUFA2" "TI_R_P3"

"1170" 1.08845895035344e-07 1.17564739385387 0.667 0.45 0.00363643250723579 "PR1" "CDKN2A" "TI_R_P3"

"1171" 1.34147537657279e-07 1.14156616374528 0.667 0.461 0.00448173508559205 "PR1" "LSM3" "TI_R_P3"

"1172" 2.26002543648913e-08 1.4725414142377 0.715 0.597 0.000755051898076654 "PR2" "CDC42EP1" "TM_R_P3"

"1173" 2.6864580624716e-08 8.4606266320114 0.315 0.441 0.000897518774091137 "PR2" "CES1" "TM_R_P3"

"1174" 3.47287289687128e-11 5.73660596395212 0.977 0.819 1.16025210611573e-06 "PR1" "CXCL17" "TM_R_P3"

"1175" 5.85595211186516e-11 1.86891292474857 0.989 0.877 1.95641504105303e-06 "PR1" "ROMO1" "TM_R_P3"

"1176" 3.39049209757845e-09 1.43854172796242 0.966 0.818 0.000113272950487998 "PR1" "OCIAD2" "TM_R_P3"

"1177" 1.1467326264972e-08 1.10693331579745 0.563 0.309 0.000383111903186449 "PR1" "WDR66" "TM_R_P3"

"1178" 1.27467724010928e-08 3.27215015390674 0.943 0.835 0.00042585691914811 "PR1" "ASAH1" "TM_R_P3"

"1179" 1.99640197384655e-08 1.60064204151977 0.977 0.864 0.000666977935442394 "PR1" "PPIB" "TM_R_P3"

"1180" 1.02372333863699e-07 1.10988445357771 0.747 0.538 0.00342015730205231 "PR1" "MPC2" "TM_R_P3"

"1181" 1.07816279852644e-07 2.81229944396508 0.908 0.735 0.00360203409359698 "PR1" "BANF1" "TM_R_P3"

"1182" 1.52049591222907e-07 1.65804990147589 0.966 0.765 0.00507982479316611 "PR1" "TESC" "TM_R_P3"

"1183" 2.10324606328925e-07 1.02145263598153 0.793 0.539 0.00702673477284305 "PR1" "SNRPE" "TM_R_P3"

"1184" 2.85846587919471e-07 4.21465345896615 0.862 0.589 0.00954984865580161 "PR1" "TMEM54" "TM_R_P3"

"1185" 5.49040986401495e-21 4.00272612505979 0.778 0.587 1.83429103146875e-16 "PR3" "DDIT3" "TM_R_P3"

"1186" 8.37859063588745e-21 1.13341208622823 0.818 0.679 2.79920334554364e-16 "PR3" "HOPX" "TM_R_P3"

"1187" 4.08088423260949e-19 56.2157672092384 0.317 0.144 1.36338261327251e-14 "PR3" "SLPI" "TM_R_P3"

"1188" 7.47340690771584e-17 3.45835228911867 0.894 0.759 2.49679051379878e-12 "PR3" "TSTD1" "TM_R_P3"

"1189" 3.33244800269929e-16 1.31987038619141 0.794 0.664 1.11333755322181e-11 "PR3" "NDUFS6" "TM_R_P3"

"1190" 5.19443207148157e-16 4.43887879447095 0.887 0.778 1.73540781076128e-11 "PR3" "FOLR1" "TM_R_P3"

"1191" 1.53364949297516e-15 3.41765431376621 0.879 0.752 5.1237695910807e-11 "PR3" "CYB5A" "TM_R_P3"

"1192" 1.25431047383757e-13 1.99864926715755 0.818 0.709 4.19052586204392e-09 "PR3" "NDUFAF3" "TM_R_P3"

"1193" 8.44551015572779e-13 1.2306135174378 0.873 0.767 2.8215604879271e-08 "PR3" "SEC61G" "TM_R_P3"

"1194" 1.18822751675871e-11 1.20367743559367 0.844 0.737 3.96974931073916e-07 "PR3" "RBX1" "TM_R_P3"

"1195" 3.72203878306512e-11 1.10282225895582 0.755 0.626 1.24349593703423e-06 "PR3" "PCBD1" "TM_R_P3"

"1196" 3.73861471071586e-11 1.01498674479858 0.865 0.75 1.24903378870306e-06 "PR3" "TACSTD2" "TM_R_P3"

"1197" 7.93167572670631e-11 1.10096201742153 0.673 0.543 2.64989354353531e-06 "PR3" "ELOC" "TM_R_P3"

"1198" 6.55932829528733e-10 1.37783345278466 0.789 0.648 2.19140599017254e-05 "PR3" "LAMTOR2" "TM_R_P3"

"1199" 7.174941790761e-09 1.9716164894946 0.799 0.679 0.000239707630287534 "PR3" "GNG5" "TM_R_P3"

"1200" 1.54601621328679e-08 2.17439959784543 0.881 0.766 0.000516508556696985 "PR3" "PRDX4" "TM_R_P3"

"1201" 2.07276605627808e-08 1.11945504395591 0.731 0.607 0.000692490411741945 "PR3" "PPA1" "TM_R_P3"

"1202" 2.78226437983877e-08 1.34322932873701 0.747 0.645 0.000929526706660333 "PR3" "CDKN2A" "TM_R_P3"

"1203" 3.84152769722179e-08 1.56790776840414 0.821 0.717 0.00128341598836483 "PR3" "DHCR24" "TM_R_P3"

"1204" 6.55692615675205e-101 6.64770583696286 0.988 0.85 2.19060345970929e-96 "PR3" "TFF3" "TI_R_P2"

"1205" 2.23276997367942e-76 9.49422717933807 0.938 0.678 7.45946120506556e-72 "PR3" "HPGD" "TI_R_P2"

"1206" 5.69439203815592e-76 8.9692398384024 0.881 0.606 1.90243943602751e-71 "PR3" "SLC1A7" "TI_R_P2"

"1207" 6.27598705840808e-65 7.86904215806355 0.95 0.769 2.09674451634355e-60 "PR3" "SCGB2A1" "TI_R_P2"

"1208" 8.97268399198346e-53 2.83114333227889 0.878 0.672 2.99768399488176e-48 "PR3" "NUDT16L1" "TI_R_P2"

"1209" 5.24657421925468e-52 2.60898151508032 0.845 0.573 1.7528279809108e-47 "PR3" "PTPN13" "TI_R_P2"

"1210" 4.37232924351365e-51 8.29847834070659 0.427 0.149 1.46075147696548e-46 "PR3" "NPTX1" "TI_R_P2"

"1211" 1.72355141356227e-50 2.25608805531827 0.697 0.39 5.75821291757019e-46 "PR3" "BMP3" "TI_R_P2"

"1212" 3.40074709767482e-50 5.6683777116816 0.852 0.667 1.13615559786218e-45 "PR3" "DHRS3" "TI_R_P2"

"1213" 4.06796158887232e-48 12.0336624198858 0.964 0.86 1.35906528722635e-43 "PR3" "CIT" "TI_R_P2"

"1214" 7.59906116270348e-47 1.83233330346233 0.833 0.628 2.5387703438476e-42 "PR3" "MID1IP1" "TI_R_P2"

"1215" 2.2110369510008e-46 4.57589683558463 0.709 0.416 7.38685334959857e-42 "PR3" "CACNA2D2" "TI_R_P2"

"1216" 3.49250117950697e-46 2.92015636168149 0.749 0.458 1.16680971906148e-41 "PR3" "PON3" "TI_R_P2"

"1217" 5.52278471334442e-43 2.67254411396604 0.874 0.635 1.84510714488124e-38 "PR3" "GPRC5C" "TI_R_P2"

"1218" 6.37206530509749e-42 1.97053562052032 0.905 0.76 2.12884329778002e-37 "PR3" "CDC25B" "TI_R_P2"

"1219" 1.1577732301252e-41 3.41374153110062 0.897 0.732 3.86800458452527e-37 "PR3" "TMEM125" "TI_R_P2"

"1220" 3.43126707521148e-41 2.78648950371504 0.761 0.51 1.1463520171574e-36 "PR3" "PARM1" "TI_R_P2"

"1221" 4.49431314453068e-41 3.37562660153605 0.866 0.683 1.50150507845625e-36 "PR3" "RNF145" "TI_R_P2"

"1222" 3.72455896919941e-38 3.41810449642044 0.575 0.303 1.24433790601983e-33 "PR3" "FGFR3" "TI_R_P2"

"1223" 2.49956264560931e-36 3.66571305951323 0.876 0.753 8.35078884271613e-32 "PR3" "NKX2-1" "TI_R_P2"

"1224" 1.52922437176528e-35 1.60405214110121 0.895 0.741 5.10898570363063e-31 "PR3" "CCND1" "TI_R_P2"

"1225" 4.39779257951793e-35 2.01491344343877 0.89 0.751 1.46925852289115e-30 "PR3" "ANAPC11" "TI_R_P2"

"1226" 8.28009664565191e-35 1.86184769753702 0.618 0.363 2.76629748834585e-30 "PR3" "KIAA1324" "TI_R_P2"

"1227" 1.65116409389615e-34 2.00864031940246 0.84 0.644 5.51637412129764e-30 "PR3" "TOB1" "TI_R_P2"

"1228" 2.06241011069614e-34 2.93348384907421 0.845 0.636 6.89030593882474e-30 "PR3" "SEZ6L2" "TI_R_P2"

"1229" 2.18376389853643e-34 1.47026038022181 0.537 0.271 7.29573680862037e-30 "PR3" "PLCXD1" "TI_R_P2"

"1230" 1.37784843574919e-33 3.58886116890748 0.73 0.492 4.60325383899447e-29 "PR3" "HSD17B6" "TI_R_P2"

"1231" 1.60887060043158e-33 3.60539183302235 0.845 0.699 5.37507578898187e-29 "PR3" "CDK5RAP3" "TI_R_P2"

"1232" 2.14536386607269e-33 2.24032546839045 0.721 0.478 7.16744614016225e-29 "PR3" "GSDMB" "TI_R_P2"

"1233" 2.85199222936046e-33 2.1952873557312 0.711 0.46 9.52822083907037e-29 "PR3" "BCAM" "TI_R_P2"

"1234" 4.89995808134073e-33 5.35910013176599 0.842 0.702 1.63702699539512e-28 "PR3" "EPS8" "TI_R_P2"

"1235" 8.16123155203999e-33 3.19034508687683 0.878 0.691 2.72658584922104e-28 "PR3" "LUC7L3" "TI_R_P2"

"1236" 9.80089233200112e-33 3.62007896848315 0.728 0.485 3.27438011919825e-28 "PR3" "PRR15L" "TI_R_P2"

"1237" 1.249783012215e-32 1.15107836675606 0.716 0.455 4.1754000655091e-28 "PR3" "DOK4" "TI_R_P2"

"1238" 2.58366629190752e-32 4.68356807563634 0.828 0.646 8.63177071463384e-28 "PR3" "TBCD" "TI_R_P2"

"1239" 2.85885417363707e-32 5.93650699617286 0.924 0.805 9.55114590870409e-28 "PR3" "CDH1" "TI_R_P2"

"1240" 3.28926315324125e-32 3.41866988381644 0.754 0.506 1.09890992686637e-27 "PR3" "IRX3" "TI_R_P2"

"1241" 3.29682847947831e-32 1.08530840280298 0.687 0.43 1.10143742670891e-27 "PR3" "SCNN1B" "TI_R_P2"

"1242" 9.45333376917401e-32 1.25234080031866 0.854 0.702 3.15826427894334e-27 "PR3" "SLC50A1" "TI_R_P2"

"1243" 4.0974184052761e-31 4.0567225422627 0.885 0.77 1.36890651501869e-26 "PR3" "RRBP1" "TI_R_P2"

"1244" 4.66336745116669e-31 3.55065243836695 0.919 0.813 1.55798443176028e-26 "PR3" "SFTA3" "TI_R_P2"

"1245" 8.39400785142667e-31 2.60389244497577 0.866 0.745 2.80435408308314e-26 "PR3" "CCNL2" "TI_R_P2"

"1246" 9.77238525966309e-31 7.04116540154288 0.845 0.708 3.26485619140084e-26 "PR3" "YPEL3" "TI_R_P2"

"1247" 1.36858280327879e-30 7.09211394981987 0.718 0.503 4.57229828747411e-26 "PR3" "BTG3" "TI_R_P2"

"1248" 1.93436215754848e-30 1.37999370867306 0.833 0.652 6.46251053215371e-26 "PR3" "CCDC47" "TI_R_P2"

"1249" 3.30449279788559e-30 2.58813421488916 0.902 0.778 1.1039979988456e-25 "PR3" "F11R" "TI_R_P2"

"1250" 4.17919050972053e-30 2.1828319799232 0.759 0.539 1.39622575739253e-25 "PR3" "TMEM243" "TI_R_P2"

"1251" 4.45385773421032e-30 1.94732507953224 0.897 0.756 1.48798933042233e-25 "PR3" "PRKAR1A" "TI_R_P2"

"1252" 4.79900433737394e-30 3.87341018472475 0.912 0.8 1.60329935907326e-25 "PR3" "EFNA1" "TI_R_P2"

"1253" 9.8766281269739e-30 3.58674729468311 0.89 0.76 3.29968269094071e-25 "PR3" "WNK1" "TI_R_P2"

"1254" 1.44433436885354e-29 3.12057893812406 0.871 0.703 4.82537669290278e-25 "PR3" "MET" "TI_R_P2"

"1255" 2.22287325663701e-29 4.73728219610098 0.828 0.677 7.4263972630986e-25 "PR3" "GGTLC1" "TI_R_P2"

"1256" 2.32338060241663e-29 2.41600107944611 0.754 0.534 7.76218225461371e-25 "PR3" "CYB561" "TI_R_P2"

"1257" 2.90490576995899e-29 1.89813307359096 0.893 0.758 9.70499968685598e-25 "PR3" "MUC21" "TI_R_P2"

"1258" 2.96186078713772e-29 1.97620348721278 0.874 0.735 9.89528070374842e-25 "PR3" "CAPN1" "TI_R_P2"

"1259" 3.9376426776869e-29 1.11034868152035 0.542 0.316 1.31552704218842e-24 "PR3" "FAM129A" "TI_R_P2"

"1260" 4.2954320282844e-29 1.30569982332506 0.601 0.34 1.43506088632954e-24 "PR3" "GPD1L" "TI_R_P2"

"1261" 6.67682093234943e-29 2.25429579560375 0.716 0.494 2.23065910528862e-24 "PR3" "PLA2G10" "TI_R_P2"

"1262" 7.79182560338027e-29 4.01987586706705 0.831 0.662 2.60317101583332e-24 "PR3" "ERBB2" "TI_R_P2"

"1263" 8.80628380766083e-29 2.17572476970071 0.866 0.716 2.94209135730141e-24 "PR3" "RBM39" "TI_R_P2"

"1264" 1.32754918600678e-28 1.53674905701337 0.802 0.629 4.43520907553004e-24 "PR3" "KDELR1" "TI_R_P2"

"1265" 1.44627280060952e-28 4.27553565418547 0.921 0.777 4.83185279955636e-24 "PR3" "TMED9" "TI_R_P2"

"1266" 2.38181581709318e-28 1.9672547781867 0.778 0.613 7.9574084633266e-24 "PR3" "CNIH4" "TI_R_P2"

"1267" 3.67752965915944e-28 5.89357437550755 0.859 0.728 1.22862588382858e-23 "PR3" "TXNDC17" "TI_R_P2"

"1268" 3.6948500492772e-28 1.53764576182933 0.666 0.434 1.23441245296302e-23 "PR3" "SLC39A11" "TI_R_P2"

"1269" 4.87690013121497e-28 1.15831217613065 0.9 0.792 1.62932356483761e-23 "PR3" "MRPL20" "TI_R_P2"

"1270" 5.72298079692619e-28 3.92190326456448 0.735 0.546 1.91199065444507e-23 "PR3" "AP1G2" "TI_R_P2"

"1271" 6.07110938571085e-28 2.77650041160384 0.9 0.751 2.02829693467214e-23 "PR3" "NME3" "TI_R_P2"

"1272" 8.50333736392574e-28 3.39905423275846 0.881 0.743 2.84087997991395e-23 "PR3" "KIAA1522" "TI_R_P2"

"1273" 1.17813606836141e-27 1.17185877308957 0.647 0.401 3.93603479078864e-23 "PR3" "BICDL2" "TI_R_P2"

"1274" 1.82239802029208e-27 2.34813594514585 0.955 0.837 6.0884495459938e-23 "PR3" "UBE2D3" "TI_R_P2"

"1275" 2.00608043577615e-27 2.69873421261399 0.881 0.746 6.70211412788455e-23 "PR3" "KIAA0319L" "TI_R_P2"

"1276" 2.04547882934248e-27 1.92473329599356 0.568 0.344 6.83374022095028e-23 "PR3" "RDH10" "TI_R_P2"

"1277" 2.69208873711137e-27 2.3799746944211 0.73 0.523 8.99399926181537e-23 "PR3" "ERN2" "TI_R_P2"

"1278" 2.89494126293822e-27 2.12308859928416 0.835 0.694 9.67170926535031e-23 "PR3" "HSPA9" "TI_R_P2"

"1279" 3.89726176670556e-27 1.44319786171659 0.535 0.306 1.30203618363866e-22 "PR3" "CRYM" "TI_R_P2"

"1280" 4.46431965428192e-27 1.52923299305013 0.802 0.596 1.49148455329905e-22 "PR3" "CDC42BPA" "TI_R_P2"

"1281" 1.08573485506616e-26 3.38443153762197 0.761 0.576 3.62733157729052e-22 "PR3" "CENPX" "TI_R_P2"

"1282" 1.18452290751345e-26 1.60387506294723 0.792 0.615 3.95737258171168e-22 "PR3" "SNRNP200" "TI_R_P2"

"1283" 1.49687502933401e-26 2.03962426127576 0.742 0.518 5.00090978550201e-22 "PR3" "CPB2" "TI_R_P2"

"1284" 1.61698476784914e-26 2.49293568358318 0.752 0.562 5.40218441090718e-22 "PR3" "TMC4" "TI_R_P2"

"1285" 2.04064844650704e-26 3.42768535963523 0.792 0.635 6.81760239493536e-22 "PR3" "LSR" "TI_R_P2"

"1286" 2.1096983754164e-26 2.43463176057299 0.618 0.405 7.04829130242866e-22 "PR3" "HMGB3" "TI_R_P2"

"1287" 3.47260980294629e-26 1.20869610173589 0.816 0.625 1.16016420906633e-21 "PR3" "MTUS1" "TI_R_P2"

"1288" 4.03569750225351e-26 5.98956187296965 0.749 0.563 1.34828617852787e-21 "PR3" "SLC27A3" "TI_R_P2"

"1289" 5.45299339229089e-26 3.65690704731954 0.723 0.532 1.82179056243046e-21 "PR3" "COL9A2" "TI_R_P2"

"1290" 7.42070172523158e-26 1.89703757287702 0.659 0.449 2.47918223938262e-21 "PR3" "UNC13B" "TI_R_P2"

"1291" 8.18479674551815e-26 1.21697634036308 0.936 0.815 2.73445874471016e-21 "PR3" "IGFBP4" "TI_R_P2"

"1292" 1.08762420247639e-25 1.5836990243712 0.644 0.442 3.63364369805338e-21 "PR3" "FASN" "TI_R_P2"

"1293" 1.33836603022604e-25 2.03376559006924 0.594 0.368 4.47134707038219e-21 "PR3" "PPP1R13L" "TI_R_P2"

"1294" 1.4382596144711e-25 1.13142296343468 0.862 0.723 4.8050815459865e-21 "PR3" "NUMA1" "TI_R_P2"

"1295" 2.05105219383402e-25 1.22240201983493 0.68 0.462 6.85236027438009e-21 "PR3" "IRX2" "TI_R_P2"

"1296" 2.21519358442428e-25 1.27660590321618 0.585 0.345 7.40074024620306e-21 "PR3" "ETV4" "TI_R_P2"

"1297" 2.85315159937182e-25 1.1704012495479 0.504 0.281 9.5320941783413e-21 "PR3" "SNED1" "TI_R_P2"

"1298" 3.5348348408674e-25 2.92658862718161 0.749 0.561 1.18095297198539e-20 "PR3" "LAD1" "TI_R_P2"

"1299" 5.44834406604101e-25 1.26267855743307 0.871 0.699 1.82023726902364e-20 "PR3" "SCARB2" "TI_R_P2"

"1300" 6.92296667667584e-25 1.57698038614018 0.723 0.539 2.31289393701063e-20 "PR3" "CTDSP1" "TI_R_P2"

"1301" 2.22024305892932e-24 4.72106794716938 0.842 0.699 7.41761003557697e-20 "PR3" "SMIM14" "TI_R_P2"

"1302" 2.80932300534044e-24 5.04403991991496 0.795 0.633 9.38566722854188e-20 "PR3" "MLPH" "TI_R_P2"

"1303" 3.09553562111349e-24 1.7851594726034 0.79 0.606 1.03418749565781e-19 "PR3" "SNRNP70" "TI_R_P2"

"1304" 3.22361258532546e-24 2.84776443131172 0.871 0.718 1.07697672863138e-19 "PR3" "MYO6" "TI_R_P2"

"1305" 3.31547565203657e-24 1.07432917081413 0.547 0.32 1.1076672605889e-19 "PR3" "HOXD1" "TI_R_P2"

"1306" 3.56343593907879e-24 1.27164575123869 0.637 0.413 1.19050831288683e-19 "PR3" "NME1" "TI_R_P2"

"1307" 4.64837476546087e-24 1.46776681319474 0.74 0.533 1.55297552539282e-19 "PR3" "RHBDD2" "TI_R_P2"

"1308" 4.98489995035579e-24 1.64738018698501 0.728 0.52 1.66540522441437e-19 "PR3" "SMPDL3B" "TI_R_P2"

"1309" 5.01312731006053e-24 2.9244677136912 0.833 0.678 1.67483570301812e-19 "PR3" "TMEM9" "TI_R_P2"

"1310" 5.49660010754178e-24 2.53591287820111 0.783 0.62 1.83635912992863e-19 "PR3" "CTBP1" "TI_R_P2"

"1311" 5.75602840652403e-24 2.27522701409856 0.819 0.673 1.92303153033561e-19 "PR3" "GANAB" "TI_R_P2"

"1312" 6.12330523038096e-24 1.07971513210957 0.752 0.551 2.04573504441798e-19 "PR3" "PPP2R5A" "TI_R_P2"

"1313" 7.29065869947803e-24 1.06038410259062 0.881 0.721 2.43573616490862e-19 "PR3" "ERBB3" "TI_R_P2"

"1314" 8.12637747839198e-24 1.54875117633208 0.659 0.435 2.71494145175598e-19 "PR3" "ACLY" "TI_R_P2"

"1315" 8.93809512466129e-24 1.61983220069974 0.666 0.448 2.98612820019809e-19 "PR3" "AHCYL2" "TI_R_P2"

"1316" 1.03880565945082e-23 1.27602724064128 0.549 0.331 3.47054582765923e-19 "PR3" "VPS13D" "TI_R_P2"

"1317" 1.11485273762963e-23 1.66143611500584 0.831 0.682 3.72461151114683e-19 "PR3" "SPCS2" "TI_R_P2"

"1318" 1.12129646318507e-23 1.01488875490646 0.597 0.373 3.74613935385499e-19 "PR3" "DGAT2" "TI_R_P2"

"1319" 1.23362512364333e-23 2.37829594054219 0.8 0.623 4.12141817557999e-19 "PR3" "TMC6" "TI_R_P2"

"1320" 1.3480400710605e-23 1.90931256252071 0.754 0.57 4.50366707340601e-19 "PR3" "MMP15" "TI_R_P2"

"1321" 1.45408117831933e-23 1.44640308082997 0.757 0.571 4.85793980864706e-19 "PR3" "WASHC1" "TI_R_P2"

"1322" 1.56351586774981e-23 3.30686361659719 0.752 0.567 5.22355016256532e-19 "PR3" "RSRP1" "TI_R_P2"

"1323" 1.98204255988726e-23 2.07806520145722 0.697 0.482 6.62180598832733e-19 "PR3" "FASTK" "TI_R_P2"

"1324" 2.43348634229566e-23 1.94687534120662 0.702 0.509 8.13003452097557e-19 "PR3" "PTOV1" "TI_R_P2"

"1325" 2.54317753695863e-23 1.36623298433343 0.914 0.786 8.4965018332251e-19 "PR3" "PDIA6" "TI_R_P2"

"1326" 2.64173083930909e-23 2.98563346503684 0.599 0.388 8.82575856104775e-19 "PR3" "FAM20A" "TI_R_P2"

"1327" 3.02054883404281e-23 2.14346777243707 0.733 0.528 1.00913515996536e-18 "PR3" "HAS3" "TI_R_P2"

"1328" 3.17567440152723e-23 1.70759473410209 0.742 0.564 1.06096106080623e-18 "PR3" "MLLT6" "TI_R_P2"

"1329" 3.29209917952418e-23 1.51574578251211 0.835 0.67 1.09985741488723e-18 "PR3" "PDIA4" "TI_R_P2"

"1330" 3.30681938944597e-23 3.52425203320613 0.754 0.571 1.10477528982e-18 "PR3" "LLGL2" "TI_R_P2"

"1331" 3.43257961171029e-23 2.30050466106078 0.893 0.757 1.14679052247629e-18 "PR3" "ATP5PD" "TI_R_P2"

"1332" 3.46173784997812e-23 2.58969802535179 0.768 0.567 1.15653199829919e-18 "PR3" "TMEM167A" "TI_R_P2"

"1333" 4.51239687920238e-23 1.2770941264891 0.766 0.601 1.50754667337272e-18 "PR3" "MAT2A" "TI_R_P2"

"1334" 6.25326114968795e-23 2.92161690815513 0.833 0.694 2.08915201749925e-18 "PR3" "SLC38A10" "TI_R_P2"

"1335" 7.0495641822586e-23 2.81929549457069 0.878 0.754 2.35518889765078e-18 "PR3" "MRPS21" "TI_R_P2"

"1336" 7.72192066895828e-23 2.25028847705497 0.845 0.687 2.57981647629227e-18 "PR3" "PPCS" "TI_R_P2"

"1337" 9.07760417238417e-23 2.12796572962749 0.862 0.74 3.03273677795183e-18 "PR3" "DDR1" "TI_R_P2"

"1338" 9.21951675037161e-23 3.04036750146511 0.771 0.609 3.08014835113165e-18 "PR3" "EMC10" "TI_R_P2"

"1339" 9.91033625012095e-23 1.17773407635382 0.788 0.636 3.31094423780291e-18 "PR3" "CSNK1D" "TI_R_P2"

"1340" 1.12397964191354e-22 2.58777661583686 0.816 0.654 3.75510358566895e-18 "PR3" "APH1A" "TI_R_P2"

"1341" 1.14003576579368e-22 1.23503044036609 0.604 0.386 3.80874548994012e-18 "PR3" "FOXA2" "TI_R_P2"

"1342" 1.17004439862951e-22 1.72496574722911 0.759 0.584 3.90900133138132e-18 "PR3" "SLC25A39" "TI_R_P2"

"1343" 1.51036354235209e-22 2.37339052791501 0.542 0.321 5.04597355864409e-18 "PR3" "NSD2" "TI_R_P2"

"1344" 1.62361022899291e-22 1.42114282980781 0.785 0.625 5.42431941404241e-18 "PR3" "ELOVL1" "TI_R_P2"

"1345" 1.65068480384578e-22 1.2959288625439 0.897 0.78 5.51477286116837e-18 "PR3" "EIF3I" "TI_R_P2"

"1346" 1.96295728965401e-22 1.51516962188425 0.802 0.652 6.55804400900507e-18 "PR3" "METTL26" "TI_R_P2"

"1347" 2.93772586217517e-22 1.1871411277155 0.632 0.439 9.81464833294103e-18 "PR3" "SNHG12" "TI_R_P2"

"1348" 2.94599962177213e-22 2.51620155986177 0.802 0.661 9.8422901363785e-18 "PR3" "RBBP4" "TI_R_P2"

"1349" 3.01514141450714e-22 2.01999508004867 0.549 0.341 1.00732859517269e-17 "PR3" "SORCS2" "TI_R_P2"

"1350" 3.64649879688023e-22 1.86467200725119 0.778 0.593 1.21825878304971e-17 "PR3" "STUB1" "TI_R_P2"

"1351" 4.27572186586372e-22 1.05941166967682 0.611 0.398 1.42847591816641e-17 "PR3" "PHKG2" "TI_R_P2"

"1352" 4.28284254035998e-22 1.04377925227488 0.63 0.411 1.43085486430887e-17 "PR3" "ESRP1" "TI_R_P2"

"1353" 5.65559565994943e-22 1.65794133346227 0.785 0.617 1.88947795403251e-17 "PR3" "STARD10" "TI_R_P2"

"1354" 5.94268954288922e-22 1.33265823858146 0.649 0.452 1.98539314938386e-17 "PR3" "GNPAT" "TI_R_P2"

"1355" 6.84948205768699e-22 1.48270295747235 0.623 0.42 2.28834346065265e-17 "PR3" "MRPL27" "TI_R_P2"

"1356" 7.48232519417209e-22 1.63996716085386 0.589 0.378 2.49977002412095e-17 "PR3" "AK1" "TI_R_P2"

"1357" 9.58240023070486e-22 2.3754558196662 0.685 0.508 3.20138409307619e-17 "PR3" "KRT10" "TI_R_P2"

"1358" 1.04411771720353e-21 1.2704442215656 0.852 0.718 3.48829288140528e-17 "PR3" "FKBP2" "TI_R_P2"

"1359" 1.08890025429052e-21 2.80801386533353 0.714 0.523 3.63790685955919e-17 "PR3" "FGGY" "TI_R_P2"

"1360" 1.22673036777034e-21 4.42520720960173 0.773 0.623 4.09838348568392e-17 "PR3" "AUP1" "TI_R_P2"

"1361" 1.44337067677146e-21 1.09799131162961 0.699 0.501 4.82215709402579e-17 "PR3" "LIMCH1" "TI_R_P2"

"1362" 1.98194844110035e-21 3.0884269206891 0.79 0.673 6.62149154687216e-17 "PR3" "OCIAD1" "TI_R_P2"

"1363" 2.07642841514424e-21 3.45978260881347 0.883 0.749 6.93713969215539e-17 "PR3" "NDUFB10" "TI_R_P2"

"1364" 2.08233002261098e-21 2.85112747123262 0.874 0.742 6.95685637254101e-17 "PR3" "CCT3" "TI_R_P2"

"1365" 2.16387141428042e-21 1.30652249085919 0.63 0.442 7.22927800796945e-17 "PR3" "IYD" "TI_R_P2"

"1366" 2.16616432309268e-21 1.64836101832398 0.527 0.328 7.23693838702034e-17 "PR3" "HEXDC" "TI_R_P2"

"1367" 2.18727057086945e-21 1.76503384603123 0.835 0.714 7.30745225021774e-17 "PR3" "ILF2" "TI_R_P2"

"1368" 2.25800606698956e-21 2.66145073041236 0.723 0.534 7.54377246920542e-17 "PR3" "CRACR2B" "TI_R_P2"

"1369" 2.29124483468425e-21 1.19446626587333 0.737 0.554 7.65481986819663e-17 "PR3" "ANKH" "TI_R_P2"

"1370" 2.91429572847638e-21 2.0651154479209 0.783 0.628 9.73637059926673e-17 "PR3" "GPBP1L1" "TI_R_P2"

"1371" 3.41037695274572e-21 1.12828236645495 0.635 0.452 1.13937283614282e-16 "PR3" "TMEM33" "TI_R_P2"

"1372" 4.21378719201542e-21 1.98057471105077 0.895 0.782 1.40778416298043e-16 "PR3" "PSME2" "TI_R_P2"

"1373" 4.24362268366263e-21 1.39679741958653 0.733 0.559 1.41775190238485e-16 "PR3" "INTS11" "TI_R_P2"

"1374" 4.38220268980255e-21 2.5797966720061 0.69 0.506 1.46405009663613e-16 "PR3" "SPAG9" "TI_R_P2"

"1375" 4.59523757004675e-21 1.29558906538374 0.714 0.527 1.53522291977692e-16 "PR3" "NPLOC4" "TI_R_P2"

"1376" 4.67427301334229e-21 1.62890400357501 0.761 0.605 1.56162787102752e-16 "PR3" "EFCAB14" "TI_R_P2"

"1377" 5.00029187955188e-21 1.94506209286956 0.764 0.617 1.67054751403949e-16 "PR3" "MPZL1" "TI_R_P2"

"1378" 5.16449488279084e-21 2.84105599009816 0.711 0.532 1.72540609539159e-16 "PR3" "VAMP3" "TI_R_P2"

"1379" 6.83876975292146e-21 1.18883033653856 0.823 0.689 2.28476458675353e-16 "PR3" "NDUFS8" "TI_R_P2"

"1380" 6.90859246888808e-21 1.47363883861994 0.704 0.523 2.30809165793082e-16 "PR3" "CHTOP" "TI_R_P2"

"1381" 7.45253270252023e-21 1.4346702871157 0.659 0.477 2.48981665058498e-16 "PR3" "DDX42" "TI_R_P2"

"1382" 7.68393226430772e-21 1.17778342539534 0.647 0.438 2.56712493018257e-16 "PR3" "ZBTB18" "TI_R_P2"

"1383" 7.86637185597474e-21 1.56546648425846 0.637 0.445 2.6280761733626e-16 "PR3" "ZNF692" "TI_R_P2"

"1384" 7.90556553161968e-21 1.92878696817631 0.804 0.694 2.64117038845882e-16 "PR3" "C1orf43" "TI_R_P2"

"1385" 8.30745962229317e-21 2.76264114844756 0.78 0.632 2.77543918521192e-16 "PR3" "TMED4" "TI_R_P2"

"1386" 8.35441539285067e-21 1.98459829109242 0.723 0.574 2.79112663859748e-16 "PR3" "DNM2" "TI_R_P2"

"1387" 9.25639467284077e-21 1.29372543222467 0.663 0.491 3.09246889624937e-16 "PR3" "DCXR" "TI_R_P2"

"1388" 9.42404678864246e-21 2.09770071712977 0.692 0.518 3.14847979161756e-16 "PR3" "NBR1" "TI_R_P2"

"1389" 1.11160195264015e-20 1.41328956813032 0.757 0.577 3.71375096357549e-16 "PR3" "CACYBP" "TI_R_P2"

"1390" 1.21469023180735e-20 1.04638203258056 0.558 0.361 4.05815859544519e-16 "PR3" "CYP4V2" "TI_R_P2"

"1391" 1.25879649768307e-20 1.50629496514044 0.831 0.689 4.20551321910936e-16 "PR3" "RCN1" "TI_R_P2"

"1392" 1.40555754792779e-20 3.43168097007257 0.821 0.681 4.69582721187196e-16 "PR3" "TMEM30A" "TI_R_P2"

"1393" 1.43234766844491e-20 1.88672195699381 0.723 0.528 4.7853303255076e-16 "PR3" "NET1" "TI_R_P2"

"1394" 1.54129304591857e-20 1.08016213860408 0.735 0.572 5.14930593710935e-16 "PR3" "PDXDC1" "TI_R_P2"

"1395" 1.57033260787098e-20 1.7076687193776 0.785 0.641 5.24632420963616e-16 "PR3" "MARCH6" "TI_R_P2"

"1396" 1.70120624760879e-20 1.25813919593501 0.845 0.719 5.68355995263621e-16 "PR3" "NDUFC1" "TI_R_P2"

"1397" 1.76680242953984e-20 1.02622482644291 0.733 0.56 5.90271023684966e-16 "PR3" "OSBPL9" "TI_R_P2"

"1398" 2.09351544452527e-20 2.42231854695386 0.811 0.667 6.99422574861448e-16 "PR3" "NDFIP1" "TI_R_P2"

"1399" 2.13607021519404e-20 1.372926065146 0.778 0.62 7.13639698194175e-16 "PR3" "HYI" "TI_R_P2"

"1400" 2.31404247075052e-20 1.40579866891108 0.692 0.514 7.73098449053042e-16 "PR3" "TMEM159" "TI_R_P2"

"1401" 3.01021395165767e-20 1.12175722921768 0.704 0.542 1.00568237910931e-15 "PR3" "CTSF" "TI_R_P2"

"1402" 3.26517537845025e-20 1.94475178163816 0.714 0.549 1.09086244218645e-15 "PR3" "MGAT4B" "TI_R_P2"

"1403" 3.77661218318975e-20 1.73793011199184 0.74 0.551 1.26172836428186e-15 "PR3" "RABGAP1L" "TI_R_P2"

"1404" 4.05334289868189e-20 1.28499562088964 0.685 0.515 1.35418132902063e-15 "PR3" "LARP1" "TI_R_P2"

"1405" 4.33429476433609e-20 1.75699153569692 0.845 0.679 1.44804453781704e-15 "PR3" "ACSL5" "TI_R_P2"

"1406" 4.40986210649092e-20 1.06500874519097 0.47 0.274 1.47329083115755e-15 "PR3" "ZNF750" "TI_R_P2"

"1407" 4.57805899993914e-20 1.00245339702899 0.68 0.497 1.52948373128967e-15 "PR3" "TMEM87A" "TI_R_P2"

"1408" 4.71839549307134e-20 2.03528058633134 0.785 0.616 1.5763687502802e-15 "PR3" "TCIRG1" "TI_R_P2"

"1409" 5.00511880041491e-20 1.87944165328979 0.673 0.513 1.67216014003062e-15 "PR3" "PSMD3" "TI_R_P2"

"1410" 5.57008748546565e-20 1.52010606896333 0.563 0.359 1.86091052801922e-15 "PR3" "ELL2" "TI_R_P2"

"1411" 5.79228155456892e-20 3.93800742693947 0.826 0.696 1.93514334456593e-15 "PR3" "NCL" "TI_R_P2"

"1412" 6.17032708146107e-20 1.05977739092258 0.678 0.507 2.06144457464533e-15 "PR3" "SRSF1" "TI_R_P2"

"1413" 7.57656750781972e-20 1.18823999163126 0.754 0.591 2.53125543868749e-15 "PR3" "HSPD1" "TI_R_P2"

"1414" 9.40081587740773e-20 1.48375913976686 0.735 0.573 3.14071857648315e-15 "PR3" "PHLDA3" "TI_R_P2"

"1415" 9.69995427892178e-20 2.05587709817706 0.759 0.609 3.24065772504498e-15 "PR3" "DDX3X" "TI_R_P2"

"1416" 9.870849274369e-20 4.14837196709584 0.854 0.748 3.29775203407394e-15 "PR3" "TUFM" "TI_R_P2"

"1417" 1.00165880346e-19 1.28036351989086 0.63 0.448 3.34644189647952e-15 "PR3" "KAT2A" "TI_R_P2"

"1418" 1.01091564036442e-19 1.89785734222756 0.647 0.489 3.37736806289348e-15 "PR3" "IRF3" "TI_R_P2"

"1419" 1.06774561537062e-19 3.44520658914447 0.783 0.632 3.5672313263917e-15 "PR3" "ACADVL" "TI_R_P2"

"1420" 1.21226203681995e-19 1.22319058199813 0.53 0.334 4.05004623881177e-15 "PR3" "ATAD3B" "TI_R_P2"

"1421" 1.23910765918639e-19 1.14068894178152 0.508 0.309 4.13973477857582e-15 "PR3" "PDE4D" "TI_R_P2"

"1422" 1.25343711364063e-19 1.54615598571266 0.776 0.636 4.18760805296198e-15 "PR3" "CAMTA1" "TI_R_P2"

"1423" 1.3138855470712e-19 3.2915830613709 0.802 0.629 4.38956022421017e-15 "PR3" "TSC22D3" "TI_R_P2"

"1424" 1.58622227155977e-19 1.01300775431058 0.671 0.478 5.29940998705405e-15 "PR3" "MAPK13" "TI_R_P2"

"1425" 1.60510071843519e-19 1.37987397177403 0.807 0.687 5.36248099022013e-15 "PR3" "TMED2" "TI_R_P2"

"1426" 1.81432634068903e-19 1.07718624723013 0.723 0.557 6.06148287160796e-15 "PR3" "MAOA" "TI_R_P2"

"1427" 2.04255579730816e-19 1.06928785240603 0.587 0.384 6.82397466322682e-15 "PR3" "GLCCI1" "TI_R_P2"

"1428" 2.2100527283305e-19 1.07304205950535 0.711 0.549 7.38356516007935e-15 "PR3" "HDAC1" "TI_R_P2"

"1429" 2.4255741529926e-19 2.50991547748578 0.852 0.719 8.10360068773297e-15 "PR3" "SRSF11" "TI_R_P2"

"1430" 2.66176246338287e-19 1.23035353549966 0.582 0.379 8.89268221391583e-15 "PR3" "C4BPB" "TI_R_P2"

"1431" 2.77459706066613e-19 1.03542358369428 0.742 0.602 9.26965131997946e-15 "PR3" "DUSP23" "TI_R_P2"

"1432" 2.85236311286821e-19 1.23961001752172 0.516 0.329 9.52945992378142e-15 "PR3" "SMC4" "TI_R_P2"

"1433" 2.99584656758371e-19 2.00765018291945 0.792 0.641 1.00088237976404e-14 "PR3" "NDUFAB1" "TI_R_P2"

"1434" 3.16455753891906e-19 1.66827479316623 0.704 0.519 1.05724702817747e-14 "PR3" "BX284668.5" "TI_R_P2"

"1435" 3.23329072168459e-19 2.48912389280764 0.766 0.596 1.08021009720761e-14 "PR3" "COX17" "TI_R_P2"

"1436" 3.45847287004023e-19 2.25445560909958 0.754 0.612 1.15544120115174e-14 "PR3" "NENF" "TI_R_P2"

"1437" 3.70663346339528e-19 5.78629266826065 0.833 0.723 1.23834917378573e-14 "PR3" "BSG" "TI_R_P2"

"1438" 4.83442681170465e-19 2.28204452655039 0.745 0.589 1.61513365352241e-14 "PR3" "COL16A1" "TI_R_P2"

"1439" 4.94274766247589e-19 1.32873313532365 0.709 0.547 1.65132256655657e-14 "PR3" "C16orf58" "TI_R_P2"

"1440" 5.56289298025205e-19 1.00198777481162 0.609 0.405 1.85850691577241e-14 "PR3" "NOMO1" "TI_R_P2"

"1441" 6.08751751493517e-19 2.48378741304033 0.754 0.62 2.03377872656469e-14 "PR3" "TRIM28" "TI_R_P2"

"1442" 6.90425996873593e-19 1.14859379818455 0.821 0.691 2.30664421295499e-14 "PR3" "SF1" "TI_R_P2"

"1443" 6.90508903259327e-19 1.21212922881312 0.599 0.41 2.30692119489908e-14 "PR3" "DELE1" "TI_R_P2"

"1444" 7.62966163136304e-19 1.76133217297734 0.864 0.739 2.54899365442208e-14 "PR3" "OS9" "TI_R_P2"

"1445" 8.67912594728705e-19 2.92736935344209 0.838 0.732 2.89960918772913e-14 "PR3" "ADAR" "TI_R_P2"

"1446" 1.01641625591029e-18 1.15837638600076 0.68 0.516 3.3957450693707e-14 "PR3" "TARDBP" "TI_R_P2"

"1447" 1.02637517219122e-18 2.9645653201863 0.847 0.735 3.42901681277366e-14 "PR3" "RNF213" "TI_R_P2"

"1448" 1.10514882025464e-18 1.10912789372067 0.656 0.445 3.69219169358873e-14 "PR3" "TRA2A" "TI_R_P2"

"1449" 1.13899441702296e-18 3.53961582517931 0.618 0.429 3.80526644783202e-14 "PR3" "ARSD" "TI_R_P2"

"1450" 1.27130185202924e-18 1.09957608446775 0.721 0.542 4.24729235744448e-14 "PR3" "GNPTG" "TI_R_P2"

"1451" 1.39016131276978e-18 1.43641817874539 0.153 0.046 4.64438992983255e-14 "PR3" "DPEP1" "TI_R_P2"

"1452" 1.43531565065527e-18 1.40696822648336 0.84 0.715 4.79524605727419e-14 "PR3" "COPS9" "TI_R_P2"

"1453" 2.0298744850499e-18 2.25921737104254 0.737 0.55 6.7816076671032e-14 "PR3" "DHX15" "TI_R_P2"

"1454" 2.03819494682291e-18 1.3594898703916 0.625 0.445 6.80940549784066e-14 "PR3" "MYH14" "TI_R_P2"

"1455" 2.3013291957119e-18 1.8571452814331 0.704 0.557 7.68851070995389e-14 "PR3" "KLF5" "TI_R_P2"

"1456" 2.4450524312719e-18 1.12916922336763 0.527 0.339 8.1686756676363e-14 "PR3" "MKL2" "TI_R_P2"

"1457" 2.6984877242508e-18 2.42612247733946 0.678 0.494 9.01537763794948e-14 "PR3" "MRPS26" "TI_R_P2"

"1458" 3.17040431973767e-18 1.71097949653845 0.699 0.523 1.05920037918116e-13 "PR3" "TRAF4" "TI_R_P2"

"1459" 3.18398139228315e-18 1.75973956515186 0.869 0.745 1.06373634334788e-13 "PR3" "NDUFS6" "TI_R_P2"

"1460" 3.26067642894897e-18 1.29037868674843 0.508 0.31 1.08935938814756e-13 "PR3" "ATP10B" "TI_R_P2"

"1461" 3.68298512827032e-18 1.00354111410749 0.706 0.535 1.23044850150383e-13 "PR3" "HADH" "TI_R_P2"

"1462" 3.97767614507192e-18 1.25647349036679 0.601 0.409 1.32890182330708e-13 "PR3" "CENPB" "TI_R_P2"

"1463" 4.01338603540564e-18 1.03956542849872 0.706 0.526 1.34083214056867e-13 "PR3" "ARHGEF2" "TI_R_P2"

"1464" 4.27073969176766e-18 1.56765872350731 0.842 0.716 1.42681142362266e-13 "PR3" "ERP29" "TI_R_P2"

"1465" 4.95235453326395e-18 2.87251549341978 0.728 0.565 1.65453212601815e-13 "PR3" "TMEM54" "TI_R_P2"

"1466" 5.12081847217072e-18 1.9335325110317 0.685 0.531 1.71081424336752e-13 "PR3" "TXNDC11" "TI_R_P2"

"1467" 5.47950408570856e-18 2.13171634399753 0.747 0.581 1.83064751999437e-13 "PR3" "SIVA1" "TI_R_P2"

"1468" 5.60845481720631e-18 2.09221492012902 0.804 0.627 1.87372866988046e-13 "PR3" "SRSF7" "TI_R_P2"

"1469" 5.62363883970162e-18 1.81720345230635 0.666 0.496 1.87880149995592e-13 "PR3" "TRAP1" "TI_R_P2"

"1470" 5.90757305269016e-18 2.04724410678597 0.819 0.698 1.97366108117326e-13 "PR3" "DAP" "TI_R_P2"

"1471" 6.39304046909424e-18 1.36084783323051 0.527 0.335 2.13585089031969e-13 "PR3" "FOXK2" "TI_R_P2"

"1472" 6.40411758825087e-18 1.97656139330971 0.692 0.52 2.13955164505873e-13 "PR3" "TNFRSF1A" "TI_R_P2"

"1473" 6.77654701905864e-18 1.14638067763448 0.389 0.214 2.2639765935973e-13 "PR3" "TRAF5" "TI_R_P2"

"1474" 7.90325702216343e-18 1.58104104658135 0.623 0.437 2.64039913853458e-13 "PR3" "BRD8" "TI_R_P2"

"1475" 8.24614324321505e-18 1.03269953954106 0.73 0.56 2.75495399612572e-13 "PR3" "MRPS36" "TI_R_P2"

"1476" 8.70141460254533e-18 2.30866480200178 0.728 0.58 2.90705560456437e-13 "PR3" "GPI" "TI_R_P2"

"1477" 9.60883036745375e-18 1.25318748338214 0.594 0.399 3.21021413746262e-13 "PR3" "MTHFR" "TI_R_P2"

"1478" 9.64128845737744e-18 1.15244302167453 0.623 0.447 3.22105806072523e-13 "PR3" "YY1AP1" "TI_R_P2"

"1479" 9.70189139689688e-18 1.09392272676224 0.728 0.569 3.24130489678928e-13 "PR3" "MKNK2" "TI_R_P2"

"1480" 9.74997597747568e-18 1.09771726045921 0.759 0.613 3.25736947431485e-13 "PR3" "SNHG8" "TI_R_P2"

"1481" 9.93049500842119e-18 1.33590427679794 0.737 0.599 3.31767907736343e-13 "PR3" "GUSB" "TI_R_P2"

"1482" 1.28592196577705e-17 2.13601493915212 0.668 0.492 4.29613669546453e-13 "PR3" "NECTIN2" "TI_R_P2"

"1483" 1.28634510463938e-17 1.18545316678276 0.726 0.553 4.29755036008971e-13 "PR3" "HSD17B4" "TI_R_P2"

"1484" 1.29158002931799e-17 1.27049666380985 0.833 0.715 4.31503971994848e-13 "PR3" "HMGN1" "TI_R_P2"

"1485" 1.4672282918268e-17 1.75063302173495 0.733 0.603 4.90186300016417e-13 "PR3" "ADD1" "TI_R_P2"

"1486" 1.51781341436496e-17 1.05482312612165 0.728 0.587 5.07086283605188e-13 "PR3" "RAB5C" "TI_R_P2"

"1487" 1.59000051510888e-17 2.91344976605296 0.828 0.724 5.31203272092724e-13 "PR3" "AK2" "TI_R_P2"

"1488" 1.6916003625825e-17 2.02296810142857 0.776 0.577 5.65146765135189e-13 "PR3" "TMPRSS2" "TI_R_P2"

"1489" 2.09609868415363e-17 1.24812795702361 0.547 0.362 7.00285609388885e-13 "PR3" "ACSS1" "TI_R_P2"

"1490" 2.33890860879212e-17 1.59321273739509 0.814 0.675 7.8140597711136e-13 "PR3" "RBMX" "TI_R_P2"

"1491" 2.34948794635552e-17 1.52162093354394 0.735 0.569 7.84940427997916e-13 "PR3" "STIP1" "TI_R_P2"

"1492" 2.45125824977254e-17 1.05303283989568 0.783 0.612 8.18940868666506e-13 "PR3" "MACF1" "TI_R_P2"

"1493" 2.6177280703017e-17 1.44969049670828 0.845 0.729 8.74556771007095e-13 "PR3" "BANF1" "TI_R_P2"

"1494" 2.74093608060927e-17 1.30353224137923 0.735 0.564 9.15719335170751e-13 "PR3" "HSD17B11" "TI_R_P2"

"1495" 2.79303886459489e-17 2.89576258941524 0.699 0.526 9.33126354272507e-13 "PR3" "PGRMC2" "TI_R_P2"

"1496" 2.87277701750078e-17 2.5759356134035 0.814 0.698 9.59766073776835e-13 "PR3" "COX14" "TI_R_P2"

"1497" 2.92378116756322e-17 1.60375883266537 0.697 0.517 9.76806050271197e-13 "PR3" "RRAS" "TI_R_P2"

"1498" 3.05870189102446e-17 1.1411594091902 0.508 0.32 1.02188171477236e-12 "PR3" "SAE1" "TI_R_P2"

"1499" 3.67502573958097e-17 1.03219200627356 0.816 0.692 1.2277893493366e-12 "PR3" "VKORC1" "TI_R_P2"

"1500" 4.168458741594e-17 1.67734185879485 0.74 0.622 1.39264038097914e-12 "PR3" "GOLPH3" "TI_R_P2"

"1501" 4.19428115714049e-17 1.49823828329676 0.699 0.536 1.40126739178907e-12 "PR3" "INTS1" "TI_R_P2"

"1502" 4.3411448056214e-17 1.02622706439357 0.671 0.496 1.45033306811005e-12 "PR3" "SMARCA5" "TI_R_P2"

"1503" 4.70850282877222e-17 1.03514412485449 0.547 0.351 1.57306371006451e-12 "PR3" "FUT8" "TI_R_P2"

"1504" 4.89860599169739e-17 1.09158889561261 0.592 0.416 1.63657527576618e-12 "PR3" "LTBR" "TI_R_P2"

"1505" 4.90552692187179e-17 1.32557238946617 0.537 0.36 1.63888748932815e-12 "PR3" "MARVELD2" "TI_R_P2"

"1506" 5.4442389156957e-17 1.01378318457548 0.585 0.42 1.81886577934478e-12 "PR3" "PTK2" "TI_R_P2"

"1507" 5.55325726240441e-17 1.39679774423078 0.733 0.558 1.85528771879669e-12 "PR3" "PARP1" "TI_R_P2"

"1508" 6.00882159766438e-17 1.2617449687952 0.563 0.374 2.00748720756369e-12 "PR3" "RETREG3" "TI_R_P2"

"1509" 7.42401873171415e-17 1.04658193522488 0.671 0.494 2.48029041807838e-12 "PR3" "NUBP2" "TI_R_P2"

"1510" 7.78197443991288e-17 1.38574298498714 0.704 0.536 2.59987984063049e-12 "PR3" "INPPL1" "TI_R_P2"

"1511" 9.41532228219734e-17 2.488039151053 0.666 0.496 3.14556502125931e-12 "PR3" "EHD2" "TI_R_P2"

"1512" 1.03285151361672e-16 1.30084718279101 0.57 0.405 3.45065362184209e-12 "PR3" "CLK2" "TI_R_P2"

"1513" 1.07302665922586e-16 1.00738776721736 0.566 0.384 3.58487476580768e-12 "PR3" "ARL16" "TI_R_P2"

"1514" 1.26509196195946e-16 1.59425859998216 0.757 0.645 4.22654573571036e-12 "PR3" "LAMTOR1" "TI_R_P2"

"1515" 1.34045273233417e-16 1.52244862431829 0.749 0.575 4.47831853345521e-12 "PR3" "GLS" "TI_R_P2"

"1516" 1.36870598700069e-16 1.1103709835537 0.504 0.321 4.57270983197062e-12 "PR3" "SNRNP40" "TI_R_P2"

"1517" 1.50962478456486e-16 1.06351508952792 0.592 0.43 5.04350544275274e-12 "PR3" "TMX4" "TI_R_P2"

"1518" 1.5601116212542e-16 1.34108116264772 0.857 0.751 5.21217691544817e-12 "PR3" "CSNK1A1" "TI_R_P2"

"1519" 1.63793125364968e-16 1.31217758519513 0.702 0.555 5.4721645253182e-12 "PR3" "SCNN1A" "TI_R_P2"

"1520" 1.65759125986484e-16 1.42807993089886 0.685 0.513 5.53784664008245e-12 "PR3" "HECTD1" "TI_R_P2"

"1521" 1.73369720963539e-16 1.51916351982912 0.699 0.557 5.79210900767088e-12 "PR3" "NRDC" "TI_R_P2"

"1522" 1.83035914467893e-16 1.06078163960343 0.8 0.658 6.11504686645784e-12 "PR3" "RNF181" "TI_R_P2"

"1523" 2.03419070481981e-16 1.51164477119862 0.663 0.474 6.79602772573249e-12 "PR3" "SRSF4" "TI_R_P2"

"1524" 2.63890943644063e-16 1.26432100838352 0.776 0.634 8.81633253620449e-12 "PR3" "RBM47" "TI_R_P2"

"1525" 2.73581378499713e-16 1.69000120561936 0.726 0.575 9.1400802742969e-12 "PR3" "GSPT1" "TI_R_P2"

"1526" 2.7647712574118e-16 1.38311269496759 0.752 0.578 9.23682429388709e-12 "PR3" "EIF2AK1" "TI_R_P2"

"1527" 2.94082701655877e-16 1.69555031220573 0.814 0.694 9.8250089796212e-12 "PR3" "CEBPA" "TI_R_P2"

"1528" 2.9855779001526e-16 1.06628387380922 0.716 0.541 9.97451720661983e-12 "PR3" "LEPROT" "TI_R_P2"

"1529" 3.06354983712955e-16 1.28754857866537 0.687 0.502 1.02350136508661e-11 "PR3" "ARL6IP1" "TI_R_P2"

"1530" 3.27203022839894e-16 1.87568746562329 0.695 0.534 1.0931525790058e-11 "PR3" "CLK1" "TI_R_P2"

"1531" 3.50160359710834e-16 1.91065634404333 0.601 0.427 1.16985074575793e-11 "PR3" "MRFAP1L1" "TI_R_P2"

"1532" 3.50183863493861e-16 1.01146813565545 0.742 0.597 1.16992926954664e-11 "PR3" "RGL2" "TI_R_P2"

"1533" 3.56282858311775e-16 1.11345100672118 0.594 0.43 1.19030540133381e-11 "PR3" "TRAPPC6A" "TI_R_P2"

"1534" 3.88590404792095e-16 1.17611726926375 0.802 0.68 1.29824168336991e-11 "PR3" "CHD4" "TI_R_P2"

"1535" 4.58739353302078e-16 1.09174731791979 0.542 0.358 1.53260230544691e-11 "PR3" "ZNF672" "TI_R_P2"

"1536" 5.17614862674659e-16 1.98067428077537 0.637 0.463 1.72929949470977e-11 "PR3" "C5orf15" "TI_R_P2"

"1537" 5.8187196813426e-16 1.15642237871755 0.788 0.639 1.94397605833975e-11 "PR3" "TMBIM4" "TI_R_P2"

"1538" 5.86489927794319e-16 2.07267709058733 0.625 0.453 1.95940419976804e-11 "PR3" "ZDHHC9" "TI_R_P2"

"1539" 6.01097476775317e-16 1.07101278274697 0.714 0.574 2.00820656015866e-11 "PR3" "RHOT2" "TI_R_P2"

"1540" 6.03628556638452e-16 1.52042869272169 0.842 0.726 2.0166626448734e-11 "PR3" "AHNAK" "TI_R_P2"

"1541" 6.34989884959071e-16 1.11673631387964 0.675 0.497 2.12143770665976e-11 "PR3" "TMX2" "TI_R_P2"

"1542" 7.44138643604728e-16 1.20160636832995 0.802 0.658 2.48609279441904e-11 "PR3" "PTTG1IP" "TI_R_P2"

"1543" 8.77137810112496e-16 3.53296576874147 0.566 0.385 2.93042970980484e-11 "PR3" "SORBS2" "TI_R_P2"

"1544" 8.81055359361653e-16 1.22901368875956 0.513 0.34 2.94351785009135e-11 "PR3" "RAB17" "TI_R_P2"

"1545" 8.96992058376093e-16 1.62093487524385 0.666 0.513 2.99676076782869e-11 "PR3" "DUS1L" "TI_R_P2"

"1546" 9.1387186762452e-16 1.05847693152999 0.606 0.427 3.05315452254676e-11 "PR3" "AFF4" "TI_R_P2"

"1547" 9.31554253280421e-16 1.02113394740663 0.589 0.419 3.11222960478456e-11 "PR3" "MAP1LC3A" "TI_R_P2"

"1548" 9.4744139803387e-16 1.04709624921065 0.513 0.348 3.16530696669136e-11 "PR3" "NIT1" "TI_R_P2"

"1549" 9.718026579136e-16 1.19882309439433 0.723 0.566 3.24669549982354e-11 "PR3" "POLR2K" "TI_R_P2"

"1550" 9.75643315926601e-16 1.2967096336992 0.444 0.26 3.25952675417918e-11 "PR3" "ANPEP" "TI_R_P2"

"1551" 1.33817867985414e-15 1.0919895876616 0.561 0.377 4.47072115152471e-11 "PR3" "EMC1" "TI_R_P2"

"1552" 1.3475348165654e-15 1.04552557285667 0.828 0.691 4.50197906866335e-11 "PR3" "BCAP31" "TI_R_P2"

"1553" 1.37105725877631e-15 3.67097641681412 0.778 0.656 4.58056519584576e-11 "PR3" "TMEM173" "TI_R_P2"

"1554" 1.37660107231557e-15 1.42623716849074 0.723 0.568 4.59908652249909e-11 "PR3" "TM7SF2" "TI_R_P2"

"1555" 1.56624848738323e-15 3.03718159066123 0.728 0.601 5.23267957149862e-11 "PR3" "NINJ2" "TI_R_P2"

"1556" 1.9006889350559e-15 2.41527413473914 0.632 0.466 6.35001166312827e-11 "PR3" "GOLGA3" "TI_R_P2"

"1557" 2.08268930382788e-15 3.86271000849157 0.74 0.616 6.95805669515855e-11 "PR3" "SYTL1" "TI_R_P2"

"1558" 2.12912880283766e-15 1.35911900474219 0.618 0.458 7.11320641740034e-11 "PR3" "SRP68" "TI_R_P2"

"1559" 2.24955819426164e-15 1.11178923051004 0.692 0.552 7.51554897120872e-11 "PR3" "WSB1" "TI_R_P2"

"1560" 2.36935096360096e-15 2.45065085346443 0.771 0.65 7.91576463429443e-11 "PR3" "TSPAN4" "TI_R_P2"

"1561" 2.43892042252159e-15 2.24681321396417 0.826 0.712 8.14818923960239e-11 "PR3" "FIS1" "TI_R_P2"

"1562" 2.44840856892123e-15 1.18791564926315 0.437 0.274 8.17988818790895e-11 "PR3" "BNIPL" "TI_R_P2"

"1563" 2.75065397095469e-15 1.42009261895558 0.635 0.475 9.18965985156252e-11 "PR3" "TSC2" "TI_R_P2"

"1564" 2.8149105713739e-15 3.457770815996 0.637 0.486 9.40433472790308e-11 "PR3" "TXLNA" "TI_R_P2"

"1565" 2.87674916975942e-15 1.24986996542144 0.652 0.49 9.61093130124925e-11 "PR3" "CXXC5" "TI_R_P2"

"1566" 3.53340979245489e-15 1.11278102029234 0.647 0.481 1.18047687756126e-10 "PR3" "ERI3" "TI_R_P2"

"1567" 3.72430756668325e-15 1.11004162396673 0.487 0.31 1.24425391495321e-10 "PR3" "WDR45B" "TI_R_P2"

"1568" 3.86636517265373e-15 1.0671207090304 0.508 0.326 1.29171394053189e-10 "PR3" "POGK" "TI_R_P2"

"1569" 4.84424951887868e-15 2.56667936452228 0.761 0.637 1.61841532176218e-10 "PR3" "ATP2A2" "TI_R_P2"

"1570" 5.04846717059147e-15 1.3991767474503 0.413 0.255 1.6866423970229e-10 "PR3" "CEACAM1" "TI_R_P2"

"1571" 5.13458706488023e-15 1.90477348503032 0.673 0.517 1.71541419250584e-10 "PR3" "TMEM63A" "TI_R_P2"

"1572" 5.60316447395411e-15 1.09544007649619 0.649 0.472 1.87196121910333e-10 "PR3" "SEPHS2" "TI_R_P2"

"1573" 6.12422280154824e-15 1.26142387318154 0.563 0.398 2.04604159576925e-10 "PR3" "MFN2" "TI_R_P2"

"1574" 6.40856261753864e-15 1.14159415687691 0.69 0.552 2.14103668489348e-10 "PR3" "NAPA" "TI_R_P2"

"1575" 6.78324946008271e-15 1.4139922244021 0.644 0.499 2.26621581211903e-10 "PR3" "LAMB2" "TI_R_P2"

"1576" 6.83774977306539e-15 1.50626372777698 0.749 0.595 2.28442382168342e-10 "PR3" "TMEM179B" "TI_R_P2"

"1577" 7.40614764510655e-15 2.84765132663478 0.532 0.368 2.47431986675365e-10 "PR3" "CXADR" "TI_R_P2"

"1578" 7.8181168499739e-15 1.02942614547635 0.551 0.372 2.61195465840778e-10 "PR3" "NR2F6" "TI_R_P2"

"1579" 7.96141880142241e-15 2.46686193611235 0.8 0.68 2.65983040736721e-10 "PR3" "HNRNPD" "TI_R_P2"

"1580" 8.51154539736173e-15 1.7495333413192 0.649 0.477 2.84362220180458e-10 "PR3" "PMVK" "TI_R_P2"

"1581" 9.37563589203215e-15 2.35868239520116 0.752 0.602 3.13230619516902e-10 "PR3" "RTN3" "TI_R_P2"

"1582" 1.00457438496035e-14 1.0477887331776 0.601 0.41 3.35618256271405e-10 "PR3" "PITRM1" "TI_R_P2"

"1583" 1.0681583812052e-14 1.37361122205559 0.644 0.487 3.56861033576844e-10 "PR3" "PYURF" "TI_R_P2"

"1584" 1.16770273363646e-14 1.05832238990627 0.685 0.525 3.90117806280605e-10 "PR3" "UBE2H" "TI_R_P2"

"1585" 1.20545092347104e-14 1.22656133437949 0.484 0.314 4.02729099022438e-10 "PR3" "NT5C3B" "TI_R_P2"

"1586" 1.23677166833206e-14 1.16043209225706 0.554 0.389 4.13193046673058e-10 "PR3" "CASC3" "TI_R_P2"

"1587" 1.23850532109612e-14 1.6837213508722 0.685 0.54 4.13772242725003e-10 "PR3" "SPCS3" "TI_R_P2"

"1588" 1.24137880633018e-14 1.28524330960319 0.625 0.444 4.14732245406851e-10 "PR3" "MAGOH" "TI_R_P2"

"1589" 1.26592532035949e-14 6.259036770267 0.687 0.533 4.22932990278902e-10 "PR3" "PARP14" "TI_R_P2"

"1590" 1.36803382178535e-14 1.07692343549195 0.549 0.392 4.57046419520267e-10 "PR3" "POGZ" "TI_R_P2"

"1591" 1.37639258414798e-14 2.1363490460193 0.768 0.608 4.59838998437998e-10 "PR3" "AKT1" "TI_R_P2"

"1592" 1.37922744714947e-14 1.22862441295257 0.563 0.392 4.60786097818166e-10 "PR3" "IPO9" "TI_R_P2"

"1593" 1.38851462972531e-14 1.2818300917555 0.599 0.431 4.63888852644929e-10 "PR3" "TLR2" "TI_R_P2"

"1594" 1.45425478101594e-14 1.02497342311015 0.368 0.206 4.85851979789616e-10 "PR3" "LRRK2" "TI_R_P2"

"1595" 1.46011147675714e-14 1.84185028923423 0.556 0.384 4.87808643269794e-10 "PR3" "HEXIM1" "TI_R_P2"

"1596" 1.50960936956588e-14 1.84537738259026 0.625 0.46 5.04345394278265e-10 "PR3" "NMRAL1" "TI_R_P2"

"1597" 1.54521354490067e-14 2.89924213828412 0.761 0.617 5.16240393215866e-10 "PR3" "TM9SF3" "TI_R_P2"

"1598" 1.65068264441656e-14 1.25565199920745 0.749 0.624 5.51476564673127e-10 "PR3" "COPA" "TI_R_P2"

"1599" 1.66651641092486e-14 1.67552637893889 0.735 0.611 5.56766467725886e-10 "PR3" "ARF3" "TI_R_P2"

"1600" 1.78723316413037e-14 1.98397028832618 0.63 0.49 5.97096727804316e-10 "PR3" "MRPL24" "TI_R_P2"

"1601" 1.81862560283791e-14 1.33857847807984 0.802 0.689 6.07584627652119e-10 "PR3" "RALY" "TI_R_P2"

"1602" 2.11519295693187e-14 1.10960173733232 0.721 0.616 7.06664814981368e-10 "PR3" "MDH2" "TI_R_P2"

"1603" 2.45237809006297e-14 1.00933047609657 0.632 0.474 8.19314996109137e-10 "PR3" "ATL3" "TI_R_P2"

"1604" 2.930923606179e-14 1.70950770826807 0.587 0.411 9.79192267588343e-10 "PR3" "LGALS8" "TI_R_P2"

"1605" 3.03129127810436e-14 1.19229259959039 0.537 0.379 1.01272410310188e-09 "PR3" "KCNK1" "TI_R_P2"

"1606" 3.05002473521534e-14 1.13204195828329 0.601 0.433 1.01898276378809e-09 "PR3" "NUDCD3" "TI_R_P2"

"1607" 3.0947798261546e-14 1.00250507914254 0.659 0.501 1.03393499211999e-09 "PR3" "LARS" "TI_R_P2"

"1608" 3.12184492401447e-14 2.21249445585658 0.671 0.509 1.04297717066399e-09 "PR3" "ECHDC1" "TI_R_P2"

"1609" 3.4166035117907e-14 1.1345204619403 0.558 0.384 1.14145306725416e-09 "PR3" "MYO1D" "TI_R_P2"

"1610" 3.49238161776505e-14 1.24356775137353 0.609 0.444 1.16676977467913e-09 "PR3" "GPR108" "TI_R_P2"

"1611" 3.66167594122793e-14 2.86031804723637 0.704 0.582 1.22332931520484e-09 "PR3" "DDOST" "TI_R_P2"

"1612" 3.70575640046538e-14 6.31175223313511 0.862 0.705 1.23805615583148e-09 "PR3" "IFI6" "TI_R_P2"

"1613" 3.97409990509917e-14 1.07049402379748 0.668 0.539 1.32770703729458e-09 "PR3" "C16orf72" "TI_R_P2"

"1614" 4.0463762995653e-14 5.91401761131975 0.477 0.309 1.35185385792177e-09 "PR3" "COMP" "TI_R_P2"

"1615" 4.3625112512896e-14 1.05169930254 0.656 0.496 1.45747138394334e-09 "PR3" "DHX9" "TI_R_P2"

"1616" 4.42748207362988e-14 2.2221128082805 0.764 0.624 1.47917748597901e-09 "PR3" "ATP6AP1" "TI_R_P2"

"1617" 4.55260388660802e-14 1.0038795272514 0.465 0.297 1.52097943247687e-09 "PR3" "SKIV2L" "TI_R_P2"

"1618" 4.60045760069508e-14 1.82321066259755 0.831 0.706 1.53696687981622e-09 "PR3" "PPP1CA" "TI_R_P2"

"1619" 4.68453255097458e-14 1.34808634703349 0.85 0.737 1.5650554799551e-09 "PR3" "ARL6IP4" "TI_R_P2"

"1620" 4.99977047438447e-14 1.52915102765519 0.532 0.363 1.67037331778711e-09 "PR3" "UBE2G2" "TI_R_P2"

"1621" 5.10735991742626e-14 2.44309077695089 0.749 0.632 1.70631787481294e-09 "PR3" "PPP4C" "TI_R_P2"

"1622" 5.16077110026028e-14 3.85841587032228 0.718 0.584 1.72416201688596e-09 "PR3" "ATP5MC1" "TI_R_P2"

"1623" 5.23972984745746e-14 1.01948510690453 0.695 0.529 1.75054134473706e-09 "PR3" "HNRNPAB" "TI_R_P2"

"1624" 5.24241672427367e-14 1.71911219082837 0.675 0.533 1.75143900341259e-09 "PR3" "MRPL57" "TI_R_P2"

"1625" 5.61694641445185e-14 1.03168956950264 0.623 0.442 1.87656562760422e-09 "PR3" "MRPS7" "TI_R_P2"

"1626" 5.66408564667165e-14 1.77782863375417 0.692 0.537 1.89231437369653e-09 "PR3" "SHC1" "TI_R_P2"

"1627" 5.72376712569837e-14 1.31075313844271 0.714 0.595 1.91225335902457e-09 "PR3" "RNF187" "TI_R_P2"

"1628" 6.03183527551784e-14 1.35294546118677 0.578 0.424 2.01517584719775e-09 "PR3" "ENTPD6" "TI_R_P2"

"1629" 6.09714883932037e-14 1.02936570747725 0.656 0.487 2.03699645572854e-09 "PR3" "CD302" "TI_R_P2"

"1630" 7.55635800355028e-14 1.56693970754939 0.673 0.53 2.52450364540611e-09 "PR3" "TBC1D9B" "TI_R_P2"

"1631" 7.72290575674071e-14 1.18797908349013 0.537 0.388 2.5801455842695e-09 "PR3" "NT5C" "TI_R_P2"

"1632" 7.92563386413463e-14 1.74118594659121 0.749 0.613 2.64787501766874e-09 "PR3" "PRDX4" "TI_R_P2"

"1633" 8.2277122644088e-14 2.1749084191824 0.53 0.375 2.74879639041634e-09 "PR3" "MAPK8IP3" "TI_R_P2"

"1634" 8.32344428868019e-14 1.55144323281791 0.499 0.329 2.78077950240517e-09 "PR3" "AP006284.1" "TI_R_P2"

"1635" 9.31381120770725e-14 1.66107526035243 0.599 0.425 3.11165118638291e-09 "PR3" "RBPMS" "TI_R_P2"

"1636" 9.39024401107778e-14 1.49707670176986 0.616 0.464 3.13718662166098e-09 "PR3" "SLC9A3R2" "TI_R_P2"

"1637" 9.44898717787731e-14 1.01965265965494 0.768 0.626 3.15681212625703e-09 "PR3" "MBNL1" "TI_R_P2"

"1638" 9.99993450955322e-14 2.56488347521534 0.582 0.407 3.34087812029663e-09 "PR3" "COA6" "TI_R_P2"

"1639" 1.01859113775788e-13 1.49296816371316 0.73 0.608 3.40301113213529e-09 "PR3" "ELOC" "TI_R_P2"

"1640" 1.04644046641263e-13 2.65091859586223 0.702 0.557 3.49605295423794e-09 "PR3" "MAZ" "TI_R_P2"

"1641" 1.07107969880555e-13 2.21068417977518 0.482 0.321 3.57837016573947e-09 "PR3" "TMEM175" "TI_R_P2"

"1642" 1.13880450752546e-13 1.1147552857437 0.671 0.535 3.8046319791918e-09 "PR3" "PFDN2" "TI_R_P2"

"1643" 1.17678194328788e-13 1.41632942596625 0.568 0.406 3.93151079433047e-09 "PR3" "PRPF3" "TI_R_P2"

"1644" 1.20760071914611e-13 1.31863544459178 0.745 0.611 4.03447324259523e-09 "PR3" "PSMB5" "TI_R_P2"

"1645" 1.26393350742451e-13 1.32602251691067 0.714 0.574 4.22267545495455e-09 "PR3" "MFSD10" "TI_R_P2"

"1646" 1.32224127340504e-13 2.03438478698008 0.587 0.452 4.4174758703189e-09 "PR3" "B4GALT2" "TI_R_P2"

"1647" 1.34533172930346e-13 1.17815610329827 0.747 0.605 4.49461877442992e-09 "PR3" "MAPKAPK2" "TI_R_P2"

"1648" 1.38850506742828e-13 1.76686045131089 0.647 0.5 4.63885657977116e-09 "PR3" "TNKS1BP1" "TI_R_P2"

"1649" 1.71488093813667e-13 1.01154765109009 0.663 0.508 5.72924572622079e-09 "PR3" "CNIH1" "TI_R_P2"

"1650" 1.77562231015374e-13 1.97327673702003 0.53 0.372 5.93217657599264e-09 "PR3" "KDM3B" "TI_R_P2"

"1651" 1.78158050053789e-13 1.07945252029726 0.673 0.506 5.95208229424703e-09 "PR3" "PDS5A" "TI_R_P2"

"1652" 1.86070878089385e-13 1.38194383767718 0.742 0.598 6.21644196608825e-09 "PR3" "TMEM248" "TI_R_P2"

"1653" 1.89439374581748e-13 1.23040652078094 0.833 0.723 6.32898006540161e-09 "PR3" "DDT" "TI_R_P2"

"1654" 1.94798141569881e-13 1.03139116985842 0.692 0.529 6.50801111170814e-09 "PR3" "FDPS" "TI_R_P2"

"1655" 2.21271517387815e-13 1.34384342859651 0.649 0.476 7.3924601244095e-09 "PR3" "PUF60" "TI_R_P2"

"1656" 2.39658236062777e-13 1.67507312382965 0.752 0.611 8.00674200862132e-09 "PR3" "C19orf53" "TI_R_P2"

"1657" 2.75460724297268e-13 1.77114287858285 0.597 0.447 9.20286733804744e-09 "PR3" "MFGE8" "TI_R_P2"

"1658" 2.85086343851458e-13 1.05888663607447 0.52 0.364 9.52444966173337e-09 "PR3" "ROGDI" "TI_R_P2"

"1659" 2.93103124057847e-13 3.60593306320013 0.623 0.479 9.7922822716486e-09 "PR3" "LENG8" "TI_R_P2"

"1660" 2.98625866840918e-13 1.55252541537425 0.513 0.343 9.97679158528823e-09 "PR3" "PHYKPL" "TI_R_P2"

"1661" 3.04712758652762e-13 1.3805679925313 0.659 0.534 1.01801485538301e-08 "PR3" "APMAP" "TI_R_P2"

"1662" 3.48043262654627e-13 1.19916117768736 0.57 0.413 1.16277773620284e-08 "PR3" "FBRS" "TI_R_P2"

"1663" 3.67526663183991e-13 1.04215483199485 0.48 0.32 1.2278698290314e-08 "PR3" "PRKD2" "TI_R_P2"

"1664" 4.28845514561193e-13 1.09881625661806 0.835 0.723 1.43272997959749e-08 "PR3" "SEPT2" "TI_R_P2"

"1665" 4.35607165973041e-13 1.05048881695222 0.439 0.273 1.45531998079933e-08 "PR3" "PHLDB1" "TI_R_P2"

"1666" 4.55108591662575e-13 1.69665612666241 0.427 0.275 1.5204722938855e-08 "PR3" "TMEM92" "TI_R_P2"

"1667" 4.56476739651839e-13 1.22925921580015 0.792 0.667 1.52504313950283e-08 "PR3" "EIF4H" "TI_R_P2"

"1668" 4.58727080804834e-13 3.64908583648336 0.821 0.717 1.53256130426087e-08 "PR3" "MRFAP1" "TI_R_P2"

"1669" 5.1404590941134e-13 1.92518384026065 0.721 0.59 1.71737597875235e-08 "PR3" "PSMA3" "TI_R_P2"

"1670" 5.44939956686404e-13 1.71456069606565 0.623 0.473 1.82058990129361e-08 "PR3" "EIF4A1" "TI_R_P2"

"1671" 5.49173800163985e-13 1.29224854221037 0.711 0.568 1.83473474896786e-08 "PR3" "GTF2H5" "TI_R_P2"

"1672" 5.50314307905139e-13 1.6130863618457 0.749 0.631 1.83854507128028e-08 "PR3" "C11orf58" "TI_R_P2"

"1673" 5.58259083266604e-13 1.61125305876522 0.804 0.695 1.8650877712854e-08 "PR3" "LAMTOR4" "TI_R_P2"

"1674" 5.72446605107926e-13 1.08552715813579 0.609 0.442 1.91248686300507e-08 "PR3" "INAVA" "TI_R_P2"

"1675" 5.88596324770592e-13 2.38839159646919 0.745 0.634 1.96644146142607e-08 "PR3" "UQCRC2" "TI_R_P2"

"1676" 6.08862761057951e-13 1.98764719362974 0.563 0.407 2.03414959841851e-08 "PR3" "MED28" "TI_R_P2"

"1677" 6.30215210490577e-13 1.47358699601601 0.623 0.443 2.10548599672797e-08 "PR3" "CAPN12" "TI_R_P2"

"1678" 6.47362940481163e-13 1.05796924463875 0.463 0.315 2.16277484785352e-08 "PR3" "CREBZF" "TI_R_P2"

"1679" 6.68459779177607e-13 1.12176927803797 0.716 0.577 2.23325727625447e-08 "PR3" "ERH" "TI_R_P2"

"1680" 7.91626248031787e-13 1.41057618113305 0.761 0.651 2.6447441320494e-08 "PR3" "BLVRA" "TI_R_P2"

"1681" 8.14267178993894e-13 1.41319675085199 0.592 0.441 2.7203852183007e-08 "PR3" "SLC12A7" "TI_R_P2"

"1682" 8.4944911591193e-13 1.00809168047033 0.537 0.375 2.83792455135017e-08 "PR3" "D2HGDH" "TI_R_P2"

"1683" 8.7201319064744e-13 1.7933704842838 0.749 0.63 2.91330886863403e-08 "PR3" "UBE2D2" "TI_R_P2"

"1684" 9.35180647646269e-13 6.49546704480793 0.716 0.582 3.12434502572142e-08 "PR3" "MEAF6" "TI_R_P2"

"1685" 1.01467490428716e-12 1.15159239335099 0.737 0.604 3.38992738773296e-08 "PR3" "GPAA1" "TI_R_P2"

"1686" 1.10096551058459e-12 1.03995498301917 0.726 0.602 3.67821567431204e-08 "PR3" "VPS51" "TI_R_P2"

"1687" 1.16076588041587e-12 1.04898358002389 0.372 0.226 3.87800272988137e-08 "PR3" "CDK5RAP1" "TI_R_P2"

"1688" 1.21774104889022e-12 1.09299230275041 0.566 0.424 4.06835107023733e-08 "PR3" "DAZAP1" "TI_R_P2"

"1689" 1.25650857819805e-12 1.53986345039167 0.597 0.437 4.19786950890185e-08 "PR3" "RPA2" "TI_R_P2"

"1690" 1.25968070964968e-12 3.48928274735496 0.831 0.729 4.20846728286861e-08 "PR3" "EIF3K" "TI_R_P2"

"1691" 1.32560146543383e-12 1.14770517695657 0.623 0.482 4.42870193586787e-08 "PR3" "GORASP2" "TI_R_P2"

"1692" 1.40054003896021e-12 1.05756223108588 0.549 0.38 4.67906421616215e-08 "PR3" "HPCAL1" "TI_R_P2"

"1693" 1.41151553906402e-12 1.28856123177039 0.661 0.506 4.715732264459e-08 "PR3" "MRPS15" "TI_R_P2"

"1694" 1.43557958654463e-12 1.45003164545476 0.621 0.463 4.79612784068697e-08 "PR3" "AAMP" "TI_R_P2"

"1695" 1.67241858765362e-12 1.29314587023994 0.625 0.478 5.58738325949199e-08 "PR3" "SPSB3" "TI_R_P2"

"1696" 1.67721491478703e-12 2.39188355192843 0.659 0.513 5.60340730881198e-08 "PR3" "MAP7D1" "TI_R_P2"

"1697" 1.72274472750567e-12 1.00564545796571 0.453 0.296 5.7555178601237e-08 "PR3" "CPSF1" "TI_R_P2"

"1698" 1.75267529824131e-12 1.0764145932283 0.654 0.505 5.85551290389438e-08 "PR3" "FAM3C" "TI_R_P2"

"1699" 1.80866086043315e-12 1.71027274862528 0.749 0.626 6.04255506862111e-08 "PR3" "TOR1AIP2" "TI_R_P2"

"1700" 1.87755137960013e-12 3.01832393325195 0.57 0.418 6.27271140410608e-08 "PR3" "ERLEC1" "TI_R_P2"

"1701" 1.89275821850686e-12 1.05209608549891 0.589 0.449 6.32351593220956e-08 "PR3" "CYP4B1" "TI_R_P2"

"1702" 1.91639699519083e-12 1.32948908257138 0.637 0.512 6.40249072123305e-08 "PR3" "MFSD4A" "TI_R_P2"

"1703" 1.92375136023331e-12 1.36252512722112 0.652 0.501 6.42706091940346e-08 "PR3" "G3BP1" "TI_R_P2"

"1704" 1.94008441297943e-12 1.16175664949375 0.616 0.461 6.48162801532298e-08 "PR3" "SCAND1" "TI_R_P2"

"1705" 1.94299534457006e-12 1.31936489562143 0.723 0.617 6.4913531466741e-08 "PR3" "EIF4G1" "TI_R_P2"

"1706" 2.15068524970438e-12 1.04639561452084 0.695 0.574 7.18522435073736e-08 "PR3" "MRPS34" "TI_R_P2"

"1707" 2.23398408189469e-12 1.21218090158055 0.599 0.469 7.46351741920195e-08 "PR3" "ECI1" "TI_R_P2"

"1708" 2.4346658576259e-12 1.34033447739649 0.647 0.501 8.13397516374236e-08 "PR3" "RUNX1" "TI_R_P2"

"1709" 2.57568172754098e-12 1.67355925141305 0.585 0.431 8.60509508354167e-08 "PR3" "SFN" "TI_R_P2"

"1710" 2.59230361661526e-12 1.76326252502176 0.477 0.328 8.66062715274991e-08 "PR3" "MLST8" "TI_R_P2"

"1711" 2.9355322395663e-12 1.47576350318491 0.652 0.506 9.80731965916704e-08 "PR3" "APP" "TI_R_P2"

"1712" 3.19188464362887e-12 1.59621615472259 0.728 0.6 1.06637674058997e-07 "PR3" "LAMTOR2" "TI_R_P2"

"1713" 3.44803030273129e-12 1.08938914577228 0.597 0.458 1.1519524438395e-07 "PR3" "TMEM8A" "TI_R_P2"

"1714" 3.67205554547036e-12 1.07271885469923 0.599 0.453 1.22679703718619e-07 "PR3" "TSPAN6" "TI_R_P2"

"1715" 3.8595345445742e-12 1.85881602798562 0.802 0.677 1.2894318959968e-07 "PR3" "BLOC1S1" "TI_R_P2"

"1716" 3.86042458446081e-12 1.44273986993771 0.585 0.432 1.28972924942251e-07 "PR3" "PEX19" "TI_R_P2"

"1717" 4.01395134936202e-12 3.49827952437563 0.74 0.624 1.34102100630836e-07 "PR3" "CCT5" "TI_R_P2"

"1718" 4.10151194603091e-12 1.42299762302024 0.587 0.441 1.37027412604947e-07 "PR3" "WDR33" "TI_R_P2"

"1719" 4.33791091552219e-12 1.26290118826296 0.659 0.532 1.44925265776681e-07 "PR3" "RNPEP" "TI_R_P2"

"1720" 4.40750114266932e-12 1.20363896935426 0.599 0.456 1.47250205675439e-07 "PR3" "CYHR1" "TI_R_P2"

"1721" 4.83654523700223e-12 1.57946902035707 0.716 0.61 1.61584139823007e-07 "PR3" "APEX1" "TI_R_P2"

"1722" 4.93963168875152e-12 1.17980199888115 0.656 0.538 1.650281550895e-07 "PR3" "SMIM26" "TI_R_P2"

"1723" 5.03704007791113e-12 2.27891884541944 0.525 0.373 1.68282471962933e-07 "PR3" "USP53" "TI_R_P2"

"1724" 5.19113061061907e-12 1.47858361514931 0.504 0.362 1.73430482570173e-07 "PR3" "NFATC4" "TI_R_P2"

"1725" 5.20321079558958e-12 1.17266620502983 0.547 0.394 1.73834069469852e-07 "PR3" "GALNT3" "TI_R_P2"

"1726" 5.47371862056294e-12 1.03129190545333 0.661 0.515 1.82871465394387e-07 "PR3" "IPO7" "TI_R_P2"

"1727" 5.83094542409077e-12 1.15086784096706 0.477 0.33 1.94806055673448e-07 "PR3" "SMPD4" "TI_R_P2"

"1728" 6.08827916801786e-12 1.87368526244382 0.792 0.664 2.03403318724309e-07 "PR3" "CTNNB1" "TI_R_P2"

"1729" 6.62186451671777e-12 1.23648153791453 0.623 0.491 2.21229871639024e-07 "PR3" "BCL7C" "TI_R_P2"

"1730" 7.78673645369281e-12 2.87768760465323 0.857 0.74 2.60147078181423e-07 "PR3" "EIF4A2" "TI_R_P2"

"1731" 8.23627382204567e-12 1.07558335600745 0.644 0.506 2.75165672120724e-07 "PR3" "LRRC41" "TI_R_P2"

"1732" 8.42827787217165e-12 1.32227482600164 0.609 0.45 2.81580335431383e-07 "PR3" "UBALD2" "TI_R_P2"

"1733" 8.55752924346001e-12 1.22900970939323 0.647 0.52 2.85898494494756e-07 "PR3" "CCND3" "TI_R_P2"

"1734" 8.74571322626199e-12 2.13205767401839 0.74 0.626 2.92185533176187e-07 "PR3" "VPS28" "TI_R_P2"

"1735" 9.24756826660391e-12 1.28532152935726 0.663 0.524 3.0895200821897e-07 "PR3" "TNS1" "TI_R_P2"

"1736" 1.10353630147766e-11 1.1813300262522 0.558 0.408 3.6868044296067e-07 "PR3" "IP6K2" "TI_R_P2"

"1737" 1.12389016157385e-11 1.77888928656261 0.652 0.52 3.75480464080207e-07 "PR3" "POLR2G" "TI_R_P2"

"1738" 1.15591062846245e-11 2.70369320868647 0.828 0.716 3.86178181863018e-07 "PR3" "BLVRB" "TI_R_P2"

"1739" 1.28271226407078e-11 1.12991501076583 0.723 0.608 4.28541340303408e-07 "PR3" "UBE2I" "TI_R_P2"

"1740" 1.3030038916859e-11 1.25023025864733 0.57 0.423 4.35320570173341e-07 "PR3" "TESC" "TI_R_P2"

"1741" 1.32955311912554e-11 1.31319090602635 0.628 0.488 4.44190401568651e-07 "PR3" "ERAP1" "TI_R_P2"

"1742" 1.38962892725441e-11 1.7537099906493 0.492 0.347 4.64261128306427e-07 "PR3" "MIR29B2CHG" "TI_R_P2"

"1743" 1.4257202987965e-11 1.00162692663196 0.649 0.511 4.76318894624921e-07 "PR3" "MRPL55" "TI_R_P2"

"1744" 1.46569822356543e-11 1.11417851715779 0.621 0.484 4.89675119510973e-07 "PR3" "YY1" "TI_R_P2"

"1745" 1.57905475348127e-11 1.05036903500875 0.494 0.35 5.27546402590557e-07 "PR3" "CSKMT" "TI_R_P2"

"1746" 1.60274894177766e-11 2.72604214488395 0.64 0.496 5.35462393958497e-07 "PR3" "PEF1" "TI_R_P2"

"1747" 1.61650693235619e-11 1.28316246768754 0.616 0.476 5.4005880103088e-07 "PR3" "SMARCA4" "TI_R_P2"

"1748" 1.62323352284401e-11 1.61751826917734 0.618 0.459 5.42306087646955e-07 "PR3" "TRIM47" "TI_R_P2"

"1749" 1.64212332235335e-11 1.16979059218216 0.654 0.504 5.48616980765032e-07 "PR3" "LSM3" "TI_R_P2"

"1750" 1.87526042074865e-11 1.15409478554215 0.57 0.439 6.26505753967917e-07 "PR3" "ESYT2" "TI_R_P2"

"1751" 1.88145538720677e-11 1.21567845009405 0.558 0.405 6.28575430311911e-07 "PR3" "SRA1" "TI_R_P2"

"1752" 1.91647829681893e-11 1.07850774979674 0.64 0.515 6.40276234184236e-07 "PR3" "PIM3" "TI_R_P2"

"1753" 2.29969966292673e-11 1.24517809741212 0.506 0.365 7.6830666038719e-07 "PR3" "PSMG3" "TI_R_P2"

"1754" 2.50914663940717e-11 1.03486609760619 0.826 0.704 8.38280800759541e-07 "PR3" "ATP5PF" "TI_R_P2"

"1755" 2.54831340253548e-11 1.1508932371807 0.697 0.593 8.51366024653077e-07 "PR3" "TMEM230" "TI_R_P2"

"1756" 2.6448649563898e-11 1.51245018270459 0.542 0.402 8.8362293328027e-07 "PR3" "CPD" "TI_R_P2"

"1757" 2.89760876179443e-11 2.07939717602463 0.692 0.574 9.680621112279e-07 "PR3" "BAD" "TI_R_P2"

"1758" 2.90848622260384e-11 1.32587885351551 0.721 0.596 9.71696162109719e-07 "PR3" "DDX24" "TI_R_P2"

"1759" 2.97609143709407e-11 1.86478542102368 0.635 0.491 9.94282388218758e-07 "PR3" "CCPG1" "TI_R_P2"

"1760" 3.14262663203935e-11 1.92338485382637 0.661 0.528 1.04992013149803e-06 "PR3" "PFKL" "TI_R_P2"

"1761" 3.29880706060287e-11 1.22961461219062 0.647 0.5 1.10209845087681e-06 "PR3" "PDAP1" "TI_R_P2"

"1762" 3.63836496308675e-11 3.31891818630893 0.21 0.102 1.21554135051765e-06 "PR3" "SPINK1" "TI_R_P2"

"1763" 3.7675779618659e-11 1.09221123002885 0.773 0.65 1.25871012127978e-06 "PR3" "PHB2" "TI_R_P2"

"1764" 4.35637203602741e-11 4.2366718153477 0.73 0.621 1.4554203335164e-06 "PR3" "STAT6" "TI_R_P2"

"1765" 4.79103576461832e-11 1.30175587031268 0.558 0.419 1.60063713860133e-06 "PR3" "ZBTB7B" "TI_R_P2"

"1766" 5.1577041056578e-11 1.44068407953916 0.618 0.492 1.72313736465921e-06 "PR3" "RBM5" "TI_R_P2"

"1767" 5.36547781004597e-11 1.14597927144775 0.537 0.378 1.79255248155826e-06 "PR3" "FAM3B" "TI_R_P2"

"1768" 5.58951264630184e-11 1.10438004780095 0.652 0.513 1.86740028000298e-06 "PR3" "SLC44A2" "TI_R_P2"

"1769" 5.96213307484203e-11 1.04852985978521 0.582 0.452 1.99188903897397e-06 "PR3" "MIEN1" "TI_R_P2"

"1770" 5.98960247791625e-11 1.44831755725966 0.544 0.412 2.00106629184704e-06 "PR3" "MCCC2" "TI_R_P2"

"1771" 6.10453458941737e-11 1.31741094846608 0.535 0.386 2.03946396097845e-06 "PR3" "IDH3B" "TI_R_P2"

"1772" 6.27103729874404e-11 1.46452949763062 0.551 0.404 2.0950908511374e-06 "PR3" "TPM2" "TI_R_P2"

"1773" 6.45968278678668e-11 1.00755873847487 0.57 0.429 2.15811542223756e-06 "PR3" "CDKN1B" "TI_R_P2"

"1774" 6.66814543749438e-11 1.71493040895136 0.353 0.212 2.2277607092125e-06 "PR3" "SSH2" "TI_R_P2"

"1775" 6.84156854416175e-11 1.01677402693259 0.43 0.281 2.285699634919e-06 "PR3" "RAB9A" "TI_R_P2"

"1776" 7.88775366007657e-11 1.16769679639242 0.635 0.493 2.63521962029498e-06 "PR3" "UBR4" "TI_R_P2"

"1777" 8.61697614790049e-11 1.17365830647757 0.597 0.468 2.87884556125208e-06 "PR3" "RAD21" "TI_R_P2"

"1778" 9.04654610745022e-11 1.20245082509837 0.516 0.369 3.02236058903804e-06 "PR3" "SNX9" "TI_R_P2"

"1779" 1.02154702618331e-10 1.02574385750361 0.706 0.594 3.41288645977583e-06 "PR3" "SNRPB" "TI_R_P2"

"1780" 1.08348038250552e-10 1.42964267080056 0.771 0.66 3.6197996099127e-06 "PR3" "XRCC6" "TI_R_P2"

"1781" 1.18930017774249e-10 1.16578460222496 0.58 0.447 3.9733329638199e-06 "PR3" "UHMK1" "TI_R_P2"

"1782" 1.2061423921868e-10 2.15695901964525 0.711 0.573 4.02960111805688e-06 "PR3" "IDS" "TI_R_P2"

"1783" 1.43002052544944e-10 1.04855080200848 0.666 0.542 4.77755557347403e-06 "PR3" "WDR13" "TI_R_P2"

"1784" 1.433723796454e-10 1.29107788245373 0.673 0.556 4.78992783157318e-06 "PR3" "CNOT1" "TI_R_P2"

"1785" 1.44097321288291e-10 1.37382826925853 0.525 0.396 4.81414740692051e-06 "PR3" "ITFG1" "TI_R_P2"

"1786" 1.58780810342875e-10 1.44355660416945 0.492 0.345 5.30470809274512e-06 "PR3" "RABGGTB" "TI_R_P2"

"1787" 1.65261981208004e-10 1.16043361833661 0.346 0.216 5.52123753017822e-06 "PR3" "CAPN10" "TI_R_P2"

"1788" 1.93907870867067e-10 1.00620654647501 0.592 0.456 6.47826805779783e-06 "PR3" "TRADD" "TI_R_P2"

"1789" 1.93939009684801e-10 2.72741039080955 0.683 0.556 6.47930837455951e-06 "PR3" "SZRD1" "TI_R_P2"

"1790" 1.99616963302035e-10 2.44082092129797 0.69 0.585 6.66900312695769e-06 "PR3" "PPP2R1A" "TI_R_P2"

"1791" 2.00729587094403e-10 2.23196264505452 0.652 0.514 6.70617477523691e-06 "PR3" "EPAS1" "TI_R_P2"

"1792" 2.08859892852206e-10 1.11337679863195 0.327 0.196 6.97780016029934e-06 "PR3" "NUDT19" "TI_R_P2"

"1793" 2.09003119302247e-10 1.98899461340177 0.68 0.531 6.98258521276876e-06 "PR3" "COL6A1" "TI_R_P2"

"1794" 2.20330963881029e-10 1.02869411006683 0.444 0.305 7.36103717230129e-06 "PR3" "KHDC4" "TI_R_P2"

"1795" 2.40266089471905e-10 1.5637476934507 0.578 0.43 8.02704978316686e-06 "PR3" "KLC1" "TI_R_P2"

"1796" 2.83021530762339e-10 3.884491145333 0.687 0.549 9.45546632123898e-06 "PR3" "TNFRSF12A" "TI_R_P2"

"1797" 2.95769586294945e-10 1.53751767161138 0.649 0.53 9.88136610852782e-06 "PR3" "THRAP3" "TI_R_P2"

"1798" 2.97453561443271e-10 1.20708495462046 0.554 0.409 9.93762603425825e-06 "PR3" "SCOC" "TI_R_P2"

"1799" 3.01551366213584e-10 2.69124893999726 0.685 0.543 1.00745295938296e-05 "PR3" "PRRC2C" "TI_R_P2"

"1800" 3.02511858985219e-10 1.46733161925286 0.57 0.435 1.01066186968372e-05 "PR3" "MR1" "TI_R_P2"

"1801" 3.05963339811111e-10 1.08062715805759 0.563 0.433 1.02219292197494e-05 "PR3" "MAF1" "TI_R_P2"

"1802" 3.09974943861651e-10 1.08472413825785 0.609 0.465 1.03559528994739e-05 "PR3" "MYO1C" "TI_R_P2"

"1803" 3.10877259700213e-10 1.45390867889375 0.649 0.538 1.03860983693244e-05 "PR3" "TRAPPC3" "TI_R_P2"

"1804" 3.11455536075736e-10 1.19572098713229 0.685 0.561 1.04054180047543e-05 "PR3" "ENY2" "TI_R_P2"

"1805" 3.34130116957006e-10 1.52287515075409 0.582 0.44 1.11629530774166e-05 "PR3" "CTNNBIP1" "TI_R_P2"

"1806" 3.64918389615195e-10 9.86414306993673 0.52 0.378 1.2191558478654e-05 "PR3" "PRELP" "TI_R_P2"

"1807" 3.97644033949341e-10 1.04120815612234 0.623 0.503 1.32848895302135e-05 "PR3" "SELENON" "TI_R_P2"

"1808" 4.12466307725499e-10 1.05478710351355 0.492 0.35 1.37800868748012e-05 "PR3" "EGLN1" "TI_R_P2"

"1809" 4.3299704297008e-10 1.42108586954571 0.473 0.335 1.44659982085874e-05 "PR3" "SMUG1" "TI_R_P2"

"1810" 5.19536292482942e-10 1.03776675938942 0.461 0.322 1.73571879955626e-05 "PR3" "NDUFV3" "TI_R_P2"

"1811" 5.51880397627062e-10 1.05719242293029 0.57 0.439 1.84377722043225e-05 "PR3" "RAB18" "TI_R_P2"

"1812" 5.66459325332689e-10 1.12778056017922 0.527 0.392 1.89248396000398e-05 "PR3" "TTC17" "TI_R_P2"

"1813" 6.19950826296531e-10 1.04860291132762 0.57 0.442 2.07119371557408e-05 "PR3" "PUM2" "TI_R_P2"

"1814" 6.82463020067362e-10 1.00509967899485 0.358 0.231 2.28004070374305e-05 "PR3" "PDIK1L" "TI_R_P2"

"1815" 7.97005239603719e-10 1.4786164856636 0.757 0.65 2.66271480499207e-05 "PR3" "SPCS1" "TI_R_P2"

"1816" 8.59690657495606e-10 1.12598653538073 0.64 0.519 2.87214051762707e-05 "PR3" "RAB11FIP1" "TI_R_P2"

"1817" 8.63802019469689e-10 1.17144880487553 0.549 0.416 2.88587616684628e-05 "PR3" "RAB4A" "TI_R_P2"

"1818" 8.7322695881023e-10 1.07188737820977 0.733 0.62 2.9173639466891e-05 "PR3" "TMEM14C" "TI_R_P2"

"1819" 1.03525476455458e-09 1.01481333741763 0.47 0.336 3.45868264290038e-05 "PR3" "RNF115" "TI_R_P2"

"1820" 1.2687477775312e-09 1.34219999133289 0.542 0.417 4.23875944995398e-05 "PR3" "YWHAG" "TI_R_P2"

"1821" 1.45485376722055e-09 1.11771717833872 0.652 0.533 4.86052095090714e-05 "PR3" "TMEM109" "TI_R_P2"

"1822" 1.57965749980379e-09 1.31552661763297 0.444 0.311 5.27747774109447e-05 "PR3" "SPSB1" "TI_R_P2"

"1823" 1.65384011686505e-09 1.67409305081432 0.611 0.492 5.52531444643443e-05 "PR3" "SNX5" "TI_R_P2"

"1824" 1.76346129994855e-09 1.05002885826879 0.439 0.301 5.89154785699809e-05 "PR3" "PLXNB1" "TI_R_P2"

"1825" 1.78669630082958e-09 2.24760498040714 0.838 0.728 5.96917367144155e-05 "PR3" "RNH1" "TI_R_P2"

"1826" 1.89818026880335e-09 2.13807340871225 0.539 0.409 6.34163046004512e-05 "PR3" "ST5" "TI_R_P2"

"1827" 1.96229664063087e-09 4.01120148296075 0.671 0.548 6.55583684668366e-05 "PR3" "RERE" "TI_R_P2"

"1828" 2.20510891845105e-09 1.83293460408288 0.563 0.426 7.36704838565313e-05 "PR3" "BTBD2" "TI_R_P2"

"1829" 2.59687398008801e-09 1.61169342160478 0.635 0.517 8.67589628007604e-05 "PR3" "DDB1" "TI_R_P2"

"1830" 2.94746520853947e-09 1.23820747028139 0.678 0.573 9.84718651520951e-05 "PR3" "NDUFB8" "TI_R_P2"

"1831" 3.10698369989313e-09 1.09741719964107 0.527 0.413 0.00010380121842973 "PR3" "ZNF593" "TI_R_P2"

"1832" 3.18891965427254e-09 1.12283745591899 0.508 0.387 0.000106538616729591 "PR3" "MRPL43" "TI_R_P2"

"1833" 3.23128853387701e-09 1.0203177874009 0.344 0.224 0.000107954118628297 "PR3" "AL162511.1" "TI_R_P2"

"1834" 3.92277437732449e-09 1.01092812147974 0.456 0.324 0.000131055969172034 "PR3" "ARL2BP" "TI_R_P2"

"1835" 4.10083927783028e-09 2.19641943852842 0.647 0.533 0.000137004939433032 "PR3" "C1orf122" "TI_R_P2"

"1836" 4.44541529420772e-09 1.19152260983259 0.609 0.488 0.000148516879564186 "PR3" "IMPDH2" "TI_R_P2"

"1837" 4.89443170721092e-09 1.03007320936336 0.329 0.213 0.00016351806890621 "PR3" "ARHGEF28" "TI_R_P2"

"1838" 5.03595922807904e-09 1.02292849928371 0.52 0.389 0.000168246361850893 "PR3" "TMEM126B" "TI_R_P2"

"1839" 5.17286062717169e-09 1.9073508249024 0.578 0.438 0.000172820100693179 "PR3" "PPP2CB" "TI_R_P2"

"1840" 5.38266357844812e-09 1.05399719397375 0.685 0.537 0.000179829407492373 "PR3" "COL6A2" "TI_R_P2"

"1841" 5.57185469653523e-09 1.20171881171569 0.642 0.514 0.000186150093556545 "PR3" "SEL1L3" "TI_R_P2"

"1842" 6.29283824484771e-09 1.24581782887245 0.549 0.424 0.000210237432922117 "PR3" "BAIAP2L1" "TI_R_P2"

"1843" 6.53919081482389e-09 3.65729571737415 0.785 0.68 0.000218467825932451 "PR3" "KRTCAP2" "TI_R_P2"

"1844" 7.52528730487231e-09 1.30619784510024 0.621 0.509 0.000251412323568479 "PR3" "TMEM147" "TI_R_P2"

"1845" 7.68335250885033e-09 1.0315420173397 0.52 0.385 0.000256693123968181 "PR3" "DECR2" "TI_R_P2"

"1846" 9.49695108944658e-09 2.55235288348245 0.341 0.217 0.000317283638947321 "PR3" "RIF1" "TI_R_P2"

"1847" 1.04672707129893e-08 1.91766020281218 0.484 0.357 0.000349701047250258 "PR3" "PTP4A3" "TI_R_P2"

"1848" 1.14098344863723e-08 1.49101875838661 0.513 0.391 0.000381191160355211 "PR3" "PMM1" "TI_R_P2"

"1849" 1.14339877686916e-08 2.90318233903501 0.501 0.378 0.000381998097364219 "PR3" "ZDHHC5" "TI_R_P2"

"1850" 1.15426328162579e-08 3.29433790660259 0.683 0.573 0.00038562781975836 "PR3" "RAB13" "TI_R_P2"

"1851" 1.2071645601058e-08 1.22505404457639 0.542 0.42 0.000403301607885748 "PR3" "DCAF6" "TI_R_P2"

"1852" 1.26891949910841e-08 1.41713210538563 0.58 0.45 0.00042393331545713 "PR3" "UROD" "TI_R_P2"

"1853" 1.34692997309278e-08 1.04644128986922 0.547 0.423 0.000449995834710566 "PR3" "CCT8" "TI_R_P2"

"1854" 1.53192877314894e-08 1.03279088065503 0.566 0.439 0.000511802083821331 "PR3" "HNRNPR" "TI_R_P2"

"1855" 1.78248136988141e-08 1.01401495696669 0.475 0.342 0.000595509200863682 "PR3" "MTREX" "TI_R_P2"

"1856" 1.80487261039397e-08 14.9040376012269 0.726 0.613 0.000602989890406521 "PR3" "AEBP1" "TI_R_P2"

"1857" 2.16031782119153e-08 2.65708563504179 0.721 0.603 0.000721740580881879 "PR3" "CNN2" "TI_R_P2"

"1858" 2.4250303721363e-08 1.23707083085062 0.427 0.307 0.000810178397027016 "PR3" "GOLT1A" "TI_R_P2"

"1859" 2.60567709952755e-08 1.0796651370344 0.501 0.392 0.000870530662181158 "PR3" "PCED1A" "TI_R_P2"

"1860" 3.12531258663513e-08 1.03667162341664 0.558 0.434 0.00104413568206893 "PR3" "SLC35B2" "TI_R_P2"

"1861" 3.16346671587565e-08 1.06317005923778 0.613 0.502 0.0010568825951069 "PR3" "IVNS1ABP" "TI_R_P2"

"1862" 4.21786649421542e-08 1.18972018311399 0.413 0.298 0.00140914701705243 "PR3" "CDK11B" "TI_R_P2"

"1863" 4.41970905914998e-08 1.11731577021393 0.63 0.516 0.00147658059957142 "PR3" "EWSR1" "TI_R_P2"

"1864" 4.50058510215913e-08 1.05847100996978 0.296 0.186 0.00150360047678034 "PR3" "ADAMTSL2" "TI_R_P2"

"1865" 4.57980235907557e-08 1.09563850677286 0.48 0.37 0.00153006617014356 "PR3" "OTUD5" "TI_R_P2"

"1866" 5.22538807152418e-08 1.04457029046065 0.551 0.403 0.00174574990081551 "PR3" "ZFAND6" "TI_R_P2"

"1867" 5.57281235289629e-08 1.03905153400781 0.53 0.406 0.00186182087897912 "PR3" "IST1" "TI_R_P2"

"1868" 5.69312525816199e-08 1.00936454000984 0.585 0.474 0.00190201621749934 "PR3" "ZMAT2" "TI_R_P2"

"1869" 7.25391225386071e-08 1.22018321111561 0.671 0.548 0.00242345954489233 "PR3" "UBE2L6" "TI_R_P2"

"1870" 1.07890100999916e-07 1.02187758993138 0.532 0.423 0.00360450038430618 "PR3" "RBM25" "TI_R_P2"

"1871" 1.37501209215915e-07 1.22245037280502 0.52 0.399 0.0045937778986945 "PR3" "SPTLC2" "TI_R_P2"

"1872" 1.49164108664268e-07 1.30880126163261 0.578 0.472 0.00498342370636454 "PR3" "BNIP3L" "TI_R_P2"

"1873" 1.6270248992698e-07 1.74469122647179 0.513 0.396 0.00543572748597049 "PR3" "NT5DC2" "TI_R_P2"

"1874" 2.08771483797665e-07 2.56766598836253 0.566 0.429 0.00697484650219619 "PR3" "CTDNEP1" "TI_R_P2"

"1875" 2.1769902089152e-07 1.34476975985756 0.499 0.385 0.0072731065889648 "PR3" "DPP9" "TI_R_P2"

"1876" 2.26444509935003e-07 1.14681734127974 0.36 0.249 0.00756528463241853 "PR3" "ARHGAP26" "TI_R_P2"

"1877" 2.77496947434068e-07 1.05621580451706 0.303 0.196 0.00927089551682476 "PR3" "LIG3" "TI_R_P2"

"1878" 4.67879220310439e-100 17.2883927657852 0.614 0.219 1.56313768713515e-95 "PR1" "TM4SF4" "TI_R_P2"

"1879" 1.27190079034935e-95 6.81235387241211 0.483 0.125 4.24929335047813e-91 "PR1" "VSIG1" "TI_R_P2"

"1880" 2.39948401246532e-84 2.12536423476709 0.601 0.233 8.01643613724539e-80 "PR1" "ANPEP" "TI_R_P2"

"1881" 3.59844609956989e-84 40.9038791891474 0.783 0.447 1.2022048574053e-79 "PR1" "MMP7" "TI_R_P2"

"1882" 2.44853770620391e-83 8.21650497367467 0.901 0.718 8.18031962265664e-79 "PR1" "CD55" "TI_R_P2"

"1883" 4.196465913779e-66 9.6022781097114 0.667 0.338 1.40199729713443e-61 "PR1" "SYT8" "TI_R_P2"

"1884" 6.42615526519812e-65 1.69061503109817 0.409 0.122 2.14691421255004e-60 "PR1" "AGER" "TI_R_P2"

"1885" 7.83727384600573e-62 4.87643860502278 0.723 0.425 2.61835481921206e-57 "PR1" "NR4A1" "TI_R_P2"

"1886" 6.39087884051692e-53 3.34009473831648 0.676 0.398 2.1351287118283e-48 "PR1" "CRIP2" "TI_R_P2"

"1887" 1.94481243382468e-52 4.03975920619148 0.45 0.178 6.49742386016487e-48 "PR1" "SCTR" "TI_R_P2"

"1888" 3.13412386866821e-51 36.7878648091986 0.11 0.007 1.04707944328336e-46 "PR1" "GRP" "TI_R_P2"

"1889" 5.86648100790367e-50 2.43034403081972 0.616 0.334 1.95993263993054e-45 "PR1" "IFT57" "TI_R_P2"

"1890" 2.26586522573744e-44 8.79428369395462 0.545 0.279 7.57002913266622e-40 "PR1" "CAV2" "TI_R_P2"

"1891" 5.24308718791696e-42 1.43793830210135 0.382 0.147 1.75166299861118e-37 "PR1" "CLIC5" "TI_R_P2"

"1892" 6.19140266886056e-42 1.62049322635067 0.419 0.173 2.06848571763962e-37 "PR1" "PEBP4" "TI_R_P2"

"1893" 2.84310430970247e-36 3.88978576017898 0.893 0.786 9.49852718828499e-32 "PR1" "LY6E" "TI_R_P2"

"1894" 1.77288090694369e-34 1.71811601851288 0.525 0.308 5.92301782200819e-30 "PR1" "LGALS4" "TI_R_P2"

"1895" 2.92278368374095e-33 3.59318637045448 0.824 0.667 9.76472800901015e-29 "PR1" "GPRC5A" "TI_R_P2"

"1896" 4.91574689013509e-31 3.5219424280718 0.86 0.722 1.64230187852523e-26 "PR1" "CRIP1" "TI_R_P2"

"1897" 6.63513895056383e-31 1.13388330724791 0.281 0.099 2.21673357199387e-26 "PR1" "IFIT1" "TI_R_P2"

"1898" 1.03936870836602e-30 1.88385711206452 0.167 0.04 3.47242691778003e-26 "PR1" "C11orf88" "TI_R_P2"

"1899" 1.31497330277795e-30 1.39752284307359 0.535 0.309 4.39319430725087e-26 "PR1" "CGN" "TI_R_P2"

"1900" 9.46740439033514e-30 1.91401231424228 0.661 0.461 3.16296513276707e-25 "PR1" "DPYSL2" "TI_R_P2"

"1901" 3.27488439617006e-29 1.31807236736268 0.467 0.242 1.09410612791645e-24 "PR1" "NR0B2" "TI_R_P2"

"1902" 4.03610107848881e-29 5.91346864043641 0.63 0.431 1.34842100931233e-24 "PR1" "ITGB6" "TI_R_P2"

"1903" 8.13492580060052e-29 5.20104927519251 0.198 0.058 2.71779736072263e-24 "PR1" "TMEM45A" "TI_R_P2"

"1904" 9.09228691322241e-29 2.51919380561141 0.376 0.174 3.03764213483847e-24 "PR1" "CAV1" "TI_R_P2"

"1905" 2.32064849703047e-28 1.71347096270683 0.18 0.049 7.75305456372908e-24 "PR1" "OMG" "TI_R_P2"

"1906" 5.68915126282029e-28 3.21159236534757 0.736 0.601 1.90068854539563e-23 "PR1" "MSLN" "TI_R_P2"

"1907" 3.11624253814079e-27 2.45783008366895 0.787 0.651 1.04110546956746e-22 "PR1" "SPINT1" "TI_R_P2"

"1908" 5.76890140918993e-27 1.64798236666528 0.624 0.394 1.92733227179626e-22 "PR1" "DMKN" "TI_R_P2"

"1909" 1.67696449025548e-26 4.09573622887475 0.678 0.51 5.60257066549454e-22 "PR1" "DPP4" "TI_R_P2"

"1910" 3.23632684731923e-26 2.82601225012272 0.777 0.653 1.08122443642088e-21 "PR1" "C19orf33" "TI_R_P2"

"1911" 2.76027999992451e-25 2.56609895546848 0.758 0.607 9.22181945174781e-21 "PR1" "PLS3" "TI_R_P2"

"1912" 3.60382921147184e-25 1.56356254015586 0.219 0.075 1.20400330126063e-20 "PR1" "CXCL1" "TI_R_P2"

"1913" 4.47176356270468e-25 1.92678400478529 0.736 0.579 1.49397148866401e-20 "PR1" "TMPRSS2" "TI_R_P2"

"1914" 1.10264532578035e-24 1.3924164788586 0.438 0.241 3.68382776889956e-20 "PR1" "ICAM4" "TI_R_P2"

"1915" 3.47276364918477e-24 1.58602626121222 0.275 0.113 1.16021560755614e-19 "PR1" "CD38" "TI_R_P2"

"1916" 4.59095033984632e-24 2.87723117648838 0.607 0.413 1.53379059903926e-19 "PR1" "PON2" "TI_R_P2"

"1917" 1.60291241080257e-23 1.14301781427116 0.628 0.434 5.35517007325032e-19 "PR1" "TRIP6" "TI_R_P2"

"1918" 1.9352545755644e-23 1.01921171068758 0.343 0.167 6.4654920115031e-19 "PR1" "RASGRF1" "TI_R_P2"

"1919" 2.57630511002689e-23 1.74950689692906 0.659 0.494 8.60717774208883e-19 "PR1" "CYB5R1" "TI_R_P2"

"1920" 4.31684168129148e-23 2.50161902910258 0.349 0.169 1.44221363730267e-18 "PR1" "CFAP221" "TI_R_P2"

"1921" 5.1025387746501e-23 1.12721751718206 0.829 0.719 1.70470717922285e-18 "PR1" "GNB2" "TI_R_P2"

"1922" 5.59746781477495e-23 2.59498143011117 0.227 0.086 1.87005802223816e-18 "PR1" "C5orf49" "TI_R_P2"

"1923" 1.04068673925775e-22 2.74501412652268 0.686 0.511 3.47683032718622e-18 "PR1" "PTP4A1" "TI_R_P2"

"1924" 5.51959953839279e-22 1.85222154352673 0.486 0.295 1.84404300978165e-17 "PR1" "SLC1A1" "TI_R_P2"

"1925" 1.26817401938199e-21 2.33681249116997 0.566 0.367 4.23684258135329e-17 "PR1" "GPC4" "TI_R_P2"

"1926" 1.42469803666582e-21 1.50155354115057 0.711 0.534 4.75977367069684e-17 "PR1" "RHBDD2" "TI_R_P2"

"1927" 2.2082779424931e-21 1.37454081933748 0.56 0.359 7.37763577807519e-17 "PR1" "LAMA5" "TI_R_P2"

"1928" 3.07772919409761e-21 1.5143356641121 0.857 0.729 1.02823854645607e-16 "PR1" "ANXA5" "TI_R_P2"

"1929" 5.78719106454582e-21 1.19322341450501 0.835 0.715 1.93344266275411e-16 "PR1" "ERP29" "TI_R_P2"

"1930" 1.57021466448059e-20 1.15301768837117 0.581 0.415 5.24593017256319e-16 "PR1" "ANXA4" "TI_R_P2"

"1931" 3.82263564558008e-20 1.12469654286274 0.388 0.212 1.27710434283185e-15 "PR1" "CRABP2" "TI_R_P2"

"1932" 4.37373247274784e-20 1.58931589663545 0.599 0.41 1.46122028182033e-15 "PR1" "ALDH3A2" "TI_R_P2"

"1933" 6.06982294724958e-20 1.36299236852001 0.738 0.578 2.02786714844661e-15 "PR1" "LTA4H" "TI_R_P2"

"1934" 6.61658208720011e-20 1.80736622553402 0.605 0.435 2.21053390951268e-15 "PR1" "SNRPN" "TI_R_P2"

"1935" 7.85180861264914e-20 1.30780563229214 0.159 0.051 2.62321073939995e-15 "PR1" "LDLRAD1" "TI_R_P2"

"1936" 9.14466739328301e-20 2.6899057162226 0.519 0.329 3.05514192942192e-15 "PR1" "ERRFI1" "TI_R_P2"

"1937" 1.1509612739655e-19 1.24936995398805 0.645 0.466 3.84524652019133e-15 "PR1" "TPM1" "TI_R_P2"

"1938" 2.24701785924736e-19 2.66622817974096 0.574 0.393 7.50706196595952e-15 "PR1" "NPTN" "TI_R_P2"

"1939" 2.40440875819722e-19 2.07623512340518 0.669 0.505 8.0328892202611e-15 "PR1" "CYBRD1" "TI_R_P2"

"1940" 4.58611364528377e-19 2.32077325196906 0.508 0.319 1.53217470775285e-14 "PR1" "CYR61" "TI_R_P2"

"1941" 1.00252105610457e-18 2.87286885636574 0.791 0.671 3.34932259633974e-14 "PR1" "HCFC1R1" "TI_R_P2"

"1942" 1.14068776480894e-18 3.07491280780781 0.188 0.071 3.81092375345019e-14 "PR1" "PLEKHS1" "TI_R_P2"

"1943" 1.28921172948305e-18 2.83992497762166 0.576 0.415 4.30712746702993e-14 "PR1" "AKAP13" "TI_R_P2"

"1944" 1.63832495227073e-18 3.74386899496537 0.632 0.461 5.47347983304129e-14 "PR1" "CLIC6" "TI_R_P2"

"1945" 2.16149934108801e-18 2.31228355818179 0.791 0.661 7.22135314864092e-14 "PR1" "TKT" "TI_R_P2"

"1946" 5.35909367742659e-18 2.13834257725903 0.7 0.555 1.79041960669145e-13 "PR1" "HEXA" "TI_R_P2"

"1947" 9.04032113506484e-18 3.30740699490841 0.593 0.434 3.02028088801381e-13 "PR1" "SFTA1P" "TI_R_P2"

"1948" 1.08219104772122e-17 1.51858529039646 0.781 0.645 3.61549207133182e-13 "PR1" "MLEC" "TI_R_P2"

"1949" 1.42421454555055e-17 1.31373777764793 0.618 0.438 4.75815837522983e-13 "PR1" "STEAP3" "TI_R_P2"

"1950" 1.76690244413117e-17 1.62432442212856 0.523 0.356 5.90304437559781e-13 "PR1" "GGCX" "TI_R_P2"

"1951" 2.55885947092534e-17 1.85247712650942 0.878 0.774 8.54889360641448e-13 "PR1" "PCBP1" "TI_R_P2"

"1952" 2.69120276650369e-17 4.50339873066418 0.752 0.621 8.99103932261217e-13 "PR1" "PDZK1IP1" "TI_R_P2"

"1953" 2.76750385567461e-17 1.2505757927825 0.533 0.375 9.24595363142331e-13 "PR1" "IVD" "TI_R_P2"

"1954" 3.62096518868034e-17 1.77259374118662 0.159 0.057 1.20972825988622e-12 "PR1" "C2orf40" "TI_R_P2"

"1955" 7.24550547569892e-17 1.74216804142859 0.674 0.522 2.42065092437625e-12 "PR1" "CEBPD" "TI_R_P2"

"1956" 8.98507323506224e-17 1.20655818458862 0.44 0.272 3.00182311710194e-12 "PR1" "PCK2" "TI_R_P2"

"1957" 1.27484493149638e-16 1.5569806996611 0.605 0.45 4.25912943163624e-12 "PR1" "MBIP" "TI_R_P2"

"1958" 1.27620277402952e-16 1.23387342239416 0.764 0.615 4.26366584775523e-12 "PR1" "UNC93B1" "TI_R_P2"

"1959" 1.52067067004676e-16 1.03239831111597 0.287 0.143 5.08040864155921e-12 "PR1" "NELL1" "TI_R_P2"

"1960" 1.894250325163e-16 20.902171816122 0.202 0.086 6.32850091133707e-12 "PR1" "SAA1" "TI_R_P2"

"1961" 2.89083185056117e-16 1.90526114078911 0.746 0.642 9.65798012953981e-12 "PR1" "HSBP1" "TI_R_P2"

"1962" 4.52578059316075e-16 2.73194317487146 0.705 0.563 1.51201803836907e-11 "PR1" "ALDH1A1" "TI_R_P2"

"1963" 6.58270050611823e-16 2.55981328422537 0.733 0.593 2.19921441208904e-11 "PR1" "PNKD" "TI_R_P2"

"1964" 1.016473496048e-15 5.88956800714123 0.721 0.572 3.39593630294675e-11 "PR1" "ITGB2" "TI_R_P2"

"1965" 1.08118939206941e-15 1.53898119463402 0.465 0.303 3.61214563996471e-11 "PR1" "WFS1" "TI_R_P2"

"1966" 1.16964122335885e-15 4.6752688658468 0.523 0.348 3.90765436311958e-11 "PR1" "DUOX1" "TI_R_P2"

"1967" 1.18053232177501e-15 1.22832527048014 0.791 0.674 3.94404043381814e-11 "PR1" "IFITM2" "TI_R_P2"

"1968" 1.54856246904698e-15 1.49237708239375 0.502 0.339 5.17359235283905e-11 "PR1" "PPP4R1" "TI_R_P2"

"1969" 4.13223793339099e-15 2.19841443855671 0.783 0.657 1.38053937116659e-10 "PR1" "GNG5" "TI_R_P2"

"1970" 1.78960119501125e-14 1.26457394161104 0.781 0.641 5.97887863241309e-10 "PR1" "ITGA3" "TI_R_P2"

"1971" 1.84384347245403e-14 1.44843462441401 0.76 0.635 6.16009665712168e-10 "PR1" "MRPL33" "TI_R_P2"

"1972" 2.86645993702201e-14 1.50335654106681 0.674 0.524 9.57655600359682e-10 "PR1" "SMPDL3B" "TI_R_P2"

"1973" 2.97358901767676e-14 1.12601807116747 0.787 0.664 9.93446354915629e-10 "PR1" "YWHAE" "TI_R_P2"

"1974" 5.30720906574945e-14 5.99467465088883 0.494 0.343 1.77308547677623e-09 "PR1" "PFKFB3" "TI_R_P2"

"1975" 5.87567216380345e-14 2.22136983078003 0.8 0.67 1.96300331320509e-09 "PR1" "PPP1CB" "TI_R_P2"

"1976" 9.00360729311049e-14 3.38085479602075 0.178 0.076 3.00801516055528e-09 "PR1" "DMBT1" "TI_R_P2"

"1977" 1.46242140125391e-13 1.84626958866925 0.804 0.701 4.88580365944919e-09 "PR1" "ATP5MC3" "TI_R_P2"

"1978" 1.51110437234612e-13 1.53879628213321 0.18 0.079 5.04844859757116e-09 "PR1" "TMEM231" "TI_R_P2"

"1979" 1.54280412697377e-13 5.64959796301671 0.568 0.419 5.15435430780668e-09 "PR1" "FBP1" "TI_R_P2"

"1980" 1.85105045001878e-13 1.53293386347299 0.403 0.258 6.18417444846773e-09 "PR1" "EVA1A" "TI_R_P2"

"1981" 1.92910263640451e-13 2.00027860036427 0.579 0.446 6.44493899796383e-09 "PR1" "CAPN12" "TI_R_P2"

"1982" 2.17934546097985e-13 1.06704134823084 0.781 0.658 7.2809752505876e-09 "PR1" "PTTG1IP" "TI_R_P2"

"1983" 2.91113458240122e-13 2.05167432505413 0.376 0.231 9.72580952634424e-09 "PR1" "ABCA1" "TI_R_P2"

"1984" 3.07796009284668e-13 2.28163796827118 0.45 0.314 1.02831568741915e-08 "PR1" "MYOF" "TI_R_P2"

"1985" 5.50992164924244e-13 1.07653685016056 0.62 0.486 1.84080972379541e-08 "PR1" "PDLIM1" "TI_R_P2"

"1986" 5.59945052488487e-13 2.57256872869643 0.545 0.412 1.87072042585879e-08 "PR1" "CFH" "TI_R_P2"

"1987" 5.68312266404709e-13 3.18684775514918 0.601 0.458 1.89867445083149e-08 "PR1" "COL18A1" "TI_R_P2"

"1988" 6.6533021530803e-13 2.44634284946598 0.384 0.242 2.2228017163226e-08 "PR1" "MYO5C" "TI_R_P2"

"1989" 8.23717893520926e-13 1.21371863268617 0.401 0.256 2.75195911046406e-08 "PR1" "UNC13D" "TI_R_P2"

"1990" 1.00518961184839e-12 2.66325060716664 0.271 0.148 3.35823797422428e-08 "PR1" "PLK2" "TI_R_P2"

"1991" 1.40946824439974e-12 1.32162444269492 0.653 0.538 4.70889245771508e-08 "PR1" "POR" "TI_R_P2"

"1992" 1.83375666397061e-12 1.45230445415926 0.76 0.65 6.12639763865941e-08 "PR1" "CERS2" "TI_R_P2"

"1993" 3.04066780720201e-12 1.1282552252737 0.459 0.31 1.01585670770812e-07 "PR1" "NEDD9" "TI_R_P2"

"1994" 3.07551126554615e-12 1.04400228989572 0.585 0.443 1.02749755870631e-07 "PR1" "INAVA" "TI_R_P2"

"1995" 3.08374246695717e-12 1.23701371366342 0.566 0.415 1.03024752078572e-07 "PR1" "PLEKHB2" "TI_R_P2"

"1996" 3.35983119920256e-12 1.60686976044197 0.283 0.159 1.12248600534158e-07 "PR1" "S100A1" "TI_R_P2"

"1997" 3.65292060381307e-12 5.11626993044124 0.764 0.662 1.22040424452791e-07 "PR1" "SLC22A31" "TI_R_P2"

"1998" 3.80770757904144e-12 1.61604922860278 0.837 0.732 1.27211702508195e-07 "PR1" "ADI1" "TI_R_P2"

"1999" 5.50301718848529e-12 1.05986841827906 0.694 0.555 1.83850301250105e-07 "PR1" "YPEL5" "TI_R_P2"

"2000" 5.82867878447863e-12 1.19423453597777 0.409 0.27 1.94730329510647e-07 "PR1" "ECE1" "TI_R_P2"

"2001" 6.02474098825825e-12 2.48171411591361 0.669 0.53 2.0128057167672e-07 "PR1" "GAS6" "TI_R_P2"

"2002" 8.01999904065724e-12 1.0847913651354 0.643 0.51 2.67940147949318e-07 "PR1" "KRTCAP3" "TI_R_P2"

"2003" 1.07247843978684e-11 2.03341234306258 0.444 0.303 3.58304321948386e-07 "PR1" "GCHFR" "TI_R_P2"

"2004" 1.84095376234153e-11 1.58196919645486 0.671 0.547 6.15044242460683e-07 "PR1" "IER3" "TI_R_P2"

"2005" 2.07134543974199e-11 3.24192256809859 0.597 0.45 6.92015797963401e-07 "PR1" "HNRNPF" "TI_R_P2"

"2006" 2.14873418983254e-11 2.7018426902701 0.298 0.178 7.17870605481153e-07 "PR1" "CES1" "TI_R_P2"

"2007" 2.80311840620985e-11 2.54331012404408 0.822 0.701 9.36493828330648e-07 "PR1" "LRPAP1" "TI_R_P2"

"2008" 3.30214848686264e-11 1.01224109207893 0.579 0.446 1.10321478797594e-06 "PR1" "PPL" "TI_R_P2"

"2009" 3.67542175466882e-11 1.12355025390395 0.415 0.282 1.22792165401731e-06 "PR1" "H2AFJ" "TI_R_P2"

"2010" 3.87706598841993e-11 4.27850942970631 0.787 0.677 1.29528897607121e-06 "PR1" "TPP1" "TI_R_P2"

"2011" 3.89311366742667e-11 1.63035515830709 0.62 0.485 1.30065034515058e-06 "PR1" "SCAMP2" "TI_R_P2"

"2012" 4.13617330793438e-11 2.26549398915149 0.436 0.299 1.3818541404478e-06 "PR1" "LAMC2" "TI_R_P2"

"2013" 4.67293777491477e-11 1.47284866602202 0.614 0.485 1.56118178122128e-06 "PR1" "NCKAP1" "TI_R_P2"

"2014" 5.16150181318798e-11 1.09778997346719 0.304 0.183 1.72440614076797e-06 "PR1" "GALNT18" "TI_R_P2"

"2015" 5.38605236621045e-11 3.50975258155101 0.51 0.372 1.79942623502725e-06 "PR1" "ASCC2" "TI_R_P2"

"2016" 5.46169398039633e-11 1.87108953303357 0.601 0.464 1.82469734191061e-06 "PR1" "TRIB1" "TI_R_P2"

"2017" 7.77498793546196e-11 8.08932090105317 0.264 0.158 2.59754571935849e-06 "PR1" "CP" "TI_R_P2"

"2018" 7.92040649520108e-11 1.88256925649695 0.758 0.652 2.64612860598173e-06 "PR1" "ADIPOR1" "TI_R_P2"

"2019" 8.31205773930707e-11 1.57154227694059 0.636 0.533 2.7769753701251e-06 "PR1" "ARHGEF2" "TI_R_P2"

"2020" 1.1759829864088e-10 1.02634071634263 0.585 0.463 3.92884155929315e-06 "PR1" "METAP2" "TI_R_P2"

"2021" 1.22101709565875e-10 1.43198027365581 0.731 0.628 4.07929601488632e-06 "PR1" "IQGAP1" "TI_R_P2"

"2022" 1.34797930738361e-10 2.10323192537807 0.37 0.242 4.5034640680379e-06 "PR1" "CFAP298" "TI_R_P2"

"2023" 1.86148941093617e-10 3.89135800496288 0.244 0.138 6.21904997299667e-06 "PR1" "OLR1" "TI_R_P2"

"2024" 1.88895594321755e-10 1.05468939022016 0.769 0.667 6.31081291069551e-06 "PR1" "MTCH1" "TI_R_P2"

"2025" 2.08779425740025e-10 2.02289983510459 0.738 0.604 6.9751118345485e-06 "PR1" "SSR3" "TI_R_P2"

"2026" 2.5350996345084e-10 1.04814789760242 0.579 0.444 8.46951436892912e-06 "PR1" "PTPMT1" "TI_R_P2"

"2027" 3.11292783988954e-10 1.0325910185081 0.57 0.44 1.0399980620287e-05 "PR1" "RAB10" "TI_R_P2"

"2028" 3.1233203729755e-10 2.09553382304043 0.738 0.625 1.04347010340738e-05 "PR1" "PBXIP1" "TI_R_P2"

"2029" 4.3945366114491e-10 1.51794276972804 0.591 0.458 1.46817073651903e-05 "PR1" "STK25" "TI_R_P2"

"2030" 7.13179020382507e-10 1.11537599460446 0.764 0.658 2.38265978919592e-05 "PR1" "RPN1" "TI_R_P2"

"2031" 7.68255808701712e-10 1.15251657050129 0.432 0.308 2.56666583129155e-05 "PR1" "TUBA4A" "TI_R_P2"

"2032" 8.71785326611883e-10 1.80631909769006 0.653 0.545 2.91254759767764e-05 "PR1" "XPO1" "TI_R_P2"

"2033" 9.37240097682295e-10 1.2200849662953 0.57 0.453 3.13122544234678e-05 "PR1" "RAB2A" "TI_R_P2"

"2034" 9.94508196726413e-10 1.00046495246999 0.564 0.44 3.32255243444327e-05 "PR1" "ARPP19" "TI_R_P2"

"2035" 1.00662845522637e-09 10.8959949703702 0.773 0.67 3.36304500606578e-05 "PR1" "CAPG" "TI_R_P2"

"2036" 1.17314246565177e-09 1.55679042770298 0.523 0.385 3.91935166349598e-05 "PR1" "DNALI1" "TI_R_P2"

"2037" 1.26477604124333e-09 2.41004452683832 0.331 0.214 4.22549027618984e-05 "PR1" "CYP4X1" "TI_R_P2"

"2038" 1.61682948512585e-09 3.84532529941609 0.678 0.559 5.40166562685696e-05 "PR1" "ADAM8" "TI_R_P2"

"2039" 2.03440457458165e-09 1.07881592800793 0.521 0.4 6.79674224321982e-05 "PR1" "TRIM22" "TI_R_P2"

"2040" 2.25794130398507e-09 1.85649699531419 0.605 0.491 7.54355610248371e-05 "PR1" "SLC39A1" "TI_R_P2"

"2041" 2.37713347783094e-09 1.23248620739147 0.667 0.543 7.94176523608539e-05 "PR1" "CTDSP1" "TI_R_P2"

"2042" 2.3980408977968e-09 1.79607747855982 0.475 0.353 8.01161483544934e-05 "PR1" "GSAP" "TI_R_P2"

"2043" 2.95475420453076e-09 1.18503334947569 0.552 0.437 9.87153832191681e-05 "PR1" "ORMDL3" "TI_R_P2"

"2044" 3.26247218527118e-09 1.00140870690552 0.731 0.619 0.000108995933237725 "PR1" "SF3B6" "TI_R_P2"

"2045" 5.48902770334652e-09 2.7592991104583 0.494 0.369 0.000183382926541104 "PR1" "CEBPB" "TI_R_P2"

"2046" 6.48892025237992e-09 1.27415716355783 0.512 0.39 0.000216788336711761 "PR1" "ACSF2" "TI_R_P2"

"2047" 8.62794627142843e-09 1.32821694682793 0.477 0.346 0.000288251056982153 "PR1" "SEMA4B" "TI_R_P2"

"2048" 1.29692366296383e-08 1.67792609337616 0.676 0.561 0.000433289226559585 "PR1" "PRKAB1" "TI_R_P2"

"2049" 1.32274367246603e-08 1.56296703761845 0.618 0.508 0.000441915433534177 "PR1" "NDUFAF3" "TI_R_P2"

"2050" 1.44767502563209e-08 1.99666577347995 0.488 0.362 0.000483653749313423 "PR1" "CCDC24" "TI_R_P2"

"2051" 2.81488537041923e-08 1.14500007736861 0.709 0.601 0.00094042505340336 "PR1" "TMBIM1" "TI_R_P2"

"2052" 3.73110802006197e-08 2.09889266456028 0.808 0.687 0.0012465258784225 "PR1" "NONO" "TI_R_P2"

"2053" 5.20387715165065e-08 1.12324713217305 0.477 0.365 0.00173856331759496 "PR1" "RCN2" "TI_R_P2"

"2054" 5.38280852326922e-08 1.2055816308097 0.626 0.519 0.00179834249953901 "PR1" "SSBP1" "TI_R_P2"

"2055" 5.74616707344763e-08 2.76068371959596 0.428 0.313 0.00191973695756812 "PR1" "ABR" "TI_R_P2"

"2056" 5.9193892747838e-08 3.03266113351773 0.483 0.362 0.00197760876281252 "PR1" "ATP6V1B2" "TI_R_P2"

"2057" 6.6831010486645e-08 1.04589792400465 0.413 0.3 0.00223275722934832 "PR1" "HK1" "TI_R_P2"

"2058" 7.16098729498245e-08 1.36118344985306 0.51 0.395 0.00239241424538069 "PR1" "YAP1" "TI_R_P2"

"2059" 8.56037716011414e-08 1.17872003813149 0.556 0.443 0.00285993640542253 "PR1" "TPD52L2" "TI_R_P2"

"2060" 1.11789170902803e-07 12.3365430476674 0.49 0.373 0.00373476441069175 "PR1" "SCD" "TI_R_P2"

"2061" 1.17390219138958e-07 1.4079253182024 0.583 0.48 0.00392188983121344 "PR1" "STAT2" "TI_R_P2"

"2062" 1.24377377704023e-07 9.42471875715136 0.341 0.24 0.0041553238117137 "PR1" "MARCO" "TI_R_P2"

"2063" 1.46341473958734e-07 1.89975920766988 0.558 0.436 0.00488912230348735 "PR1" "SLC25A29" "TI_R_P2"

"2064" 1.59714404023239e-07 1.49793558101882 0.572 0.459 0.00533589852401241 "PR1" "LARP4B" "TI_R_P2"

"2065" 1.63048873080697e-07 1.31203525724507 0.401 0.291 0.005447299800753 "PR1" "PHLDA1" "TI_R_P2"

"2066" 2.02617037079053e-07 1.44675014488697 0.583 0.465 0.00676923259177408 "PR1" "INTS3" "TI_R_P2"

"2067" 2.09889569992135e-07 1.27712181176542 0.415 0.308 0.00701220064386725 "PR1" "PDHA1" "TI_R_P2"

"2068" 3.81320491446155e-11 9.94645473427529 0.659 0.542 1.27395362987246e-06 "PR2" "GSTA1" "TI_R_P2"

"2069" 5.77663398475262e-09 1.05451561680437 0.509 0.381 0.0001929915647966 "PR2" "SLC39A4" "TI_R_P2"

"2070" 6.96575835566538e-09 3.17479071044555 0.893 0.792 0.000232719020904425 "PR2" "SEM1" "TI_R_P2"

"2071" 1.14061287895643e-08 1.80024883068848 0.891 0.782 0.000381067356730554 "PR2" "HNRNPA3" "TI_R_P2"

"2072" 2.77965699942391e-08 4.18434910224518 0.517 0.384 0.000928655606937534 "PR2" "KLK11" "TI_R_P2"

"2073" 6.15135427495363e-08 4.44706323028645 0.788 0.677 0.00205510594971926 "PR2" "TALDO1" "TI_R_P2"

"2074" 6.2871160245089e-08 3.47623400064249 0.848 0.74 0.00210046259262818 "PR2" "SERPINB1" "TI_R_P2"
