# Supplementary figures and images for: Multidirectional characterization of cellular composition and spatial architecture in human multiple primary lung cancers
Source: Cell Death Dis. 2023 Jul 25;14(7):462. doi: 10.1038/s41419-023-05992-w (PMC10366158; doi:10.1038/s41419-023-05992-w)

NM\_R\_P1

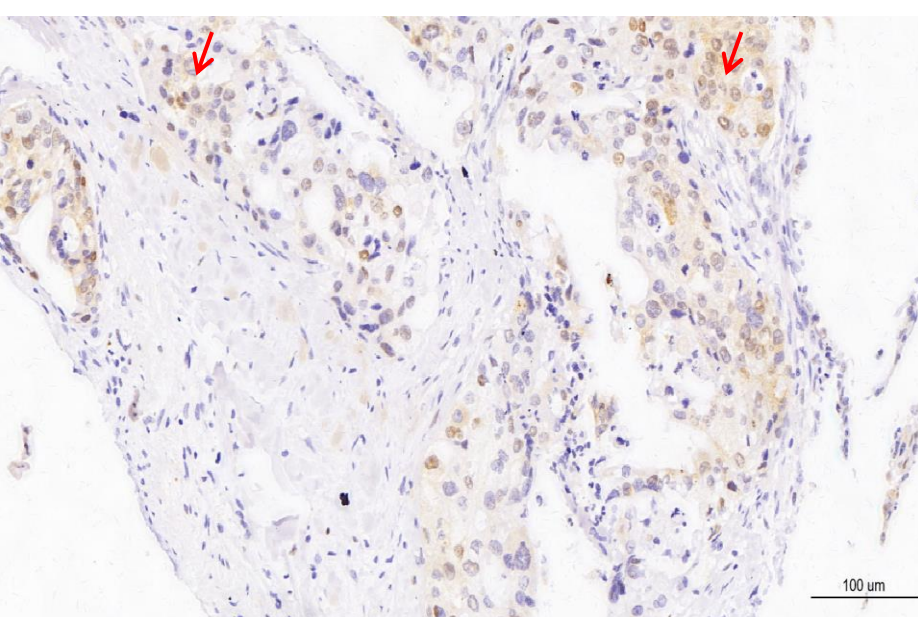

TM\_R\_P1

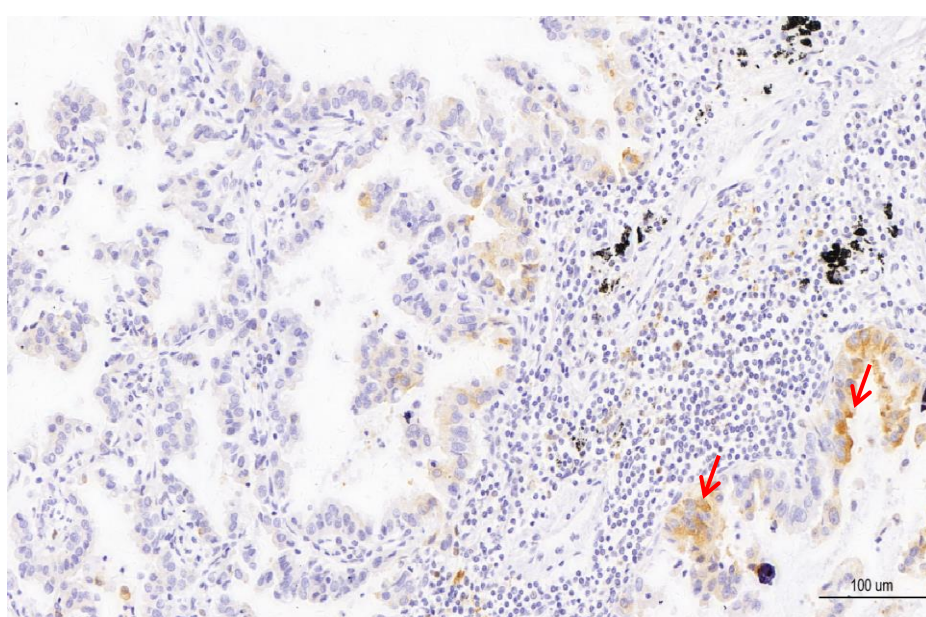

NI\_R\_P1

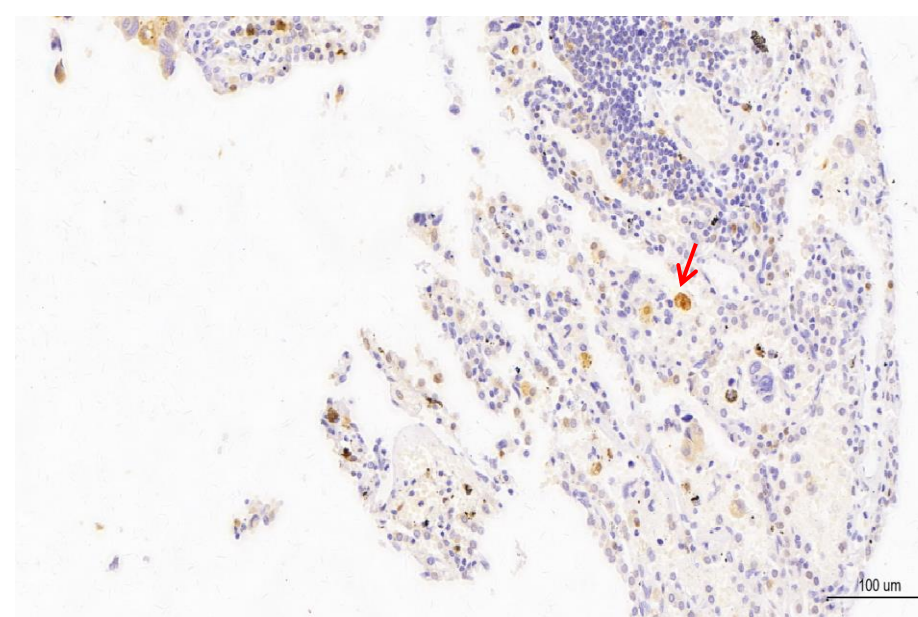

TI\_R\_P1

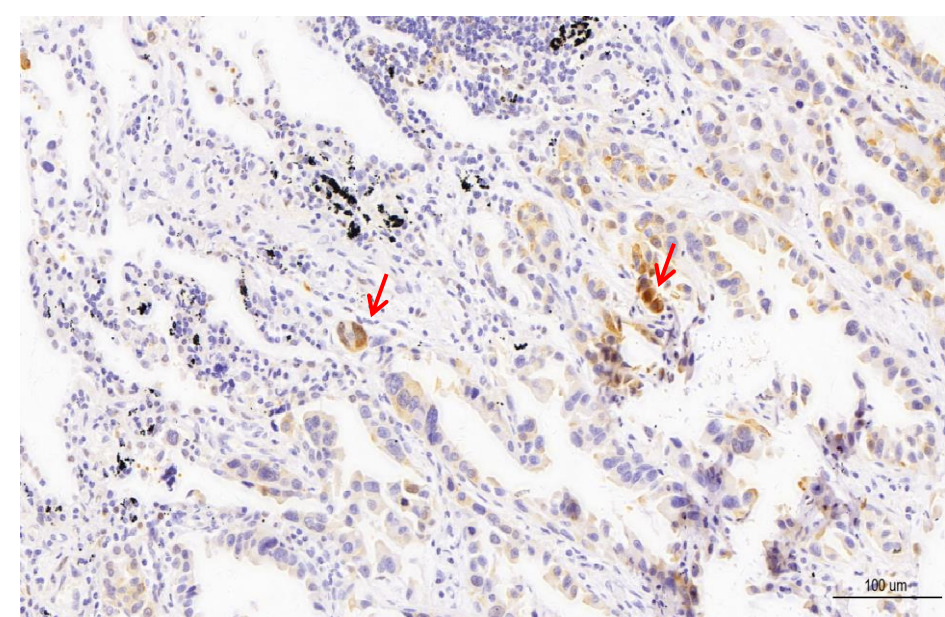

NM\_R\_P2

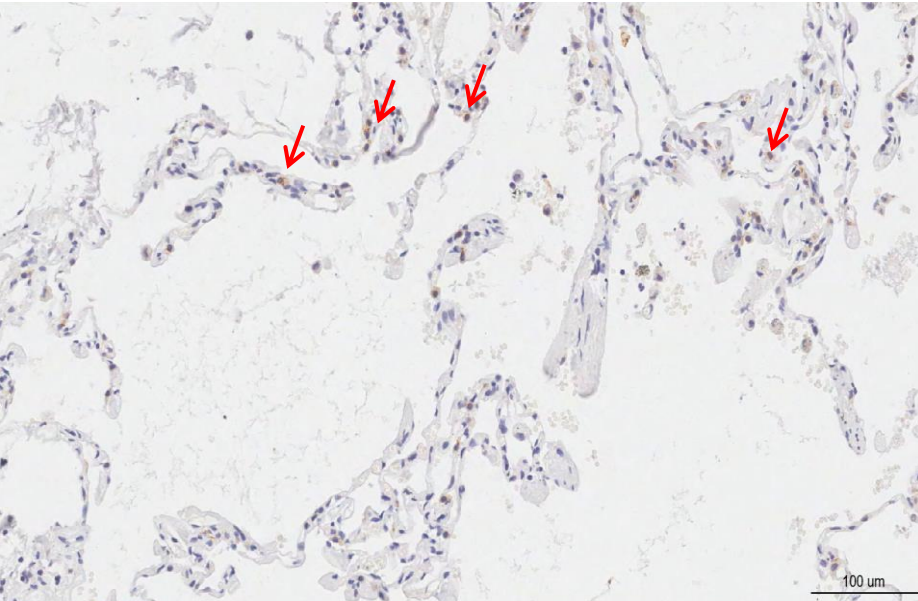

TM\_R\_P2

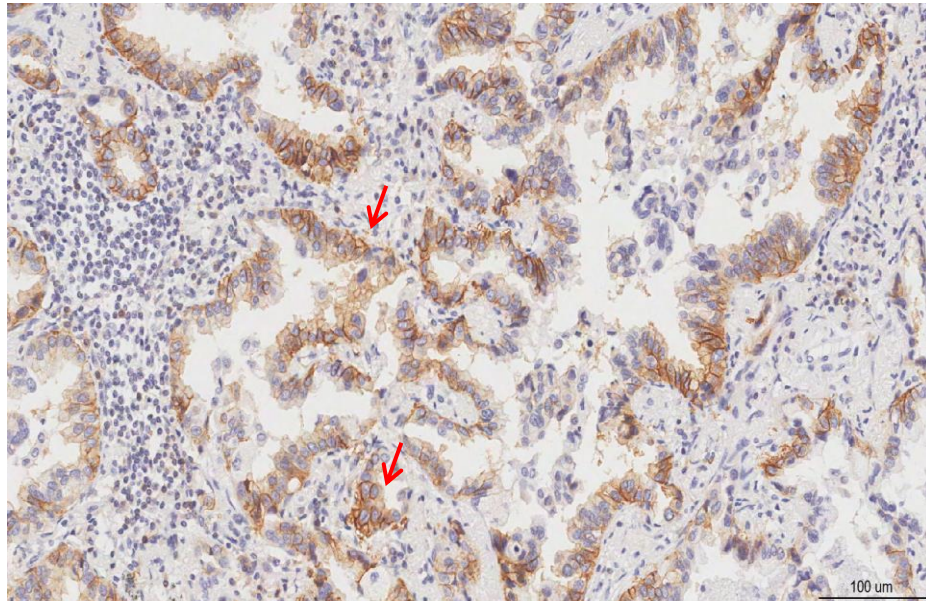

NI\_R\_P2

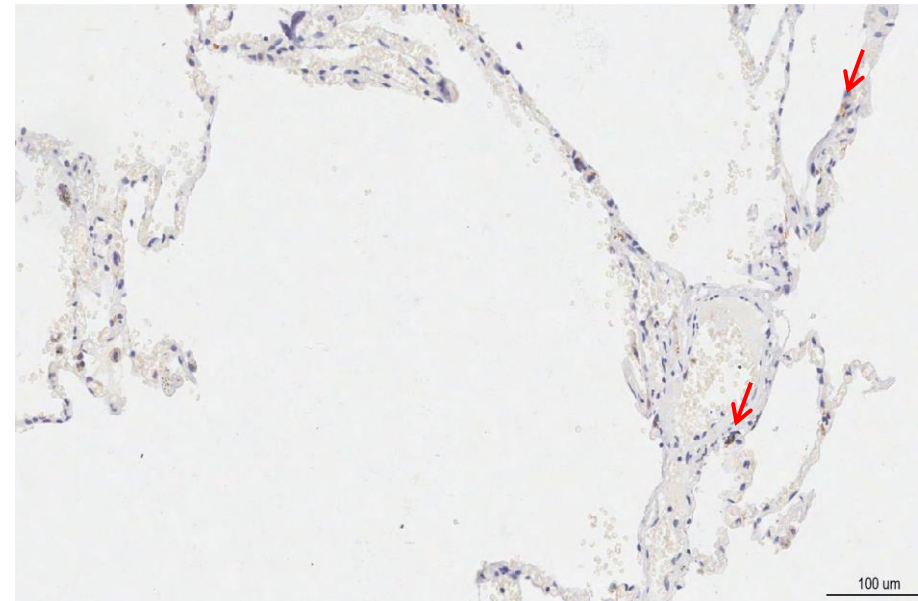

TI\_R\_P2

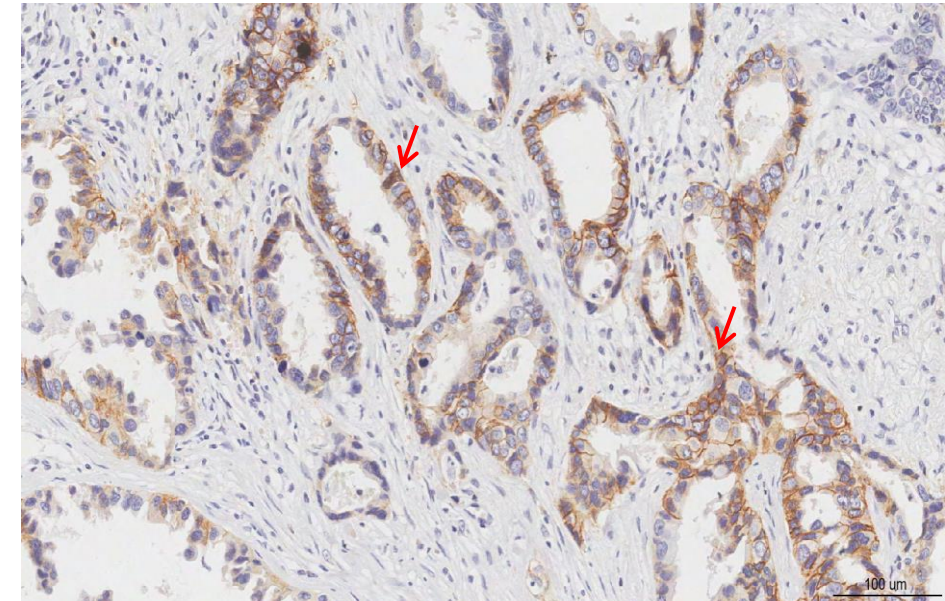

NM\_R\_P3

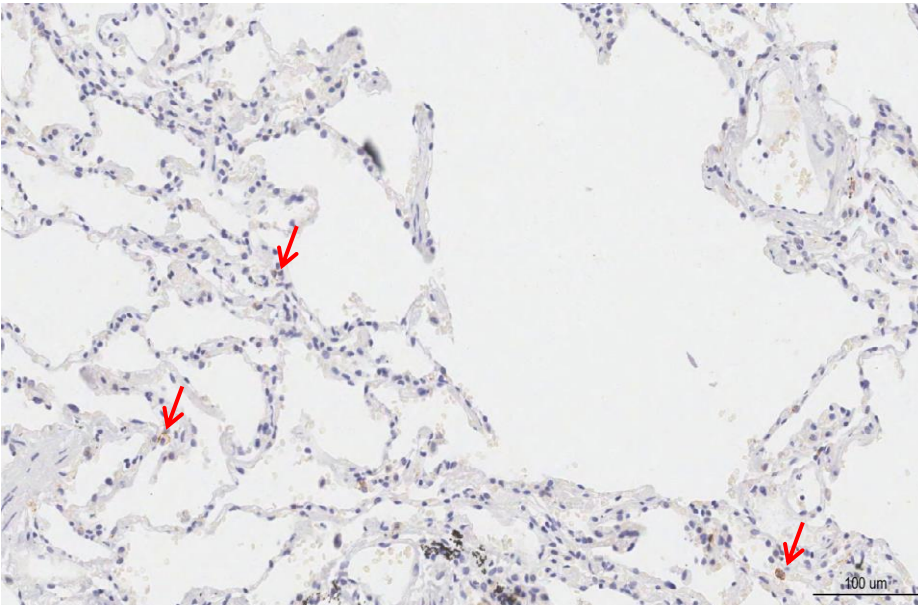

TM\_R\_P3

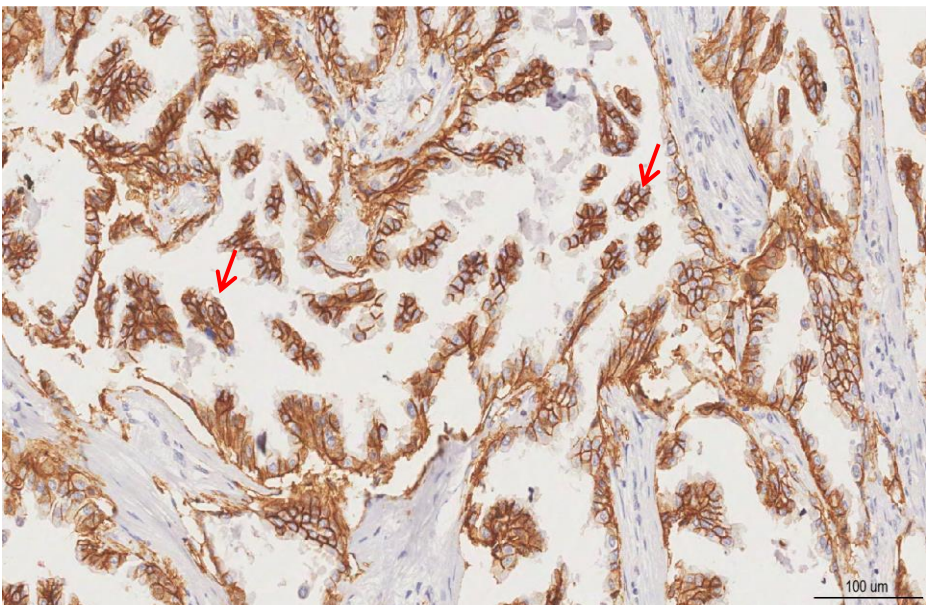

NI\_R\_P3

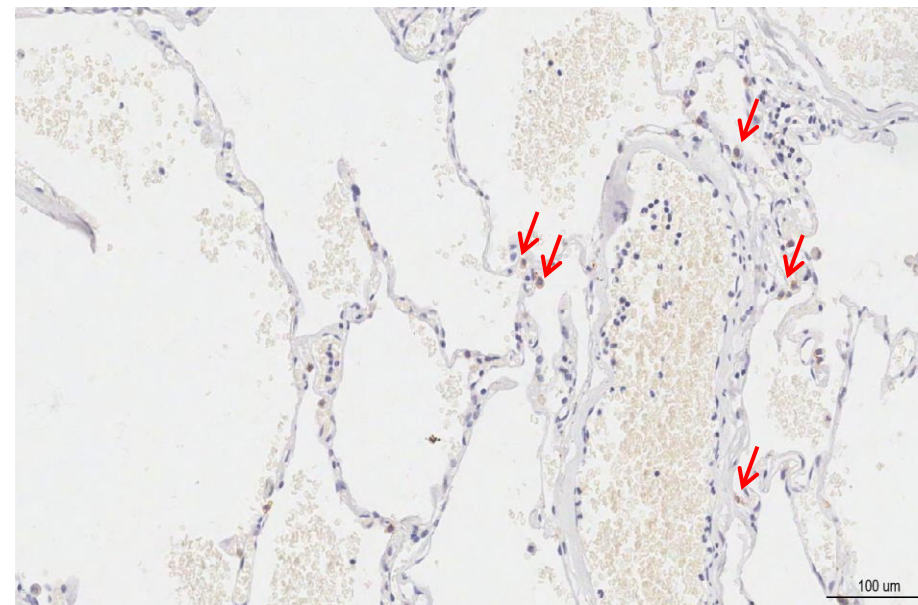

TI\_R\_P3

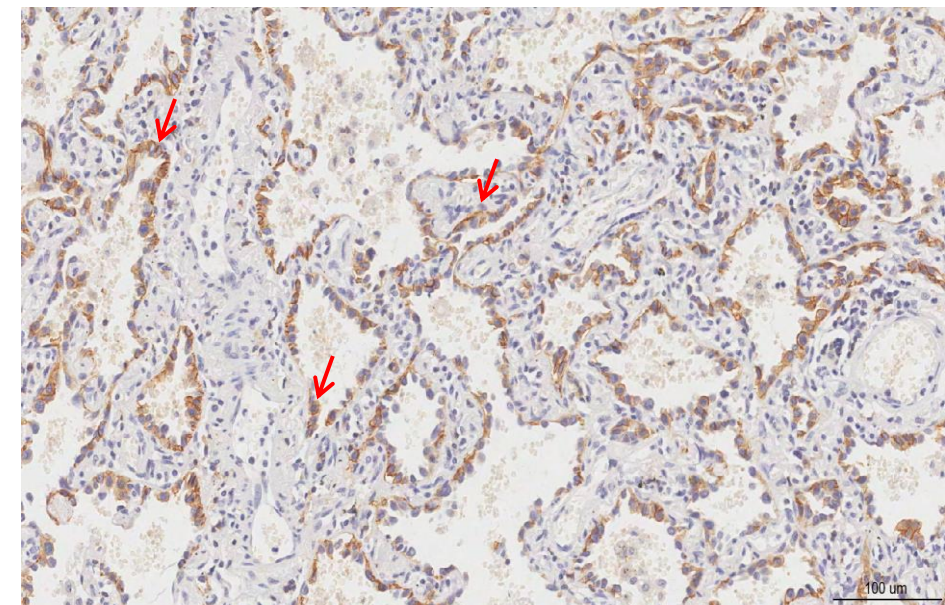

NI\_L\_P4

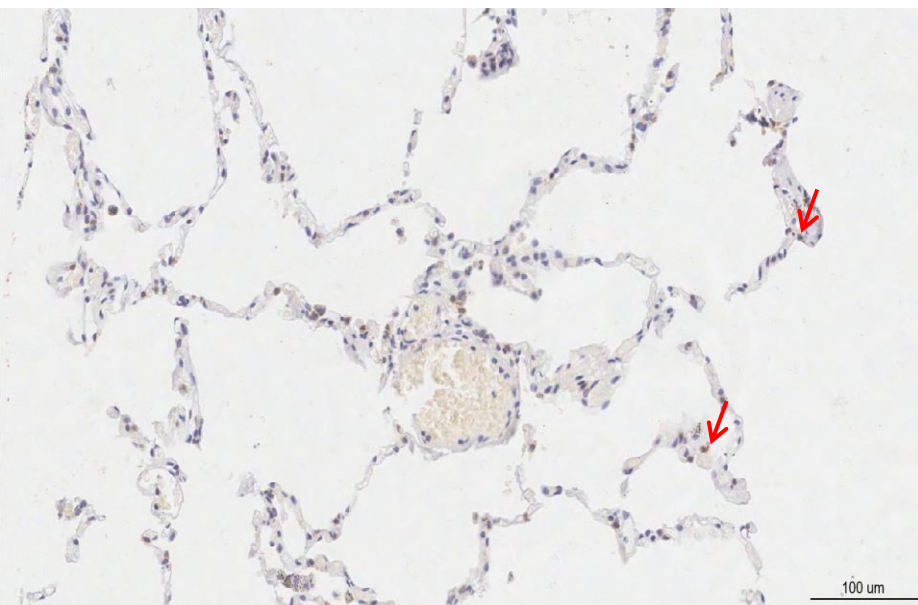

TI\_L\_P4

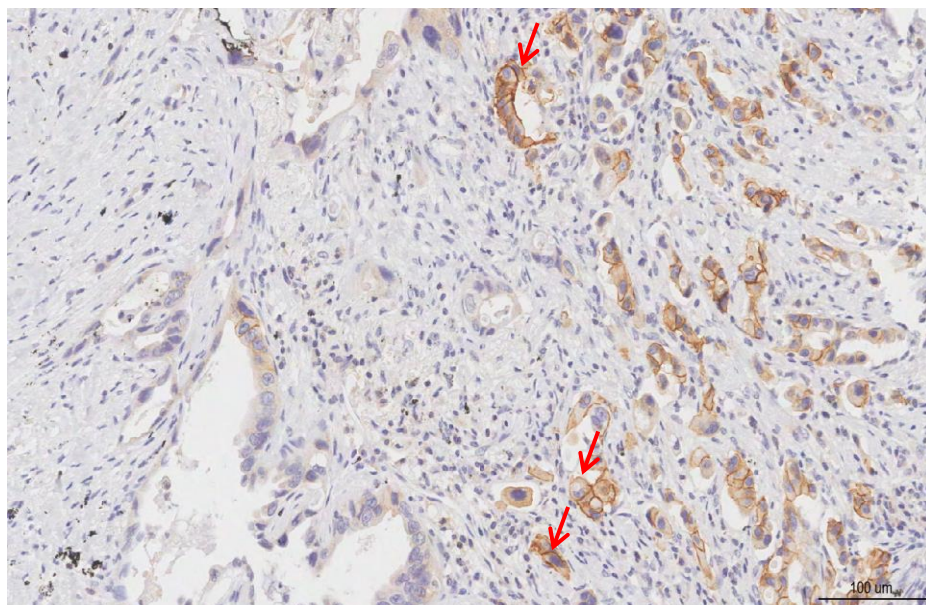

NS\_L\_P4

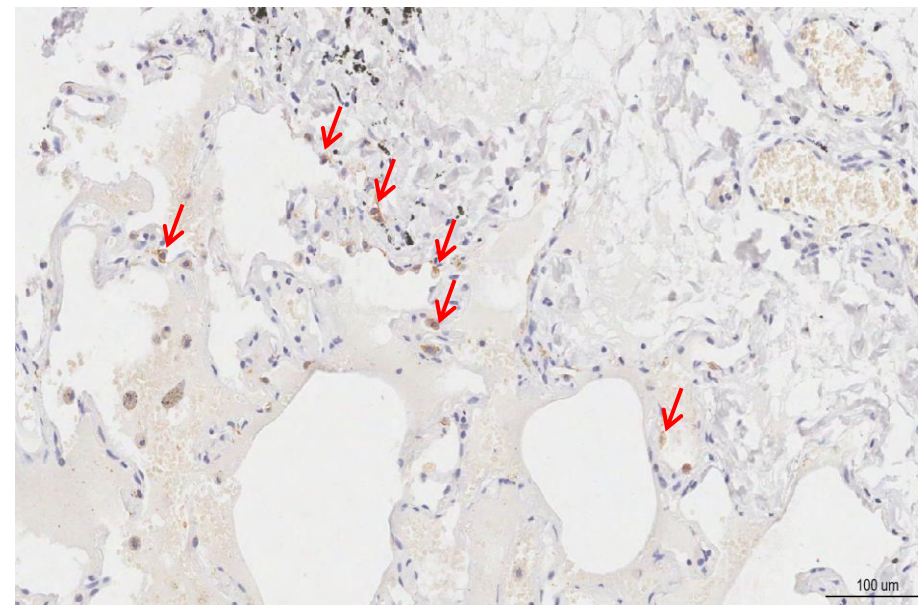

TS\_L\_P4

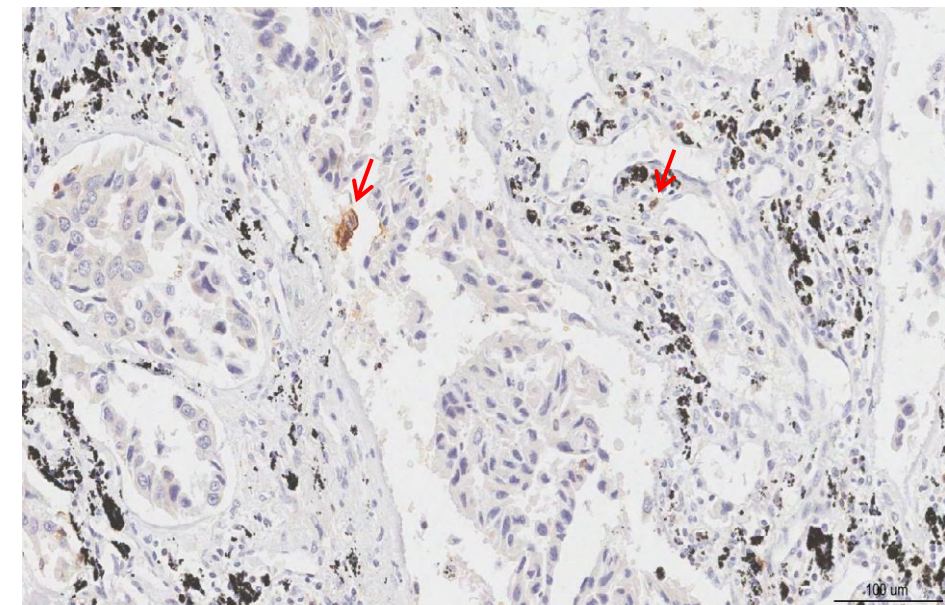

Supplement: Supplementary file 5 — Supplementary Data 1 [file 41419_2023_5992_MOESM5_ESM.pdf]
